# Transannular Approach to 2,3-Dihydropyrrolo[1,2-*b*]isoquinolin-5(1*H*)-ones through Brønsted Acid-Catalyzed Amidohalogenation

**DOI:** 10.1021/acs.joc.2c01045

**Published:** 2022-07-26

**Authors:** Estefanía Capel, Javier Luis-Barrera, Ana Sorazu, Uxue Uria, Liher Prieto, Efraím Reyes, Luisa Carrillo, Jose L. Vicario

**Affiliations:** Department of Organic and Inorganic Chemistry, University of the Basque Country (UPV/EHU), P.O. Box 644, 48080 Bilbao, Spain

## Abstract

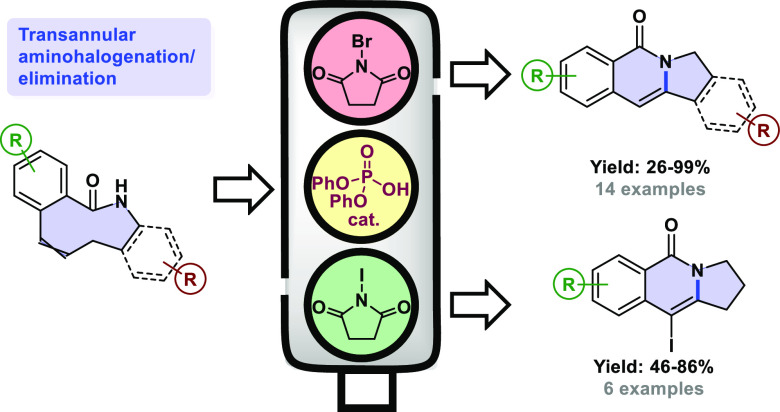

A transannular approach has been developed for the construction of pyrrolo[1,2-*b*]isoquinolinones starting from benzo-fused nine-membered enelactams. This process takes place in the presence of a halogenating agent and under Brønsted acid catalysis and proceeds via a transannular amidohalogenation, followed by elimination. The reaction has been found to be wide in scope, enabling the formation of a variety of tricyclic products in good overall yield, regardless of the substitution pattern in the initial lactam substrate. The reaction has also been applied to the total synthesis of a reported topoisomerase I inhibitor and to the formal synthesis of rosettacin. Further extension of this methodology allows the preparation of 10-iodopyrrolo[1,2-*b*]isoquinolinones by using an excess of halogenating agent and these compounds can be further manipulated through standard Suzuki coupling chemistry into a variety of 10-aryl-substituted pyrrolo[1,2-*b*]isoquinolinones.

## Introduction

**Scheme 1:**
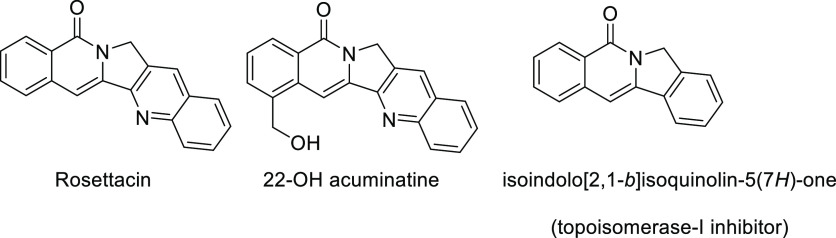
Representative Examples of Bioactive Molecules Containing the 2,3-Dihydropyrrolo[1,2-*b*]isoquinolin-5(1*H*)-one Scaffold

The 2,3-dihydropyrrolo[1,2-*b*]isoquinolin-5(1*H*)-one framework constitutes the central core of several families of bioactive compounds, some of them with relevant therapeutic potential (see Scheme 1).^1^ In particular, this molecular architecture is the main structural feature of aromathecins, a family of topoisomerase I inhibitors that constitute promising chemotherapeutic agents against cancer, with some members already even approved for clinical uses.^2^ In addition, some other members of this family have been identified as highly active antiparasitic compounds which are able to selectively inhibit the phylogenetically unique topoisomerase IB present in the protozoan parasites *Trypanosoma brucei*, *Trypanosoma cruzi*, and the Leishmania species that cause African sleeping sickness (African trypanosomiasis) and Chagas disease (American trypanosomiasis). Moreover, isoindolo[2,1-*b*]isoquinolin-5(7*H*)-one, which also shares the same pyrroloisoquinoline motif has shown to exhibit similar topoisomerase I activity to camptothecin.^3^ Despite this promising activity, the use of aromathecin derivatives in clinical trials still suffers from poor solubility and dose-limiting toxicity,^4^ and consequently, there is still a constant need for the development of effective protocols that enable the construction of this scaffold with a special interest on those strategies that provide a diversity-oriented synthesis approach.

**Scheme 2:**
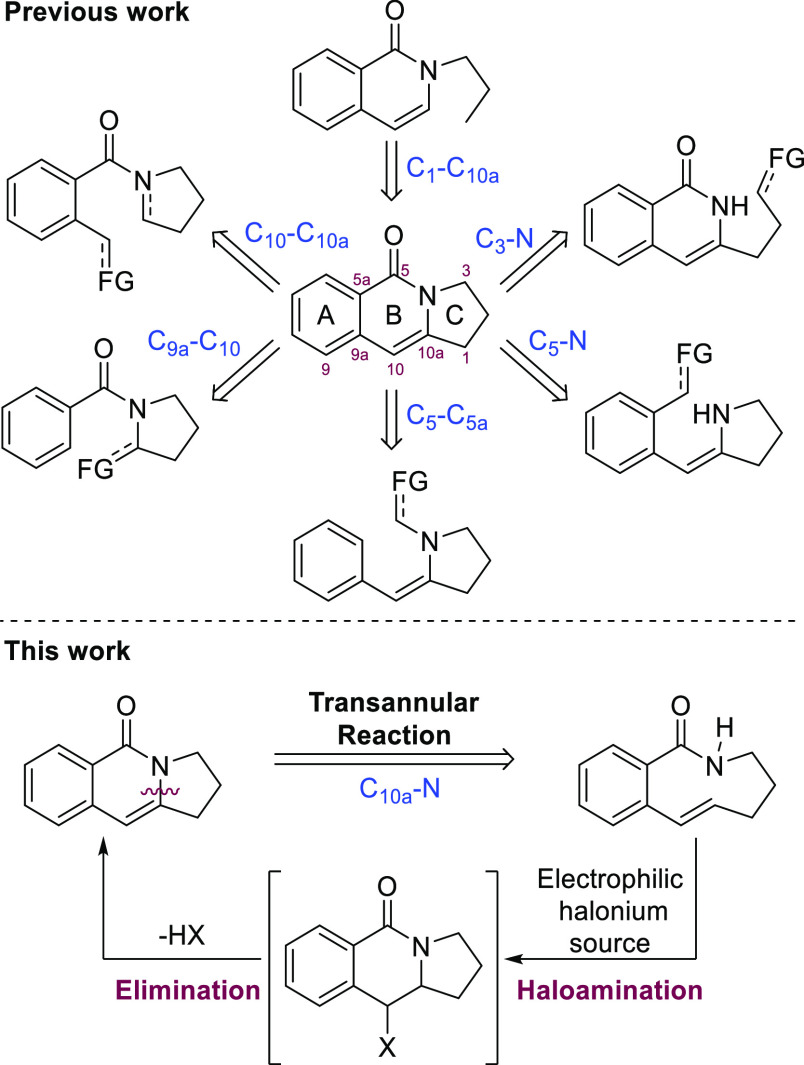
Strategic Disconnections to the 2,3-Dihydropyrrolo[1,2-*b*]isoquinolin-5(1*H*)-one Core

In general, the synthetic routes described up to date for the formation of the 2,3-dihydropyrrolo[1,2-*b*]isoquinolin-5(1*H*)-one scaffold involve the generation of the B and/or C rings through cyclization or cyclocondensation reactions. In all these cases, the retrosynthetic design involves disconnections of the C_10_–C_10a_,^5^ C_3_–N,^6^ C_5_–N,^7^ C_5_–C_5a_,^8^ C_9a_–C_10_,^9^ or C_1_–C_10a_^10^ (see Scheme 2), either individually or by combining different reactions in a cascade process that involves the simultaneous formation of more than one of these bonds at a time.^11^ Similar strategies have been taken for the synthesis of the isoindolo[2,1-*b*]isoquinolin-5(7*H*)-one core.^12^ As an alternative, we propose herein an unconventional and yet unexplored approach to this scaffold that comprises the generation of the C_10a_–N bond *via* formal oxidative transannular amido functionalization of a benzo-fused medium-sized unsaturated lactam, as shown in Scheme 2. In particular, we have directed our attention to study the transannular amidohalogenation reaction that should eventually provide a 10-halo-substituted pyrroloisoquinolin-5-one intermediate, this being a direct precursor of the target 2,3-dihydropyrrolo[1,2-*b*]isoquinolin-5(1*H*)-one upon the in situ elimination process. Transannular reactions, in which the two reacting sites are located within the cyclic structure of the starting material, have been widely used as a key step in the design of efficient syntheses of rather complex molecular scaffolds,^13^ including several examples of elegant total syntheses of natural products.^14^ In most cases, the transannular approach has demonstrated its performance as a key strategic decision that reduces the number of steps involved in the synthetic route and/or enables highly efficient stereocontrol due to the limited degree of conformational freedom associated with the medium- or large-size cyclic starting material, the latter effect also being exploited for the development of several enantioselective variants.^15^ Specifically, there are several precedents of highly effective transannular aminohalogenation or amidohalogenation reactions employed for the synthesis of bicyclic nitrogen-containing heterocycles such as pyrrolizidines,^16^ indolizidines,^17^ and related derivatives.^18^ Despite all progress in this field, the transannular approach has not been already employed for the construction of the isoquinoline core.

## Results and Discussion

The synthesis of the starting benzo-fused lactam **5a** required for the transannular reaction was accomplished by employing standard methodologies through the pathway shown in Table 1. Starting from methyl *o*-bromobenzoate (entry 1), Sonogashira coupling with 5-chloropent-1-yne delivered functionalized alkyne **1a** in excellent yield, which was subsequently subjected to semihydrogenation under Lindlar catalysis, followed by standard Gabriel synthesis, leading to amine **4a**, the latter undergoing lactamization upon treatment with a strong bulky base such as LiHMDS. This synthetic route provided the key lactam **5a** in a very straightforward way (five steps from cheap and readily available starting materials) in good overall yield and could be implemented to the multigram scale. Next, we also proceeded to prepare several other lactam substrates through the same methodological approach, starting from *o*-bromobenzoates and incorporating other substituents in different positions. As it can be seen in Table 1, most compounds **5b-f** could be obtained with good overall yields (entries 2–6).

**Table 1:**
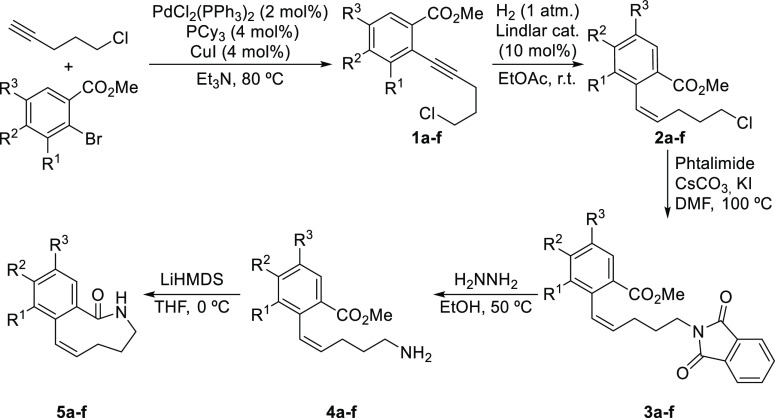
Synthesis of Lactam Precursors **5**

entry	R^1^	R^2^	R^3^	yield **1a–f** (%)^a^	yield **2a–f** (%)^a^	yield **3a–f** (%)^a^	yield **4a–f** (%)^a^	yield **5a–f** (%)^a^
1	H	H	H	93 (**1a**)	91 (**2a**)	87 (**3a**)	74 (**4a**)	50 (**5a**)
2	H	F	H	97 (**1b**)	94 (**2b**)	82 (**3b**)	76 (**4b**)	73 (**5b**)
3	H	H	F	94 (**1c**)	81 (**2c**)	79 (**3c**)	67 (**4c**)	44 (**5c**)
4	H	H	MeO	91 (**1d**)	55 (**2d**)	94 (**3d**)	71 (**4d**)	43 (**5d**)
5	H	H	Me	94 (**1e**)	92 (**2e**)	92 (**3e**)	60 (**4e**)	67 (**5e**)
6	Me	H	H	76 (**1f**)	98 (**2f**)	77 (**3f**)	48 (**4f**)	86 (**5f**)

a Yield of pure products after flash column chromatography purification.

We next proceeded to evaluate the best conditions for the key transannular amidohalogenation/elimination process to take place with the highest possible yield and regioselectivity (Table 2). The initial reaction using *N*-bromosuccinimide (NBS) as the electrophilic halogenation reagent provided the desired transannular amidobromination/elimination product **6a** after stirring for 1 h in toluene in an acceptable 78% yield (entry 1). Based on the known ability of Brønsted acids to accelerate this type of electrophilic aminohalogenation reaction, we surveyed the possibility of improving this result by using a Brønsted acid-catalyzed version of this transformation (entries 2–5). Indeed, the reaction in the presence of 10 mol % of trifluoroacetic acid took place faster (complete conversion of the starting material was observed after 30 min) but with a slightly lower yield (entry 2), but moving to the more acidic diphenylphosphoric acid resulted in a very clean and effective reaction (entry 3). Increasing the acidity of the catalyst to the corresponding triflamide was also effective, but the yield of the reaction was somewhat affected, observing the formation of several minor side products whose structure could not be elucidated (entry 4). Sulfonic acids result in lower yields for the same reaction (entry 5). We next evaluated other parameters of the reaction, such as the solvent or the temperature (entries 6–11). Carrying out the reaction in THF led to a rather sluggish reaction (entry 6), with a notable decrease in the yield of the process, but moving to acetonitrile resulted in a very high yield of the desired adduct **6a** (entry 7). Other halogenated solvents were also tested (entries 8–10), observing that, in general, the reaction performed well in all cases, with the exceptional case of dichloromethane, which resulted in an almost quantitative formation of the transannular aminohalogenation/elimination product (entry 8). We tried to accelerate the reaction by working at a higher temperature (entry 11), but in this case, even though complete consumption of the starting material could be observed after 5 min, product **6a** was isolated in poor yield as a result of the formation of many side products. Catalyst loading could be lowered down to 2.5 mol % without affecting the performance of the reaction (entry 12) and only requiring 1 h for the reaction to reach completion. When we attempted to work with 1 mol % of the Brønsted acid catalyst, the reaction was also very efficient, although it proceeded rather slowly (entry 13). Finally, we also checked the beneficial effect of the Brønsted acid catalyst under these conditions, observing that in the absence of diphenylphosphoric acid, the reaction required a very long time to reach completion and also provided a very low yield of adduct **6a** (entry 14).

**Table 2:**
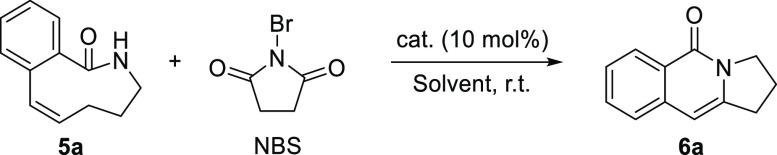
Optimization of the Reaction Conditions for the Transannular Amidobromination/Elimination Process Using **5a** as the Model Compound

entry	catalyst	solvent	time (min)	yield (%)^a^
1	none	toluene	60	78
2	TFA	toluene	30	67
3	(PhO)_2_P(O)OH	toluene	30	90
4	(PhO)_2_P(O)NHTf	toluene	15	66
5	(±)-CSA	toluene	20	67
6	(PhO)_2_P(O)OH	THF	30	36
7	(PhO)_2_P(O)OH	MeCN	30	85
8	(PhO)_2_P(O)OH	CH_2_Cl_2_	30	96
9	(PhO)_2_P(O)OH	CHCl_3_	30	83
10	(PhO)_2_P(O)OH	DCE	30	73
11^b^	(PhO)_2_P(O)OH	CH_2_Cl_2_	5	54
12^c^	(PhO)_2_P(O)OH	CH_2_Cl_2_	60	98
13^d^	(PhO)_2_P(O)OH	CH_2_Cl_2_	120	83
14	none	CH_2_Cl_2_	300	39

a Yield of pure product **6a** after flash column chromatography purification.

b Reaction carried out at 50 °C.

c Reaction carried out using 2.5 mol % of the catalyst.

d Reaction carried out using 1 mol % of the catalyst.

**Scheme 3:**
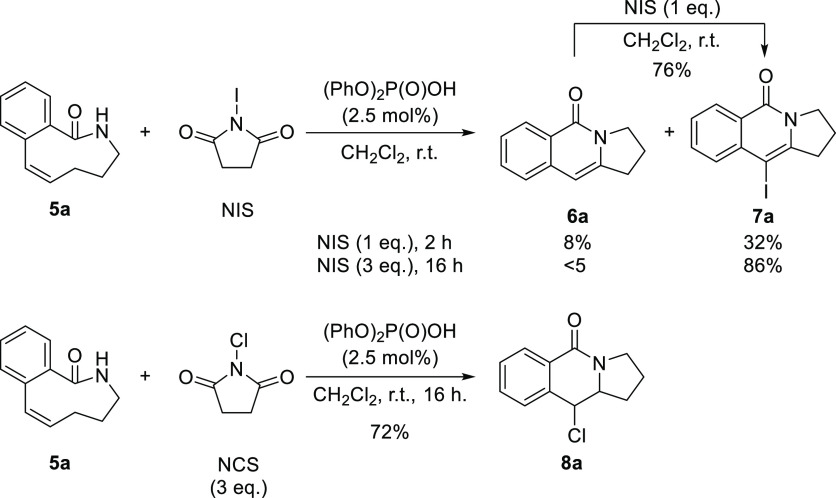
Transannular Amidoiodination/Elimination and Amidochlorination Reactions on Model Substrate **5a**

Remarkably, when we tested these reaction conditions on substrate **5a** but employing *N*-iodosuccinimide (NIS) as the halogenating reagent, product **6a** was formed in low yield (Scheme 3) and the formation of iodinated adduct **7a** was detected as the major product of the reaction, this being presumably generated after electrophilic iodination of the former. This was checked by treating isolated **6a** with 1 equiv of NIS, observing the formation of **7a** in a 76% yield after 24 h. This interesting new compound **7a** was obtained in excellent yield from **5a** by carrying out the reaction in the presence of 3 equiv of NIS. The reaction using *N*-chlorosuccinimide as the halogenating agent provided compound **8a** in a 72% yield, which is the precursor to **6a** in which elimination had not taken place.

With an optimized protocol in hand, we decided to explore the potential of this methodology to prepare different families of 2,3-dihydropyrrolo[1,2-*b*]isoquinolin-5(1*H*)-one incorporating different substitution patterns (Table 3). As it can be seen in this table, the transannular amidobromination/elimination process leading to adducts **6** proceeded smoothly for all substrates regardless of the nature of the substituent placed at the 7 and 8 positions of the isoquinoline core (entries 1–5), only observing that the reaction required a longer time to reach to completion when electron-donating substituents were placed (entries 4 and 5). In fact, the reaction on substrate **5d** containing a strongly electron-donating substituent such as a methoxy group provided only a 40% yield of the aminohalogenated precursor (together with other unidentified byproducts), and in this case, 1.2 equiv of a tertiary amine base such as DBU had to be added after consumption of the starting material in order to assist the elimination process (entry 4). Placing a substituent at the 9-position was much more challenging for the reaction, presumably because of the increased steric congestion, and adduct **6f** could be only obtained in moderate yield (entry 6), observing the formation of several decomposition products after prolonged reaction times. It should be highlighted that it has been demonstrated that the amidobromination/elimination process could be carried out at a bigger scale, isolating the compound **6b** with an excellent 98% yield starting from 1 mmol of **5b**. Moreover, the applicability of this methodology is corroborated due to the fact that the total synthesis of rosettacin is described in the literature from adduct **6a**.^19^ The amidoiodination/elimination/electrophilic iodination sequence leading to adducts **7a–f** also proceeded efficiently, only observing a decrease in the yield of the reaction for the 9-substituted substrate **5f**, although in this case, the unreacted starting material could be recovered intact (entry 6).

**Table 3:**
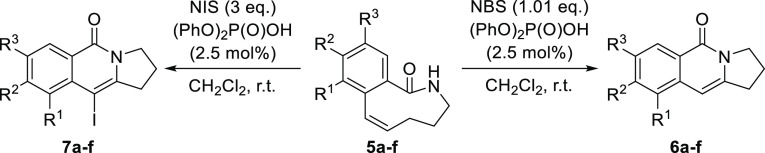
Brønsted Acid-Catalyzed Transannular Aminohalogenation on Lactams **5a–f**

entry	R^1^	R^2^	R^3^	compd	yield **6a–f** (%)^a^	compd	yield **7a–f** (%)^a^
1	H	H	H	**6a**	98 (40 min)	**7a**	86 (16 h)
2	H	F	H	**6b**	98 (60 min)	**7b**	86 (16 h)
3	H	H	F	**6c**	68 (60 min)	**7c**	81 (16 h)
4	H	H	MeO	**6d**^b^	41 (21 h)	**7d**	71 (23 h)
5	H	H	Me	**6e**	79 (3 h)	**7e**	85 (16 h)
6	Me	H	H	**6f**^b^	26 (72 h)	**7f**	46^c^ (72 h)

a Yield of pure products after flash column chromatography purification.

b 1.2 equiv of DBU was added after complete conversion of the starting material had been observed.

c 98% yield based on the recovered starting material.

**Scheme 4:**
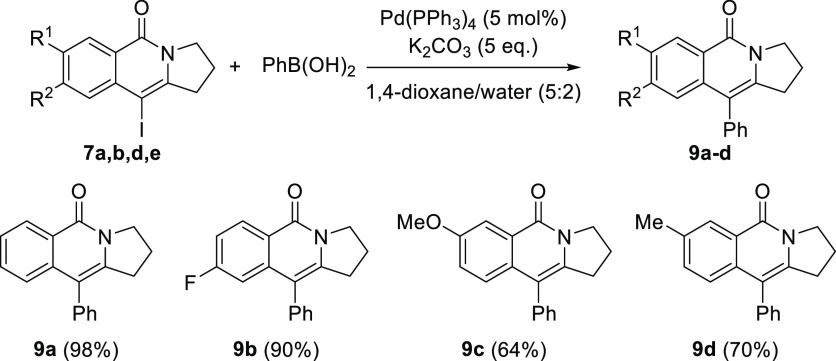
Suzuki Coupling on 3-Iodo-Substituted Substrates **7a–e**

In addition, substrates **7** are suitable to be further diversified by capitalizing the vinyl iodide moiety present in their structure and their potential to undergo Suzuki coupling with aryl boronates under Pd-catalysis. In order to illustrate this possibility, several of these substrates **7** were reacted with phenylboronic acid under standard Suzuki coupling conditions, providing the corresponding 10-aryl-substituted 2,3-dihydropyrrolo[1,2-*b*]isoquinolin-5(1*H*)-ones **9a–d** in excellent yields (Scheme 4).

On the other hand, we also faced the possibility of using this approach to the synthesis of isoindolo[2,1-*b*]isoquinolin-5(7*H*)-ones, which are well-known topoisomerase I inhibitors (see Scheme 1 for one example). We started with the synthesis of the key macrocyclic lactam precursor **12a**, which was accomplished by initial amide formation between differently substituted *o*-halobenzylamines and *o*-vinylbenzoates to form compounds **10a–h** in good overall yields after protection as the corresponding *N*-Boc derivatives. These were subjected subsequently to the intramolecular Pd-catalyzed Heck reaction that took place with complete diastereoselectivity to deliver (*E*)-configured lactams **12a–h** after *N*-deprotection under standard conditions.^20^ This approach for the formation of the medium-sized lactam moiety was especially successful when arylamide **10**-incorporated electron-donating substituents on any of the aryl moieties, providing significantly lower yields in the intramolecular Heck reactions in the cases in which fluorine or chlorine atoms were installed. Once the key lactams **12a–h** had been prepared, these were submitted to the transannular amidobromination/elimination process under the standard conditions. However, in an initial attempt, the reaction provided the amidohalogenated product in which elimination had not taken place. This situation was solved by adding 1 equiv of a Brønsted base such as DBU together with NaI; the latter was required to promote a Finkelstein-type process that inverted the relative configuration of the amidohalogenation product to facilitate the elimination reaction through the E2 process.^21^ As it can be seen in Table 4, isoindolo[2,1-*b*]isoquinolin-5(7*H*)-one **13a**, which had been reported to be a highly active topoisomerase I inhibitor was formed in excellent yield from lactam **12a** under these conditions. In addition, the other lactams **12b–h** prepared were also converted into isoindolo[2,1-*b*]isoquinolin-5(7*H*)-ones **13b–h** through this transannular process in excellent yields regardless of the substitution patterns in any of the aryl moieties.

**Table 4:**
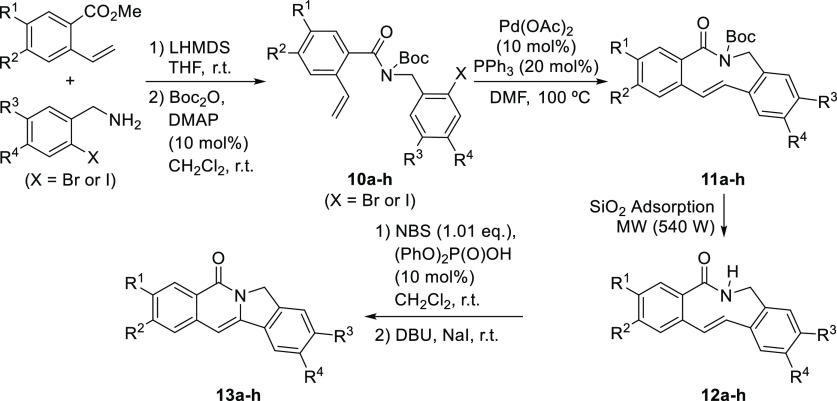
Synthesis of Isoindolo[2,1-*b*]isoquinolin-5(7*H*)-ones

entry	R^1^	R^2^	R^3^	R^4^	yield **10a–f** (%)^a^	yield **11a–f** (%)^a^	yield **12a–f** (%)^a^	yield **13a–f** (%)^a^
1	H	H	H	H	72 (**10a**)	60 (**11a**)	63 (**12a**)	91 (**13a**)
2	F	H	H	H	68 (**10b**)	26 (**11b**)	80 (**12b**)	83 (**13b**)
3	H	F	H	H	59 (**10c**)	16 (**11c**)	58 (**12c**)	74 (**13c**)
4	Cl	H	H	H	51 (**10d**)	23 (**11d**)	62 (**12d**)	96 (**13d**)
5	Me	H	H	H	67 (**10e**)	68 (**11e**)	59 (**12e**)	92 (**13e**)
6	OMe	H	H	H	62 (**10f**)	64 (**11f**)	69 (**12f**)	45 (**13f**)^b^
7	H	H	H	F	55 (**10g**)	45 (**11g**)	74 (**12g**)	99 (**13g**)
8	H	H	Me	H	75 (**10h**)	92 (**11h**)	66 (**12h**)	82 (**13h**)

a Yield of pure products after flash column chromatography purification.

b Addition of the NaI/DBU system was not necessary for performing the elimination step.

In conclusion, we have demonstrated that nine-membered enelactams can be used as useful starting materials for the preparation of differently substituted pyrrolo[1,2-*b*]isoquinolin-5(1*H*)-ones using a transannular approach as the key transformation for the construction of the tricyclic heterocyclic scaffold. This crucial transannular reaction consists of a Brønsted acid-catalyzed amidohalogenation process, followed by elimination, and provides the target products in good yields regardless of the substitution pattern in the cyclic lactam. Moreover, when the reaction is carried out in the presence of an excess of NIS as the electrophilic halogen source, a second halogenation reaction took place on the obtained adducts, furnishing the corresponding iodinated derivatives in good yields, with these compounds having a convenient functionality to be further functionalized through Suzuki coupling chemistry. The protocol described herein has been successfully applied to the preparation of the topoisomerase I inhibitor isoindolo[2,1-*b*]isoquinolin-5(7*H*)-one and several other related compounds, also including the formal total synthesis of rosettacin.

## Experimental Section

### General Methods and Materials

Analytical-grade solvents and commercially available reagents were purchased from commercial sources and used without further purification. Anhydrous solvents were purified and dried with activated molecular sieves prior to use.^22^ For reactions carried out under inert conditions, argon was previously dried through a column of P_2_O_5_ and a column of KOH and CaCl_2_. All the glassware was dried for 12 h prior to use in an oven at 140 °C and allowed to cool under a dehumidified atmosphere. Reactions at reduced temperatures were carried out using a Thermo Haake EK90 refrigerator. Reactions were monitored using analytical thin-layer chromatography (TLC) in precoated silica-backed plates (Merck Kiesegel 60 F254). These were visualized by ultraviolet irradiation, *p*-anisaldehyde, phosphomolybdic acid, or potassium permanganate dips.^23^ For flash chromatography, Silicycle 40–63 and 230–400 mesh silica gel was used.^24^ Monodimensional and/or bidimensional nuclear magnetic resonance proton and carbon spectra (^1^H NMR and ^13^C {^1^H} NMR) were acquired at 25 °C on a Bruker AC-300 spectrometer (300 MHz for ^1^H, 75.5 MHz for ^13^C and 282 MHz for ^19^F) and a Bruker AC-500 spectrometer (500 MHz for ^1^H and 125.7 MHz for ^13^C) at the indicated temperature. Chemical shifts (δ) are reported in parts per million relative to residual solvent signals (CHCl_3_, 7.26 ppm for ^1^H NMR, CDCl3, 77.16 ppm for ^13^C {^1^H} NMR) and coupling constants (*J*) in hertz (Hz). The following abbreviations are used to indicate the multiplicity in NMR spectra: s, singlet; d, doublet; t, triplet; q, quartet; p, pentet; app, apparent; m, multiplet; bs, broad signal. ^13^C {^1^H} NMR spectra were acquired on a broad-band decoupled mode using distortion-less enhancement by polarization transfer experiments for assigning different types of carbon environments. Assignments were made based upon the IUPAC numbering system. Mass spectra were recorded on an Agilent 7890 A gas chromatograph coupled to an Agilent 5975 C quadrupole mass spectrometer under electronic impact (EI) ionization at 70 eV. The obtained data is presented in mass units (*m*/*z*), and the values found in brackets belong to the relative intensities compared to the base peak (100%). High-resolution mass spectra were recorded on an Acquity UPLC system coupled to a quadrupole time-of-flight mass spectrometer (SYNAPT G2 HDMS) using electrospray ionization (ESI^+^). Infrared (IR) spectra were measured in a Jasco FT/IR 4100 (ATR), in the interval between 4000 and 400 cm^–1^ with a 4 cm^–1^ resolution. Only characteristic bands are given in each case. Melting points (mp) were measured using a Buchi B-540 apparatus in open capillary tubes and were uncorrected.

### Procedure for the Synthesis of Methyl 2-(5-Chloropent-1-yn-1-yl)benzoate (**1a**)

In an oven-dried, two-necked round-bottom flask equipped with a condenser and stir bar with methyl 2-bromobenzoate (9 mL, 64 mmol), PdCl_2_(PPh_3_)_2_ (0.898 g, 1.28 mmol), PCy_3_ (0.718 g, 2.56 mmol), and CuI (0.49 g, 2.56 mmol) in freshly distilled Et_3_N (256 mL) under an Ar atmosphere, 5-chloropent-1-yne (10.6 mL, 95.5 mmol) was added. The mixture was heated using a heating plate to 80 °C over 18 h. Then, the reaction mixture was cooled to room temperature and was filtrated through a plug of Celite. Aq HCl 1 M (10 mL) was added to the filtrate, and the organic layer was extracted with EtOAc (3 × 10 mL). All the organic layers were washed with aq std. NaHCO_3_ (10 mL), dried over Na_2_SO_4_, and concentrated under vacuum. The crude was purified by silica gel flash chromatography (petroleum ether/EtOAc 19:1), obtaining **1a** (13.3 g, 60 mmol, 93%) as a yellow oil. *R*_*f*_: 0.6 (petroleum ether/EtOAc 9:1). ^1^H NMR (300 MHz, CDCl_3_): δ 7.88 (ddd, *J* = 7.8, 1.5, 0.6 Hz, 1H), 7.49 (dd, *J* = 7.8, 1.2 Hz, 1H), 7.41 (td, *J* = 7.5, 1.5 Hz, 1H), 7.31 (td, *J* = 7.6, 1.5 Hz, 1H), 3.90 (s, 3H), 3.77 (t, *J* = 6.4 Hz, 2H), 2.66 (t, *J* = 6.7 Hz, 2H), 2.07 (p, *J* = 6.6 Hz, 2H). ^13^C {^1^H} NMR (75 MHz, CDCl_3_): δ 166.9 (C), 134.3 (CH), 132.0 (C), 131.6 (CH), 130.3 (CH), 127.5 (CH), 124.1 (C), 93.7 (C), 80.3 (C), 52.2 (CH_3_), 43.8 (CH_2_), 31.5 (CH_2_), 17.3 (CH_2_). IR (ATR, cm^–1^): 2264 (C≡C), 1726 (C=O). MS (EI) *m*/*z* (%): 174 (100, M^+^ – CH_3_CH_2_Cl), 159 (21, M^+^ – CH_3_CH_2_CH_2_Cl), 115 (21, M^+^ – CO_2_Me–CH_3_CH_2_Cl). HRMS (ESI) *m*/*z*: [M + H]^+^ calcd for [C_13_H_14_ClO_2_]^+^, 237.0677; found, 237.0689 for compound: **1a**.

### Procedure for the Synthesis of Methyl (*Z*)-2-(2-(5-Chloropent-1-en-1-yl)benzoate) (**2a**)

To a two-necked round-bottom flask equipped with a stirring bar and H_2_ balloon with methyl 2-(5-chloropent-1-yn-1-yl)benzoate **1a** (3 g, 12.7 mmol) in EtOAc (127 mL) and quinoline (0.12 mL, 1.02 mmol, 8 mol %), Pd on CaCO_3_ (1.35 g, 0.63 mmol, 5 mol %) was added. The air flask was evacuated under vacuum and backfilled with hydrogen three times, and the reaction mixture was allowed to stir at room temperature under a hydrogen atmosphere (balloon pressure) until full consumption of the starting material, 1 h as judged by TLC. The reaction mixture was filtered through a plug of Celite, and the filtrate was concentrated under vacuum. The crude was purified by silica gel flash chromatography (petroleum ether/EtOAc 19:1) obtaining **2a** (3.0 g, 11.6 mmol, 91%) as a yellow oil. *R*_*f*_: 0.62 (petroleum ether/EtOAc). ^1^H NMR (300 MHz, CDCl_3_): δ 7.95 (dd, *J* = 7.8, 1.2 Hz, 1H), 7.48 (td, *J* = 7.5, 1.3 Hz, 1H), 7.36–7.28 (m, 2H), 6.92 (d, *J* = 11.5 Hz, 1H), 5.67 (dt, *J* = 11.5, 7.4 Hz, 1H), 3.87 (s, 3H), 3.49 (t, *J* = 6.7 Hz, 2H), 2.27 (m, 2H), 1.92–1.78 (m, 2H). ^13^C {^1^H} NMR (75 MHz, CDCl_3_): δ 167.6 (C), 138.9 (C), 131.8 (CH), 130.8 (CH), 130.6 (CH), 130.5 (CH), 129.8 (CH), 129.4 (C), 127.0 (CH), 52.0 (CH_3_), 44.5 (CH_2_), 32.7 (CH_2_), 25.6 (CH_2_). IR (ATR, cm^–1^): 1720 (C=O). MS (EI) *m*/*z* (%): 238 (M^+^, 11), 161 (M^+^ – CH_3_CH_2_Cl–CH_3_, 100), 128 (M^+^ – CH_3_Cl–CO_2_Me, 35), 115 (M^+^ – CH_3_CH_2_Cl–CO_2_Me, 68). HRMS (ESI) *m*/*z*: [M + H]^+^ calcd for [C_13_H_16_ClO_2_]^+^, 239.0833; found, 239.0838 for compound: **2a**.

### Procedure for the Synthesis of Methyl (*Z*)-2-(5-(1,3-Dioxoisoindolin-2-yl)pent-1-en-1-yl)benzoate (**3a**)

An oven-dried two-necked round-bottom flask provided with a condenser and a magnetic bar with a suspension of methyl (*Z*)-2-(2-(5-chloropent-1-en-1-yl)benzoate) **2a** (2.9 g, 12.2 mmol), Cs_2_CO_3_ (8.73 g, 26.8 mmol), phthalimide (2.69 g, 18.3 mmol), and KI (20.5 mg, 0.122 mmol) in DMF (70 mL) under an Ar atmosphere was heated using a heating plate at 100 °C for 2 h. Then, the reaction mixture was let to cool down to room temperature and water (100 mL) and EtOAc (50 mL) was added. The aqueous layer was extracted with EtOAc (3 × 25 mL), washed with H_2_O (2 × 75 mL), dried with Na_2_SO_4_, filtered and concentrated under vacuum. The crude was purified by silica gel flash chromatography (petroleum ether/EtOAc 9:1 to petroleum ether/EtOAc 8:2) in order to obtain **3a** (3.7 g, 10.6 mmol, 87%) as a white solid. *R*_*f*_: 0.5 (petroleum ether/EtOAc 8:2). mp 69–71 °C. ^1^H NMR (300 MHz, CDCl3): δ 7.89 (dd, *J* = 7.8, 1.0 Hz, 1H), 7.80 (dd, *J* = 5.5, 3.0 Hz, 2H), 7.68 (dd, *J* = 5.3, 3.2 Hz, 2H), 7.40 (td, *J* = 7.5, 1.2 Hz, 1H), 7.29–7.19 (m, 2H), 6.88 (d, *J* = 11.5 Hz, 1H), 5.72 (dt, *J* = 11.6, 7.4 Hz, 1H), 3.85 (s, 3H), 3.66–3.58 (m, 2H), 2.23–2.09 (m, 2H), 1.83–1.69 (m, 2H). ^13^C {^1^H} NMR (75 MHz, CDCl_3_): δ 168.5 (2xC), 167.8 (C), 138.8 (C), 134.0 (2xCH), 132.3 (2× C), 131.7 (CH), 130.7 (CH), 130.6 (CH), 130.4 (CH), 130.0 (CH), 129.5 (C), 126.9 (CH), 123.3 (2× CH), 52.1 (CH_3_), 37.8 (CH_2_), 28.7 (CH_2_), 25.8 (CH_2_). IR (ATR, cm^–1^): 1771 (O=C–N–C=O), 1708 (C=O). MS (EI) *m*/*z* (%): 317 (M^+^ – MeOH, 37), 170 (M^+^ – MeOH–Phth, 100). HRMS (ESI) *m*/*z*: [M + H]^+^ calcd for [C_21_H_20_NO_4_]^+^, 350.1387; found, 350.1390 for compound: **3a**.

### Procedure for the Synthesis of Methyl (*Z*)-2-(5-Aminopent-1-en-1-yl)benzoate (**4a**)

A round-bottom flask equipped with a magnetic bar was provided with (*Z*)-2-(5-(1,3-dioxoisoindolin-2-yl)pent-1-en-1-yl)benzoate **3a** (3.55 g, 10.2 mmol) and hydrazine (50% w/w in water, 1.4 mL, 28.6 mmol) in EtOH (51 mL). The reaction mixture was heated using a heating plate at 50 °C for 2 h. Then, the reaction mixture was let to cool down to room temperature and was filtered through a plug of Celite The filtrate was concentrated under reduced pressure, and 1 M HCl (50mL) was added; the aqueous layer was washed with EtOAc (3 × 25 mL), basified with 4 M NaOH to pH = 9, extracted with CH_2_Cl_2_ (4 × 25 mL), dried with Na_2_SO_4_, filtered, and concentrated under vacuum to obtain **4a** (1.66 g, 7.5 mmol, 74%) as a yellow oil. *R*_*f*_: 0.2 (MeOH). ^1^H NMR (300 MHz, CDCl_3_): δ 7.96 (d, *J* = 7.8 Hz, 1H), 7.49 (t, *J* = 7.5 Hz, 1H), 7.37–7.28 (m, 2H), 6.90 (d, *J* = 11.6 Hz, 1H), 5.74 (dt, *J* = 11.6, 7.4 Hz, 1H), 3.90 (s, 3H), 2.68 (t, *J* = 6.5 Hz, 2H), 2.19 (q, *J* = 7.4 Hz, 2H), 1.57 (p, *J* = 7.2 Hz, 2H), 1.36 (br s, 2H). ^13^C {^1^H} NMR (75 MHz, CDCl3): δ 168.1 (C), 139.2 (C), 132.0 (CH), 131.4 (CH), 131.1 (CH), 130.8 (CH), 129.9 (C), 129.7 (CH), 127.2 (CH), 52.4 (CH_3_), 41.3 (CH_2_), 32.4 (CH_2_), 26.0 (CH_2_). IR (ATR, cm^–1^): 1719 (C=O), 1636 (N–H). MS (EI) *m*/*z* (%): 219 (M^+^, 10), 202 (M^+^ – NH_2_, 59), 161 (M^+^ – CH_3_–CH_3_CH_2_NH_2_, 47), 115 (M^+^ – CO_2_Me–CH_3_CH_2_NH_2_, 100). HRMS (ESI) *m*/*z*: [M + H]^+^ calcd for [C_13_H_18_NO_2_]^+^, 220.1332; found, 220.1340 for compound: **4a**.

### Procedure for the Synthesis of (*Z*)-2,3,4,5-Tetrahydro-1*H*-benzo[*c*]azonin-1-one (**5a**)

To an oven-dried two-necked round-bottom flask provided with a magnetic bar with methyl (*Z*)-2-(5-aminopent-1-en-1-yl)benzoate **4a** (0.5 g, 2.3 mmol) in dry THF (115 mL) under an Ar atmosphere, LiHMDS 1 M in THF (6.9 mL, 6.9 mmol) was added dropwise at 0 °C. The reaction mixture was stirred at room temperature and monitored by TLC. Once the starting material (SM) was consumed, MeOH (1 mL) was added, and the solvent was removed under vacuum. The residue was dissolved in CH_2_Cl_2_ and filtrated through Celite. The filtrate was concentrated under vacuum and purified by silica gel flash chromatography (petroleum ether/EtOAc 8:2 to petroleum ether/EtOAc 7:3) to obtain **5a** (0.21 g, 1.1 mmol, 50%) as a white solid. *R*_*f*_: 0.4 (petroleum ether/EtOAc 7:3). mp 183–186 °C. ^1^H NMR (300 MHz, CDCl_3_): δ 7.42–7.27 (m, 3H), 7.17 (d, *J* = 7.0 Hz, 1H), 6.62 (d, *J* = 10.8 Hz, 1H), 6.30 (br s, 1H), 6.00 (dt, *J* = 10.6, 8.4 Hz, 1H), 3.41–2.99 (m, 2H), 2.30–1.78 (m, 2H), 1.51 (app p, *J* = 5.2 Hz, 2H). ^13^C {^1^H} NMR (75 MHz, CDCl_3_): δ 174.0 (C), 137.1 (C), 136.2 (C), 134.3 (CH), 128.7 (CH), 128.7 (CH), 128.3 (CH), 127.3 (CH), 125.2 (CH), 45.4 (CH_2_), 29.3 (CH_2_), 28.9 (CH_2_). IR (ATR, cm^–1^): 3287 (N–H), 1646 (N–C=O). MS (EI) *m*/*z* (%): 187 (M^+^, 60), 158 (M^+^ – CH_3_NH_2_, 100). HRMS (ESI) *m*/*z*: [M + H]^+^ calcd for [C_13_H_17_NO]^+^, 202.1232; found, 202.1235 for compound: **5a**.

### Procedure for the Synthesis of 2,3-Dihydropyrrolo[1,2-*b*]isoquinolin-5(1*H*)-one (**6a**)

To a reaction tube provided with a magnetic bar with (*Z*)-2,3,4,5-tetrahydro-1*H*-benzo[*c*]azonin-1-one **5a** (20 mg, 0.107 mmol), a stock solution of diphenylphosphoric acid (0.7 mg, 0.003 mmol) in dry dichloromethane (220 μL) was added at 25 °C, followed by the addition of NBS (19 mg, 0.108 mmol). The reaction mixture was subjected to TLC, and when all starting material was consumed (typically 40 min), the solvent was evaporated under vacuum, and Et_2_O (1 mL) and a standard. aq solution of NaHCO_3_ (1 mL) were added. The layers were separated, and the aqueous phase was extracted with Et_2_O (3 × 1 mL). All organic layers were washed with water (3 × 1 mL) and brine (1 mL), dried with Na_2_SO_4_, filtered, and concentrated under vacuum. The crude was purified by silica gel flash chromatography (CH_2_Cl_2_ to CH_2_Cl_2_/MeOH 99:1) obtaining **6a** (19 mg, 0.103 mmol, 98%) as a white solid. *R*_*f*_: 0.3 (CH_2_Cl_2_/MeOH 99:1). mp 102–105 °C. ^1^H NMR (300 MHz, CDCl_3_): δ 8.31 (dq, *J* = 8.03, 0.61 Hz, 1H), 7.51 (ddd, *J* = 8.2, 7.0, 1.4 Hz, 1H), 7.38 (d, *J* = 7.8 Hz, 1H), 7.32 (dd, *J* = Hz, 1H), 6.34 (s, 1H), 4.15–4.06 (m, 2H), 3.02 (td, *J* = 7.7, 1.4 Hz, 2H), 2.20–2.04 (m, 2H). ^13^C {^1^H} NMR (75 MHz, CDCl_3_): δ 161.6 (C), 143.7 (C), 138.1 (C), 131.9 (CH), 127.3 (CH), 125.5 (CH), 125.5 (CH), 124.7 (C), 100.3 (CH), 47.9 (CH_2_), 31.3 (CH_2_), 22.0 (CH_2_). IR (ATR, cm^–1^): 2985 (C–H), 1657 (N–C=O), 1624 (C=C). MS (EI) *m*/*z* (%): 184.1 (M^+^, 100). HRMS (ESI) *m*/*z*: [M + H]^+^ calcd for [C_12_H_12_NO]^+^, 186.0919; found, 186.0919 for compound: **6a**.

### Procedure for the Synthesis of 10-Iodo-2,3-dihydropyrrolo[1,2-*b*]isoquinolin-5(1*H*)-one (**7a**)

To a reaction tube provided with a magnetic bar with (*Z*)-2,3,4,5-tetrahydro-1*H*-benzo[*c*]azonin-1-one **5a** (27.4 mg, 0.145 mmol), a stock solution of diphenylphosphoric acid (0.9 mg, 0.004 mmol) in dry dichloromethane (290 μL) was added at 25 °C, followed by the addition of NIS (98 mg, 0.434 mmol). The reaction mixture was subjected to TLC, and when all starting material was consumed (typically 16 h), the solvent was evaporated under vacuum, and Et_2_O (1 mL) and a standard aq solution of Na_2_S_2_O_3_ (1 mL) were added; the mixture was stirred for 30 min at room temperature. Then, the layers were separated, and the aqueous phase was extracted with Et_2_O (3 × 1 mL). All organic layers were washed with water (3 × 1 mL), brine (1 mL), dried with Na_2_SO_4_, filtered, and concentrated under vacuum. The crude was purified by silica gel flash chromatography (CH_2_Cl_2_ to CH_2_Cl_2_/MeOH 99:1) in order to obtain **7a** (38.6 mg, 0.124 mmol, 86%) as a pale-yellow solid. *R*_*f*_: 0.3 (CH_2_Cl_2_/MeOH 99:1). mp 141–143 °C. ^1^H NMR (300 MHz, CDCl_3_): δ 8.37 (dd, *J* = 8.0, 0.9 Hz, 1H), 7.76 (dd, *J* = 8.1, 0.6 Hz, 1H), 7.69 (ddd, *J* = 8.3, 6.9, 1.14 Hz, 1H), 7.47 (ddd, *J* = 8.2, 7.0, 1.2 Hz, 1H), 4.36 (t, *J* = 7.4 Hz, 2H), 3.26 (t, *J* = 7.8 Hz, 2H), 2.25 (p, *J* = 7.5 Hz, 2H). ^13^C {^1^H} NMR (75 MHz, CDCl_3_): δ 160.9 (C), 146.7 (C), 138.0 (C), 133.1 (CH), 129.3 (CH), 127.7 (CH), 126.6 (CH), 124.9 (C), 68.7 (C), 50.2 (CH_2_), 37.5 (CH_2_), 20.8 (CH_2_). IR (ATR, cm^–1^): 1638 (N–C=O), 1609 (C=C). MS (EI) *m*/*z* (%): 311.0 (M^+^, 100). HRMS (ESI) *m*/*z*: [M + H]^+^ calcd for [C_12_H_11_INO]^+^, 311.9885; found, 311.9891 for compound: **7a**.

### Procedure for the Synthesis of 10-Phenyl-2,3-dihydropyrrolo[1,2-*b*]isoquinolin-5(1*H*)-one (**9a**)

An oven-dried 50 mL two-necked flask was charged with 10-iodo-2,3-dihydropyrrolo[1,2-*b*]isoquinolin-5(1*H*)-one **7a** (31.2 mg, 0.1 mmol), phenyl boronic acid (15.9 mg, 0.13 mmol), Pd(PPh_3_)_4_ (5.7 mg, 0.005 mmol), and K_2_CO_3_ (69.1 mg, 0.5 mmol). Under an argon atmosphere, 1,4-dioxane (5 mL) and water (2 mL) were added, and the reaction mixture was subjected to vacuum and refilled with argon three times. The reaction mixture was heated using a heating plate up at 65 °C for 21 h. After completion (typically 24 h), the mixture was cooled to room temperature and diluted with EtOAc (10 mL). The mixture was filtered through a small pad of Celite. Afterward, the solvent was concentrated in vacuum. The resulting crude was purified by flash chromatography (petroleum ether/EtOAc 7:3 to 1:1) in order to obtain **9a** (25.7 mg, 0.098 mmol, 98%) as a yellow solid. *R*_*f*_: 0.27 (petroleum ether/EtOAc 1:1). mp 169–171 °C. ^1^H NMR (300 MHz, CDCl_3_): δ 8.48 (dd, *J* = 7.9, 0.8 Hz, 1H), 7.59–7.34 (m, 5H), 7.36–7.23 (m, 3H), 4.28 (t, *J* = 7.2 Hz, 2H), 2.93 (t, *J* = 7.6 Hz, 2H), 2.15 (p, *J* = 7.5 Hz, 2H). ^13^C {^1^H} NMR (75 MHz, CDCl_3_): δ 161.2 (C), 141.4 (C), 138.2 (C), 136.5 (C), 132.0 (CH), 130.7 (2× CH), 128.8 (2× CH), 127.7 (CH), 127.5 (CH), 125.7 (CH), 125.1 (C), 124.4 (CH), 113.8 (C), 48.6 (CH_2_), 31.2 (CH_2_), 22.0 (CH_2_). IR (ATR, cm^–1^): 1647 (C=O st), 1622 (C_Arom_–C_Arom_ st), 1598 (C_Arom_–C_Arom_ st). MS (EI) *m*/*z* (%): 261.0 (M^+^, 100), 260.1 (67), 165.0 (36), 163.0 (20), 76.9 (26). HRMS (ESI) *m*/*z*: [M + Hc^+^ Calcd for [C_18_H_16_NO]^+^, 262.1226; found, 262.1237 for compound: **9a**.

### Procedure for the Synthesis of *tert*-Butyl(2-iodobenzyl)(2-vinylbenzoyl)carbamate (**10a**)

In the first step, to an oven-dried 100 mL two-necked flask provided with the corresponding methyl 2-vinylbenzoate (1.20 g, 7.40 mmol) and (2-iodophenyl)methanamine (1.81 g, 7.80 mmol) under an Ar atmosphere, LiHMDS in THF 1 M (22.2 mL, 22.2 mmol) was added dropwise at room temperature to the stirring mixture, and it was allowed to stir for 16 h at this temperature. The solvent was evaporated under vacuum and purified by silica gel column chromatography (petroleum ether/EtOAc 8:2) to obtain *N*-(2-iodobenzyl)-2-vinylbenzylamide (2.21 g, 6.08 mmol, 82%) as a solid. *R*_*f*_: 0.53 (petroleum ether/EtOAc 8:2). mp 136–138 °C. ^1^H NMR (300 MHz, CDCl_3_): δ 7.85 (dd, *J* = 7.9, 1.3 Hz, 1H), 7.61–7.23 (m, 6H), 6.28 (br s, 1H), 5.69 (dd, *J* = 17.5, 1.2 Hz, 1H), 5.33 (dd, *J* = 11.0, 1.2 Hz, 1H), 4.65 (d, *J* = 6.0 Hz, 2H). ^13^C {^1^H} NMR (75 MHz, CDCl_3_): δ 169.1 (C), 140.4 (C), 139.7 (CH), 136.3 (C), 135.0 (C), 134.7 (CH), 130.5 (CH), 130.2 (CH), 129.6 (CH), 128.8 (CH), 127.9 (CH), 127.7 (CH), 126.6 (CH), 117.1 (CH_2_), 99.3 (C), 48.9 (CH_2_). IR (ATR, cm^–1^): 3264 (N–H), 1640 (N–C=O), 1525 (C=C). MS (EI) *m*/*z* (%): 363.0 (M^+^, 15), 236.1 (M^+^ – I, 100). HRMS (ESI) *m*/*z*: [M + H]^+^ calcd for [C_16_H_15_INO]^+^, 364.0193; found, 364.0197. In the second step, to an oven-dried 50 mL two-necked flask, *N*-(2-iodobenzyl)-2-vinylbenzamide (1.92 g, 6.08 mmol) and di-*tert*-butyl dicarbonate (2.00 g, 9.12 mmol) under an Ar atmosphere were dissolved in dry CH_2_Cl_2_ (12 mL), and under stirring, DMAP (74 mg, 0.60 mmol) was added at room temperature. The reaction mixture was allowed to stir at room temperature for 16 h. Then, it was concentrated under vacuum and purified by silica gel column chromatography FC (petroleum ether/EtOAc 9:1 to 8:2) in order to obtain **10a** (2.48 g, 5.35 mmol, 88%) (overall yield for the two steps = 72%) as a yellow solid. *R*_*f*_: 0.47 (petroleum ether/EtOAc 8:2). mp 75–76 °C. ^1^H NMR (300 MHz, CDCl_3_): δ 7.76 (dd, *J* = 7.9, 1.3 Hz, 1H), 7.49 (d, *J* = 7.8 Hz, 1H), 7.35–7.16 (m, 4H), 7.10 (dd, *J* = 7.8, 1.6 Hz, 1H), 6.91–6.71 (m, 2H), 5.65 (dd, *J* = 17.4, 1.1 Hz, 1H), 5.28 (dd, *J* = 10.9, 1.1 Hz, 1H), 4.97 (s, 2H), 0.99 (s, 9H). ^13^C {^1^H} NMR (75 MHz, CDCl_3_): δ 172.1 (C), 152.3 (C), 139.5 (CH), 139.4 (C), 137.1 (C), 135.3 (C), 133.8 (CH), 129.7 (CH), 128.7 (CH), 128.4 (CH), 127.3 (CH), 126.3 (CH), 126.2 (CH), 125.8 (CH), 117.0 (CH_2_), 97.7 (C), 83.7 (C), 53.3 (CH_2_), 27.3 (3× CH_3_). IR (ATR, cm^–1^): 1733 (C=O), 1671 (N–C=O). MS (EI) *m*/*z* (%): 362.0 (M^+^, 15). HRMS (ESI) *m*/*z*: [M + Na]^+^ calcd for [C_21_H_22_INO_3_Na]^+^, 486.0537; found, 486.0538 for compound: **10a**.

### Procedure for the Synthesis of *tert*-Butyl(*E*)-5-oxo-5,7-dihydro-6*H*-dibenzo[*c*,*g*]azonine-6-carboxylate (**11a**)

To an oven-dried 250 mL flask provided with *tert*-butyl(2-iodobenzyl)(2-vinylbenzoyl)carbamate **10a** (1.18 g, 2.55 mmol) in dry DMF (12.8 mL) under an Ar atmosphere, sodium acetate (418 mg, 5.10 mmol), triphenylphosphine (67 mg, 0.26 mmol), and palladium acetate (29 mg, 0.13 mmol) were added. The reaction mixture was stirred at 100 °C using a heating plate for 72 h. Then, it was cooled down and water (20 mL) and EtOAc (20 mL) were added; the mixture was filtered through Celite, the filtrate was extracted with EtOAc (4 × 20 mL), and the organic phase was washed with water (3 × 5 mL) and brine (10 mL), dried with Na_2_SO_4_ anhydrous, filtered, and concentrated under vacuum. The crude was purified by silica gel column chromatography FC (petroleum ether/EtOAc 9:1) in order to obtain **11a** (513 mg, 1.53 mmol, 60%) as a yellow solid. *R*_*f*_: 0.45 (petroleum ether/EtOAc 95:5). mp 74–77 °C. ^1^H NMR (300 MHz, CDCl_3_): δ 7.70 (dd, *J* = 7.5, 1.6 Hz, 1H), 7.53–7.41 (m, 2H), 7.40–7.25 (m, 5H), 6.59 (d, *J* = 16.6 Hz, 1H), 6.51 (d, *J* = 16.6 Hz, 1H), 5.41 (d, *J* = 15.8 Hz, 1H), 4.63 (d, *J* = 15.8 Hz, 1H), 1.00 (s, 9H). ^13^C {^1^H} NMR (75 MHz, CDCl_3_): δ 177.8 (C), 151.4 (C), 140.0 (C) 137.8 (C), 137.7 (C), 136.3 (CH), 133.0 (C), 130.1 (CH), 129.5 (CH), 128.3 (CH), 128.1 (CH), 128.0 (CH), 127.9 (CH), 127.5 (CH), 127.3 (CH), 125.1 (CH), 82.0 (C), 52.0 (CH_2_), 27.5 (3× CH_3_). IR (ATR, cm^–1^): 1722 (C=O), 1680 (N–C=O). MS (EI) *m*/*z* (%): 232.9 (M^+^ – Boc). HRMS (ESI) *m*/*z*: [M + Na]^+^ calcd for [C_21_H_21_NNaO_3_]^+^, 358.1414; found, 358.1420 for compound: **11a**.

### Procedure for the Synthesis of (*E*)-6,7-Dihydro-5*H*-dibenzo[*c*,*g*]azonin-5-one (**12a**)

*tert*-Butyl(*E*)-5-oxo-5,7-dihydro-6*H*-dibenzo[*c*,*g*]azonine-6-carboxylate **11a** (67,1 mg, 0.200 mmol) was dissolved in CH_2_Cl_2_ (5 mL), and silica gel (230–400 mesh) (2.00 g) was added. The solvent was vacuumed, and the powdered solid obtained was irradiated in the microwave oven in an open Erlenmeyer flask at 540 W. The reaction was checked by TLC every 6 min until it was completed (typically 30 min). The reaction mixture was deadsorbed by thoroughly washing the silica gel with petroleum ether/EtOAC 1:1 with pressure in a column. The crude was purified by silica gel column chromatography (petroleum ether/EtOAc 8:2 to 7:3) in order to obtain **12a** (29.6 mg, 0.126 mmol, 63%) as a white solid. *R*_*f*_: 0.39 (petroleum ether/EtOAc 9:1). mp 205–208 °C. ^1^H NMR (300 MHz, CDCl_3_): δ 7.66–7.60 (m, 1H), 7.44–7.28 (m, 3H), 7.28–7.17 (m, 3H), 7.17–7.11 (m, 1H), 6.65 (d, *J* = 17.0 Hz, 1H), 6.41 (d, *J* = 17.0 Hz, 1H), 5.38 (dd, *J* = 16.1, 10.7 Hz, 1H), 4.82 (d, *J* = 10.7 Hz, 1H), 4.18 (d, *J* = 16.1 Hz, 1H). ^13^C {^1^H} NMR (75 MHz, CDCl_3_): δ 176.0 (C), 139.1 (C), 138.4 (C), 138.3 (C), 136.0 (CH), 132.6 (C), 130.4 (CH), 129.3 (CH), 129.0 (CH), 128.7 (CH), 128.1 (CH), 128.0 (CH), 127.9 (CH), 127.3 (CH), 125.7 (CH), 48.9 (CH_2_). IR (ATR, cm^–1^): 3280 (N–H), 1637 (N–C=O), 1521 (C=C). MS (EI) *m*/*z* (%): 235.1 (M^+^,100). HRMS (ESI) *m*/*z*: [M + H]^+^ calcd for [C_16_H_14_NO]^+^, 236.1075; found, 236.1074 for compound: **12a**.

### Procedure for the Synthesis of Isoindolo[2,1-*b*]isoquinolin-5(7*H*)-one (**13a**)

To a reaction tube provided with a magnetic bar with (*E*)-6,7-dihydro-5*H*-dibenzo[*c*,*g*]azonin-5-one **12a** (23.5 mg, 0.100 mmol), a stock solution of diphenylphosphoric acid (0.6 mg, 0.003 mmol) in dry dichloromethane (200 μL) was added at 25 °C, followed by the addition of NBS (18 mg, 0.101 mmol). The reaction mixture was subjected to TLC (CH_2_Cl_2_/MeOH 98:2). Once the starting material was consumed (5h), DBU (18 μL, 0.120 mmol) and NaI (18 mg, 0.120 mmol) were added to the reaction mixture at 25 °C. The new reaction mixture was allowed to stir and was subjected to TLC until the intermediate was consumed (16 h). Then, a standard aq. solution of NH_4_Cl (1 mL) was added; the layers were separated, and the aqueous phase was extracted with CH_2_Cl_2_ (3 × 1 mL). All organic layers were washed with water (3 × 1 mL) and brine (1 mL), dried with Na_2_SO_4_, filtered, and concentrated under vacuum. The crude was purified by silica gel flash chromatography (CH_2_Cl_2_ to CH_2_Cl_2_/MeOH 99:1) to obtain **13a** (21 mg, 0.09 mmol, 91%) as a pale-yellow solid. *R*_*f*_: 0.49 (petroleum ether/EtOAc 1:1). mp 186–188 °C. ^1^H NMR (300 MHz, CDCl_3_): δ 8.52–8.44 (m, 1H), 7.82–7.75 (m, 1H), 7.70–7.60 (m, 2H), 7.58–7.52 (m, 1H), 7.53–7.41 (m, 3H), 7.01 (s, 1H), 5.18 (s, 2H). ^13^C {^1^H} NMR (75 MHz, CDCl_3_): δ 161.2 (C), 142.2 (C), 138.0 (C), 137.7 (C), 134.1 (C), 132.2 (CH), 129.9 (CH), 128.4 (CH), 127.5 (CH), 126.4 (CH), 126.2 (CH), 124.8 (C), 123.5 (CH), 121.1 (CH), 98.1 (CH), 52.1 (CH_2_). IR (ATR, cm^–1^): 1660 (N–C=O), 1627 (C=C). MS (EI) *m*/*z* (%): 233.0 (M^+^, 100). HRMS (ESI) *m*/*z*: [M + H]^+^ calcd for [C_16_H_12_NO]^+^, 234.0919; found, 234.0922 for compound: **13a**.

## Supporting Information Available

Screening of reaction conditions, full experimental procedures, characterization data, and NMR spectra for new compounds prepared (PDF)
