# Supplementary material for: Transannular Approach to 2,3-Dihydropyrrolo[1,2-b]isoquinolin-5(1H)-ones through Brønsted Acid-Catalyzed Amidohalogenation
Source: J Org Chem. 2022 Jul 26;87(15):10062–72. doi: 10.1021/acs.joc.2c01045 (PMC9361296; doi:10.1021/acs.joc.2c01045)
Supplement: Supplementary file 1 — jo2c01045_si_001.pdf [file jo2c01045_si_001.pdf]

Supporting Information

The Transannular Approach to 2,3-Dihydropyrrolo[1,2-*b*]isoquinolin-5(1*H*)-ones through Brønsted Acid-Catalyzed Amidohalogenation.

Estefanía Capel, Javier Luis-Barrera, Ana Sorazu, Uxue Uria, Liher Prieto, Efraím Reyes, Luisa Carrillo and Jose L. Vicario**

Department of Organic and Inorganic Chemistry. University of the Basque Country (UPV/EHU). P.O. Box 644, 48080, Bilbao, Spain.

uxue.uria@ehu.es; joseluis.vicario@ehu.es

Index

1. Screening of halogentaing reagents	S3
2. General Procedures and Characterizations	S4
3. NMR spectra	S68

1. Screening of halogenating reagents

Table SI-1.

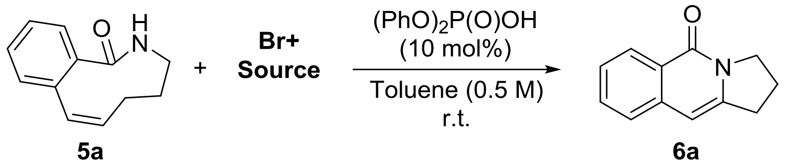

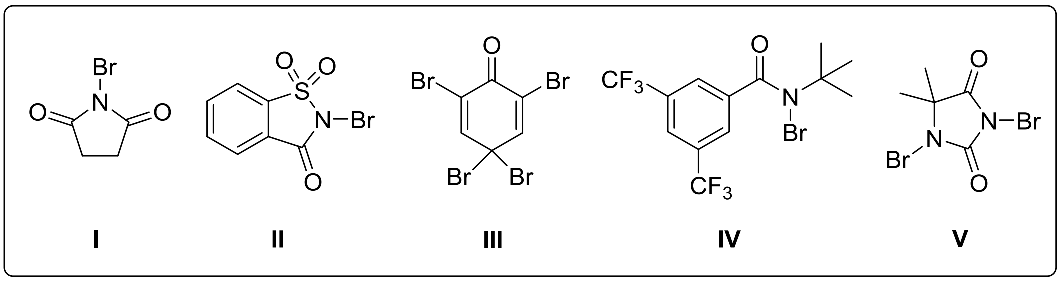

Entry	Br$^+$ Source	Time (min)	Yield (%)[a]
1	**I**	40	94
2	**II**	60	55
3	**III**	50	88
4	**IV**	15	52
5	**V**	10	76

[a] ^{1}H-NMR yield, using 1,3,5-trimethoxybenzene as internal standard.

2. General Procedures and Characterizations

General procedure for the synthesis of methyl 2-(5-chloropent-1-yn-1-yl)benzoate derivatives (1a-f). In an oven-dried, two-necked bottom flask equipped with a condenser and stir bar with the corresponding methyl 2-bromobenzoate (64 mmol), $PdCl_2(PPh_3)_2$ (0.898 g, 1.28 mmol), PCy_3 (0.718 g, 2.56 mmol) and CuI (0.49 g, 2.56 mmol) in freshly distilled Et_3N (256 mL) under Ar atmosphere, 5-chloropent-1-yne (10.6 mL, 95.5 mmol) was added. The mixture was heated in a heating plate to 80 ºC over 18 h. Then, the reaction was cooled to room temperature and was filtrated through a plug of Celite®. Aq. HCl 1 M (10 mL) was added to the filtrate and the organic layer was extracted with EtOAc (3 × 10 mL). The combined organic layers were washed with aq. std. $NaHCO_3$ (10 mL) dried over Na_2SO_4 and concentrated under vacuum. The crude was purified by silica gel flash chromatography.

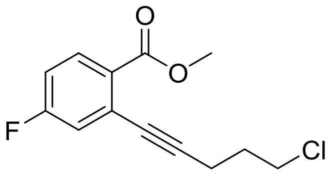

Methyl 2-(5-chloropent-1-yn-1-yl)-4-fluorobenzoate (1b). Following the general procedure A **1b** was obtained as a yellow oil (5.28 g, 20.75 mmol, 97%) by FC (PE/EtOAc 19:1) starting from methyl 2-bromo-4-fluorobenzoate (5 g, 21.46 mmol), 5-chloropent-1-yne (3.5 mL, 32.18 mmol), $PdCl_2(PPh_3)_2$ (0.301 g, 0.429 mmol), PCy_3 (0.240 g, 0.858 mmol), CuI (0.163 g, 0.858 mmol) and Et_3N (86 mL). $R_f = 0.6$ (PE/EtOAc 9:1). ^{1}H-NMR (300 MHz, $CDCl_3$) δ 7.95 (dd, $J = 8.8, 5.9$ Hz, 1H), 7.21 (dd, $J = 9.2, 2.6$ Hz, 1H), 7.04 (ddd, $J = 8.8, 7.9, 2.7$ Hz, 1H), 3.93 (s, 3H), 3.79 (t, $J = 6.5$ Hz, 2H), 2.70 (t, $J = 6.5$ Hz, 2H), 2.11 (p, $J = 6.5$ Hz, 2H). ^{13}C {^{1}H} NMR (75 MHz, $CDCl_3$) δ 165.7 (C), 164.2 (d, $J = 253.5$ Hz, C), 132.8 (d, $J = 9.7$ Hz, CH), 128.1 (d, $J = 3.2$ Hz, C), 126.8 (d, $J = 10.5$ Hz, C), 120.9 (d, $J = 23.1$ Hz, CH), 114.9 (d, $J = 21.6$ Hz, CH), 95.1 (C), 79.3

(d, J = 2.6 Hz, C), 52.1 (CH_3), 43.6 (CH_2), 31.2 (CH_2), 17.2 (CH_2). ^{19}F NMR (282 MHz, $CDCl_3$) δ -107.62. IR (ATR, cm^{-1}): 2232 (C≡C), 1730 (C=O), 1604 (C_{Arom}-C_{Arom}), 1575 (C_{Arom}-C_{Arom}). MS (EI) m/z (%): 219 (M^+-Cl, 5), 192 (100, M^+-CH_3CH_2Cl), 161 (14, M^+-CO_2Me-Cl). HRMS (ESI) m/z: $[M+H]^+$ Calcd for $[C_{13}H_{13}ClFO_2]^+$ 255.0583; Found 255.0592 for compound: **1b**.

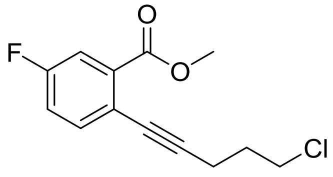

Methyl 2-(5-Chloropent-1-yn-1-yl)-5-fluorobenzoate (1c). Following the general procedure A **1c** was obtained as a yellow oil (0.98 g, 3.83 mmol, 94%) by FC (PE/EtOAc 8:2) starting from methyl 2-bromo-5-fluorobenzoate (0.60 mL, 4.05 mmol), 5-chloropent-1-yne (0.65 mL, 6.15 mmol), $PdCl_2(PPh_3)_2$ (0.06 g, 0,08 mmol), PCy_3 (0.05 g, 0.17 mmol), CuI (0.03 g, 0.17 mmol) and Et_3N (16 mL). R_f = 0.54 (PE/EtOAc 9:1). ^{1}H-NMR (300 MHz, $CDCl_3$) δ (ppm): 7.60 (dd, J = 9.2, 2.8 Hz, 1H), 7.49 (dd, J = 8.6, 5.5 Hz, 1H), 7.15 (ddd, J = 8.6, 7.8, 2.8 Hz, 1H), 3.92 (s, 3H), 3.77 (t, J = 6.4 Hz, 2H), 2.67 (t, J = 6.7 Hz, 2H), 2.08 (p, J = 6.6 Hz, 2H). ^{13}C {^{1}H} NMR (75 MHz, $CDCl_3$) δ (ppm): 165.7 (d, J = 2.5 Hz, C), 161.4 (d, J = 249.9 Hz, C), 136.2 (d, J = 7.8 Hz, CH) 133.9 (d, J = 7.5 Hz, C), 120.4 (d, J = 3.7 Hz, C), 119.2 (d, J = 21.9 Hz, CH), 117.4 (d, J = 24.0 Hz, CH), 93.4 (d, J = 1.6 Hz, C), 79.3 (C), 52.6 (CH_3), 43.8 (CH_2), 31.5 (CH_2), 17.3 (CH_2). ^{19}F-NMR (282 MHz, $CDCl_3$) δ (ppm): -111.4. IR (ATR, cm^{-1}): 2257 (C≡C), 1734 (C=O). MS (IE) m/z (%): 192 (100, M^+-CH_3CH_2Cl). HRMS (ESI) m/z: $[M+H]^+$ Calcd for $[C_{13}H_{13}ClFO_2]^+$ 255.0583; Found 255.0591 for compound: **1c**.

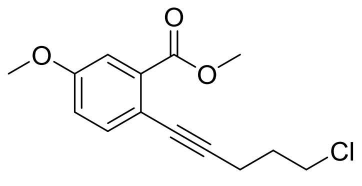

Methyl 2-(5-chloropent-1-yn-1-yl)-5-methoxybenzoate (1d). Following the general procedure A, **1d** was obtained as an orange oil (4.929 g, 18.48 mmol, 91%) by FC (PE/EtOAc 1:0 to 95:05) starting from methyl 2-bromo-5-methoxybenzoate (5.00 g, 20.40 mmol), 5-chloro-1-pentyn (3.24 mL, 3.138 g, 30.60 mmol), $PdCl_2(PPh_3)_2$ (0.286 g, 0.408 mmol, 2 mol%), PCy_3 (0.229 g, 0.816 mmol), CuI (0.155 g, 0.816 mmol) and Et_3N (82 mL). R_f = 0.26 (PE/EtOAc 95:05). ^{1}H-NMR (300 MHz, $CDCl_3$): 7.41 (d, J = 9.0 Hz, 1H), 7.39 (d, J = 3.2 Hz, 1H), 6.96 (dd, J = 8.6, 2.8 Hz, 1H), 3.91 (s, 3H), 3.82 (s, 3H), 3.76 (t, J = 6.4 Hz, 2H), 2.64 (t, J = 6.7 Hz, 2H), 2.06 (p, J = 6.6 Hz, 2H). ^{13}C {^{1}H} NMR (75 MHz, $CDCl_3$) δ 166.8 (C), 158.8 (C), 135.6 (CH), 133.3 (C), 118.3 (CH), 116.3 (C), 114.9 (CH), 91.6 (C), 80.0 (C), 55.6 (CH_3), 52.3 (CH_3), 43.9 (CH_2), 31.6 (CH_2), 17.3 (CH_2). IR (ATR, cm^{-1}): 2952 (C-H st), 2193 (C≡C st), 2225 (C≡C st), 1727 (C=O st), 1288 (CO-O st as), 1223 (O-C-C st as). MS (EI) m/z (%): 268.1 (32), 267.0 (37), 266.1 (M^+, 100), 229.0 (9). HRMS (ESI) m/z: $[M+H]^+$ Calcd for $[C_{14}H_{16}ClO_3]^+$ 267.0782; Found 267.0795 for compound: **1d**.

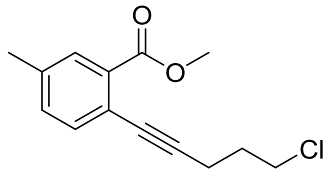

Methyl 2-(5-chloropent-1-yn-1-yl)-5-methylbenzoate (1e). Following the general procedure A **1e** was obtained as a pale yellow oil (1.09 g, 4.36 mmol, 94%) by FC (PE/EtOAc 95:5) starting from methyl 2-bromo-5-methylbenzoate (1.08 g, 4.65 mmol), 5-chloro-1-pentyn (0.8 mL, 6.96 mmol), $PdCl_2(PPh_3)_2$ (63 mg, 0.09 mmol), PCy_3 (52 mg, 0.18 mmol), CuI (17 mg, 0.18 mmol) and Et_3N (18 mL). R_f = 0.56 (PE/EtOAc 9:1). ^{1}H-NMR (300 MHz, $CDCl_3$) δ7.72 (dd, J = 1.2, 0.5 Hz, 1H), 7.41 (d, J = 7.9 Hz, 1H), 7.25

(dd, J = 7.9, 1.2 Hz, 1H), 3.93 (s, 3H), 3.79 (t, J = 6.5 Hz, 2H), 2.68 (t, J = 6.7 Hz, 2H), 2.38 (s, 3H), 2.10 (p, J = 6.6 Hz, 2H). ^{13}C {^{1}H} NMR (75 MHz, $CDCl_3$) δ 166.9 (C), 137.6 (C), 134.0 (CH), 132.4 (CH), 131.7 (C), 130.7 (CH), 121.0 (C), 92.5 (C), 80.2 (C), 52.1 (CH_3), 43.7 (CH_2), 31.4 (CH_2), 21.1 (CH_3), 17.2 (CH_2). IR (ATR, cm^{-1}): 2221 (C≡C), 1728 (C=O), 1608 (C_{Arom}-C_{Arom}), 1559 (C_{Arom}-C_{Arom}). MS (EI) m/z (%): 188.1 (M^+-C_3H_2Cl, 100), 115 (M^+-CO_2Me-CH_3CH_2Cl, 24). HRMS (ESI) m/z: $[M+H]^+$ Calcd for $[C_{14}H_{16}ClO_2]^+$ 251.0833; Found 251.0845 for compound: **1e**.

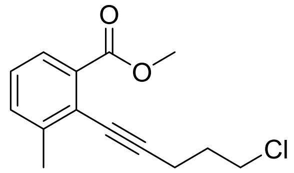

Methyl 2-(5-chloropent-1-yn-1-yl)-3-methylbenzoate (1f). Following the general procedure A **1f** was obtained as a yellow oil (4.14 g, 16.2 mmol, 76%) by FC (PE/EtOAc 95:5) starting from methyl 2-bromo-3-methylbenzoate (5 g, 21.83 mmol), 5-chloropent-1-yne (3.6 mL, 32.74 mmol), $PdCl_2(PPh_3)_2$ (0.306 g, 0.437 mmol), PCy_3 (0.245 g, 0.873 mmol), CuI (0.166 g, 0.873 mmol) and Et_3N (87 mL). R_f = 0.6 (PE/EtOAc 9:1). ^{1}H-NMR (300 MHz, $CDCl_3$) δ 7.70 (dd, J = 7.8, 0.6 Hz, 1H), 7.37 (dd, J = 7.8, 0.6 Hz, 1H), 7.23 (t, J = 7.8 Hz, 1H), 3.93 (s, 3H), 3.80 (t, J = 6.4 Hz, 2H), 2.75 (t, J = 6.7 Hz, 2H), 2.47 (s, 3H), 2.12 (p, J = 6.6 Hz, 2H). ^{13}C {^{1}H} NMR (75 MHz, $CDCl_3$) δ 167.4 (C), 141.8 (C), 132.8 (C), 132.6 (CH), 127.3 (CH), 126.9 (CH), 123.2 (C), 98.4 (C), 78.4 (C), 52.1 (CH_3), 43.7 (CH_2), 31.4 (CH_2), 21.3 (CH_3), 17.3 (CH_2). IR (ATR, cm^{-1}): 2227 (C≡C), 1727 (C=O), 1592 (C_{Arom}-C_{Arom}), 1574 (C_{Arom}-C_{Arom}). MS (EI) m/z (%): 250.1 (M^+, 0.9), 215.1 (M^+-Cl, 5), 188.1 (M^+-CH_3CH_2Cl, 100). HRMS (ESI) m/z: $[M+H]^+$ Calcd for $[C_{14}H_{16}ClO_2]^+$ 251.0833; Found 251.0845 for compound: **1f**.

General procedure B for the synthesis of methyl (*Z*)-2-(2-(5-chloropent-1-en-1-yl)benzoate derivatives (2a-f). To a two-necked bottom flask equipped with a stirring bar and H_2 balloon with the corresponding methyl 2-(5-chloropent-1-yn-1-yl)benzoate **1a-f** (12.7 mmol) in EtOAc (127 mL) and quinoline (0.6 mL, 5 mmol), Pd on $CaCO_3$ (1.06 g, 0.5 mmol, 8 mol%) was added. The air flask was evacuated under vacuum and backfilled with hydrogen three times and the reaction mixture was allowed to stir at room temperature under hydrogen atmosphere (balloon pressure) until full consumption of starting material, 1 h as judged by thin layer chromatography. The reaction mixture was filter through a plug of Celite® and the filtrate was concentrated under vacuum. The crude was purified by silica gel flash chromatography. NOTE: The synthesis of **2b** required a modification of this procedure (see below).

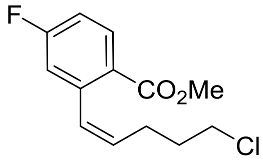

Methyl (*Z*)-2-(5-chloropent-1-en-1-yl)-4-fluorobenzoate (2b). Hydrogenator flask containing 2-(5-chloropent-1-yn-1-yl)-4-fluorobenzoate **1b** (4.43 g, 17.25 mmol), quinoline (0.17 mL, 1.38 mmol) and Pd on $CaCO_3$ (3.6 g, 1.7 mmol) in EtOAc (173 mL) was evacuated and backfilled with hydrogen three times and the reaction mixture was allowed to shake in the hydrogenator at 2.5 atm pressure of hydrogen until full consumption of starting material, 2 h as judged by TLC. The reaction mixture was filter through a plug of Celite® and the filtrate was concentrated under vacuum. The crude was purified by silica gel flash chromatography (PE/EtOAc 19:1) to obtain methyl (*Z*)-2-(2-(5-chloropent-1-en-1-yl)benzoate as a yellow oil (4.16 g, 16.22 mmol, 94%). ^{1}H-NMR (300 MHz, $CDCl_3$) δ 8.01 (dd, *J* = 8.7, 5.9 Hz, 1H), 7.07 – 6.96 (m, 2H), 6.92 (d, *J* = 11.7 Hz, 1H), 5.72 (dt, *J* = 11.7, 7.4 Hz, 1H), 3.88 (s, 3H), 3.52 (t, *J* = 6.8 Hz, 2H), 2.31 (app qd, *J* = 7.3, 1.7 Hz, 2H), 1.88 (m, 2H). ^{13}C {^{1}H} NMR (75 MHz, $CDCl_3$) δ 166.5 (C),

164.3 (d, *J* = 253.7 Hz, C), 141.9 (d, *J* = 8.8 Hz, C), 133.1 (d, *J* = 9.4 Hz, CH), 130.4 (CH), 129.6 (d, *J* = 1.5 Hz, CH), 125.3 (d, *J* = 3.3 Hz, C), 117.5 (d, *J* = 21.8 Hz, CH), 113.8 (d, *J* = 21.5 Hz, CH), 51.9 (CH_3), 44.2 (CH_2), 32.3 (CH_2), 25.4 (CH_2). ^{19}F NMR (282 MHz, $CDCl_3$) δ -107.16. IR (ATR, cm^{-1}): 1721 (C=O), 1605 (C_{Arom}-C_{Arom}), 1577 (C_{Arom}-C_{Arom}). MS (EI) m/z (%): 188 (M^+-MeOH-Cl, 61), 160 (M^+-MeOH-CH_3CH_2Cl, 100). HRMS (ESI) m/z: $[M+H]^+$ Calcd for $[C_{13}H_{15}ClFO_2]^+$ 257.0739; Found 257.0751 for compound: **2b**.

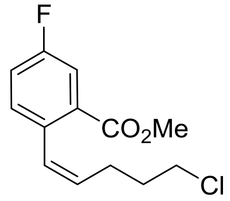

Methyl (*Z*)-2-(5-chloropent-1-en-1-yl)-5-fluorobenzoate (2c). Following the general procedure B **2c** was obtained as a yellow oil (0.76 g, 2.97 mmol, 81%) by FC (PE/EtOAc 19:1) starting from methyl 2-(5-chloropent-1-yn-1-yl)-5-fluorobenzoate **1c** (0.94 g, 3.68 mmol), quinoline (0.036 mL, 0.29 mmol) and Pd on $CaCO_3$ (0.39 g, 0.18 mmol). R_f = 0.60 (PE/EtOAc 9:1). ^{1}H-NMR (300 MHz, $CDCl_3$; * indicates *trans* diastereoisomer resonances) δ (ppm): 7.73 (dd, *J* = 9.4, 2.6 Hz, 1H), 7.64* (dd, *J* = 9.4, 2.8 Hz, 1H), 7.57* (dd, *J* = 8.7, 5.5 Hz, 1H), 7.42 – 7.22 (m, 2H), 7.20* (m, 1H), 6.93 (d, *J* = 11.5 Hz, 1H), 6.09* (dt, *J* = 15.7, 6.9 Hz, 1H), 5.75 (dt, *J* = 11.5, 7.4 Hz, 1H), 3.99* (s, 3H), 3.96 (s, 3H), 3.69* (t, *J* = 6.6 Hz, 2H), 3.57 (t, *J* = 6.6 Hz, 2H), 2.49* (q, *J* = 6.7 Hz, 2H), 2.33 (qd, *J* = 7.1, 1.7 Hz, 2H), 2.05* (p, *J* = 6.8 Hz, 2H), 1.93 (p, *J* = 6.6 Hz, 2H). ^{13}C {^{1}H} NMR (75 MHz, $CDCl_3$; * indicates *trans* diastereoisomer resonances,) δ (ppm): 166.8* (d, *J* = 2.6 Hz, C), 166.4 (d, *J* = 2.8 Hz, C), 161.4* (d, *J* = 247.1 Hz, C), 161.3 (d, *J* = 247.0 Hz, C), 135.8* (d, *J* = 3.5 Hz, C), 135.0 (d, *J* = 3.5 Hz, C), 132.5 (d, *J* = 7.4 Hz, CH), 130.9 (d, *J* = 7.2 Hz, C), 130.2 (CH), 129.6 (CH), 129.3* (d, *J* = 7.5 Hz, CH), 129.2* (CH), 119.4* (d, *J* = 21.3 Hz, CH), 118.9 (d, *J* = 21.1 Hz, CH), 117.5* (d, *J* = 23.0 Hz,

CH), 117.4 (d, J = 23.3 Hz, CH), 116.9* (CH), 52.4* (CH_3), 52.3 (CH_3), 45.1* (CH_2), 44.4 (CH_2), 33.7* (CH_2), 32.5 (CH_2), 26.9* (CH_2), 25.5 (CH_2). ^{19}F- NMR (282 MHz, $CDCl_3$; * indicates *trans* diastereoisomer resonances,) δ (ppm): -114.7, -116.7*. IR (ATR, cm^{-1}): 1728 (st C=O), 1607 (CH=CH), 1205 (st C=O). EM (EI) m/z (%): 256 (M^+, 15), 179 (M^+-C_3H_6Cl, 100). HRMS (ESI) m/z: $[M+H]^+$ Calcd for $[C_{13}H_{15}ClFO_2]^+$ 257.0739; Found 257.0751 for compound: **2c**.

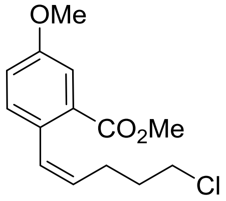

Methyl (*Z*)-2-(5-chloropent-1-en-1-yl)-5-methoxybenzoate (2d). Following the general procedure B, **2d** was obtained as a yellow oil (2.90 g, 10.85 mmol, 55%) by FC (PE/EtOAc 9:1) starting from methyl 2-(5-chloropent-1-yn-1-yl)-5-methoxybenzoate **1d** (5.24 g, 19.66 mmol), quinoline (0.187 mL, 1.573 mmol, 8 mol%) and Pd on $CaCO_3$ (2.09 g, 0.983 mmol, 5 mol%) in EtOAc (197 mL). R_f = 0.50 (PE/EtOAc 9:1) ^{1}H-NMR (300 MHz, $CDCl_3$): δ 7.48 (d, J = 2.8 Hz, 1H), 7.21 (d, J = 8.5 Hz, 1H), 7.04 (dd, J = 8.5, 2.8 Hz, 1H), 6.86 (dt, J = 11.4, 1.7 Hz, 1H), 5.63 (dt, J = 11.5, 7.4 Hz, 1H), 3.88 (s, 3H), 3.86 (s, 3H), 3.51 (t, J = 6.7 Hz, 2H), 2.28 (qd, J = 7.4, 1.7 Hz, 2H), 1.92 – 1.81 (m, 2H). ^{13}C {^{1}H} NMR (75 MHz, $CDCl_3$): δ 167.5 (C), 158.3 (C), 131.9 (CH), 131.3 (C), 130.3 (C), 130.0 (CH), 129.2 (CH), 118.0 (CH), 115.1 (CH), 55.6 (CH_3), 52.1 (CH_3), 44.5 (CH_2), 32.7 (CH_2), 25.7 (CH_2). IR (ATR, cm^{-1}): 1721 (C=O st), 1285 (CO-O st as), 1222 (O-C-C st as). MS (EI) m/z (%): 270.1 (27), 269.1 (27), 268.1 (M^+, 100), 267.0 (16), 252.0 (M^+ – O, 16), 233.0 (M^+ – Cl, 11), 232.0 (20). HRMS (ESI) m/z: $[M+H]^+$ Calcd for $[C_{14}H_{18}ClO_3]^+$ 269.0939; Found 269.0951 for compound: **2d**.

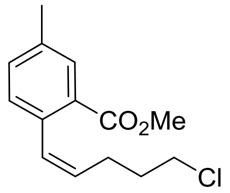

Methyl (*Z*)-2-(5-chloropent-1-en-1-yl)-5-methylbenzoate (2e). Following the general procedure B **2e** was obtained as a yellow oil (1.02 g; 4.02 mmol, 92%) by FC (PE/EtOAc 19:1) starting from methyl 2-(5-chloropent-1-yn-1-yl)-5-methylbenzoate **1e** (1.1 g, 4.374 mmol), quinoline (0.05 mL, 0.35 mmol; 8 mol%) and Pd on $CaCO_3$ (0.927 g, 0.44 mmol; 10 mol%). R_f = 0.62 (PE/EtOAc 9:1). ^{1}H-NMR (300 MHz, $CDCl_3$) δ 7.68 (s, 1H), 7.21 (d, *J* = 7.8 Hz, 1H), 7.09 (d, *J* = 7.8 Hz, 1H), 6.81 (d, *J* = 11.5 Hz, 1H), 5.57 (dt, *J* = 11.5, 7.4 Hz, 1H), 3.79 (s, 3H), 3.42 (t, *J* = 6.8 Hz, 2H), 2.31 (s, 3H), 2.19 (app qd, *J* = 7.1, 1.6 Hz, 2H), 1.84 – 1.72 (m, 2H). ^{13}C {^{1}H} NMR (75 MHz, $CDCl_3$) δ 167.7 (C), 136.6 (C), 135.8 (C), 132.4 (CH), 130.9 (CH), 130.5 (CH), 130.2 (CH), 129.4 (CH), 129.0 (C), 51.8 (CH_3), 44.4 (CH_2), 32.6 (CH_2), 25.5 (CH_3), 20.9 (CH_2). IR (ATR, cm^{-1}): 1727 (C=O), 1610 (C_{Arom}-C_{Arom}), 1560 (C_{Arom}-C_{Arom}). MS (EI) m/z (%): 251.1 (M^+, 64), 171.1 (M^+-MeOH-CH_3Cl, 47), 128.0 (M^+-CO_2Me-CH_3CH_2Cl, 100). HRMS (ESI) m/z: $[M+H]^+$ Calcd for $[C_{14}H_{18}ClO_2]^+$ 253.0990; Found 253.1001 for compound: **2e**.

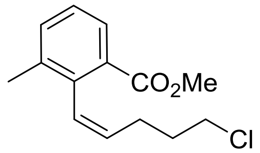

Methyl (*Z*)-2-(5-chloropent-1-en-1-yl)-3-methylbenzoate (2f). Following the general procedure B **2f** was obtained as a yellow oil (1.90 g; 7.5 mmol, 98%) by FC (PE/EtOAc 19:1) starting from methyl 2-(5-chloropent-1-yn-1-yl)-3-methylbenzoate **1f** (1.929 g, 7.69 mmol), quinoline (0.07 mL, 0.6 mmol, 8 mol%) and Pd on $CaCO_3$ (1.59 g, 0.75 mmol, 10 mol%). R_f = 0.61 (PE/EtOAc 9:1). ^{1}H-NMR (300 MHz, $CDCl_3$) δ 7.57 (d, *J* = 7.6 Hz, 1H), 7.26 (d, *J* = 7.6 Hz, 1H), 7.13 (t, *J* = 7.6 Hz, 1H), 6.54 (d, *J* = 11.3 Hz, 1H), 5.59 (dt, *J* = 11.3, 7.3 Hz, 1H), 3.75 (s, 3H), 3.32 (t, *J* = 6.7 Hz, 2H), 2.17 (s, 3H), 1.83

(app. qd, *J* = 7.5, 1.5 Hz, 2H), 1.66 (m, 2H). ^{13}C {^{1}H} NMR (75 MHz, CDCl3) δ 168.5 (C), 137.3 (C), 137.1 (C), 133.1 (CH), 130.9 (C), 130.0 (CH), 128.8 (CH), 127.1 (CH), 126.6 (CH), 51.9 (CH_3), 44.2 (CH_2), 31.9 (CH_2), 25.6 (CH_2), 20.2 (CH_3). IR (ATR, cm^{-1}): 2232 (C≡C), 1730 (C=O), 1605 (C_{Arom}-C_{Arom}), 1568 (C_{Arom}-C_{Arom}). MS (EI) m/z (%): 252.1 (M+, 13), 175.1 (M+-CH_3CH_2Cl, 100), 115.1 (M^+-CO_2Me-$CH_3CH_2CH_2Cl$, 41).. HRMS (ESI) m/z: $[M+H]^+$ Calcd for $[C_{14}H_{18}ClO_2]^+$ 253.0990; Found 253.1001 for compound: **2f**.

General procedure C for the synthesis of methyl (*Z*)-2-(5-(1,3-dioxoisoindolin-2-yl)pent-1-en-1-yl)benzoate derivatives (3a-f). An oven-dried two necked bottom flask provided with a condenser and a magnetic bar with a suspension of the corresponding methyl (*Z*)-2-(2-(5-chloropent-1-en-1-yl)benzoate **2a-f** (12.2 mmol), Cs_2CO_3 (8.73 g, 26.8 mmol), phthalimide (2.69 g, 18.3 mmol) and KI (20.5 mg, 0.122 mmol) in DMF (70 mL) under Ar atmosphere was heated in a heating plate at 100 ºC for 2 h. Then, the reaction mixture was let cool down to room temperature and water (100 mL) and EtOAc (50 mL) was added. The aqueous layer was extracted with EtOAc (3 × 25 mL), washed with H_2O (2 × 75 mL), dried with Na_2SO_4, filtrated and concentrated under vacuum. The crude was purified with silica gel flash chromatography.

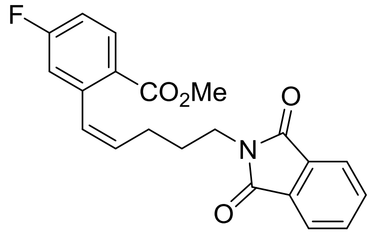

Methyl (*Z*)-2-(5-(1,3-dioxoisoindolin-2-yl)pent-1-en-1-yl)-4-fluorobenzoate (3b). Following the general procedure H **3b** as obtained as a white solid (5.17 g, 14.1 mmol, 82%) by FC (PE EtOAc 9:1 to PE EtOAc 8:2) starting from methyl (*Z*)-2-(5-chloropent-1-en-1-yl)-4-fluorobenzoate **2b** (4.43 g, 17.25 mmol), Cs_2CO_3 (12.4 g, 37.9 mmol), phthalimide (3.05 g, 20.7 mmol) and KI (28.7 mg, 0.173 mmol) in DMF (86 mL) R_f = 0.34 (PE/EtOAc 8:2). mp: 74-76 °C. ^{1}H-NMR (300 MHz, $CDCl_3$) δ 7.97 (dd, *J* = 8.6, 6.0 Hz, 1H), 7.83 (dd, *J* = 5.5, 3.0 Hz, 2H), 7.71 (dd, *J* = 5.5, 3.0 Hz, 2H), 7.04 – 6.91 (m, 2H), 6.88 (d, *J* = 11.6 Hz, 1H), 5.78 (dt, *J* = 11.6, 7.4 Hz, 1H), 3.87 (s, 3H), 3.66 (t, *J* = 7.4 Hz, 2H), 2.20 (m, 2H), 1.80 (p, *J* = 7.4 Hz, 2H). ^{13}C {^{1}H} NMR (75 MHz, $CDCl_3$) δ 168.2 (2xC), 166.6 (C), 164.2 (d, *J* = 253.4 Hz, C), 141.8 (d, *J* = 8.8 Hz, C), 133.8 (2 × CH), 133.1 (d, *J* = 9.4 Hz, CH), 132.0 (2 × C), 131.0 (CH), 129.0 (d, J = 1.3 Hz, CH), 125.3 (d, *J* = 3.0 Hz, C), 123.1 (2 × CH), 117.4 (d, *J* = 21.8 Hz, CH), 113.8 (d, *J* = 21.5

Hz, CH), 51.9 (CH_3), 37.5 (CH_2), 28.4 (CH_2), 25.6 (CH_2). ^{19}F NMR (282 MHz, $CDCl_3$) δ -107.14. IR (ATR, cm^{-1}): 1770 (O=C-N-C=O), 1707 (C=O), 1607 (C_{Arom}-C_{Arom}), 1577 (C_{Arom}-C_{Arom}). MS (EI) m/z (%): 317 (M^+-MeOH, 45), 170 (M^+-CH_3OH-Phth, 100). HRMS (ESI) m/z: $[M+H]^+$ Calcd for $[C_{21}H_{20}FNO_4]^+$ 368.1293; Found 368.1295 for compound: **3b.**

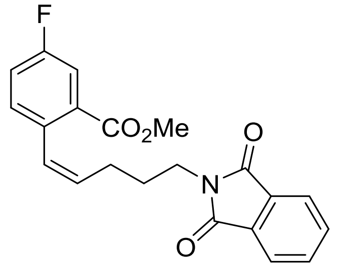

Methyl (*Z*)-2-(5-(1,3-dioxoisoindolin-2-yl)pent-1-en-1-yl)-5-fluorobenzoate (3c). Following the general procedure H **3c** as obtained as a white solid (0.77 g, 2.09 mmol, 79%) by FC (PE EtOAc 9:1 to PE EtOAc 8:2) starting from methyl (*Z*)-2-(5-chloropent-1-en-1-yl)-4-fluorobenzoate **2c** (0.68 g, 2.65 mmol), Cs_2CO_3 (1.89 g, 5.82 mmol), phthalimide (0.58 g, 3.97 mmol) and KI (4.4 mg, 0.027 mmol) in DMF (15 mL) R_f = 0.30 (PE/EtOAc 8:2). mp: 73-75 °C. ^{1}H-NMR (300 MHz, $CDCl_3$; * indicates *trans* diastereoisomer) δ (ppm): 7.74 (dd, J = 5.5, 3.0 Hz, 2H), 7.63 (dd, J = 5.5, 3.0 Hz, 2H), 7.52 (dd, J = 9.4, 2.8 Hz, 1H), 7.45* (dd, J = 9.4, 2.8 Hz, 1H), 7.39* (dd, J = 8.8, 5.5 Hz, 1H), 7.14 (dd, J = 8.5, 5.6 Hz, 1H), 7.07 (td, J = 8.1, 2.8 Hz, 1H), 6.76 (d, J = 11.5 Hz, 1H), 6.01* (dt, J = 15.7, 6.8 Hz, 1H), 5.67 (dt, J = 11.5, 7.4 Hz, 1H), 3.83* (s, 3H), 3.81 (s, 3H), 3.70* (t, J = 7.2 Hz, 2H), 3.57 (t, J = 7.3 Hz, 2H), 2.35 – 2.20* (m, 2H), 2.08 (qd, J = 7.5, 1.7 Hz, 2H), 1.84* (p, J = 7.2 Hz, 2H), 1.73 (p, J = 7.6 Hz, 2H). ^{13}C {^{1}H} NMR (75 MHz, $CDCl_3$; * indicates *trans* diastereoisomer) δ (ppm): 168.3* (2xC), 168.2 (2xC), 166.6* (d, J = 2.6 Hz, C), 166.2 (d, J = 2.7 Hz, C), 161.1* (d, J = 245.3 Hz, C), 161.0 (d, J = 246.9 Hz, C), 135.6* (d, J = 3.4 Hz, C), 134.7 (d, J= 3.5 Hz, C), 133.8 (2xCH), 132.2 (d, J = 7.4 Hz, CH), 132.0 (2xC), 130.8 (d, J = 7.2 Hz, C), 130.5 (CH), 129.3* (d, J = 7.1

Hz, C), 129.0* (d, J = 7.5 Hz, CH), 128.8 (CH), 128.4* (CH), 123.1 (2xCH), 119.1* (d, J = 21.3 Hz, CH), 118.6 (d, J = 21.0 Hz, CH), 117.2 (d, J = 23.3 Hz, CH), 116.8* (d, J = 23.3 Hz, CH), 52.2* (CH_3), 52.1 (CH_3), 37.5 (CH_2), 30.4* (CH_2), 28.5 (CH_2), 27.9* (CH_2), 25.6 (CH_2). ^{19}F-NMR (282 MHz, $CDCl_3$; * indicates *trans* diastereoisomer) δ (ppm): -114.8, -115.2*. IR (ATR, cm^{-1}): 1770 (O-C=O), 1706 (O=C-N-C=O). MS (EI) m/z (%): 367 (M^+, 1), 335 (M^+-MeOH, 31), 188 (M^+-MeOH-Phth, 100), 133 (M^+-CO_2Me-CH_3CH_2Phth, 57). HRMS (ESI) m/z: $[M+H]^+$ Calcd for $[C_{21}H_{20}FNO_4]^+$ 368.1293; Found 368.1300 for compound: **3c.**

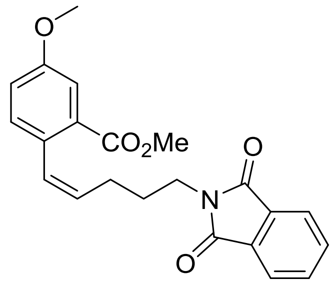

Methyl (*Z*)-2-(5-(1,3-dioxoisoindolin-2-yl)pent-1-en-1-yl)-5-methoxybenzoate (3d). Following the general procedure C, **3d** was obtained as a thick white oil (3.57 g, 10.24 mmol, 94%) by FC (PE/EtOAc 9:1 to PE/EtOAc 7:3) starting from methyl (*Z*)-2-(5-chloropent-1-en-1-yl)-5-methoxybenzoate **2d** (2.91 g, 10.85 mmol), Cs_2CO_3 (7.77 g, 23.87 mmol), phthalimide (2.394 g, 16.275 mmol) and KI (18.1 mg, 0.109 mmol) in DMF (55 mL). R_f = 0.47 (PE/EtOAc 7:3). ^{1}H-NMR (300 MHz, $CDCl_3$): δ 7.84 – 7.77 (m, 2H), 7.72 – 7.65 (m, 2H), 7.42 (d, J = 2.8 Hz, 1H), 7.14 (d, J = 8.5 Hz, 1H), 6.95 (dd, J = 8.5, 2.8 Hz, 1H), 6.80 (dt, J = 11.6, 1.7 Hz, 1H), 5.66 (dt, J = 11.5, 7.4 Hz, 1H), 3.85 (s, 3H), 3.81 (s, 3H), 3.63 (t, J = 7.4 Hz, 2H), 2.16 (dq, J = 7.6, 1.7 Hz, 2H), 1.82 – 1.70 (m, 2H). ^{13}C {^{1}H} NMR (75 MHz, $CDCl_3$): δ 168.4 (2 × C), 167.6 (C), 158.2 (C), 134.0 (2 × CH), 132.2 (2 × C), 131.9 (CH), 131.2 (C), 130.4 (C), 129.7 (CH), 129.5 (CH), 123.3 (2 × CH), 118.0 (CH), 115.1 (CH), 55.6 (CH_3), 52.1 (CH_3), 37.8 (CH_2), 28.7 (CH_2), 25.8 (CH_2). IR (ATR, cm^{-1}): 1708 (C=O st), 1285 (CO-O st as), 1222 (O-C-C st as). MS (EI) m/z (%):

381.1 (11), 380.1 (45), 379.1 (M^+, 100), 378.1 (55), 377.1 (27), 348.0 (14), 347.1 (18).. HRMS (ESI) m/z: $[M+H]^+$ Calcd for $[C_{22}H_{22}NO_5]^+$ 380.1492; Found 380.1503 for compound: **3d.**

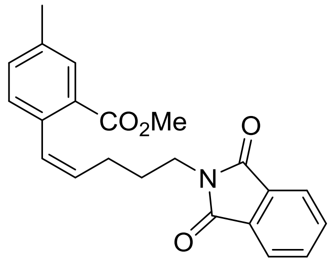

Methyl (*Z*)-2-(5-(1,3-dioxoisoindolin-2-yl)pent-1-en-1-yl)-5-methylbenzoate (3e). Following the general procedure C **3e** as obtained as a white solid (1.576 g, 4.34 mmol, 92%) by FC (PE EtOAc 9:1 to PE EtOAc 8:2) starting from methyl (*Z*)-2-(5-chloropent-1-en-1-yl)-4-fluorobenzoate **2e** (1.95 g, 4.712 mmol), Cs_2CO_3 (3.38 g, 10.37 mmol), phthalimide (1.04 g, 7.07 mmol) and KI (7.8 mg 0.0471 mmol) in DMF (24 mL) R_f = 0.75 (PE/EtOAc 7:3). mp: 92-94 °C. ^{1}H-NMR (300 MHz, $CDCl_3$) δ 7.84 (dd, J = 5.5, 3.0 Hz, 2H), 7.71 (dd, J = 5.4, 3.0 Hz, 3H), 7.23 (dd, J = 7.9, 1.6 Hz, 1H), 7.14 (d, J = 7.9 Hz, 1H), 6.87 (d, J = 11.5 Hz, 1H), 5.71 (dt, J = 11.5, 7.4 Hz, 1H), 3.87 (s, 3H), 3.67 (t, J = 7.4 Hz, 2H), 2.36 (s, 3H), 2.19 (app qd, J = 7.5, 1.4 Hz, 2H), 1.79 (p, J = 7.5 Hz, 2H). ^{13}C {^{1}H} NMR (75 MHz, $CDCl_3$) δ 168.3 (2 × C), 167.8 (C), 136.5 (C), 135.7 (C), 133.8 (2 × CH), 132.3 (CH), 132.1 (2 × C), 130.9 (CH), 130.5 (CH), 129.9 (CH), 129.7 (CH), 129.1 (C), 123.1 (2 × CH), 51.8 (CH_3), 37.6 (CH_2), 28.6 (CH_2), 25.7 (CH_2), 20.9 (CH_3). IR (ATR, cm^{-1}): 1771 (O=C-N-C=O), 1707 (C=O), 1609 (C_{Arom}-C_{Arom}), 1576 (C_{Arom}-C_{Arom}). MS (EI) m/z (%): 331.1 (M^+-MeOH, 40), 184.1 (M^+-MeOH-Phth, 61). HRMS (ESI) m/z: $[M+H]^+$ Calcd for $[C_{22}H_{22}NO_4]^+$ 364.1543; Found 364.1547 for compound: **3e.**

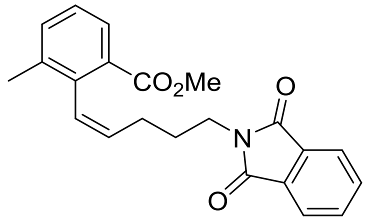

Methyl (*Z*)-2-(5-(1,3-dioxoisoindolin-2-yl)pent-1-en-1-yl)-3-methylbenzoate (3f). Following the general procedure C **3f** as obtained as a white solid (2.13 g, 5.86 mmol, 77%) by FC (PE EtOAc 9:1 to PE EtOAc 8:2) starting from methyl (*Z*)-2-(5-chloropent-1-en-1-yl)-4-fluorobenzoate **2f** (1.9 g, 7.5 mmol), Cs_2CO_3 (5.5 g, 16.72 mmol), phthalimide (1.7 g, 11.4 mmol) and KI (12.4 mg, 0.075 mmol) in DMF (38 mL) R_f = 0.38 (PE/EtOAc 8:2). mp: 89-90 °C. ^{1}H-NMR (300 MHz, $CDCl_3$) δ 7.83 (dd, *J* = 5.5, 3.0 Hz, 2H), 7.71 (dd, *J* = 5.5, 3.0 Hz, 2H), 7.58 (d, *J* = 7.4 Hz, 1H), 7.32 (d, *J* = 7.4 Hz, 1H), 7.18 (t, *J* = 7.4 Hz, 1H), 6.61 (d, *J* = 11.3 Hz, 1H), 5.76 (dt, *J* = 11.3, 7.5 Hz, 1H), 3.84 (s, 3H), 3.56 (t, *J* = 7.5, 2H), 2.25 (s, 3H), 1.84 (q, *J* = 7.5, 2H), 1.68 (p, *J* = 7.5 Hz, 2H). ^{13}C {^{1}H} NMR (75 MHz, $CDCl_3$) δ 168.7 (C), 168.2 (2 × C), 137.2 (C), 137.1 (C), 133.8 (2 × CH), 133.1 (CH), 132.1 (2 × C), 131.1 (C), 130.5 (CH), 128.3 (CH), 127.0 (CH), 126.6 (CH), 123.1 (2 × CH), 51.9 (CH_3), 37.6 (CH_2), 27.9 (CH_2), 25.7 (CH_2), 20.2 (CH_3). IR (ATR, cm^{-1}): 1771 (O=C-N-C=O), 1709 (C=O), 1605 (C_{Arom}-C_{Arom}), 1593 (C_{Arom}-C_{Arom}). MS (EI) m/z (%): 331.1 (M^+-MeOH, 29), 188 (M^+-Phth-MeOH, 2). HRMS (ESI) m/z: $[M+H]^+$ Calcd for $[C_{22}H_{22}NO_4]^+$ 364.1543; Found 364.1550 for compound: **3f.**

General procedure D for the synthesis of methyl (*Z*)-2-(5-aminopent-1-en-1-yl)benzoate derivatives (4a-f). A round-bottom flask equipped with a magnetic bar was provided with the corresponding (*Z*)-2-(5-(1,3-dioxoisoindolin-2-yl)pent-1-en-1-yl)benzoate **3a-f** (10.2 mmol) and hydrazine (50% w/w in water, 1.4 mL, 28.6 mmol) in EtOH (51 mL). The reaction mixture was heated in a heating plate at 50 ºC for 2 h. Then, the reaction mixture was let cool down to room temperature and was filtered through a plug of Celite® The filtrate was concentrated under reduced pressure and 1 M HCl (50mL) was added, the aqueous layer was washed with EtOAc (3x25 mL), basified with 4 M NaOH to pH = 9 and extracted with DCM (4 × 25 mL), dried with Na_2SO_4, filtered and concentrated under vacuum.

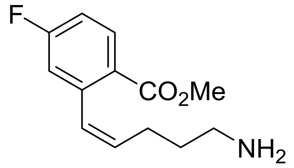

Methyl (*Z*)-2-(5-aminopent-1-en-1-yl)-4-fluorobenzoate (4b). Following the general procedure D **4b** was obtained as a yellow oil (2.5 g, 10.58 mmol, 76%) by acid-basic extraction starting from methyl (*Z*)-2-(5-(1,3-dioxoisoindolin-2-yl)pent-1-en-1-yl)-4-fluorobenzoate **3b** (5.17 g, 14.0 mmol) and hydrazine (50% w/w in water, 2.8 mL, 42 mmol) in EtOH (70 mL). R_f = 0.2 (MeOH). ^{1}H-NMR (300 MHz, $CDCl_3$) δ 8.00 – 7.93 (m, 1H), 6.96 (d, *J* = 8.5 Hz, 2H), 6.84 (d, *J* = 11.6 Hz, 1H), 5.73 (dt, *J* = 11.6, 7.5 Hz, 1H), 3.85 (s, 3H), 2.66 (t, *J* = 7.5 Hz, 2H), 2.17 (app qd, *J* = 7.5, 1.5 Hz, 2H), 1.55 (p, *J* = 7.5 Hz, 2H). ^{13}C {^{1}H} NMR (75 MHz, $CDCl_3$) δ 166.5 (C), 164.2 (d, *J* = 253.3 Hz, C), 142.0 (d, *J* = 8.9 Hz, C), 133.0 (d, *J* = 9.5 Hz, CH), 132.1 (CH), 128.4 (d, *J* = 1.3 Hz, CH), 125.3 (d, *J* = 3.1 Hz, C), 117.5 (d, *J* = 21.7 Hz, CH), 113.7 (d, *J* = 21.5 Hz, CH), 51.9 (CH_3), 41.5 (CH_2), 33.3 (CH_2), 25.6 (CH_2). ^{19}F NMR (282 MHz, $CDCl_3$) δ -107.30. IR (ATR, cm^{-1}): 1720 (C=O), 1604 (C_{Arom}-C_{Arom}), 1577 (C_{Arom}-C_{Arom}). MS (EI) m/z (%): 237.3 (M^+, 8), 221.1 (M^+-NH_2, 6), 205 (M^+-CH_3NH_2, 40), 133 (M^+-CO_2Me-$CH_3CH_2NH_2$,

100). HRMS (ESI) m/z: $[M+H]^+$ Calcd for $[C_{13}H_{17}FNO_2]^+$ 238.1238; Found 238.1248 for compound: **4b.**

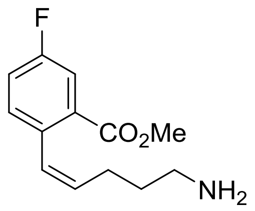

Methyl (*Z*)-2-(5-aminopent-1-en-1-yl)-5-fluorobenzoate (4c). Following the general procedure D **4c** was obtained as a yellow oil in 88:12 *cis*:*trans* ratio (0.30 g, 1.27 mmol, 67%) by acid-basic extraction starting from methyl (*Z*)-2-(5-(1,3-dioxoisoindolin-2-yl)pent-1-en-1-yl)-5-fluorobenzoate **3c** (0.70 g, 1.91 mmol) and hydrazine (50% w/w in water, 0.33 mL, 5.34 mmol) in EtOH (9.6 mL). R_f = 0.2 (MeOH). ^{1}H-NMR (300 MHz, $CDCl_3$: * indicates *trans* diastereoisomer): δ 7.63 (dd, *J* = 9.4, 2.7 Hz, 1H), 7.55* (dt, *J* = 9.4, 2.8 Hz, 1H), 7.40* (dd, *J* = 9.4, 2.7 Hz, 1H), 7.25 (dd, *J* = 8.6, 5.6 Hz, 1H), 7.17 (td, *J* = 8.2, 2.7 Hz, 1H), 7.12 – 7.07* (m, 1H), 6.79 (d, *J* = 11.5 Hz, 1H), 6.05* (dt, *J* = 15.6, 6.9 Hz, 1H), 5.71 (dt, *J* = 11.5, 7.4 Hz, 1H), 3.89* (s, 3H), 3.87 (s, 3H), 2.91* (t, *J* = 7.7 Hz, 2H), 2.65 (d, *J* = 7.0 Hz, 2H), 2.28* (qd, *J* = 7.2, 1.02 Hz, 2H), 2.13 (qd, *J* = 7.4, 1.7 Hz, 2H), 1.84* (m, 2H), 1.54 (p, *J* = 7.2 Hz, 2H), 1.47 – 1.32 (m, 2H). ^{13}C {^{1}H} NMR (75 MHz, $CDCl_3$) δ (ppm): 166.5 (d, *J* = 2.7 Hz, C), 161.0 (d, *J* = 246.8 Hz, C), 135.0 (d, *J* = 3.6 Hz, C), 132.4 (d, *J* = 7.3 Hz, CH), 131.7 (CH), 130.8 (d, *J* = 7.1 Hz, C), 128.2 (CH), 118.7 (d, *J* = 21.0 Hz, CH), 117.2 (d, *J* = 23.2 Hz, CH), 52.2 (CH_3), 41.7 (CH_2), 33.7 (CH_2), 25.7 (CH_2). ^{19}F-NMR (282 MHz, $CDCl_3$; * indicates *trans* diastereoisomer) δ (ppm): -114.9, -116.9*. IR (ATR, cm^{-1}): 1726 (st C=O), 1639 (δ N-H), 1206 (st C-O), 1067 (st C-N). MS (EI) m/z (%): 237 (M^+, 10). HRMS (ESI) m/z: $[M+H]^+$ Calcd for $[C_{13}H_{17}FNO_2]^+$ 238.1238; Found 238.1245 for compound: **4c.**

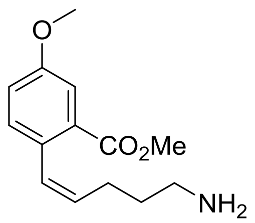

Methyl (*Z*)-2-(5-aminopent-1-en-1-yl)-5-methoxybenzoate (4d). Following the general procedure D, **4d** was obtained as yellow oil (1.81 g, 7.26 mmol, 71%) by FC (EtOAc to EtOAc: MeOH 6:4 to EtOAc: MeOH 6:4 + 1% NH_3) starting from methyl (*Z*)-2-(5-(1,3-dioxoisoindolin-2-yl)pent-1-en-1-yl)-5-methoxybenzoate **3d** (3.58 g, 10.24 mmol) and hydrazine (50%w/w in water, 1.8 mL, 28.7 mmol) in EtOH (52 mL), R_f = 0.06 (EtOAc). ^{1}H-NMR (300 MHz, $CDCl_3$): δ 7.37 (d, *J* = 2.8 Hz, 1H), 7.12 (d, *J* = 8.5 Hz, 1H), 6.94 (dd, *J* = 8.5, 2.8 Hz, 1H), 6.71 (dt, *J* = 11.5, 1.8 Hz, 1H), 5.57 (dt, *J* = 11.5, 7.1 Hz, 1H), 3.79 (s, 3H), 3.76 (s, 3H), 2.58 (t, *J* = 7.1 Hz, 2H), 2.08 (qd, *J* = 7.5, 1.8 Hz, 2H), 1.47 (p, *J* = 7.3 Hz, 2H). ^{13}C {^{1}H} NMR (75 MHz, $CDCl_3$): δ 167.6 (C), 158.2 (C), 132.0 (CH), 131.5 (C), 130.8 (CH), 130.3 (C), 128.9 (CH), 118.0 (CH), 115.0 (CH), 55.6 (CH_3), 52.1 (CH_3), 41.7 (CH_2), 33.5 (CH_2), 25.7 (CH_2). IR (ATR, cm^{-1}): 1719 (C=O st), 1604 (C=C st), 1283 (CO-O st as), 1219 (O-C-C st as). MS (EI) m/z (%): 267.8 (38), 250.1 (MH^+, 100), 195.0 (91), 96.2 (33). HRMS (ESI) m/z: $[M+H]^+$ Calcd for $[C_{14}H_{20}NO_3]^+$ 250.1438; Found 250.1445 for compound: **4d.**

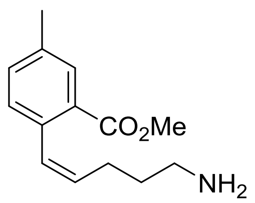

Methyl (*Z*)-2-(5-aminopent-1-en-1-yl)-5-methylbenzoate (4e). Following the general procedure D **4e** was obtained as a yellow oil (0.612 g, 2.62 mmol, 60%) by acid-basic extraction starting from methyl (*Z*)-2-(5-(1,3-dioxoisoindolin-2-yl)pent-1-en-1-yl)-5-methylbenzoate **3e** (1.576 g, 4.337 mmol) and hydrazine (50% w/w in water, 0.84 mL, 13.01 mmol) in EtOH (22 mL). R_f = 0.2 (MeOH). ^{1}H-NMR (300 MHz, $CDCl_3$) δ 7.71 (s,

1H), 7.23 (t, J = 7.4 Hz, 1H), 7.14 (d, J = 7.4 Hz, 1H), 6.78 (t, J = 11.5 Hz, 1H), 5.65 (dt, J = 11.5, 7.5 Hz, 1H), 3.84 (s, 3H), 2.62 (t, J = 7.3 Hz, 2H), 2.33 (s, 3H), 2.13 (qd, J = 7.5 ,1.4 Hz, 2H), 1.50 (p, J = 7.4 Hz, 2H). ^{13}C {^{1}H} NMR (75 MHz, $CDCl_3$) δ 167.8 (C), 150.3 (C), 136.4 (C), 136.0 (C), 132.3 (CH), 131.0 (CH), 130.8 (CH), 130.6 (CH), 129.0 (CH), 51.8 (CH_3), 41.7 (CH_2), 33.7 (CH_2), 25.6 (CH_2), 20.9 (CH_3). IR (ATR, cm^{-1}): 1718 (C=O), 1637 (N-H), 1610 (C_{Arom}-C_{Arom}), 1594 (C_{Arom}-C_{Arom}). MS (EI) m/z (%): 216.1 (M^+-NH_2, 13), 201.1 (M^+-NH_2-CH_3, 32), 173.1 (M^+-$CH_3CH_2NH_2$-CH_3, 13), 128 (M^+-$CH_3CH_2NH_2$-CO_2Me, 100). HRMS (ESI) m/z: $[M+H]^+$ Calcd for $[C_{14}H_{20}NO_2]^+$ 234.1489; Found 234.1497 for compound: **4e.**

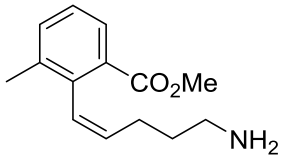

Methyl (*Z*)-2-(5-aminopent-1-en-1-yl)-3-methylbenzoate (4f). Following the general procedure D **4f** was obtained as a yellow oil (0.65 g, 2.786 mmol, 48%) by acid-basic extraction starting from methyl (*Z*)-2-(5-(1,3-dioxoisoindolin-2-yl)pent-1-en-1-yl)-3-methylbenzoate **3f** (2.13 g, 5.86 mmol) and hydrazine (50% w/w in water, 1.2 mL, 17.58 mmol) in EtOH (29 mL). R_f = 0.2 (MeOH). ^{1}H-NMR (300 MHz, $CDCl_3$) δ 7.61 (d, J = 7.7 Hz, 1H), 7.33 (d, J = 7.3 Hz, 1H), 7.21 (t, J = 7.7 Hz, 1H), 6.55 (d, J = 11.3 Hz, 1H), 5.70 (dt, J = 11.3, 7.5 Hz, 1H), 3.82 (s, 3H), 2.56 (t, J = 6.9 Hz, 2H), 2.25 (s, 3H), 1.79 (qd, J = 7.5, 1.4 Hz, 2H), 1.43 (p, J = 7.3 Hz, 2H), 1.31 (bs, 2H). ^{13}C {^{1}H} NMR (75 MHz, $CDCl_3$) δ 168.8 (C), 137.5 (C), 137.3 (C), 133.0 (CH), 131.6 (CH), 131.2 (C), 127.6 (CH), 127.0 (CH), 126.5 (CH), 51.9 (CH_3), 41.6 (CH_2), 33.0 (CH_2), 25.7 (CH_2), 20.3 (CH_3). IR (ATR, cm^{-1}): 1720 (C=O), 1637 (N-H), 1604 (C_{Arom}-C_{Arom}), 1577 (C_{Arom}-C_{Arom}). MS (EI) m/z (%): 201.1 (M^+-NH_2-CH_3, 43), 175.0 (M^+-$CH_3CH_2NH_2$-CH_3, 49), 115 (M^+-$CH_3CH_2CH_2NH_2$-CO_2Me, 100). HRMS (ESI) m/z: $[M+H]^+$ Calcd for $[C_{14}H_{20}NO_2]^+$ 234.1489; Found 234.1499 for compound: **4f.**

General procedure E for the synthesis of (*Z*)-2,3,4,5-tetrahydro-1*H*-benzo[*c*]azonin-1-one derivatives (5a-f). To an oven dried two necked bottom flask provided with a magnetic bar with the corresponding methyl (*Z*)-2-(5-aminopent-1-en-1-yl)benzoate **4a-f** (2.3 mmol) in dry THF (115 mL) under Ar atmosphere, LiHMDS 1 M in THF (6.9 mL, 6.9 mmol) was added dropwise at 0 ºC. The reaction was let stirring at room temperature and monitored by TLC if it was necessary 3 more equiv. of LiHMDS were added to finish the reaction. Once the SM was consumed, MeOH (1 mL) was added, and the solvent was removed under vacuum. The residue was dissolved in DCM and filtrated by Celite®. The filtrated was concentrated under vacuum and purified by silica gel flash chromatography.

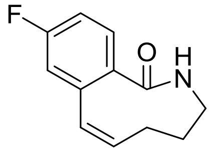

(*Z*)-9-Fluoro-2,3,4,5-tetrahydro-1*H*-benzo[*c*]azonin-1-one (5b). Following the general procedure E **5b** was obtained as a white solid (1.5 g, 7.3 mmol, 73%) by FC (PE/EtOAc 8:2 to PE/EtOAc 7:3) starting from methyl (*Z*)-2-(5-aminopent-1-en-1-yl)-4-fluorobenzoate **4b** (2.5 g, 10 mmol), LiHMDS 1 M in THF (60 mL, 60 mmol) in THF (1500 mL). R_f = 0.71 (MeOH). mp: 184-186 ºC. ^{1}H-NMR (300 MHz, $CDCl_3$) δ 7.31 (dd, *J* = 8.4, 5.6 Hz, 1H), 7.04 (tdd, *J* = 8.5, 2.6, 0.6 Hz, 1H), 6.92 (dd, *J* = 9.2, 2.3 Hz, 1H), 6.57 (d, *J* = 10.8 Hz, 1H), 6.46 (s, 1H), 6.03 (dt, *J* = 10.8, 8.4 Hz, 1H), 3.20 (bs, 2H), 2.01 (bs, 2H), 1.58 – 1.50 (m, 2H). ^{13}C {^{1}H} NMR (75 MHz, $CDCl_3$) δ 173.2 (C), 162.4 (d, *J* = 249.0 Hz, C), 138.7 (d, *J* = 8.2 Hz, C), 134.7 (CH), 133.2 (d, *J* = 3.5 Hz, C), 127.5 (d, *J* = 1.5 Hz, CH), 127.1 (d, *J* = 8.8 Hz, CH), 115.3 (d, *J* = 21.3 Hz, CH), 114.3 (d, *J* = 21.8 Hz, CH), 45.3 (CH_2), 29.0 (CH_2), 28.8 (CH_2). ^{19}F NMR (282 MHz, $CDCl_3$) δ -112.15. IR (ATR, cm^{-1}): 3281 (N-H), 2929 (C-H), 1650 (N-C=O). MS (EI) m/z (%): 205.1 (M^+, 62), 176.1 (M^+-CH_3NH_2, 100). HRMS (ESI) m/z: $[M+H]^+$ Calcd for $[C_{12}H_{13}FNO]^+$ 206.0981; Found 206.0986 for compound: **5b.**

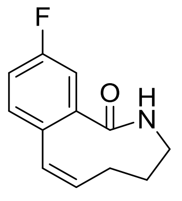

(*Z*)-10-Fluoro-2,3,4,5-tetrahydro-1*H*-benzo[*c*]azonin-1-one (5c). Following the general procedure E **5c** was obtained as a white solid (0.083 g, 0.40 mmol, 44%) by FC (PE/EtOAc 8:2 to PE/EtOAc 7:3) starting from methyl (*Z*)-2-(5-aminopent-1-en-1-yl)-4-fluorobenzoate **4c** (0.22 g, 0.91 mmol), LiHMDS 1 M in THF (2.7 mL, 2.7 mmol) in THF (130 mL). R_f = 0.32 (PE/EtOAc 7:3). mp: 175-178 ºC. ^{1}H NMR (300 MHz, $CDCl_3$): δ 7.21 – 7.09 (m, 1H), 7.09 – 6.90 (m, 3H), 6.53 (d, *J* = 10.7 Hz, 1H), 6.01 (dt, *J* = 10.9, 8.4 Hz, 1H), 3.52 – 2.89 (m, 2H), 2.25 – 1.73 (m, 2H), 1.52 (p, *J* = 5.2 Hz, 2H). ^{13}C {^{1}H} NMR (75 MHz, $CDCl_3$) δ (ppm): 172.7 (C), 161.6 (d, *J* = 247.4 Hz, C), 138.8 (d, *J* = 7.0 Hz, C), 135.2 (CH), 132.1 (d, *J* = 3.5 Hz, C), 130.3 (d, *J* = 7.9 Hz, CH), 127.6 (CH), 115.8 (d, *J* = 21.2 Hz, CH), 112.4 (d, *J* = 23.0 Hz, CH), 45.3 (CH_2), 29.1 (CH_2), 28.9 (CH_2). ^{19}F NMR (282 MHz, $CDCl_3$) δ -114.54. IR (ATR, cm^{-1}): 3187 (N-H), 1651 (N-C=O). MS (EI) m/z (%): 205 (M^+, 83), 176 (M^+-CH_3NH_2, 100). HRMS (ESI) m/z: $[M+H]^+$ Calcd for $[C_{12}H_{13}FNO]^+$ 206.0981; Found 206.0984 for compound: **5c.**

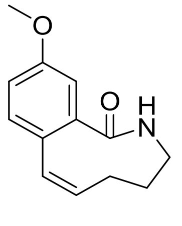

(*Z*)-10-Methoxy-2,3,4,5-tetrahydro-1*H*-benzo[*c*]azonin-1-one (5d). Following the general procedure E, **5d** was obtained as a white solid (352.4 mg, 1.62 mmol, 43%) by FC (PE/EtOAc 2:8) starting from methyl (*Z*)-2-(5-aminopent-1-en-1-yl)-5-methoxybenzoate **4d** (940 mg, 3.77 mmol), LiHMDS 1 M in THF (18.8 mL, 18.8 mmol) in THF (754 mL) R_f = 0.32 (PE/EtOAc 2:8). mp: 128-137 °C. ^{1}H-NMR (300 MHz,

$CDCl_3$): δ 7.07 (d, *J* = 8.4 Hz, 1H), 6.89 (dd, *J* = 8.4, 2.7 Hz, 1H), 6.84 (d, *J* = 2.7 Hz, 1H), 6.54 (d, *J* = 10.7 Hz, 1H), 5.98 (dt, *J* = 10.6, 8.4 Hz, 1H), 3.82 (s, 3H), 3.42 – 3.01 (m, 2H), 2.27 – 1.85 (m, 2H), 1.57 – 1.47 (m, 2H). ^{13}C {^1H} NMR (75 MHz, $CDCl_3$): δ 173.7 (C), 158.7 (C), 138.1 (C), 134.7 (CH), 129.6 (CH), 128.4 (CH), 128.4 (C), 114.9 (CH), 110.2 (CH), 55.5 (CH_3), 45.4 (CH_2), 29.4 (CH_2), 28.9 (CH_2). IR (ATR, cm^{-1}): 3284 (N-H st), 3209 (N-H st), 1650 (C=O st). MS (EI) m/z (%): 217.1 (M^+, 83), 188.0 (100), 160.0 (67), 115.0 (65), 89.0 (32), 62.9 (32). HRMS (ESI) m/z: $[M+H]^+$ Calcd for $[C_{13}H_{16}NO_2]^+$ 218.1176; Found 218.1183 for compound: **5d.**

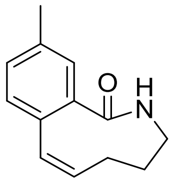

(*Z*)-10-Methyl-2,3,4,5-tetrahydro-1*H*-benzo[*c*]azonin-1-one (5e). Following the general procedure E **5e** was obtained as a white solid (294 g, 1.46 mmol, 67%) by FC (PE/EtOAc 1:1 to EtOAc/MeOH 98:2) starting from methyl (*Z*)-2-(5-aminopent-1-en-1-yl)-5-methylbenzoate **4e** (0.521 g, 2.23 mmol), LiHMDS 1 M in THF (6.7 mL, 7.86 mmol) in THF (262 mL). R_f = 0.31 (EtOAc). mp: 173-175 ºC. ^1H NMR (300 MHz, $CDCl_3$) δ 7.36 (bs, 1H), 7.13 (d, *J* = 10.6 Hz, 2H), 7.04 (d, *J* = 7.6 Hz, 1H), 6.56 (d, *J* = 10.7 Hz, 1H), 5.97 (dd, *J* = 18.7, 8.6 Hz, 1H), 3.24 (bs, 1H), 3.06 (bs, 1H), 2.35 (s, 3H), 2.07 (bs, 1H), 1.86 (bs, 1H), 1.49 (bs, 2H). ^{13}C {^1H} NMR (75 MHz, $CDCl_3$) δ 174.5 (C), 137.0 (C), 136.8 (C), 134.3 (CH), 133.0 (C), 129.3 (CH), 128.3 (CH), 128.0 (CH), 125.5 (CH), 45.2 (CH_2), 29.1 (CH_2), 28.8 (CH_2), 20.9 (CH_3). IR (ATR, cm^{-1}): 3276 (N-H), 2858 (C-H), 1644 (N-C=O). MS (EI) m/z (%): 201.1 (M^+, 98), 172.1 (M^+-CH_3NH_2, 99). HRMS (ESI) m/z: $[M+H]^+$ Calcd for $[C_{13}H_{16}NO]^+$ 202.1232; Found 202.1237 for compound: **5e.**

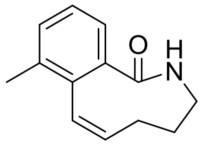

(*Z*)-8-Methyl-2,3,4,5-tetrahydro-1*H*-benzo[*c*]azonin-1-one (5f). Following the general procedure *J* **5f** was obtained as a white solid (0.454 g, 2.255 mmol, 86%) by FC (PE/EtOAc 1:1 to EtOAc/MeOH 98:2) starting from methyl (*Z*)-2-(5-aminopent-1-en-1-yl)-4-fluorobenzoate **4f** (0.610 g, 2.61 mmol), LiHMDS 1 M in THF (8 mL, 7.84 mmol) in THF (260 mL). R_f =0.30 (EtOAc). mp: 188-191 ºC. ^{1}H-NMR (300 MHz, $CDCl_3$) δ 7.26 – 7.20 (m, 2H), 7.14 (dd, *J* = 6.3, 2.5 Hz, 1H), 6.51 (bs + d, *J* = 10.8 Hz, 2H), 6.03 (dt, *J* = 10.8, 6.1 Hz, 1H), 3.35 – 3.23 (m, 1H), 3.17 – 2.99 (m, 1H), 2.23 (s, 3H), 2.25 – 2.10 (m, 1H), 1.88 – 1.69 (m, 1H), 1.58 – 1.49 (m, 2H). ^{13}C {^{1}H} NMR (75 MHz, $CDCl_3$) δ 174.4 (C), 137.0 (C), 136.6 (C), 135.1 (C), 133.8 (CH), 129.7 (CH), 127.8 (CH), 127.3 (CH), 122.3 (CH), 45.2 (CH_2), 29.2 (CH_2), 28.9 (CH_2), 19.9 (CH_3). IR (ATR, cm^{-1}): 3278 (N-H), 2853 (C-H), 1649 (N-C=O). MS (EI) m/z (%): 201.1 (M^+, 77), 172.1 (M^+-CH_3NH_2, 42). HRMS (ESI) m/z: $[M+H]^+$ Calcd for $[C_{13}H_{16}NO]^+$ 202.1232; Found 202.1236 for compound: **5f.**

General procedure F for the synthesis of 2,3-dihydropyrrolo[1,2-b]isoquinolin-5(1H)-one derivatives derivatives (6a-c and 6e). To reaction tube provided with a magnetic bar with the corresponding (*Z*)-2,3,4,5-tetrahydro-1*H*-benzo[*c*]azonin-1-one (**5a-c** or **5e**) (20.0 mg, 0.108 mmol), a Stock solution of diphenylphosphoric acid (0.7 mg, 0.003 mmol) in dry dichloromethane (220 μL) was added at 25 ºC followed by the addition of *N*-bromosuccinimide (19 mg, 0.108 mmol). The reaction mixture was followed by TLC and when all starting material was consumed the solvent was evaporated under vacuum and Et_2O (1 mL) and std. aq. solution of $NaHCO_3$ (1 mL) were added. The layers were separated, and aqueous phase was extracted with Et_2O (3 × 1 mL). All organic layers were washed with water (3 × 1 mL), brine (1 mL), dried with Na_2SO_4, filtrated and concentrated under vacuum. The crude was purified by silica gel flash chromatography.

General procedure G for the synthesis of 2,3-dihydropyrrolo[1,2-*b*]isoquinolin-5(1*H*)-one derivatives derivatives (6d and 6f). To reaction tube provided with a magnetic bar with the corresponding (*Z*)-2,3,4,5-tetrahydro-1*H*-benzo[*c*]azonin-1-one (**5d** or **5f**) (0.108 mmol), a Stock solution of diphenylphosphoric acid (0.7 mg, 0.003 mmol) in dry dichloromethane (220 μL) was added at 25 ºC followed by the addition of *N*-bromosuccinimide (19 mg, 0.108 mmol). The reaction mixture was followed by TLC. Once the starting material was consumed, DBU (19.4 μg, 0.130 mmol) were added to the reaction mixture at 25 ºC. The new reaction mixture was let stirring 16 h. Then, the solvent was evaporated under vacuum and Et_2O (1 mL) and std. aq. solution of NH_4Cl (1 mL) were added. The layers were separated, and aqueous phase was extracted with Et_2O (3 × 1 mL). All organic layers were washed with water (3 × 1 mL), brine (1 mL), dried with Na_2SO_4, filtrated, and concentrated under vacuum. The crude was purified by silica gel flash chromatography (CH_2Cl_2 to CH_2Cl_2/MeOH 99:1).

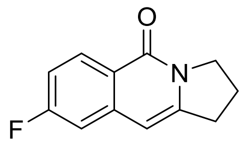

8-Fluoro-2,3-dihydropyrrolo[1,2-*b*]isoquinolin-5(1*H*)-one (6b). Following the general procedure F **6b** was obtained as a white solid (199 mg, 0.980 mmol, 98%) by FC (CH_2Cl_2 to CH_2Cl_2/MeOH 99:1) starting from (*Z*)-9-fluoro-2,3,4,5-tetrahydro-1*H*-benzo[*c*]azonin-1-one **5b** (205 mg, 1 mmol), NBS (179.8 mg, 1.01 mmol), diphenyl phosphoric acid (6.3 mg; 0.025mmol) and CH_2Cl_2 (2 mL). R_f = 0.3 (DCM/MeOH 99:1). mp: 146-149 ºC. ^{1}H-NMR (300 MHz, $CDCl_3$) δ 8.26 (dd, *J* = 8.8, 5.9 Hz, 1H), 7.02 – 6.91 (m, 2H), 6.21 (s, 1H), 4.05 (t, *J* = 7.2, 2H), 2.97 (app td, *J* = 7.7, 1.1 Hz, 2H), 2.09 (p, *J* = 7.5 Hz, 2H). ^{13}C {^{1}H} NMR (75 MHz, $CDCl_3$) δ 166.7 (C), 162.2 (d, *J* = 189.7 Hz, C), 145.3 (C), 140.3 (d, *J* = 10.3 Hz, C), 130.4 (d, *J* = 10.1 Hz, CH), 121.3 (d, *J* = 1.4 Hz, C), 114.2 (d, *J* = 23.6 Hz, CH), 110.2 (d, *J* = 21.8 Hz, CH), 99.8 (d, *J* = 3.3 Hz, CH), 48.0 (CH_2), 31.3 (CH_2), 22.0 (CH_2). ^{19}F NMR (282 MHz, $CDCl_3$) δ - 107.58. IR (ATR, cm^{-1}): 2993 (C-H), 1659 (N-C=O), 1629 (C=C). MS (EI) m/z (%): 202.1 (M^+, 100) HRMS (ESI) m/z: $[M+H]^+$ Calcd for $[C_{12}H_{11}FNO]^+$ 204.0825; Found 204.0827 for compound: **6b.**

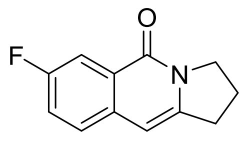

7-Fluoro-2,3-dihydropyrrolo[1,2-*b*]isoquinolin-5(1*H*)-one (6c). Following the general procedure F **6c** was obtained as a white solid (21.7 mg, 0.107 mmol, 68%) by FC (CH_2Cl_2 to CH_2Cl_2/MeOH 99:1) starting from (*Z*)-10-fluoro-2,3,4,5-tetrahydro-1*H*-benzo[*c*]azonin-1-one **5c** (32 mg, 0.156 mmol), NBS (27.9 mg, 0.157 mmol) and a Stock solution of diphenylphosphoric acid (1.0 mg, 0.004 mmol) in dry CH_2Cl_2 (310 µL). R_f = 0.3 (DCM/MeOH 99:1). mp: 213-216 ºC. ^{1}H-NMR (300 MHz, $CDCl_3$) δ 8.05 (dd, *J* = 9.5, 2.7 Hz, 1H), 7.48 (dd, *J* = 8.5, 5.2 Hz, 1H), 7.35 (td, *J* = 8.5, 2.7 Hz, 1H), 6.43 (s,

1H), 4.21 (t, J = 7.2 Hz, 2H), 3.12 (t, J = 7.6 Hz, 2H), 2.32 – 2.16 (m, 2H). ^{13}C {^{1}H} NMR (75 MHz, $CDCl_3$) δ 160.8 (d, J = 245.7 Hz, C), 160.7 (d, J = 3.8 Hz, C), 143.1 (d, J = 2.7 Hz, C), 134.7 (d, J = 1.9 Hz, C), 127.7 (d, J = 7.8 Hz, CH), 126.1 (d, J = 7.8 Hz, C), 120.8 (d, J = 23.8 Hz, CH), 112.3 (d, J = 22.6 Hz, CH), 99.8 (CH), 48.0 (CH_2), 31.0 (CH_2), 22.0 (CH_2). ^{19}F NMR (282 MHz, $CDCl_3$) δ -115.02. IR (ATR, cm^{-1}): 2992 (C-H), 1657 (N-C=O), 1626 (C=C). MS (EI) m/z (%): 202.1 (M^+, 100). HRMS (ESI) m/z: $[M+H]^+$ Calcd for $[C_{12}H_{11}FNO]^+$ 204.0825; Found 204.0828 for compound: **6c.**

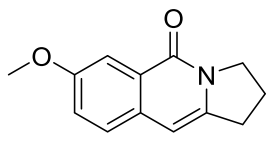

7-Methoxy-2,3-dihydropyrrolo[1,2-*b*]isoquinolin-5(1*H*)-one (6d). Following the general procedure G, **6d** was obtained as a white oil (4.4 mg, 0.0204 mmol, 41%) by FC (CH_2Cl_2/EtOAc 1:1) starting from (*Z*)-10-methoxy-2,3,4,5-tetrahydro-1*H*-benzo[*c*]azonin-1-one **5d** (10.9 mg, 0.05 mmol), NBS (13.4 mg, 0.075 mmol), a Stock solution of diphenylphosphoric acid (0.314 mg, 0.00125 mmol) in dry CH_2Cl_2 (0.1 mL) and DBU (15 μL, 0.1 mmol). R_f = 0.27 (CH_2Cl_2/EtOAc 1:1). ^{1}H-NMR (300 MHz, $CDCl_3$): δ 7.80 (d, J = 2.8 Hz, 1H), 7.39 (d, J = 8.7 Hz, 1H), 7.22 (dd, J = 8.7, 2.7 Hz, 1H), 6.40 (app s, 1H), 4.20 (t, J = 7.1 Hz, 2H), 3.92 (s, 3H), 3.09 (td, J = 7.6, 1.4 Hz, 2H), 2.21 (p, J = 7.4 Hz, 2H). ^{13}C {^{1}H} NMR (75 MHz, $CDCl_3$): 161.4 (C), 158.1 (C), 141.5 (C), 132.5 (C), 127.3 (CH), 125.8 (C), 123.0 (CH), 107.2 (CH), 100.4 (CH), 55.8 (CH_3), 48.3 (CH_2), 31.1 (CH_2), 22.3 (CH_2). IR (ATR, cm^{-1}): 2969 (C-H st), 1738 (C=O st), 1216 (C_{Arom}-O-C st as) MS (EI) m/z (%): 217.1 (16), 216.1 (70), 215.1 (M^+, 100), 213.9 (13). HRMS (ESI) m/z: $[M+H]^+$ Calcd for $[C_{13}H_{14}NO_2]^+$ 216.1019; Found 216.1028 for compound: **6d.**

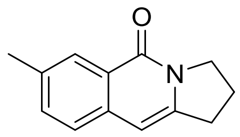

7-Methyl-2,3-dihydropyrrolo[1,2-*b*]isoquinolin-5(1*H*)-one. (6e). Following the general procedure F **6e** was obtained as a white solid (17.8 mg, 0.09 mmol, 79%) by FC (CH_2Cl_2 to CH_2Cl_2/MeOH 99:1) starting from (*Z*)-10-methyl-2,3,4,5-tetrahydro-1*H*-benzo[*c*]azonin-1-one **5e** (22.8 mg, 0.113 mmol), NBS (20.4 mg, 0.114 mmol) and a Stock solution of diphenylphosphoric acid (0.7 mg, 0.003 mmol) in dry CH_2Cl_2 (230 μL). R_f = 0.3 (DCM/MeOH 99:1). mp: 112-113 ºC. ^{1}H NMR (300 MHz, $CDCl_3$) δ 8.21 – 8.14 (m, 1H), 7.41 (dd, *J* = 8.1, 1.8 Hz, 1H), 7.35 (d, *J* = 8.1 Hz, 1H), 6.37 (t, *J* = 1.6 Hz, 1H), 4.17 (t, *J* = 7.14 Hz, 2H), 3.08 (td, *J* = 7.6, 1.4 Hz, 2H), 2.46 (s, 3H), 2.18 (p, *J* = 7.5 Hz, 2H). ^{13}C {^{1}H} NMR (75 MHz, $CDCl_3$) δ 161.5 (C), 142.7 (C), 135.8 (C), 135.5 (C), 133.5 (CH), 126.8 (CH), 125.4 (CH), 124.6 (C), 100.2 (CH), 47.9 (CH_2), 31.1 (CH_2), 22.1 (CH_2), 21.4 (CH_3). IR (ATR, cm^{-1}): 2963 (C-H), 1645 (N-C=O), 1624 (C=C). MS (EI) m/z (%): 198.1 (M^+, 100). HRMS (ESI) m/z: $[M+H]^+$ Calcd for $[C_{13}H_{14}NO]^+$ 200.1075; Found 200.1078 for compound: **6e.**

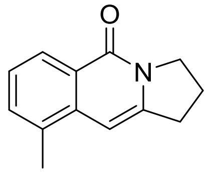

9-Methyl-2,3-dihydropyrrolo[1,2-*b*]isoquinolin-5(1*H*)-one (6f). Following the general procedure G **6f** was obtained as a white solid (5.9 mg, 0.03 mmol, 26%) by FC (CH_2Cl_2 to CH_2Cl_2/MeOH 99:1) starting from (*Z*)-8-methyl-2,3,4,5-tetrahydro-1*H*-benzo[*c*]azonin-1-one **5f** (23 mg, 0.114 mmol), NBS (20.5 mg, 0.115 mmol), a Stock solution of diphenylphosphoric acid (0.7 mg, 0.003 mmol) in dry CH_2Cl_2 (230 μL) and DBU (17 μL, 0.114 mmol). R_f = 0.3 (DCM/MeOH 99:1). mp: 103-105 ºC. ^{1}H-NMR (300 MHz, $CDCl_3$): δ 8.27 (d, *J* = 8.1 Hz, 1H), 7.42 (d, *J* = 7.0 Hz, 1H), 7.29 (t, *J* = 7.6 Hz,

1H), 6.53 (t, $J = 1.7$ Hz, 1H), 4.19 (t, $J = 7.2$ Hz, 2H), 3.13 (td, $J = 7.7$, 1.5 Hz, 2H), 2.50 (s, 3H), 2.20 (p, $J = 7.5$ Hz, 2H). ^{13}C {^{1}H} NMR (75 MHz, $CDCl_3$) δ 161.8 (C), 143.4 (C), 137.0 (C), 132.7 (CH), 132.5 (C), 125.4 (CH), 125.1 (CH), 124.8 (C), 97.0 (CH), 48.0 (CH_2), 31.4 (CH_2), 22.0 (CH_2), 19.2 (CH_3). IR (ATR, cm^{-1}): 2925 (C-H), 1653 (N-C=O), 1623 (C=C). MS (EI) m/z (%): 198.1 (M^+, 100). HRMS (ESI) m/z: $[M+H]^+$ Calcd for $[C_{13}H_{14}NO]^+$ 200.1075; Found 200.1080 for compound: **6f.**

General procedure H for the synthesis of 10-iodo-2,3-dihydropyrrolo[1,2-*b*]isoquinolin-5(1*H*)-one derivatives (7a-f). To reaction tube provided with a magnetic bar with the corresponding (*Z*)-2,3,4,5-tetrahydro-1*H*-benzo[*c*]azonin-1-one **5a-f** (27.4 mg, 0.145 mmol), a Stock solution of diphenylphosphoric acid (0.9 mg, 0.004 mmol) in dry dichloromethane (290 µL) was added at 25 ºC followed by the addition of *N*-iodosuccinimide (98 mg, 0.434 mmol). The reaction mixture was followed by TLC and when all starting material was consumed the solvent was evaporated under vacuum and Et_2O (1 mL) and std. aq. solution of $Na_2S_2O_3$ (1 mL) were added, the mixture was let stirring for 30 min. at room temperature. Then, the layers were separated, and aqueous phase was extracted with Et_2O (3 × 1 mL). All organic layers were washed with water (3 × 1 mL), brine (1 mL), dried with Na_2SO_4, filtrated and concentrated under vacuum. The crude was purified by silica gel flash chromatography.

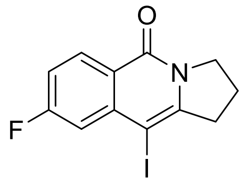

8-Fluoro-10-iodo-2,3-dihydropyrrolo[1,2-*b*]isoquinolin-5(1*H*)-one (7b). Following the general procedure H **7b** was obtained as a pale yellow solid (21.9 mg, 0.067 mmol, 86%) by FC (CH_2Cl_2 to CH_2Cl_2/MeOH 99:1) starting from (*Z*)-9-fluoro-2,3,4,5-tetrahydro-1*H*-benzo[*c*]azonin-1-one **5b** (15.9 mg, 0.078 mmol), NIS (52.3 mg, 0.232 mmol) and a Stock solution of diphenylphosphoric acid (0.5 mg, 0.002 mmol) in dry CH_2Cl_2 (160 µL). R_f = 0.3 (DCM/MeOH 99:1). mp: 165-167 ºC. ^{1}H NMR (300 MHz, $CDCl_3$) δ 8.40 (dd, *J* = 8.8, 5.9 Hz, 1H), 7.45 (dd, *J* = 10.5, 2.4 Hz, 1H), 7.15 (ddd, *J* = 8.9, 8.0, 2.5 Hz, 1H), 4.43 – 4.29 (t, *J* = 7.45 Hz, 2H), 3.27 (t, *J* = 7.8 Hz, 2H), 2.27 (p, *J* = 7.7 Hz, 2H). ^{13}C {^{1}H} NMR (75 MHz, $CDCl_3$) δ 165.9 (d, *J* = 252.4 Hz, C), 160.2 (C), 148.4 (C) , 140.9 (d, *J* = 10.4 Hz, C), 131.0 (d, *J* = 10.0 Hz, CH), 121.5 (d, *J* = 1.6 Hz,

C), 115.1 (d, J = 23.6 Hz, CH), 114.9 (d, J = 24.8 Hz, CH), 67.2 (d, J = 3.1 Hz, C), 50.3 (CH_2), 37.7 (CH_2), 20.8 (CH_2). ^{19}F NMR (282 MHz, $CDCl_3$) δ -105.34. IR (ATR, cm^{-1}): 1635 (N-C=O), 1607 (C=C). MS (EI) m/z (%): 328.8 (M^+, 100). HRMS (ESI) m/z: $[M+H]^+$ Calcd for $[C_{12}H_{10}FINO]^+$ 329.9791; Found 329.9792 for compound: **7b.**

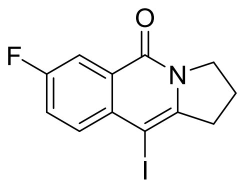

7-Fluoro-10-iodo-2,3-dihydropyrrolo[1,2-*b*]isoquinolin-5(1*H*)-one (7c). Following the general procedure H **7c** was obtained as a pale yellow solid (18 mg, 0.055 mmol, 81%) by FC (CH_2Cl_2 to CH_2Cl_2/MeOH 99:1) starting from (*Z*)-10-fluoro-2,3,4,5-tetrahydro-1*H*-benzo[*c*]azonin-1-one **7c** (13.8 mg, 0.067 mmol), NIS (45.4 mg, 0.202 mmol) and a Stock solution of diphenylphosphoric acid (0.2 mg, 0.002 mmol) in dry CH_2Cl_2 (140 µL). R_f = 0.3 (DCM/MeOH 99:1). mp: 133-134 ºC. ^{1}H NMR (300 MHz, $CDCl_3$) δ 8.05 (dd, J = 9.1, 2.8 Hz, 1H), 7.78 (dd, J = 9.0, 5.0 Hz, 1H), 7.41 (ddd, J = 8.9, 8.0, 2.8 Hz, 1H), 4.37 (t, J = 7.3 Hz, 2H), 3.26 (t, J = 7.7 Hz, 2H), 2.27 (p, J =7.5 Hz, 2H). ^{13}C {^{1}H} NMR (75 MHz, $CDCl_3$) δ 161.5 (d, J = 248.1 Hz, C), 160.1 (d, J = 3.5 Hz, C), 146.1 (d, J = 2.7 Hz, C), 134.6 (d, J = 1.9 Hz, C), 131.8 (d, J = 7.8 Hz, CH), 126.1 (d, J = 7.7 Hz, C), 121.5 (d, J = 23.4 Hz, CH), 112.6 (d, J = 23.1 Hz, CH), 67.5 (C), 50.3 (CH_2), 37.4 (CH_2), 20.9 (CH_2). ^{19}F NMR (282 MHz, $CDCl_3$) δ -114.30. IR (ATR, cm^{-1}): 1641 (N-C=O), 1605 (C=C). MS (EI) m/z (%): 328.9 (M^+, 100). HRMS (ESI) m/z: $[M+H]^+$ Calcd for $[C_{12}H_{10}FINO]^+$ 329.9791; Found 329.9800 for compound: **7c.**

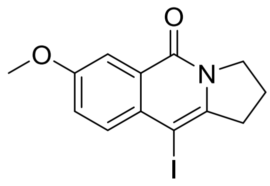

10-Iodo-7-methoxy-2,3-dihydropyrrolo[1,2-*b*]isoquinolin-5(1*H*)-one (7d). Following the general procedure H, **7d** was obtained as a yellow solid (11.0 mg, 0.0354 mmol, 71%) by FC (CH_2Cl_2/EtOAc 8:2) starting from (*Z*)-10-methoxy-2,3,4,5-tetrahydro-1*H*-benzo[*c*]azonin-1-one **5d** (10.9 mg, 0.05 mmol), NIS (33.7 mg, 0.15 mmol) and a Stock solution of diphenylphosphoric acid (0.313 mg, 0.00125 mmol) in dry CH_2Cl_2 (0.1 mL). R_f = 0.33 (CH_2Cl_2/EtOAc 8:2). mp: 167 - 168 °C. ^{1}H NMR (300 MHz, $CDCl_3$): 7.78 (d, *J* = 2.8 Hz, 1H), 7.68 (d, *J* = 8.9 Hz, 1H), 7.27 (dd, *J* = 8.9, 2.8 Hz, 1H), 4.40 – 4.32 (m, 2H), 3.93 (s, 3H), 3.23 (t, *J* = 7.7 Hz, 2H), 2.23 (p, *J* = 7.7 Hz, 2H). ^{13}C {^{1}H} NMR (75 MHz, $CDCl_3$): 160.7 (C), 158.8 (C), 144.4 (C), 132.2 (C), 131.1 (CH), 125.9 (C), 123.4 (CH), 107.5 (CH), 68.6 (C), 55.9 (CH_3), 50.4 (CH_2), 37.2 (CH_2), 21.1 (CH_2). IR (ATR, cm^{-1}): 2922 (C-H st), 1637 (C=O st), 1597 (C_{Arom}-C_{Arom} st). MS (EI) m/z (%): 341.0 (M^+, 100), 326.0 (M^+ – CH_3, 47), 214.1 (M^+ – I, 12), 126.9 (I^+, 36). HRMS (ESI) m/z: $[M+H]^+$ Calcd for $[C_{13}H_{13}INO_2]^+$ 341.9985; Found 341.9996 for compound: **7d.**

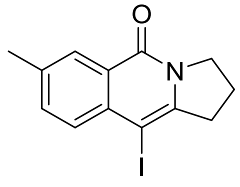

10-Iodo-7-methyl-2,3-dihydropyrrolo[1,2-*b*]isoquinolin-5(1*H*)-one (7e). Following the general procedure H, **7e** was obtained as a pale yellow solid (32.7 mg, 0.1 mmol, 85%) by FC (CH_2Cl_2 to CH_2Cl_2/MeOH 99:1) starting from (*Z*)-10-methyl-2,3,4,5-tetrahydro-1*H*-benzo[*c*]azonin-1-one **5e** (23.4 mg, 0.116 mmol), NIS (78.5 mg, 0.349 mmol) and a Stock solution of diphenylphosphoric acid (0.7 mg, 0.003 mmol) in dry CH_2Cl_2 (230 μL). R_f = 0.3 (DCM/MeOH 99:1). mp: 143-145 ºC. ^{1}H NMR (300 MHz,

$CDCl_3$) δ 8.18 – 8.12 (t, *J* = 0.9 Hz, 1H), 7.61 (d, *J* = 8.3 Hz, 1H), 7.46 (dd, *J* = 8.4, 1.9 Hz, 1H), 4.33 (t, *J* = 7.3 Hz, 2H), 3.21 (t, *J* = 7.7 Hz, 2H), 2.48 (s, 3H), 2.21 (p, *J* = 7.6 Hz, 2H). ^{13}C {^{1}H} NMR (75 MHz, $CDCl_3$) δ 160.9 (C), 145.7 (C), 136.8 (C), 135.7 (C), 134.5 (CH), 129.2 (CH), 127.2 (CH), 124.7 (C), 68.7 (C), 50.2 (CH_2), 37.3 (CH_2), 21.1 (CH_3), 20.8 (CH_2). IR (ATR, cm^{-1}): 1645 (N-C=O), 1615 (C=C). MS (EI) m/z (%): 328.9 (M^+, 100). HRMS (ESI) m/z: $[M+H]^+$ Calcd for $[C_{13}H_{13}INO]^+$ 326.0042; Found 326.0046 for compound: **7e.**

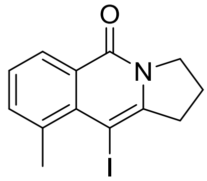

10-Iodo-9-methyl-2,3-dihydropyrrolo[1,2-*b*]isoquinolin-5(1*H*)-one (7f). Following the general procedure H, **7f** was obtained as a yellow solid (14.4 mg, 0.046 mmol, 46%), by FC (PE/EtOAc 6:4 to 3:7), starting from (*Z*)-8-methyl-2,3,4,5-tetrahydro-1*H*-benzo[*c*]azonin-1-one **5f** (20.1 mg, 0.1 mmol), *N*-Iodosuccinimide (67.5 mg, 0.3 mmol), and a stock solution of of diphenyl phosphate (0.63 mg, 0.0025 mmol) in dry CH_2Cl_2 (0.2 mL). R_f = 0.60 (PE/EtOAc 3:7). mp: 105-107 °C. ^{1}H NMR (300 MHz, $CDCl_3$): δ 8.41 (d, *J* = 7.8 Hz, 1H), 7.48 (d, *J* = 7.0 Hz, 1H), 7.33 (t, *J* = 7.7 Hz, 1H), 4.36 (t, *J* = 7.4 Hz, 2H), 3.26 (t, *J* = 7.8 Hz, 2H), 3.03 (s, 3H), 2.19 (p, *J* = 7.7 Hz, 2H). ^{13}C {^{1}H} NMR (75 MHz, $CDCl_3$): δ 161.5 (C) 148.1 (C), 137.4 (CH), 136.4 (C), 134.7 (C), 127.4 (CH), 127.2 (C), 126.6 (CH), 61.5 (C), 51.0 (CH_2), 40.8 (CH_2), 26.4 (CH_3), 20.9 (CH_2). IR (ATR, cm^{-1}): 1639 (C=O st), 1585 (C_{Arom}-C_{Arom} st). HRMS (ESI) m/z: $[M+H]^+$ Calcd for $[C_{13}H_{13}INO]^+$ 326.0042; Found 326.0045 for compound: **7f.**

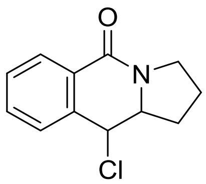

10-Chloro-2,3,10,10a-tetrahydropyrrolo[1,2-*b*]isoquinolin-5(1*H*)-one (8a). To reaction tube provided with a magnetic bar with (*Z*)-2,3,4,5-tetrahydro-1*H*-benzo[*c*]azonin-1-one **5a** (20, 0.108 mmol), a Stock solution of diphenylphosphoric acid (0.7 mg, 0.003 mmol) in dry dichloromethane (220 μL) was added at 25 ºC followed by the addition of *N*-chlorosuccinimide (14.4 mg, 0.108 mmol). The reaction mixture was followed by TLC (CH_2Cl_2/MeOH 98:2) when all starting material was consumed the solvent was evaporated under vacuum and Et_2O (1 mL) and std. aq. solution of $Na_2S_2O_3$ (1 mL) were added, the mixture was let stirring for 30 min. at room temperature. Then, the layers were separated, and aqueous phase was extracted with Et_2O (3 × 1 mL). All organic layers were washed with water (3 × 1 mL), brine (1 mL), dried with Na_2SO_4, filtrated and concentrated under vacuum. The crude was purified by silica gel flash chromatography (DCM/PE/EtOAc 2:1:0.05 to 2:1:0.1) to obtain 10-chloro-2,3,10,10a-tetrahydropyrrolo[1,2-*b*]isoquinolin-5(1*H*)-one as a white solid (17.2 mg, 0.078 mmol, 72%). mp: 118-121 ºC. ^{1}H NMR (300 MHz, $CDCl_3$): δ 8.16 – 8.06 (m, 1H), 7.55 – 7.42 (m, 2H), 7.36 (dd, *J* = 7.9, 5.6 Hz, 1H,), 5.12 (d, *J* = 2.5 Hz, 1H), 4.09 (t, *J* = 7.7 Hz, 1H), 3.90 – 3.76 (m, 1H), 3.73 – 3.58 (m, 1H), 2.43 – 2.24 (m, 1H), 2.24 – 2.08 (m, 2H), 1.99 – 1.79 (m, 1H). ^{13}C {^{1}H} NMR (75 MHz, $CDCl_3$): δ (75 MHz, $CDCl_3$) δ: 162.4 (C), 138.3 (C), 132.6 (CH), 130.2 (CH), 129.8 (C), 128.8 (CH), 127.8 (CH), 60.6 (CH), 58.8 (CH), 45.8 (CH_2), 30.0 (CH_2), 23.5 (CH_2). IR (ATR, cm-1): 2976 (C-H st), 1645 (N-C=O st), 746 (C-Cl st). MS (EI) m/z (%): 185 (M^+-HCl, 75), 184 (100), 182 (14), 128 (10).

General procedure I for the Suzuki coupling of 2,3-dihydropyrrolo[1,2-*b*]isoquinolin-5(1*H*)-one derivatives (9a-d). An oven-dried 50 mL two-necked flask was charged with the corresponding 10-iodo-2,3-dihydropyrrolo[1,2-*b*]isoquinolin-5(1*H*)-one **7a-e** (31.2 mg, 0.1 mmol), phenyl bornic acid (15.9 mg, 0.13 mmol), $Pd(PPh_3)_4$ (5.7 mg, 0.005 mmol), and K_2CO_3 (69.1 mg, 0.5 mmol). Under Argon atmosphere, 1,4-dioxane (5 mL) and water (2 mL) were added, and the reaction mixture was subjected to vacuum and refilled with Argon three times. The reaction was heated in a heating plate at 65ºC for 21h. After completion, the mixture was cooled to room temperature and diluted with EtOAc. The mixture was filtered through a small pad of Celite®. Afterwards the solvent was concentrated in vacuum. The resulting crude was purified by flash chromatography (PE/EtOAc 7:3 to 1:1).

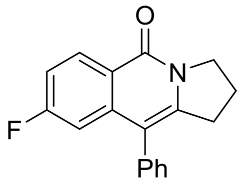

8-Fluoro-10-phenyl-2,3-dihydropyrrolo[1,2-*b*]isoquinolin-5(1*H*)-one (9b). Following the general procedure I for the Suzuki coupling, **9b** was obtained as a yellow solid (23.4 mg, 0.0895 mmol, 90%) by FC (PE/EtOAc 3:7) starting from 8-fluoro-10-iodo-2,3-dihydropyrrolo[1,2-*b*]isoquinolin-5(1*H*)-one **7b** (32.91 mg, 0.1 mmol), phenylboronic acid (16.7 mg, 0.13 mmol), $Pd(PPh_3)_4$ (5.8 mg, 0.005 mmol, 5 mol%), K_2CO_3 (69.1 mg, 0.5 mmol), in a mixture of 1,4-dioxane (5 mL) and water (2 mL). R_f = 0.35 (PE/EtOAc 3:7). mp: 127-136 °C. ^{1}H-NMR (300 MHz, $CDCl_3$): δ 8.48 (dd, *J* = 8.9, 6.0 Hz, 1H), 7.53 – 7.41 (m, 3H), 7.32 – 7.23 (m, 2H), 7.12 (td, *J* = 8.5, 2.5 Hz, 1H), 6.89 (dd, *J* = 10.6, 2.5 Hz, 1H), 4.27 (t, *J* = 7.1 Hz, 2H), 2.94 (t, *J* = 7.6 Hz, 2H), 2.16 (p, *J* = 7.5 Hz, 2H). ^{13}C {^{1}H} NMR (75 MHz, $CDCl_3$): δ 167.0 (C), 162.1 (d, *J* = 235.3 Hz, C), 143.0 (C), 140.7 (d, *J* = 9.9 Hz, C), 135.9 (C), 130.7 (d, *J* = 9.9 Hz, CH), 130.6 (2 × CH), 129.0 (2 × CH),

127.9 (CH), 121.8 (d, J = 1.7 Hz, C), 114.4 (d, J = 23.7 Hz, CH), 113.4 (d, J = 3.5 Hz, C), 109.5 (d, J = 23.1 Hz, CH), 48.7 (CH_2), 31.3 (CH_2), 22.0 (CH_2). ^{19}F NMR (282 MHz, $CDCl_3$) δ -106.71. IR (ATR, cm^{-1}): 2969 (C-H st), 1738 (C=O st), 1654 (C_{Arom}-C_{Arom} st) 1610 (C_{Arom}-C_{Arom} st). MS (EI) m/z (%): 280.0 (36), 279.1 (M^+ 100), 277.0 (11). HRMS (ESI) m/z: $[M+H]^+$ Calcd for $[C_{18}H_{15}FNO]^+$ 280.1132; Found 280.1144 for compound: **9b.**

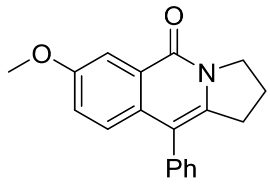

7-Methoxy-10-phenyl-2,3-dihydropyrrolo[1,2-*b*]isoquinolin-5(1*H*)-one (9c). Following the general procedure I for the Suzuki coupling, **9c** was obtained as a yellow solid (23.4 mg, 0.0283 mmol, 64%) by FC (CH_2Cl_2/EtOAc 8:2) starting from 10-iodo-7-methoxy-2,3-dihydropyrrolo[1,2-*b*]isoquinolin-5(1*H*)-one **7d** (15.1 mg, 0.044 mmol), phenylboronic acid (7.4 mg, 0.0576 mmol), $Pd(PPh_3)_4$ (2.5 mg, 0.00215 mmol, 5 mol%), K_2CO_3 (30.6 mg, 0.222 mmol) in a mixture of 1,4-dioxane (2 mL) and water (1 mL). R_f = 0.28 (CH_2Cl_2/EtOAc 8:2). mp: 105 – 114 °C. ^{1}H-NMR (300 MHz, $CDCl_3$): δ 7.89 (d, J = 2.7 Hz, 1H), 7.52 – 7.36 (m, 3H), 7.33 – 7.27 (m, 2H), 7.22 (d, J = 8.9 Hz, 1H), 7.14 (dd, J = 8.9, 2.7 Hz, 1H), 4.29 (t, J = 7.2 Hz, 2H), 3.93 (s, 3H), 2.93 (t, J = 7.6 Hz, 2H), 2.15 (p, J = 7.5 Hz, 2H). ^{13}C {^{1}H} NMR (75 MHz, $CDCl_3$): δ 160.8 (C), 158.1 (C), 139.0 (C), 136.6 (C), 132.4 (C), 130.7 (2 × CH), 128.8 (2 × CH), 127.6 (CH), 126.3 (C), 126.1 (CH), 122.6 (CH), 113.9 (C), 107.3 (CH), 55.8 (CH_3), 48.8 (CH_2), 30.9 (CH_2), 22.2 (CH_2). IR (ATR, cm^{-1}): 2998 (C-H st), 1738 (C=O st), 1611 (C_{Arom}-C_{Arom} st), 1216 (C_{Arom}-O-C st as). MS (EI) m/z (%): 292.1 (45), 291.0 (M^+, 100), 191.0 (24). HRMS (ESI) m/z: $[M+H]^+$ Calcd for $[C_{19}H_{18}NO_2]^+$ 292.1332; Found 292.1344 for compound: **9c.**

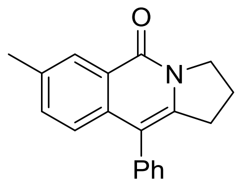

7-Methyl-10-phenyl-2,3-dihydropyrrolo[1,2-*b*]isoquinolin-5(1*H*)-one (9d). Following the general procedure I for the Suzuki coupling, **9d** was obtained as a yellow solid (19.2 mg, 0.0697 mmol, 70%) by FC (CH_2Cl_2/EtOAc 7:3 to 1:1) starting from 10-iodo-7-methyl-2,3-dihydropyrrolo[1,2-*b*]isoquinolin-5(1*H*)-one **7e** (33.4 mg, 0.10 mmol), phenylboronic acid (10.7 mg, 0.13 mmol), $Pd(PPh_3)_4$ (5.8 mg, 0.005 mmol, 5 mol%), K_2CO_3 (69.1 mg, 0.5 mmol) in a mixture of 1,4-dioxane (5 mL) and water (2 mL). R_f = 0.32 (PE/EtOAc 1:1). mp: 113-115 °C. ^{1}H-NMR (300 MHz, $CDCl_3$): δ 8.30 (d, *J* = 1.0 Hz, 1H), 7.52 – 7.28 (m, 6H), 7.20 (d, *J* = 8.3 Hz, 1H), 4.28 (t, *J* = 7.2 Hz, 2H), 2.94 (t, *J* = 7.6 Hz, 2H), 2.48 (s, 3H, CH_3), 2.16 (p, *J* = 7.5 Hz, 2H). ^{13}C {^{1}H} NMR (75 MHz, $CDCl_3$): δ 161.2 (C), 140.4 (C), 136.6 (C), 135.9 (C), 135.7 (C), 133.5 (CH), 130.7 (2 × CH), 128.8 (2 × CH), 127.6 (CH), 127.1 (CH), 125.0 (C), 124.4 (CH), 113.8 (C), 48.6 (CH_2), 31.0 (CH_2), 22.1 (CH_2), 21.4 (CH_3). IR (ATR, cm^{-1}): 1650 (C=O st), 1611 (C_{Arom}-C_{Arom} st). MS (EI) m/z (%): 274.1 (11), 275.1 (M^+, 100), 276.1 (33). HRMS (ESI) m/z: $[M+H]^+$ Calcd for $[C_{19}H_{18}NO]^+$ 276.1388; Found 276.1389 for compound: **9d.**

General procedure J for the synthesis of *tert*-butyl (2-iodobenzyl)(2-vinylbenzoyl)carbamate (10a-h). In a first step, an oven-dried 100 mL two-necked flask provided with the corresponding methyl 2-vinylbenzoate (7.40 mmol) and the corresponding (2-iodophenyl)methanamine (7.40 mmol) under Ar atmosphere, LiHMDS in THF 1 M (22.2 mL, 22.2 mmol) was added dropwise at room temperature to the stirring mixture and it was let stirring 16 h at this temperature. MeOH (22 mL) was added and the crude mixture was concentrated under vacuum and was then diluted with CH_2Cl_2 and filtrated by Celite®. The filtrate was concentrated under vacuum and purified by silica gel column chromatography to obtain the corresponding *N*-(2-iodobenzyl)-2-vinylbenzylamide (1.92 g, 6.08 mmol). In a second step, an oven-dried 50 mL two-necked flask provided with the corresponding *N*-(2-iodobenzyl)-2-vinylbenzamide (6.08 mmol) and di-*tert*-butyl dicarbonate (2.00 g, 9.12 mmol) under Ar atmosphere were dissolved in dry CH_2Cl_2 (12 mL) and under stirring DMAP (74 mg, 0.60 mmol) was added at room temperature. The reaction mixture was let stirring at rt for 16 h. Then, it was concentrated under vacuum and purified by silica gel column chromatography. Note: methyl 2-vinylbenzoate,[1] methyl 5-fluoro-2-vinylbenzoate,[1] methyl 4-fluoro-2-vinylbenzoate,[2] methyl 5-chloro-2-vinylbenzoate,[1] methyl 5-methyl-2-vinylbenzoate,[3] methyl 5-methoxy-2-vinylbenzoate,[3] (2-iodophenyl)methanamine,[4] (4-fluoro-2-iodophenyl)methanamine,[5] (2-iodo-5-methylphenyl)methanamine[6] were prepared following procedures previously described in the literature.

---

1 Möckel, R.; Babaoglu, E.; Hilt, G. *Chem. Eur. J.* **2018**, *24*, 15781–15785
2 Cai, Z.; Guo, Y.; Liu, F.; Liu, Y.; Tang, X.; Xin, X. CN111499514A , 2020
3 Dydio, P.; Reek, J. N. H. *Angew. Chem. Int. Ed.* **2013**, *52*, 3878-3882
4 Ren, W.; Yamane, M. *J. Org. Chem.* **2010**, *75*, 8410–8415
5 Naidu, B.; Ueda, Y.; Matiskella, J.; Walker, M.; Banville, J.; Beaulieu, F.; Ouellet, C.; Plamondon, S. US20070111984, 2007
6 Okuro, K.; Alper, H. *Synlett* **2012**, *23*, 2531-2533

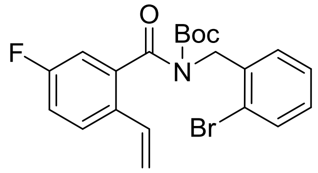

***tert*-Butyl (2-bromobenzyl)(5-fluoro-2-vinylbenzoyl)carbamate (10b).** Following the first step of the general procedure J, *N*-(2-bromobenzyl)-5-fluoro-2-vinylbenzamide was obtained as a white solid (1.98 g, 5.19 mmol, 73%) by FC (PE/EtOAc 8:2 to 7:3) starting from methyl 5-fluoro-2-vinylbenzoate (1.28 g, 7.08 mmol), (2-bromophenyl)methanamine (1.65 g, 7.0 mmol) and LiHMDS 1 M in THF (21.3 mL, 21.3 mmol). R_f = 0.5 (PE/EtOAc 7:3). mp: 97-98 ºC. ^{1}H NMR (300 MHz, $CDCl_3$) δ 7.60 (dd, *J* = 7.9, 1.3 Hz, 1H), 7.53 (dd, *J* = 8.1, 4.8 Hz, 1H), 7.51 – 7.45 (m, 1H), 7.33 (td, *J* = 7.5, 1.3 Hz, 1H), 7.24 – 7.19 (m, 2H), 7.11 (td, *J* = 8.5, 2.9 Hz, 1H), 6.95 (dd, *J* = 17.4, 10.9 Hz, 1H), 6.34 (bs, 1H), 5.64 (dd, *J* = 17.4, 1.0 Hz, 1H), 5.32 (dt, *J* = 11.0, 0.8 Hz, 1H), 4.70 (d, *J* = 6.0 Hz, 2H). ^{13}C {^{1}H} NMR (75 MHz, $CDCl_3$) δ 167.7 (d, *J* = 2.2 Hz, C), 161.9 (d, *J* = 249.2 Hz, C), 136.8 (C), 136.4 (d, *J* = 6.6 Hz, C), 133.5 (CH), 132.9 (CH), 132.3 (d, *J* = 3.7 Hz, C), 130.8 (CH), 129.5 (CH), 128.5 (d, *J* = 7.7 Hz, CH), 127.9 (CH), 123.9 (C), 117.5 (d, *J* = 21.3 Hz, CH), 117.0 (d, *J* = 1.9 Hz, CH_2), 114.5 (d, *J* = 23.0 Hz, CH), 44.5 (CH_2). ^{19}F NMR (282 MHz, $CDCl_3$) δ -113.40. IR (ATR, cm^{-1}): 3274 (N-H), 1640 (N-C=O), 1605 (C=C). MS (EI) m/z (%): 254.1 (M^+-I, 72), 164.0 (M^+-$CH_3C_6H_4I$, 100). HRMS (ESI) m/z: $[M+H]^+$ Calcd for $[C_{16}H_{14}BrFNO]^+$ 334.0237; Found 334.0243 Following the second step of general procedure J, **10b** was obtained as a yellow oil (2.30 g, 4.78 mmol, 93%) (overall yield for the two steps = 68%) by FC (PE/EtOAc 95:5) starting from *N*-(2-bromobenzyl)-5-fluoro-2-vinylbenzamide (1.98 g, 5.12 mmol), di-*tert*-butyl carbonate (1.70 g, 7.79 mmol), DMAP (61 mg, 0.50 mmol) in CH_2Cl_2 (10 mL). R_f = 0.67 (PE/EtOAc 9:1). ^{1}H NMR (300 MHz, $CDCl_3$) δ 7.60 (dd, *J* = 7.9, 1.2 Hz, 1H), 7.56 (dd, *J* = 8.6, 5.3 Hz, 1H), 7.34 (td, *J* = 7.5, 1.3 Hz, 1H), 7.23 (dd, *J* = 7.9, 1.2 Hz,

1H), 7.18 (dd, J = 7.9, 1.8 Hz, 1H), 7.15 – 7.08 (m, 1H), 7.05 (dd, J = 8.6, 2.7 Hz, 1H), 6.81 (dd, J = 17.4, 11.0 Hz, 1H), 5.68 (dd, J = 17.4, 0.7 Hz, 1H), 5.37 (dd, J = 11.0, 0.8 Hz, 1H), 5.15 (s, 2H), 1.15 (s, 9H). ^{13}C {^{1}H} NMR (75 MHz, $CDCl_3$) δ 170.7 (d, J = 2.3 Hz, C), 161.7 (d, J = 248.7 Hz, C), 152.0 (C), 138.5 (d, J = 7.2 Hz, C), 136.3 (C), 132.9 (CH), 132.7 (CH), 131.4 (d, J = 3.8 Hz, C), 128.7 (CH), 127.7 (d, J = 8.0 Hz, CH), 127.5 (CH), 127.2 (CH), 122.8 (C), 116.9 (d, J = 3.0 Hz, CH_2), 116.7 (d, J = 23.14 Hz, CH), 113.3 (d, J = 23.4 Hz, CH), 84.1 (C) , 48.3 (CH_2), 27.34 (3 × CH_3). ^{19}F NMR (282 MHz, $CDCl_3$) δ -114.21. IR (ATR, cm^{-1}): 1737 (C=O), 1673 (N-C=O). MS (EI) m/z (%): 254.1 (M^+ - Boc-I, 84). HRMS (ESI) m/z: $[M+Na]^+$ Calcd for $[C_{21}H_{21}BrFNNaO_3]^+$ 456.0581; Found 456.0585 for compound: **10b**.

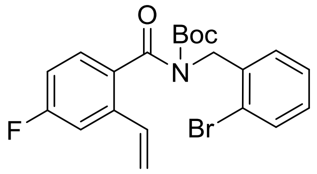

***tert*-Butyl (2-bromobenzyl)(4-fluoro-2-vinylbenzoyl)carbamate (10c).** Following the first step of the general procedure J, *N*-(2-bromobenzyl)-4-fluoro-2-vinylbenzamide was obtained as a white solid (2.31 g, 6.07 mmol, 71%) by FC (PE/EtOAc 9:1 to 8:2) starting from methyl 4-fluoro-2-vinylbenzoate (1.53 g, 8.50 mmol), (2-bromophenyl)methanamine (1.98 g, 8.50 mmol) and LiHMDS 1 M in THF (25.5 mL, 25.5 mmol). R_f = 0.31 (PE/EtOAc 9:1). mp: 95-98 ºC. ^{1}H NMR (300 MHz, $CDCl_3$) δ 7.55 (d, J = 7.9 Hz, 1H), 7.38 (td, J = 5.4, 2.6 Hz, 2H), 7.27 (td, J = 7.4, 1.8 Hz, 1H), 7.22 – 7.10 (m, 2H), 6.97 (dd, J = 17.4, 11.0 Hz, 1H), 6.1-6.87 (m, 1H), 6.73 (bs, 1H), 5.65 (dd, J = 17.4, 2.1 Hz, 1H), 5.33 (dd, J = 11.0, 2.3 Hz, 1H), 4.67 – 4.57 (m, 2H). ^{13}C {^{1}H} NMR (75 MHz, $CDCl_3$) δ 168.3 (d, J = 2.1 Hz, C), 163.6 (d, J = 249.6 Hz, C), 138.8 (d, J = 8.0 Hz, C), 137.0 (C), 133.4 (d, J = 2.1 Hz, CH), 132.8 (CH), 131.0 (d, J = 2.9 Hz, C), 130.2 (CH), 129.7 (d, J = 8.8 Hz, CH), 129.3 (CH), 127.7 (CH), 123.7 (C), 117.8 (d,

J = 2.9 Hz, CH_2), 114.6 (d, J = 22.0 Hz, CH), 112.9 (dd, J = 22.2, 2.5 Hz, CH), 44.4 (CH_2). ^{19}F NMR (282 MHz, $CDCl_3$) δ -109.98. IR (ATR, cm^{-1}): 3274 (N-H), 1640 (N-C=O), 1605 (C=C). MS (EI) m/z (%): 254.1 (M^+-I, 100). HRMS (ESI) m/z: $[M+H]^+$ Calcd for $[C_{16}H_{14}BrFNO]^+$ 334.0237; Found 334.0250. Following the second step of general procedure J, **10c** was obtained as a white solid (2.42 g, 5.02 mmol, 83%) (overall yield for the two steps = 59%) by FC (PE/EtOAc 95:5) starting from *N*-(2-bromobenzyl)-4-fluoro-2-vinylbenzamide (2.32 g, 6.07 mmol), di-*tert*-butyl carbonate (1.99 g, 9.11 mmol), DMAP (74 mg, 0.60 mmol) in CH_2Cl_2 (13 mL). R_f = 0.56 (PE/EtOAc 9:1). mp: 92-93 ºC. ^{1}H NMR (300 MHz, $CDCl_3$) δ 7.59 (dd, J = 7.9, 1.2 Hz, 1H), 7.35 (dd, J =8.4, 5.4 Hz 1H), 7.32 – 7.21 (m, 3H), 7.14 (td, J = 7.1, 2.0 Hz, 1H), 7.02 (td, J = 8.3, 2.6 Hz, 1H), 6.90 (ddd, J = 17.4, 11.0, 1.5 Hz, 1H), 5.77 (dd, J = 17.4, 0.8 Hz, 1H), 5.44 (dd, J = 11.0, 0.8 Hz, 1H), 5.16 (s, 2H), 1.15 (s, 9H). ^{13}C {^{1}H} NMR (75 MHz, $CDCl_3$) δ 171.3 (C) , 163.4 (d, J = 249.3 Hz, C), 152.3 (C), 138.1 (d, J = 7.9 Hz, C), 136.5 (C), 133.2 (d, J = 3.3 Hz, C), 132.9 (CH), 132.9 (CH), 128.7 (CH), 128.6 (d, J = 8.8 Hz, CH), 127.5 (CH), 127.3 (CH), 122.8 (C), 118.1 (CH_2), 114.4 (d, J = 22.1 Hz, CH), 112.5 (d, J = 22.4 Hz, CH), 83.8 (C), 48.5 (CH_2), 27.4 (3 × CH_3). ^{19}F NMR (282 MHz, $CDCl_3$) δ -110.55. IR (ATR, cm^{-1}): 1737 (C=O), 1674 (N-C=O). MS (EI) m/z (%): 254 (M^+ - Boc - I, 54). HRMS (ESI) m/z: $[M+Na]^+$ Calcd for $[C_{21}H_{21}BrFNNaO_3]^+$ 456.0581; Found 456.0589 for compound: **10c**.

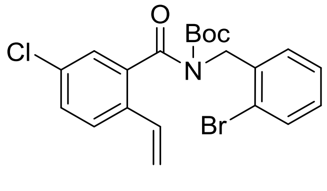

***tert*-Butyl (2-bromobenzyl)(5-chloro-2-vinylbenzoyl) carbamate (10d).** Following the first step of the general procedure J, *N*-(2-bromobenzyl)-5-chloro-2-vinylbenzamide was obtained as a white solid (2.58 g, 6.49 mmol, 66%) by FC (PE/EtOAc 9:1 to 7:3) starting

from methyl 5-chloro-2-vinylbenzoate (1.94 g, 9.80 mmol), (2-bromophenyl)methanamine (2.28 g, 9.80 mmol) and LiHMDS 1 M in THF (29.4 mL, 29.4 mmol). R_f = 0.21 (PE/EtOAc 9:1). mp: 117-118 ºC. ^{1}H NMR (300 MHz, $CDCl_3$) δ 7.54 (d, *J* = 7.9 Hz, 1H), 7.42 (d, *J* = 8.4 Hz, 1H), 7.39 – 7.33 (m, 2H), 7.31 – 7.24 (m, 2H), 7.15 (t, *J* = 7.7 Hz, 1H), 6.88 (ddd, *J* = 17.6, 11.0, 2.0 Hz, 1H), 6.77 (bs, 1H), 5.63 (dt, *J* = 17.4, 1.1 Hz, 1H), 5.29 (dt, *J* = 11.0, 1.1 Hz, 1H), 4.61 – 4.55 (m, 2H). ^{13}C {^{1}H} NMR (75 MHz, $CDCl_3$) δ 167.9 (C), 136.8 (C), 136.2 (C), 134.4 (C), 133.3 (CH), 130.2 (CH), 130.1 (C), 132.9 (CH), 130.2 (CH), 129.3 (CH), 127.7 (CH), 127.6 (CH), 127.4 (CH), 123.7 (C), 117.3 (CH_2), 44.4 (CH_2). IR (ATR, cm^{-1}): 3257 (N-H), 1639 (N-C=O), 1535 (C=C). MS (EI) m/z (%): 270.1 (M^+-I, 70). HRMS (ESI) m/z: $[M+H]^+$ Calcd for $[C_{16}H_{14}BrClNO]^+$ 349.9942; Found 349.9947. Following the second step of general procedure J, **10d** was obtained as a yellow oil (2.50 g, 5.02 mmol, 77%) (overall yield for the two steps = 51%) by FC (PE/EtOAc 95:5) starting from *N*-(2-bromobenzyl)-5-chloro-2-vinylbenzamide (2.58 g, 6.49 mmol), di-*tert*-butyl carbonate (2.13 g, 9.74 mmol), DMAP (79 mg, 0.65 mmol) in CH_2Cl_2 (13 mL). R_f = 056 (PE/EtOAc 9:1). ^{1}H NMR (300 MHz, $CDCl_3$) δ 7.58 (dd, *J* = 8.0, 1.3 Hz, 1H), 7.51 (d, *J* = 8.4 Hz, 1H), 7.40 – 7.28 (m, 3H), 7.21 (dd, *J* = 7.8, 1.9 Hz, 1H), 7.14 (ddd, *J* = 8.5, 7.9, 1.7 Hz, 1H), 6.79 (dd, *J* = 17.4, 11.0 Hz, 1H), 5.72 (dd, *J* = 17.4, 0.9 Hz, 1H), 5.38 (dd, *J* = 11.0, 0.9 Hz, 1H), 5.13 (s, 2H), 1.13 (s, 9H). ^{13}C {^{1}H} NMR (75 MHz, $CDCl_3$) δ 170.6 (C), 152.0 (C), 138.3 (C), 136.2 (C), 133.6 (C), 133.2 (C), 132.9 (CH), 132.6 (CH), 129.6 (CH), 128.7 (CH), 127.5 (CH), 127.2 (CH), 127.1 (CH), 126.3 (CH), 122.8 (C), 117.6 (CH_2), 84.1 (C), 48.3 (CH_2), 27.3 (3 × CH_3). IR (ATR, cm^{-1}): 1721 (C=O), 1680 (N-C=O). MS (EI) m/z (%): 269.8 (M^+ - Boc - I, 11). HRMS (ESI) m/z: $[M+Na]^+$ Calcd for $[C_{21}H_{21}BrClNNaO_3]^+$ 472.0286; Found 472.0288 for compound: **10d**.

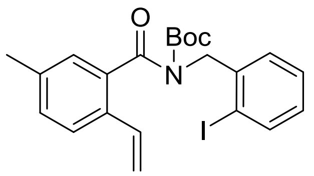

***tert*-Butyl (2-iodobenzyl)(5-methyl-2-vinylbenzoyl)carbamate (10e)**. Following the first step of the general procedure J, *N*-(2-iodobenzyl)-5-methyl-2-vinylbenzamide was obtained as a white solid (2.70 g, 7.16 mmol, 75%) by FC (PE/EtOAc 8:2 to PE/EtOAc 7:3) starting from methyl 5-methyl-2-vinylbenzoate (1.69 g, 9.57 mmol), (2-iodophenyl)methanamine (2.34 g, 10.05 mmol) and LiHMDS in THF 1 M (28,7 mL, 28,7 mmol). R_f = 0.54 (PE/EtOAc 8:2). mp: 138 - 139 °C. ^{1}H NMR (300 MHz, $CDCl_3$): δ 7.84 (dd, *J* = 7.9, 1.2 Hz, 1H), 7.46 (dd, *J* = 7.5, 1.8 Hz, 1H), 7.44 (d, *J* = 7.7 Hz, 1H), 7.34 (td, *J* = 7.5, 1.3 Hz, 1H), 7.32 – 7.29 (m, 1H), 7.23 – 7.17 (m, 1H), 7.06 – 6.93 (m, 2H), 6.27 (br t, *J* = 6.1 Hz, 1H), 5.64 (dd, *J* = 17.5, 1.2 Hz, 1H), 5.27 (dd, *J* = 11.0, 1.2 Hz, 1H), 4.65 (d, *J* = 6.0 Hz, 2H), 2.34 (s, 3H). ^{13}C {^{1}H} NMR (75 MHz, $CDCl_3$) δ 169.3 (C), 140.4 (C), 139.6 (CH), 137.8 (C), 134.9 (C), 134.5 (CH), 133.4 (C), 131.2 (CH), 130.2 (CH), 129.6 (CH), 128.8 (CH), 128.2 (CH), 126.5 (CH), 116.2 (CH_2), 99.3 (C), 48.8 (CH_2), 21.2 (CH_3). IR (ATR, cm^{-1}): 3260 (N-H st), 1633 (C=O st), 1535 (N-H δ). MS (EI) m/z (%): 251.20 (14), 250.1 (M^+ - I, 15), 216.9 (benzylic cleavage, benzylic fragment, 59), 160.0 (benzylic cleavage, amide fragment, 72), 126.9 (I^+, 60), 115.0 (100), 91.0 (52). HRMS (ESI) m/z: $[M+H]^+$ Calcd for $[C_{17}H_{17}INO]^+$ 378.0349; Found 378.0356. Following the second step of general procedure J, **10e** was obtained as a white solid (2.94 g, 6.15 mmol, 89%) (overall yield for the two steps = 67%) by FC (PE/EtOAc 99:01 to PE/EtOAc 90:10) starting from *N*-(2-iodobenzyl)-5-methyl-2-vinylbenzamide (2.63 g, 6.93 mmol), di-*tert*-butyl carbonate (2.26 g, 10.40 mmol), DMAP (84.7 mg, 0.69 mmol) in CH_2Cl_2 (14 mL). R_f = 0.51 (PE/EtOAc 9:1). mp: 80 °C – 83 °C. ^{1}H NMR (300 MHz, $CDCl_3$): δ 7.86 (dd, *J* = 7.9, 1.2 Hz, 1H), 7.48 (d, *J* = 8.0 Hz, 1H), 7.35 (td, *J* = 7.5, 1.3

Hz, 1H), 7.24 – 7.12 (m, 3H), 6.97 (td, *J* = 7.8, 1.7 Hz, 1H), 6.85 (dd, *J* = 17.4, 11.0 Hz, 1H) , 5.69 (dd, *J* = 17.4, 1.1 Hz, 1H), 5.31 (dd, *J* = 11.0, 1.1 Hz, 1H), 5.04 (s, 2H), 2.36 (s, 3H), 1.09 (s, 9H). ^{13}C {^{1}H} NMR (75 MHz, $CDCl_3$) :δ 172.4 (C), 152.5 (C), 139.6 (CH), 139.5 (C), 137.4 (C), 137.0 (C), 133.7 (CH), 132.7 (C), 130.6 (CH), 128.8 (CH), 128.5 (CH), 127.0 (CH), 126.3 (CH), 125.8 (CH), 116.1 (CH_2), 97.8 (C), 83.7 (C), 53.5 (CH_2), 27.4 (3 × CH_3), 21.2 (CH_3). IR (ATR, cm^{-1}): 1733 (C=$O_{carbamate}$ st), 1671 (C=O_{amide} st). MS (EI) m/z (%): 377.1 (MH^+ - Boc, 9). HRMS (ESI) m/z: $[M+Na]^+$ Calcd for $[C_{22}H_{24}INNaO_3]^+$ 500.0693; Found 500.0701 for compound: **10e**.

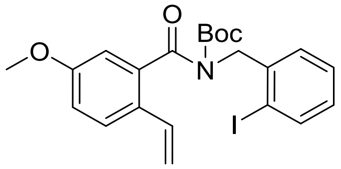

***tert*-Butyl (2-iodobenzyl)(5-methoxy-2-vinylbenzoyl)carbamate (10f).** Following the first step of the general procedure J, *N*-(2-iodobenzyl)-5-methoxy-2-vinylbenzamide was obtained as a yellow solid (5.13 g, 8.87 mmol, 67%) by FC (PE/EtOAc 95:05 to 1:1) starting from methyl 5-methoxy-2-vinylbenzoate (3.74 g, 19.49 mmol), (2-iodophenyl)methanamine (4.89 g, 20.98 mmol) and LiHMDS 1 M in THF (58.46 mL, 58.46 mmol). R_f = 0.44 (PE/EtOAc 8:2). mp: 122-124 °C. ^{1}H-NMR (300 MHz, $CDCl_3$): δ 7.70 (dd, *J* = 7.9, 1.2 Hz, 1H), 7.33 (d, *J* = 8.5 Hz, 2H), 7.24 – 7.15 (m, 1H), 6.92 – 6.73 (m, 4H), 6.11 (br t, *J* = 5.8 Hz, 1H), 5.43 (dd, *J* = 17.5, 1.2 Hz, 1H), 5.08 (dd, *J* = 11.0, 1.2 Hz, 1H), 4.51 (d, *J* = 5.9 Hz, 2H), 3.67 (s, 3H). ^{13}C {^{1}H} NMR (75 MHz, $CDCl_3$): δ 168.9 (C), 159.2 (C), 140.3 (C), 139.7 (CH), 136.1 (C), 134.1 (CH), 130.3 (CH), 129.6 (CH), 128.8 (CH), 128.7 (C), 128.0 (CH), 116.7 (CH), 115.4 (CH_2), 112.5 (CH), 99.3 (C), 55.6 (CH_3), 48.9 (CH_2). IR (ATR, cm^{-1}): 3264 (N-H st), 1642 (C=O st), 1517 (C=C st). MS (EI) m/z (%): 393.0 (M^+, 59), 392.0 (100), 391.0 (48). HRMS (ESI) m/z: $[M+H]^+$ Calcd for $[C_{17}H_{17}INO_2]^+$ 394.0298; Found 394.0304. Following the second step of

general procedure J, **10f** was obtained as a white solid (4.38 g, 8.89 mmol, 92%) (overall yield for the two steps = 62%) by FC (PE/EtOAc 95:05) starting from *N*-(2-iodobenzyl)-5-methoxy-2-vinylbenzamide (3.80 g, 9.66 mmol), di-*tert*-butyl dicarbonate (3.16 g, 14.49 mmol), DMAP (118 mg, 0.96 mmol) in CH_2Cl_2 (19 mL). R_f = 0.52 (PE/EtOAc 95:05). mp: 112-114 °C. ^{1}H-NMR (300 MHz, $CDCl_3$): δ 7.86 (dd, *J* = 7.9, 1.3 Hz, 1H), 7.51 (d, *J* = 8.7 Hz, 1H), 7.35 (td, *J* = 7.5, 1.2 Hz, 1H), 7.20 – 7.14 (m, 1H), 7.01 – 6.91 (m, 2H), 6.86 – 6.74 (m, 2H), 5.61 (d, *J* = 17.4 Hz, 1H), 5.26 (d, *J* = 11.1 Hz, 1H), 5.04 (s, 2H), 3.82 (s, 3H), 1.11 (s, 9H). ^{13}C {^{1}H} NMR (75 MHz, $CDCl_3$): δ 171.9 (C), 159.0 (C), 152.4 (C), 139.7 (CH), 139.4 (C), 138.1 (C), 133.2 (CH), 128.9 (CH), 128.5 (CH), 128.1 (C), 127.2 (CH), 126.4 (CH), 116.1 (CH), 115.1 (CH_2), 111.4 (CH), 97.9 (C), 83.9 (C), 55.7 (CH_3), 53.5 (CH_2), 27.5 (3 × CH_3). IR (ATR, cm^{-1}): 1732 ($C=O_{carbamate}$ st), 1671 ($C=O_{amide}$ st). MS (EI) m/z (%): 392.9 (80), 392.0 (M^+- Boc, 100), 391.0 (44). HRMS (ESI) m/z: $[M+Na]^+$ Calcd for $[C_{22}H_{24}INNaO_4]^+$ 516.0642; Found 516.0645 for compound: **10f**.

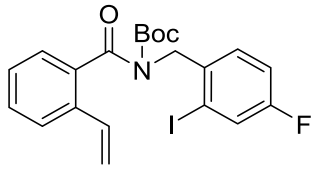

***tert*-Butyl (4-fluoro-2-iodobenzyl)(2-vinylbenzoyl)carbamate (10g).** Following the first step of the general procedure J, *N*-(4-fluoro-2-iodobenzyl)-2-vinylbenzamide was obtained as a white solid (2.24 g, 5.88 mmol, 64%) by FC (PE/EtOAc 8:2 to PE/EtOAc 7:3) starting from methyl 2-vinylbenzoate (1.7 g, 9.25 mmol), (4-fluoro-2-iodophenyl)methanamine (2.32 g, 9.25 mmol) and LiHMDS 1 M in THF (28 mL, 28 mmol). R_f= 0.47 (PE/EtOAc 8:2). mp: 110-112 ºC. ^{1}H NMR (300 MHz, $CDCl_3$) δ 7.64 – 7.49 (m, 2H), 7.54 – 7.47 (m, 2H), 7.43 (td, *J* = 8.2, 1.9 Hz, 1H), 7.32 (td, *J* = 7.5, 1.3 Hz, 1H), 7.09 (td, *J* = 8.5, 2.7 Hz, 1H), 7.06 – 6.97 (m, 1H), 6.29 (bs, 1H) 5.71 (dd, *J* =

17.4, 1.2 Hz, 1H), 5.36 (dd, *J* = 10.9, 1.2 Hz, 1H), 4.65 (d, *J* = 6.0 Hz, 2H). $^{13}C\ \{^{1}H\}$ NMR (75 MHz, $CDCl_3$) δ 169.1 (C), 161.5 (d, *J* = 252.0 Hz, C), 136.4 (d, *J* = 3.5 Hz, C), 136.1 (C), 134.7 (C), 134.5 (CH), 131.0 (d, *J* = 8.2 Hz, CH), 130.5 (CH), 127.7 (d, *J* = 20.2 Hz, CH), 126.6 (CH), 126.5 (CH), 126.3 (CH), 117.1 (CH_2), 115.7 (d, *J* = 20.9 Hz, CH), 98.3 (d, *J* = 8.2 Hz, C), 47.9 (CH_2). ^{19}F NMR (282 MHz, $CDCl_3$) δ -114.24. IR (ATR, cm^{-1}): 3374 (N-H), 1640 (N-C=O), 1593 (C=C). MS (EI) m/z (%): 234.9 (M^+-I-F, 83), 146.0 (M^+-$CH_3C_6H_3IF$, 77). HRMS (ESI) m/z: $[M+H]^+$ Calcd for $[C_{16}H_{14}FINO]^+$ 382.0099; Found 382.0105. Following the second step of general procedure J, **10g** was obtained as a white solid (2.41 g, 5.01 mmol, 85%) (overall yield for the two steps = 55%) by FC (PE/EtOAc 9:1 to 8:2) starting from *N*-(4-fluoro-2-iodobenzyl)-2-vinylbenzamide (2.24 g, 5.88 mmol), di-*tert*-butyl carbonate (1.93 g, 8.83 mmol), DMAP (72 mg, 0.59 mmol) in CH_2Cl_2 (12 mL). R_f = 0.38 (PE/EtOAc 9:1). mp: 135-138 ºC. ^{1}H NMR (300 MHz, $CDCl_3$) δ 7.65 – 7.57 (m, 2H), 7.43 (td, *J* = 8.0, 4.0 Hz, 1H), 7.32 (d, *J* = 4.0 Hz, 2H), 7.20 (dd, *J* = 8.6, 5.8 Hz, 1H), 7.11 (td, *J* = 8.4, 2.5 Hz, 1H), 6.89 (dd, *J* = 17.4, 11.0 Hz, 1H), 5.76 (d, *J* = 17.4 Hz, 1H), 5.39 (d, *J* = 11.0 Hz, 1H), 5.04 (s, 2H), 1.11 (s, 9H). $^{13}C\ \{^{1}H\}$ NMR (75 MHz, $CDCl_3$) δ 172.1 (C), 161.1 (d, *J* = 250.6 Hz, C), 152.3 (C), 137.0 (C), 135.4 (d, *J* = 3.3 Hz, C), 135.3 (C), 133.7 (CH), 129.8 (CH), 127.4 (CH), 127.3 (d, *J* = 8.2 Hz, CH), 126.4 (d, *J* = 23.7 Hz, CH), 126.3 (CH), 125.9 (CH), 117.1 (CH_2), 115.4 (d, *J* = 21.1 Hz, CH), 96.9 (d, *J* = 8.1 Hz, C), 83.9 (C), 52.5 (CH_2), 27.3 (3 × CH_3). ^{19}F NMR (282 MHz, $CDCl_3$) δ -114.31. IR (ATR, cm^{-1}): 1731 (C=O), 1671 (N-C=O). MS (EI) m/z (%): 381.0 (M^+, 13), 234.9 (M^+ -F -I, 94). HRMS (ESI) m/z: $[M+Na]^+$ Calcd for $[C_{21}H_{21}FINNaO_3]^+$ 504.0442; Found 504.0448 for compound: **10g**.

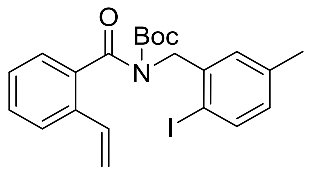

***tert*-Butyl (2-iodo-5-methylbenzyl)(2-vinylbenzoyl)carbamate (10h).** Following the first step of the general procedure J, *N*-(2-iodo-5-methylbenzyl)-2-vinylbenzamide was obtained as a white solid (1.79 g, 4.77 mmol, 80%) by FC (PE/EtOAc 9:1 to 7:3) starting from methyl 2-vinylbenzoate (1.01 g, 6.25 mmol), (2-iodo-5-methylphenyl)methanamine (1.62 g, 6.56 mmol) and LiHMDS 1 M in THF (18.76 mL, 18.76 mmol). R_f = 0.55 (PE/EtOAc 8:2). mp: 110-111 °C. ^{1}H-NMR (300 MHz, $CDCl_3$): δ 7.71 (d, *J* = 8.0 Hz, 1H), 7.57 (dd, *J* = 7.8, 1.3 Hz, 1H), 7.50 (dd, *J* = 7.6, 1.5 Hz, 1H), 7.41 (td, *J* = 7.6, 1.5 Hz, 1H), 7.34 – 7.26 (m, 2H), 7.08 (dd, *J* = 17.5, 11.0 Hz, 1H), 6.84 (dd, *J* = 8.1, 2.2 Hz, 1H), 6.33 (t, *J* = 5.9 Hz, 1H), 5.71 (dd, *J* = 17.5, 1.2 Hz, 1H), 5.35 (dd, *J* = 11.0, 1.2 Hz, 1H), 4.62 (d, *J* = 5.9 Hz, 2H), 2.32 (s, 3H). ^{13}C {^{1}H} NMR (75 MHz, $CDCl_3$): δ 169.1 (C), 140.0 (C), 139.4 (CH), 138.9 (C), 136.3 (C), 135.1 (C), 134.7 (CH), 131.3 (CH), 130.6 (CH), 130.5 (CH), 127.9 (CH), 127.7 (CH), 126.6 (CH), 117.1 (CH_2), 95.2 (C), 48.7 (CH_2), 21.1 (CH_3). IR (ATR, cm^{-1}): 3311 (N-H st), 1643 (C=O st), 1522 (C=C st). MS (EI) m/z (%): 377.8 (M^+, 100), 376.0 (70), 250.1 (M^+ - I, 53), 247.8 (24), 244.7 (30).. HRMS (ESI) m/z: $[M+H]^+$ Calcd for $[C_{17}H_7INO]^+$ 378.0349; Found 378.0360. Following the second step of general procedure J, **10h** was obtained as a white solid (2.14 g, 4.48 mmol, 94%) (overall yield for the two steps = 75%) by FC (PE/EtOAc 95:05) starting from *N*-(2-iodo-5-methylbenzyl)-2-vinylbenzamide (1.79 g, 4.77 mmol), di-*tert*-butyl dicarbonate (1.56 g, 7.15 mmol), DMAP (58.28 mg, 0.47 mmol) in CH_2Cl_2 (10 mL). R_f = 0.7 (PE/EtOAc 8:2). mp: 106-109 °C. ^{1}H-NMR (300 MHz, $CDCl_3$): δ 7.73 (d, *J* = 8.0 Hz, 1H), 7.60 (d, *J* = 8.0 Hz, 1H), 7.43 (ddd, *J* = 8.0 , 6.1, 2.6 Hz, 1H), 7.37 – 7.31 (m, 2H), 7.00 (t, *J* = 1.6 Hz, 1H), 6.92 (dd, *J* = 17.4, 11.0 Hz, 1H), 6.81 (dd, *J* = 8.0, 2.1 Hz,

1H), 5.76 (dd, *J* = 17.4, 1.1 Hz, 1H), 5.39 (dd, *J* = 11.0, 1.1 Hz, 1H), 5.04 (s, 2H), 2.32 (s, 3H), 1.12 (s, 9H). ^{13}C {^{1}H} NMR (75 MHz, $CDCl_3$): δ 172.2 (C), 152.4 (C), 139.4 (CH), 139.0 (C), 138.4 (C), 137.3 (C), 135.3 (C), 134.0 (CH), 129.8 (CH), 129.8 (CH), 127.5 (CH), 127.3 (CH), 126.3 (CH), 125.9 (CH), 117.1 (CH_2), 93.7 (C), 83.8 (C), 53.2 (CH_2), 27.4 (3 × CH_3), 21.3 (CH_3). IR (ATR, cm^{-1}): 1735 ($C=O_{carbamate}$ st), 1671 ($C=O_{amide}$ st), MS (EI) m/z (%): 378.0 (100), 376.0 (M^+ - Boc, 52), 359.9 (24), 250.1 (MH^+ - Boc - I, 61). HRMS (ESI) m/z: $[M+Na]^+$ Calcd for $[C_{22}H_{24}INNaO_3]^+$ 500.0693; Found 500.0694 for compound: **10h**.

General procedure K for the synthesis of *tert*-butyl (*E*)-5-oxo-5,7-dihydro-6*H*-dibenzo[*c*,*g*]azonine-6-carboxylate derivatives (11a-h). An oven-dried 250 mL flask provided with the corresponding *tert*-butyl (2-iodobenzyl)(2-vinylbenzoyl)carbamate **10a-h** (2.55 mmol) in dry DMF (12.8 mL) under Ar atmosphere, sodium acetate (418 mg, 5.10 mmol), triphenylphosphine (67 mg, 0.26 mmol) and palladium acetate (29 mg, 0.13 mmol) were added. The reaction mixture was let stir at 100 ºC for 72 h. Then, it was cooled down and water (20 mL) and EtOAc (20 mL) were added, the mixture was filtered by Celite®, the filtrate was extracted with EtOAc (4 × 20 mL) and the organic phase was washed with water (3 × 5 mL), brine (10 mL), dried with Na_2SO_4 anhydrous, filtered and concentrated under vacuum. The crude was purified by silica gel column chromatography.

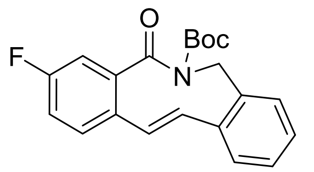

***tert*-Butyl (*E*)-3-fluoro-5-oxo-5,7-dihydro-6*H*-dibenzo[*c*,*g*]azonine-6-carboxylate (11b).** Following the general procedure K, **11b** was obtained as a yellow solid (129.2 g, 0.170 mmol, 26%) by FC (PE/EtOAc 9:1) starting from *tert*-butyl (5-fluoro-2-vinylbenzoyl)(2-iodobenzyl)carbamate **10b** (684.8 mg, 1.423), sodium acetate (234 mg, 2.85 mmol), triphenylphosphine (223.9 mg, 0.854 mmol) and palladium acetate (95.8 mg, 0.427 mmol) in DMF (7 mL). R_f = 0.30 (PE/EtOAc 9:1). mp: 149-151 ºC. ^{1}H NMR (300 MHz, $CDCl_3$) δ 7.33 – 7.02 (m, 7H), 6.46 (d, *J* = 16.1 Hz, 1H), 6.34 (d, *J* = 16.5 Hz, 1H), 5.30 (d, *J* = 15.8 Hz, 1H), 4.53 (d, *J* = 15.8 Hz, 1H), 0.95 (s, 9H). ^{13}C {^{1}H} NMR (75 MHz, $CDCl_3$) δ 176.3 (C), 162.4 (d, *J* = 248.8 Hz, C), 151.3 (C), 137.6 (CH), 137.4 (d, *J* = 4.7 Hz, C), 136.5 (CH), 133.7 (d, *J* = 3.4 Hz, C), 132.9 (C), 131.5 (d, *J* = 9.6 Hz, C), 129.5 (d, *J* = 8.1 Hz, CH), 128.1 (CH), 127.6 (CH), 127.2 (CH), 125.1 (CH), 117.3 (d, *J*

= 21.9 Hz, CH), 116.4 (d, J = 23.4 Hz, CH), 82.4 (C), 52.0 (CH_2), 27.6 (3 × CH_3). ^{19}F NMR (282 MHz, $CDCl_3$) δ -109.31. IR (ATR, cm^{-1}): 1724 (C=O), 1685 (N-C=O). MS (EI) m/z (%): 250.7 (M^+ - Boc, 100). HRMS (ESI) m/z: $[M+Na]^+$ Calcd for $[C_{21}H_{20}FNNaO_3]^+$ 376.1319; Found 376.1334 for compound: **11b**.

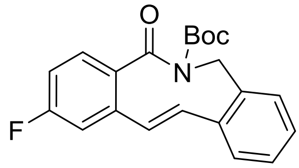

***tert*-Butyl (*E*)-2-fluoro-5-oxo-5,7-dihydro-6*H*-dibenzo[*c*,*g*]azonine-6-carboxylate (11c).** Following the general procedure K **11c** was obtained as a yellow solid (208.0 mg, 0.589 mmol, 16%) by FC (PE/EtOAc 9:1) starting from *tert*-butyl (4-fluoro-2-vinylbenzoyl)(2-iodobenzyl)carbamate **10c** (1.77 g, 3.68 mmol), sodium acetate (603 mg, 7.35 mmol), triphenylphosphine (578.1 mg, 2.208 mmol) and palladium acetate (247.9 mg, 1.104 mmol) in DMF (18 mL). R_f = 0.32 (PE/EtOAc 9:1). mp: 162-164 ºC. ^1H NMR (300 MHz, $CDCl_3$) δ 7.59 (dd, J = 8.5, 5.6 Hz, 1H), 7.32 – 7.11 (m, 4H), 7.07 (td, J = 8.5, 2.5 Hz, 1H), 7.03 – 6.84 (d, J = 9.0, 2.5 Hz, 1H), 6.51 (d, J = 16.6 Hz, 1H), 6.35 (d, J = 16.6 Hz, 1H), 5.28 (d, J = 15.8 Hz, 1H), 4.53 (d, J = 15.8 Hz, 1H), 0.94 (s, 9H). ^{13}C {^1H} NMR (75 MHz, $CDCl_3$) δ 176.8 (C), 163.3 (d, J = 251.8 Hz, C), 151.3 (C), 140.2 (d, J = 8.6 Hz, C), 137.4 (CH), 137.4 (C), 136.2 (C), 132.9 (C), 131.5 (d, J = 9.2 Hz, CH), 128.1 (CH), 127.6 (CH), 127.5 (CH), 127.1 (d, J = 2.3 Hz, CH), 125.0 (CH), 115.4 (d, J = 22.2 Hz, CH), 114.5 (d, J = 21.6 Hz, CH), 82.2 (C), 52.0 (CH_2), 27.6 (3 × CH_3). ^{19}F NMR (282 MHz, $CDCl_3$) δ -109.32. IR (ATR, cm^{-1}): 1723 (C=O), 1673 (N-C=O). MS (EI) m/z (%): 251.8 (M^+ - Boc, 15). HRMS (ESI) m/z: $[M+Na]^+$ Calcd for $[C_{21}H_{20}FNNaO_3]^+$ 376.1319; Found 376.1320 for compound: **11c**.

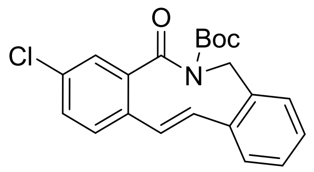

***tert*-Butyl (*E*)-3-chloro-5-oxo-5,7-dihydro-6*H*-dibenzo[*c*,*g*]azonine-6-carboxylate (11d).** Following the general procedure K, **11d** was obtained as a yellow solid (248.2 mg, 0.682 mmol, 23%) by FC (PE/EtOAc 9:1) starting from *tert*-butyl (5-chloro-2-vinylbenzoyl)(2-iodobenzyl)carbamate **10d** (1.491 g, 2.996 mmol), sodium acetate (491 mg, 5.991 mmol), triphenylphosphine (471 mg, 1.797 mmol) and palladium acetate (202 mg, 0.899 mmol) in DMF (15 mL). R_f = 0.30 (PE/EtOAc 9:1). mp: 184-186 ºC. ^{1}H NMR (300 MHz, $CDCl_3$) δ 7.61 (d, *J* = 2.2 Hz, 1H), 7.40 (dd, *J* = 8.2, 2.2 Hz, 1H), 7.32 – 7.18 (m, 5H), 6.53 (d, *J* = 16.6 Hz, 1H), 6.37 (d, *J* = 16.6 Hz, 1H), 5.34 (d, *J* = 15.8 Hz, 1H), 4.57 (d, *J* = 15.8 Hz, 1H), 0.99 (s, 9H). ^{13}C {^{1}H} NMR (75 MHz, $CDCl_3$) δ 176.3 (C), 151.2 (C), 141.2 (C), 137.5 (C), 136.9 (CH), 135.9 (C), 134.2 (C), 132.8 (C), 130.1 (CH), 129.4 (CH), 129.1 (CH), 128.1 (CH), 127.6 (CH), 127.4 (CH), 127.0 (CH), 125.1 (CH), 82.5 (C), 51.9 (CH_2), 27.5 (3 × CH_3). IR (ATR, cm^{-1}): 1721 (C=O), 1680 (N-C=O). MS (EI) m/z (%): 268.0 (M^+ - Boc, 100). HRMS (ESI) m/z: $[M+Na]^+$ Calcd for $[C_{21}H_{20}ClNNaO_3]^+$ 392.1024; Found 392.1028 for compound: **11d**.

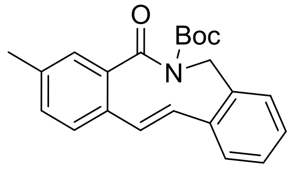

***tert*-Butyl (*E*)-3-methyl-5-oxo-5,7-dihydro-6*H*-dibenzo[*c*,*g*]azonine-6-carboxylate (11e).** Following the general procedure K, **11e** was obtained as a white solid (1.42 g, 4.01 mmol, 68%) by FC (PE/EtOAc 98:02 to PE/EtOAc 90:10) starting from *tert*-butyl (2-iodobenzyl)(5-methyl-2-vinylbenzoyl)carbamate **10e** (2.86 g, 6.04 mmol), sodium acetate (992 mg, 12.09 mmol), triphenylphosphine (317 mg, 1.21 mmol) and palladium

acetate (136 mg, 0.60 mmol) in DMF (30.2 mL) R_f = 0.32 (PE/EtOAc 95:05). mp: 138 - 140 °C. ^{1}H NMR (300 MHz, $CDCl_3$): δ7.51 – 7.46 (m, 1H), 7.36 – 7.19 (m, 6H), 6.52 (d, *J* = 16.6 Hz, 1H), 6.44 (d, *J* = 16.6 Hz, 1H), 5.38 (d, *J* = 15.8 Hz, 1H), 4.59 (d, *J* = 15.8 Hz, 1H), 2.41 (s, 3H), 0.99 (s, 9H). ^{13}C {^{1}H} NMR (75 MHz, $CDCl_3$): δ 178.1 (C), 151.6 (C), 139.9 (C), 138.2 (C), 138.1 (C), 135.8 (CH), 135.1 (C), 133.2 (C), 131.1 (CH), 130.1 (CH) , 128.4 (CH), 128.2 (CH), 127.9 (CH), 127.6 (CH), 127.3 (CH), 125.2 (CH), 82.1 (C), 52.1 (CH_2), 27.6 (3 × CH_3), 21.3 (CH_3). IR (ATR, cm^{-1}): 1725 ($C{=}O_{carbamate}$ st), 1671.98 ($C{=}O_{amide}$ st). MS (EI) m/z (%): 249.1 (MH^+ - Boc, 100), 131.9 (81), 115.9 (41), 104.0 (71), 88.9 (75), 77.1 (35). HRMS (ESI) m/z: $[M+Na]^+$ Calcd for $[C_{22}H_{23}NNaO_3]^+$ 372.1570; Found 372.1571 for compound: **11e**.

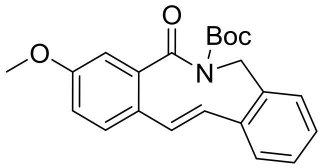

***tert*-Butyl (*E*)-3-methoxy-5-oxo-5,7-dihydro-6*H*-dibenzo[*c*,*g*]azonine-6-carboxylate (11f).** Following the general procedure K, **11f** was obtained as a white solid (1.65 g, 4.5 mmol, 64%) by FC (PE/EtOAc 95:05) starting from *tert*-butyl (2-iodobenzyl)(5-methoxy-2-vinylbenzoyl)carbamate **10f** (3.47 g, 7.04 mmol), sodium acetate (1.15 g, 14.08 mmol), triphenylphosphine (369.41 mg, 1.41 mmol) and palladium acetate (237.13 mg, 1.06 mmol) in DMF (35 mL). R_f = 0.3 (PE/EtOAc 95:05). mp: 135-137 °C. ^{1}H-NMR (300 MHz, $CDCl_3$): δ 7.35 – 7.19 (m, 6H), 7.05 (dd, *J* = 8.5, 2.7 Hz, 1H), 6.50 (d, *J* = 16.7 Hz, 1H), 6.43 (d, *J* = 16.7 Hz, 1H), 5.40 (d, *J* = 15.8 Hz, 1H), 4.61 (d, *J* = 15.8 Hz, 1H), 3.88 (s, 3H), 1.03 (s, 9H).^{13}C {^{1}H} NMR (75 MHz, $CDCl_3$): δ 177.7 (C), 159.6 (C), 151.5 (C), 141.1 (C), 138.1 (C), 135.3 (CH), 133.1 (C), 130.6 (C), 129.2 (CH), 128.1 (CH), 128.1 (CH), 127.6 (CH), 127.3 (CH), 125.3 (CH), 117.4 (CH), 113.8 (CH), 82.2 (C), 55.6 (CH_3), 52.1 (CH_2), 27.6 (3 × CH_3). IR (ATR, cm^{-1}): 1725 ($C{=}O_{carbamate}$ st), 1673

(C=O$_{amide}$ st). MS (EI) m/z (%): 264.1 (M^+ - Boc, 29), 263.1 (100). HRMS (ESI) m/z: $[M+Na]^+$ Calcd for $[C_{22}H_{23}NNaO_4]^+$ 388.1519; Found 388.1522 for compound: **11f**.

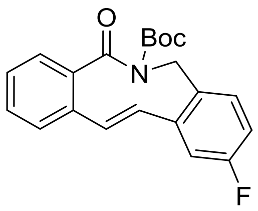

***tert*-Butyl (*E*)-2-fluoro-7-oxo-5,7-dihydro-6*H*-dibenzo[*c*,*g*]azonine-6-carboxylate (11g).** Following the general procedure K, **11g** was obtained as a solid (779 mg, 2.21 mmol, 45%) by FC (PE/EtOAc 95:5) starting from *tert*-butyl (4-fluoro-2-iodobenzyl)(2-vinylbenzoyl)carbamate **10g** (2.30 g, 4.90 mmol), sodium acetate (804 mg, 9.80 mmol), triphenylphosphine (125 mg, 0.48 mmol) and palladium acetate (54 mg, 0.24 mmol) in DMF (12.8 mL). R_f = 0.36 (PE/EtOAc 95:5). mp: 135-138 ºC. ^{1}H NMR (300 MHz, $CDCl_3$) δ 7.56 (dd, *J* = 7.2, 1.9 Hz, 1H), 7.32 (td, *J* = 7.1, 1.7 Hz, 2H), 7.22 (dd, *J* = 7.1, 1.8 Hz, 1H), 7.12 (dd, *J* = 8.0, 5.4 Hz, 1H), 6.90 – 6.82 (m, 2H), 6.39 (d, *J* = 16.7 Hz, 1H), 6.33 (d, *J* = 16.7 Hz, 1H), 5.22 (d, *J* = 15.8 Hz, 1H), 4.48 (d, *J* = 15.8 Hz, 1H), 0.87 (s, 9H). ^{13}C {^{1}H} NMR (75 MHz, $CDCl_3$) δ 177.4 (C), 161.8 (d, *J* = 246.2 Hz, C), 151.3 (C), 139.9 (C), 139.6 (d, *J* = 8.2 Hz, C), 137.2 (C), 134.9 (d, *J* = 2.0 Hz, CH), 130.2 (CH) , 129.7 (d, *J* = 8.3 Hz, CH), 129.6 (CH), 129.0 (CH), 128.9 (d, *J* = 4.6 Hz, C), 128.2 (CH), 128.0 (CH), 113.8 (d, *J* = 21.1 Hz, CH), 112.5 (d, *J* = 22.1 Hz, CH), 82.1 (C), 51.2 (CH_2), 27.5 (3 × CH_3). ^{19}F NMR (282 MHz, $CDCl_3$) δ -114.79. IR (ATR, cm^{-1}): 1724 (C=O), 1673 (N-C=O). MS (EI) m/z (%): 251.1 (M^+ - Boc). HRMS (ESI) m/z: $[M+Na]^+$ Calcd for $[C_{21}H_{20}FNNaO_3]^+$ 376.1319; Found 376.1322 for compound: **11g**.

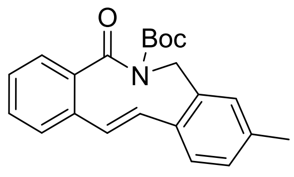

***tert*-Butyl (*E*)-3-methyl-7-oxo-5,7-dihydro-6*H*-dibenzo[*c*,*g*]azonine-6-carboxylate (11h).** Following the general procedure K, **11h** was obtained as a white solid (0.78 g, 3.14 mmol, 92%) by FC (PE/EtOAc 95:05) starting from *tert*-butyl (2-iodo-5-methylbenzyl)(2-vinylbenzoyl)carbamate **10h** (1.64 g, 3.43 mmol), sodium acetate (562.6 mg, 6.86 mmol), triphenylphosphine (269.8 mg, 1.03 mmol) and palladium acetate (154.0 mg, 0.69 mmol) in DMF (17 mL). R_f = 0.3 (PE/EtOAc 95:05). mp: 138-141 °C. ^{1}H-NMR (300 MHz, $CDCl_3$): δ 7.69 (dd, *J* = 7.4, 1.7 Hz, 1H), 7.54 – 7.40 (m, 2H), 7.36 (dd, *J* = 7.4, 1.7 Hz, 1H), 7.20 – 7.09 (m, 3H), 6.56 (d, *J* = 16.6 Hz, 1H), 6.49 (d, *J* = 16.6 Hz, 1H), 5.38 (d, *J* = 15.8 Hz, 1H), 4.58 (d, *J* = 15.8 Hz, 1H), 2.38 (s, 3H), 1.00 (s, 9H). ^{13}C {^{1}H} NMR (75 MHz, $CDCl_3$): δ 178.0 (C), 151.6 (C), 140.2 (C), 138.0 (C), 137.0 (C), 136.6 (CH), 135.0 (C), 132.9 (C), 130.2 (CH), 129.6 (CH), 128.9 (CH), 128.4 (CH), 128.1 (CH), 128.1 (CH), 128.0 (CH), 125.1 (CH), 82.1 (C), 52.1 (CH_2), 27.6 (3 × CH_3), 21.4 (CH_3). IR (ATR, cm^{-1}): 1726 ($C{=}O_{carbamate}$ st), 1673 ($C{=}O_{amide}$ st). MS (EI) m/z (%): 248.1 (M^+ - Boc, 51), 247.0 (100), 246.0 (18). HRMS (ESI) m/z: $[M+Na]^+$ Calcd for $[C_{22}H_{23}NNaO_3]^+$ 372.1570; Found 372.1580 for compound: **11h**.

General procedure L for the synthesis of (*E*)-6,7-dihydro-5*H*-dibenzo[*c*,*g*]azonin-5-one derivatives (12a-h). The corresponding *tert*-butyl (*E*)-5-oxo-5,7-dihydro-6*H*-dibenzo[*c*,*g*]azonine-6-carboxylate **11a-h** (0.2 mmol) was dissolved in CH_2Cl_2 (5 mL) and silica gel (230-400 mesh) (2 g) was added. The solvent was taken off on vacuum and the powdered solid obtained was irradiated in the microwave oven, in an open erlenmeyer flask at 540 watts. The reaction is checked by TLC every 6 minutes until it was completed. The reaction mixture was disadsorbed by thoroughly washing the silica gel with PE/EtOAC 1:1 with pressure in a column. The crude was purified by silica gel column chromatography.

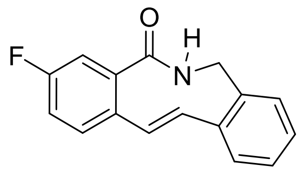

(*E*)-3-Fluoro-6,7-dihydro-5*H*-dibenzo[*c*,*g*]azonin-5-one (12b). Following the general procedure L, **12b** was obtained as a white solid (40.5 mg, 0.160 mmol, 80%) by FC (PE/EtOAc 8:2 to 7:3) starting from *tert*-butyl (*E*)-3-fluoro-5-oxo-5,7-dihydro-6*H*-dibenzo[*c,g*]azonine-6-carboxylate **11b** (70.7 mg, 0.2 mmol) and silica gel (2.00 g). R_f = 0.17 (PE/EtOAc 8:2). mp: 205-206 6ºC. ^{1}H NMR (300 MHz, $CDCl_3$) δ 7.72 (dd, *J* = 8.2, 5.7 Hz, 1H), 7.42 – 7.18 (m, 4H), 7.12 (t, *J* = 8.6 Hz, 2H), 6.77 (d, *J* = 16.9 Hz, 1H), 6.45 (d, *J* = 16.9 Hz, 1H), 5.45 (dd, *J* = 16.2, 10.7 Hz, 1H), 4.90 (d, *J* = 10.7 Hz, 1H), 4.28 (d, *J* = 16.1 Hz, 1H). ^{13}C {^{1}H} NMR (75 MHz, $CDCl_3$) δ 175.0 (C), 163.4 (d, *J* = 251.5 Hz, C), 140.7 (d, *J* = 8.6 Hz, C), 138.7 (C), 137.2 (CH), 134.7 (C), 132.5 (C), 131.4 (d, *J* = 9.3 Hz, CH), 128.0 (CH), 128.0 (CH), 127.6 (CH), 127.5 (CH), 125.7 (CH), 115.5 (d, *J* = 22.0 Hz, CH), 115.4 (d, *J* = 21.5 Hz, CH), 48.9 (CH_2). ^{19}F NMR (282 MHz, $CDCl_3$) δ -109.12. IR (ATR, cm^{-1}): 3282 (N-H), 1637 (N-C=O), 1514 (C=C). MS (EI) m/z (%):

252.0 (M^+, 100). HRMS (ESI) m/z: $[M+H]^+$ Calcd for $[C_{16}H_{13}FNO]^+$ 254.0981; Found 254.0988 for compound: **12b**.

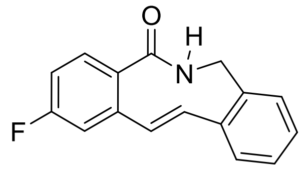

(*E*)-2-Fluoro-6,7-dihydro-5*H*-dibenzo[*c*,*g*]azonin-5-one (12c). Following the general procedure L, **12c** was obtained as a white solid (29.4 mg, 0.116 mmol, 58%) by FC (PE/EtOAc 8:2 to 7:3) starting from *tert*-butyl (*E*)-2-fluoro-5-oxo-5,7-dihydro-6*H*-dibenzo[*c,g*]azonine-6-carboxylate **11c** (70.7 mg, 0.200 mmol) and silica gel (2.00 g). R_f = 0.15 (PE/EtOAc 8:2). mp: 178-179 ºC. ^{1}H NMR (300 MHz, $CDCl_3$) δ 7.72 (dd, *J* = 8.2, 5.7 Hz, 1H), 7.42 – 7.18 (m, 4H), 7.12 (t, *J* = 8.6 Hz, 2H), 6.77 (d, *J* = 16.9 Hz, 1H), 6.45 (d, *J* = 16.9 Hz, 1H), 5.45 (dd, *J* = 16.2, 10.7 Hz, 1H), 4.90 (d, *J* = 10.7 Hz, 1H), 4.28 (d, *J* = 16.1 Hz, 1H). ^{13}C {^{1}H} NMR (75 MHz, $CDCl_3$) δ 175.0 (C), 163.4 (d, *J* = 251.5 Hz, C), 140.7 (d, *J* = 8.6 Hz, C), 138.7 (C), 137.2 (CH), 134.7 (C), 132.5 (C), 131.4 (d, *J* = 9.3 Hz, CH), 128.0 (CH), 128.0 (CH), 127.6 (CH), 127.5 (CH), 125.7 (CH), 115.5 (d, *J* = 22.0 Hz, CH), 115.4 (d, *J* = 21.5 Hz, CH), 48.9 (CH_2). ^{19}F NMR (282 MHz, $CDCl_3$) δ -109.12. IR (ATR, cm^{-1}): 3305 (N-H), 1639 (N-C=O), 1498 (C=C). MS (EI) m/z (%): 252.0 (M^+, 100). HRMS (ESI) m/z: $[M+H]^+$ Calcd for $[C_{16}H_{13}FNO]^+$ 254.0981; Found 254.0988 for compound: **12c**.

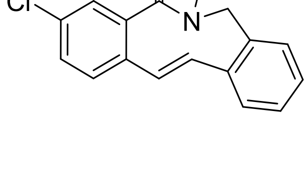

(*E*)-3-Chloro-6,7-dihydro-5*H*-dibenzo[*c*,*g*]azonin-5-one (12d). Following the general procedure L, **12d** was obtained as a white oil (33.4 mg, 0.124 mmol, 62%) by FC

(PE/EtOAc 8:2 to 7:3 to 1:1) starting from *tert*-butyl (*E*)-3-chloro-5-oxo-5,7-dihydro-6*H*-dibenzo[*c,g*]azonine-6-carboxylate **11d** (74.9 mg, 0.2 mmol) and silica gel (2.00 g), in CH_2Cl_2 (3.4 mL). R_f = 0.12 (PE/EtOAc 8:2). ^{1}H NMR (300 MHz, $CDCl_3$) δ 7.69 (d, *J* = 2.2 Hz, 1H), 7.44 (dd, *J* = 8.3, 2.2 Hz, 1H), 7.30 (m, 5H), 6.74 (d, *J* = 16.9 Hz, 1H), 6.42 (d, *J* = 16.9 Hz, 1H), 5.45 (dd, *J* = 16.2, 10.6 Hz, 1H), 4.86 (d, *J* = 10.5 Hz, 1H), 4.27 (d, *J* = 16.1 Hz, 1H). ^{13}C {^{1}H} NMR (75 MHz, $CDCl_3$) δ 174.5 (C), 139.7 (C), 138.8 (C), 136.8 (CH), 136.7 (C), 134.4 (C), 132.4 (C), 130.6 (CH), 130.2 (CH), 129.5 (CH), 128.0 (CH), 128.0 (CH), 127.5 (CH), 127.4 (CH), 125.7 (CH), 48.9 (CH_2). IR (ATR, cm^{-1}): 3304 (N-H), 1638 (N-C=O), 1495 (C=C). MS (EI) m/z (%): 268.1 (M+, 100). HRMS (ESI) m/z: $[M+H]^+$ Calcd for $[C_{16}H_{13}ClNO]^+$ 270.0686; Found 270.0691 for compound: **12d**.

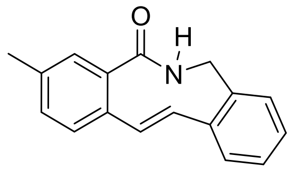

(*E*)-3-Methyl-6,7-dihydro-5*H*-dibenzo[*c,g*]azonin-5-one (12e). Following the general procedure L, **12e** was obtained as a white solid (29.4 mg, 0,118 mmol, 59%) by FC (PE/EtOAc 8:2 to 7:3) starting from *tert*-butyl (*E*)-3-methyl-5-oxo-5,7-dihydro-6*H*-dibenzo[*c,g*]azonine-6-carboxylate **11e** (69.9 mg, 2.00 mmol) and silica gel (2.00 g). R_f = 0.39 (PE/EtOAc 9:1). mp: 207 °C - 210 °C. ^{1}H NMR (300 MHz, $CDCl_3$): 7.52 (s, 1H), 7.41 – 7.16 (m, 6H), 6.68 (d, *J* = 17.0 Hz, 1H), 6.45 (d, *J* = 16.9 Hz, 1H), 5.45 (dd, *J* = 16.1, 10.7 Hz, 1H), 4.85 (d, *J* = 10.7 Hz, 1H), 4.25 (d, *J* = 16.1 Hz, 1H), 2.41 (s, 3H). ^{13}C {^{1}H} NMR (75 MHz, $CDCl_3$): δ 176.2 (C), 139.3 (C), 138.3 (C), 138.3 (C), 135.6 (C), 135.5 (CH), 132.7 (C), 131.3 (CH), 129.8 (CH), 128.8 (CH), 128.6 (CH), 128.0 (CH), 127.9 (CH), 127.2 (CH), 125.7 (CH), 49.0 (CH_2), 21.1 (CH_3). IR (ATR, cm^{-1}): 3283 (N-H st), 1637 (C=O st). MS (EI) m/z (%): 249.1 (M^+, 100), 132.1 (81), 102.9 (44), 88.8

(33). HRMS (ESI) m/z: $[M+H]^+$ Calcd for $[C_{17}H_{16}NO]^+$ 250.1226; Found 250.1236 for compound: **12e**.

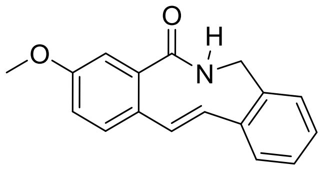

(*E*)-3-Methoxy-6,7-dihydro-5*H*-dibenzo[*c*,*g*]azonin-5-one (12f). Following the general procedure L, **12f** was obtained as a white solid (47.7 mg, 0.18 mmol, 69%) by FC (PE/EtOAc 8:2 to 7:3) starting from *tert*-butyl (*E*)-3-methoxy-5-oxo-5,7-dihydro-6H-dibenzo[*c*,*g*]azonine-6-carboxylate **11f** (94.8 mg, 0.26 mmol), and silica gel (2.60 g). R_f = 0.36 (PE/EtOAc 8:2). mp: 169-173 °C. ^{1}H-NMR (300 MHz, $CDCl_3$): δ 7.40 – 7.20 (m, 6H), 7.04 (dd, *J* = 8.5, 2.8 Hz, 1H), 6.65 (d, *J* = 16.9 Hz, 1H), 6.44 (d, *J* = 16.9 Hz, 1H), 5.46 (dd, *J* = 16.1, 10.7 Hz, 1H), 4.90 (d, *J* = 10.8 Hz, 1H), 4.27 (d, *J* = 16.1 Hz, 1H), 3.88 (s, 3H). ^{13}C {^{1}H} NMR (75 MHz, $CDCl_3$): δ 176.0 (C), 159.6 (C), 139.7 (C), 139.5 (C), 135.0 (CH), 132.8 (C), 131.2 (C), 130.3 (CH), 128.5 (CH), 128.1 (CH), 128.0 (CH), 127.3 (CH), 125.9 (CH), 117.7 (CH), 113.4 (CH), 55.7 (CH_3), 49.1 (CH_2). IR (ATR, cm^{-1}): 3276 (N-H st), 1639 (C=O st). MS (EI) m/z (%): 265.1 (M^+, 45), 264.1 (38), 263.1 (100). HRMS (ESI) m/z: $[M+H]^+$ Calcd for $[C_{17}H_{16}NO_2]^+$ 266.1176; Found 266.1173 for compound: **12f**.

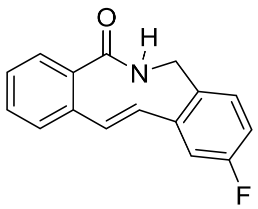

(*E*)-10-Fluoro-6,7-dihydro-5*H*-dibenzo[*c*,*g*]azonin-5-one (12g). Following the general procedure L, **12g** was obtained as a white solid (37,7 mg, 0.149 mmol, 74%) by FC (PE/EtOAc 8:2 to 7:3) starting from *tert*-butyl (*E*)-2-fluoro-7-oxo-5,7-dihydro-6*H*-

dibenzo[*c,g*]azonine-6-carboxylate **11g** (70.7 mg, 0.2 mmol) and silica gel (2.00 g). R_f = 0.12 (PE/EtOAc 8:2). mp: 198-201 ºC. ^{1}H NMR (300 MHz, $CDCl_3$) δ 7.72 (d, *J* = 7.7 Hz, 1H), 7.62 – 7.38 (m, 3H), 7.20 (dd, *J* = 8.4, 5.5 Hz, 1H), 7.05 (dd, *J* = 8.7, 2.7 Hz, 1H), 6.97 (td, *J* = 8.5, 2.8 Hz, 1H), 6.67 (d, *J* = 17.0 Hz, 1H), 6.52 (d, *J* = 17.0 Hz, 1H), 5.42 (dd, *J* = 16.1, 10.7 Hz, 1H), 4.87 (d, *J* = 10.9 Hz, 1H), 4.26 (d, *J* = 16.0 Hz, 1H). ^{13}C {^{1}H} NMR (75 MHz, $CDCl_3$) δ 175.9 (C), 162.1 (d, *J* = 247.6 Hz, C), 141.0 (d, *J* =8.4 Hz, C), 138.3 (C), 137.9 (C), 134.7 (CH), 134.7 (CH), 130.5 (CH), 129.6 (d, *J* = 8.4 Hz, CH), 129.3 (CH), 129.0 (CH), 128.4 (C), 128.3 (CH), 113.8 (d, *J* = 21.0 Hz, CH), 113.1 (d, *J* = 22.0 Hz, CH), 48.3 (CH_2). ^{19}F NMR (282 MHz, $CDCl_3$) δ -114.14. IR (ATR, cm^{-1}): 3292 (N-H), 1633 (N-C=O), 1514 (C=C). MS (EI) m/z (%): 252.2 (M+, 100). HRMS (ESI) m/z: $[M+H]^+$ Calcd for $[C_{16}H_{13}FNO]^+$ 254.0981; Found 254.0988 for compound: **12g**.

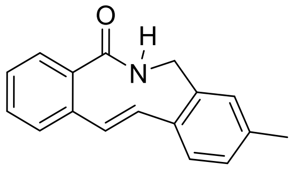

(*E*)-9-Methyl-6,7-dihydro-5*H*-dibenzo[*c,g*]azonin-5-one (12h). Following the general procedure L, **12h** was obtained as a white solid (32.9 mg, 0.132 mmol, 66%) by FC (PE/EtOAc 8:2 to 7:3) starting from *tert*-butyl (*E*)-3-methyl-7-oxo-5,7-dihydro-6*H*-dibenzo[*c*,*g*]azonine-6-carboxylate **11h** (69.9 mg, 0.2 mmol), and silica gel (2.00 g). R_f = 0.3 (PE/EtOAc 8:2). mp: 200-204°C. ^{1}H-NMR (300 MHz, $CDCl_3$): δ 7.74 – 7.69 (m, 1H), 7.50 – 7.36 (m, 3H), 7.20 (d, *J* = 7.8 Hz, 1H), 7.14 (d, *J* = 7.8 Hz, 1H), 7.05 (s, 1H), 6.71 (d, *J* = 16.9 Hz, 1H), 6.47 (d, *J* = 16.9 Hz, 1H), 5.44 (dd, *J* = 16.1, 11.2 Hz, 1H), 4.84 (d, *J* = 11.2 Hz, 1H), 4.22 (d, *J* = 16.1 Hz, 1H), 2.36 (s, 3H). ^{13}C {^{1}H} NMR (75 MHz, $CDCl_3$) δ 176.2 (C), 138.6 (C), 138.6 (C), 137.1 (C), 136.3 (CH), 136.3 (C) , 132.6 (C), 130.5 (CH), 129.5 (CH), 129.1 (CH), 128.9 (CH), 128.8 (CH), 128.5 (CH), 128.2 (CH), 125.7

(CH), 49.1 (CH_2), 21.3 (CH_3). IR (ATR, cm^{-1}): 3275 (N-H st), 1633 (C=O st), 1515 (C=C st). MS (EI) m/z (%): 249.10 (M^+, 100), 248.10 (51), 119.0 (74), 118.0 (73), 90.0 (76), 89.0 (44). HRMS (ESI) m/z: $[M+H]^+$ Calcd for $[C_{17}H_{16}NO]^+$ 250.1226; Found 250.1227 for compound: **12h**.

General procedure M for the synthesis of isoindolo[2,1-*b*]isoquinolin-5(7*H*)-one derivatives (13a-h). To reaction tube provided with a magnetic bar with the corresponding (*E*)-6,7-dihydro-5*H*-dibenzo[*c,g*]azonin-5-one **12a-h** (23.5 mg, 0.100 mmol), a Stock solution of diphenylphosphoric acid (0.6 mg, 0.003 mmol) in dry dichloromethane (200 µL) was added at 25 ºC followed by the addition of *N*-bromosuccinimide (18 mg, 0.101 mmol). The reaction mixture was followed by TLC (CH_2Cl_2 : MeOH 98:2). Once the starting material was consumed, DBU (18 µL, 0.120 mmol) and NaI (18 mg, 0.120 mmol) were added to the reaction mixture at 25 ºC. The new reaction mixture was let stirring and was followed by TLC until the intermediate was consumed. Then, std. aq. solution of NH_4Cl (1 mL) was added, the layers were separated, and aqueous phase was extracted with CH_2Cl_2 (3 × 1 mL). All organic layers were washed with water (3 × 1 mL), brine (1 mL), dried with Na_2SO_4, filtrated and concentrated under vacuum. The crude was purified by silica gel flash chromatography.

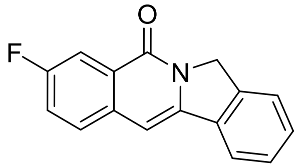

3-Fluoroisoindolo[2,1-*b*]isoquinolin-5(7*H*)-one (13b). Following the general procedure M **13b** was obtained as a solid (10.8 mg, 0.0430 mmol, 83%) by FC (CH_2Cl_2 to CH_2Cl_2/MeOH 99:1) starting from (*E*)-3-fluoro-6,7-dihydro-5*H*-dibenzo[*c*,*g*]azonin-5-one **12b** (13.2 mg, 0.052 mmol), NBS (9.4 mg, 0.053 mmol), a Stock solution of diphenylphosphoric acid (0.0.3 mg, 0.0013 mmol) in dry CH_2Cl_2 (100 µL), NaI (9.4 mg, 0.063 mmol) and DBU (9.4 µL, 0.063 mmol). Reaction time for first step: 5h. Reaction time for second step: 48h. R_f = 0.49 (PE/EtOAc 1:1). mp: 116-118 ºC. ^{1}H NMR (300 MHz, $CDCl_3$) δ 8.14 (dd, *J* = 9.4, 2.8 Hz, 1H), 7.80 (dd, *J* = 5.4, 3.3 Hz, 1H), 7.65 (dd, *J* = 8.8, 5.1 Hz, 1H), 7.59 (dd, *J* = 5.4, 3.5 Hz, 1H), 7.50 (dd, *J* = 5.7, 3.1 Hz, 2H), 7.41 (td,

J = 8.5, 2.8 Hz, 1H), 7.03 (s, 1H), 5.20 (s, 2H). $^{13}C\ \{^1H\}$ NMR (75 MHz, $CDCl_3$) δ 161.2 (d, J = 247.1 Hz, C), 160.4 (d, J = 3.8 Hz, C), 141.8 (d, J = 2.7 Hz, C), 137.5 (C), 134.6 (d, J = 1.7 Hz, C), 134.0 (C), 130.0 (CH), 128.7 (d, J = 7.8 Hz, CH), 128.6 (CH), 126.3 (d, J = 8.1 Hz, C), 123.6 (CH), 121.2 (d, J = 23.8 Hz, CH), 121.1 (CH), 112.7 (d, J = 22.8 Hz, CH), 97.5 (CH), 52.2 (CH_2). ^{19}F NMR (282 MHz, $CDCl_3$) δ -113.51. IR (ATR, cm^{-1}): 1656 (N-C=O), 1611 (C=C). MS (EI) m/z (%): 251.2 (M+, 93). HRMS (ESI) m/z: $[M+H]^+$ Calcd for $[C_{16}H_{11}FNO]^+$ 252.0825; Found 252.0827 for compound: **13b**.

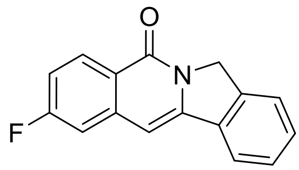

2-fluoroisoindolo[2,1-*b*]isoquinolin-5(7*H*)-one (13c). Following the general procedure M **13c** was obtained as a solid (11.4 mg, 0.0452 mmol, 74%) by FC (CH_2Cl_2 to CH_2Cl_2/MeOH 99:1) starting from (*E*)-2-fluoro-6,7-dihydro-5*H*-dibenzo[*c*,*g*]azonin-5-one **12c** (15.7 mg, 0.062 mmol), NBS (11.1 mg, 0.063 mmol), a Stock solution of diphenylphosphoric acid (0.4 mg, 0.0016 mmol) in dry CH_2Cl_2 (130 μL), NaI (11.1 mg, 0.0744 mmol) and DBU (11 μL, 0.0744 mmol). R_f = 0.49 (PE/EtOAc 1:1). Reaction time for first step: 5h. Reaction time for second step: 48h. mp: 211-214 ºC. ^1H NMR (300 MHz, $CDCl_3$) δ 8.51 (dd, J = 8.9, 5.8 Hz, 1H), 7.85 – 7.78 (m, 1H), 7.66 – 7.57 (m, 1H), 7.57 – 7.47 (m, 2H), 7.28 (dd, J = 9.5, 2.5 Hz, 1H), 7.19 (td, J = 8.6, 2.5 Hz, 1H), 6.98 (s, 1H), 5.20 (s, 2H). $^{13}C\ \{^1H\}$ NMR (75 MHz, $CDCl_3$) δ 165.2 (d, J = 252.0 Hz, C), 160.5 (C), 143.5 (C), 140.3 (d, J = 10.4 Hz, C), 137.9 (C), 133.7 (C), 130.6 (d, J = 10.3 Hz, CH), 130.2 (CH), 128.5 (CH), 123.5 (CH), 121.4 (d, J = 1.5 Hz, C), 121.2 (CH), 114.8 (d, J = 23.6 Hz, CH), 111.0 (d, J = 22.0 Hz, CH), 97.4 (d, J = 3.4 Hz, CH), 52.1 (CH_2). ^{19}F NMR (282 MHz, $CDCl_3$) δ -107.06. IR (ATR, cm^{-1}): 1653 (N-C=O), 1613 (C=C).

MS (EI) m/z (%): 251.0 (M^+, 100). HRMS (ESI) m/z: $[M+H]^+$ Calcd for $[C_{16}H_{11}FNO]^+$ 252.0825; Found 252.0823 for compound: **13c**.

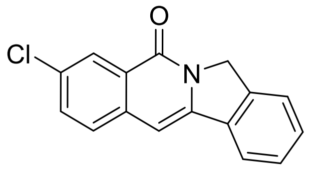

3-Chloroisoindolo[2,1-*b*]isoquinolin-5(7*H*)-one (13d). Following the general procedure M, **13d** was obtained as a solid (10.8 mg, 0.0403 mmol, 96%) by FC (CH_2Cl_2 to CH_2Cl_2/MeOH 99:1) starting from (*E*)-3-chloro-6,7-dihydro-5*H*-dibenzo[*c*,*g*]azonin-5-one **12d** (11.3 mg, 0.0422 mmol), NBS (7.6 mg, 0.0426 mmol), a Stock solution of diphenylphosphoric acid (0.3 mg, 0.0012 mmol) in dry CH_2Cl_2 (85 µL), NaI (7.6 mg, 0.0507 mmol) and DBU (7.6 µL, 0.0507 mmol). Reaction time for first step: 5h. Reaction time for second step: 48h. R_f = 0.49 (PE/EtOAc 1:1). mp: 227-229 ºC. ^{1}H NMR (300 MHz, $CDCl_3$) δ 8.45 (d, *J* = 1.9 Hz, 1H), 7.84 – 7.76 (m, 1H), 7.59 (q, *J* = 3.8, 3.3 Hz, 3H), 7.54 – 7.47 (m, 2H), 6.99 (s, 1H), 5.19 (s, 2H). ^{13}C {^{1}H} NMR (75 MHz, $CDCl_3$) δ 160.0 (C), 142.6 (C), 137.6 (C), 136.3 (C), 133.8 (C), 132.6 (CH), 132.0 (C), 130.1 (CH), 128.5 (CH), 127.9 (CH), 126.9 (CH), 125.7 (C), 123.5 (CH), 121.1 (CH), 97.4 (CH), 52.2 (CH_2). IR (ATR, cm^{-1}): 1648 (N-C=O), 1626 (C=C). MS (EI) m/z (%): 267.0 (M+, 100). HRMS (ESI) m/z: $[M+H]^+$ Calcd for $[C_{16}H_{11}ClNO]^+$ 268.0529; Found 268.0533 for compound: **13d**.

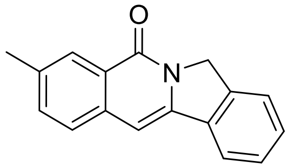

3-Methylisoindolo[2,1-*b*]isoquinolin-5(7*H*)-one (13e). Following the general procedure M, **13e** was obtained as a pink solid (22.6 mg, 0.092 mmol, 92%) by FC (PE/EtOAc 8:2

to PE/EtOAc 6:4) starting from (*E*)-3-methyl-6,7-dihydro-5*H*-dibenzo[*c*,*g*]azonin-5-one **12e** (24.9 mg, 0.100 mmol), NBS (18 mg, 0.101 mmol), a Stock solution of diphenylphosphoric acid (0.63 mg, 0.0025 mmol) in dry CH_2Cl_2 (200 µL), NaI (18 mg, 0.12 mmol) and DBU (18 µL, 18.3 mg, 0.120 mmol). Reaction time for first step: 5h. Reaction time for second step: 72h. R_f= 0.73 (PE/EtOAc 7:3). mp: 197 - 198 °C. ^{1}H NMR (300 MHz, $CDCl_3$): 8.28 (s, 1H), 7.81 – 7.71 (m, 1H), 7.59 – 7.50 (m, 2H), 7.50 – 7.41 (m, 3H), 6.98 (s, 1H), 5.17 (s, 2H), 2.50 (s, 3H). ^{13}C {^{1}H} NMR (75 MHz, $CDCl_3$): δ 161.2 (C), 141.4 (C), 137.7 (C), 136.4 (C), 135.7 (C), 134.3 (C), 133.8 (CH), 129.7 (CH), 128.4 (CH), 127.1 (CH), 126.4 (CH), 124.8 (C), 123.6 (CH), 121.0 (CH), 98.1 (CH), 52.2 (CH_2), 21.6 (CH_3). IR (ATR, cm^{-1}): 1652 (C=O st). 1606 (C_{Arom}-C_{Arom} st). MS (EI) m/z (%): 247.0 (M^+, 100), 245.9 (88), 207.0 (21). HRMS (ESI) m/z: $[M+H]^+$ Calcd for $[C_{17}H_{14}NO]^+$ 248.1070; Found 248.1082 for compound: **13e**.

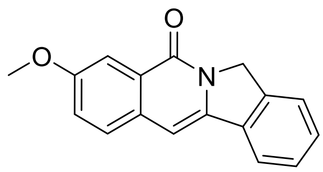

3-Methoxyisoindolo[2,1-*b*]isoquinolin-5(7*H*)-one (13f). Following the general procedure M, **13f** was obtained as a yellow solid (11.8 mg, 0.04 mmol, 45%) by FC (PE/EtOAc 1:1) starting from (*E*)-3-methoxy-6,7-dihydro-5*H*-dibenzo[*c*,*g*]azonin-5-one (26.6 mg, 0.1 mmol) **12f**, NBS (17.97 mg, 0.101 mmol), a Stock solution of diphenylphosphoric acid (0.62 mg, 0.0025 mmol) in dry CH_2Cl_2 (0.2 mL) after 26 hours (the addition of NaI and DBU was not necessary). R_f = 0.4 (PE/EtOAc 1:1). m.p: 171-180 °C. ^{1}H-NMR (300 MHz, $CDCl_3$): δ 7.92 – 7.88 (m, 1H), 7.81 – 7.74 (m, 1H), 7.62 – 7.55 (m, 2H), 7.50 – 7.43 (m, 2H), 7.29 (dd, J = 8.7, 2.8 Hz, 1H), 7.02 (s, 1H), 5.21 (s, 2H), 3.96 (s, 3H).^{13}C {^{1}H} NMR (75 MHz, $CDCl_3$): δ 159.8 (C), 157.5 (C), 139.1 (C), 136.2 (C), 133.3 (C), 131.0 (C), 128.4 (CH), 127.3 (CH), 127.0 (CH), 122.4 (CH), 122.1

(C), 122.0 (CH), 119.7 (CH), 106.2 (CH), 97.1 (CH), 54.7 (CH_2), 51.1 (CH_3). IR (ATR, cm^{-1}): 1655 (C=O st), 1606 (C=C st). MS (EI) m/z (%): 263.0 (M^+, 100), 262.0 (12), 248.0 (M^+- CH_3, 52), 232.0 (M^+ - OCH_3, 12), 220.0 (34), 191.0 (28). HRMS (ESI) m/z: $[M+H]^+$ Calcd for $[C_{17}H_{14}NO_2]^+$ 264.1019; Found 264.1017 for compound: **13f**.

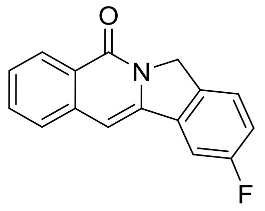

10-Fluoroisoindolo[2,1-*b*]isoquinolin-5(7*H*)-one (13g). Following the general procedure M **13g** was obtained as a solid (18.4 mg, 0.0732 mmol, 99%) by FC (CH_2Cl_2 to CH_2Cl_2/MeOH 99:1) starting from (*E*)-10-fluoro-6,7-dihydro-5*H*-dibenzo[*c*,*g*]azonin-5-one **12g** (18.6 mg, 0.0734 mmol), NBS (13.2 mg, 0.0742 mmol), a Stock solution of diphenylphosphoric acid (0.5 mg, 0.002 mmol) in dry CH_2Cl_2 (150 μL), NaI (13.2 mg, 0.088 mmol) and DBU (11 μL, 0.088 mmol). Reaction time for first step: 5h. Reaction time for second step: 48h. R_f = 0.49 (PE/EtOAc 1:1). mp: 242-245 ºC. ^1H NMR (300 MHz, CDl_3) δ 8.41 (dd, *J* = 8.1, 1.2 Hz, 1H), 7.65 – 7.53 (m, 2H), 7.49 – 7.40 (m, 2H), 7.38 (dd, *J* = 8.4, 2.5 Hz, 1H), 7.11 (td, *J* = 8.7, 2.4 Hz, 1H), 6.92 (s, 1H), 5.08 (t, *J* = 1.2 Hz, 2H). ^{13}C {^1H} NMR (75 MHz, $CDCl_3$) δ 163.2 (d, *J* = 246.5 Hz, C), 161.1 (C), 141.5 (d, *J* = 3.9 Hz, C), 137.8 (C), 136.1 (d, *J* = 9.4 Hz, C), 133.3 (d, *J* = 2.3 Hz, C), 132.4 (CH), 127.6 (CH), 126.7 (CH), 126.7 (CH), 125.1 (d, *J* = 8.9 Hz, CH), 125.1 (C) 117.4 (d, *J* = 23.6 Hz, CH), 108.0 (d, *J* = 24.2 Hz, CH), 98.9 (CH), 51.7 (CH_2). ^{19}F NMR (282 MHz, $CDCl_3$) δ -113.08. IR (ATR, cm^{-1}): 1653 (N-C=O), 1623 (C=C). MS (EI) m/z (%): 251.0 (M^+, 100). HRMS (ESI) m/z: $[M+H]^+$ Calcd for $[C_{16}H_{11}FNO]^+$ 252.0825; Found 252.0830 for compound: **13g**.

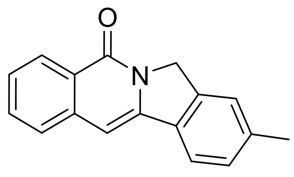

9-Methylisoindolo[2,1-*b*]isoquinolin-5(7*H*)-one (13h). Following the general procedure M, **13h** was obtained as a pale orange solid (20.1 mg, 0.082 mmol, 82%) by FC (PE/EtOAc 1:1) starting from (*E*)-9-methyl-6,7-dihydro-5*H*-dibenzo[*c*,*g*]azonin-5-one **12h** (24.9 mg, 0.1 mmol), NBS (18.0 mg, 0.101 mmol), a Stock solution of diphenylphosphoric acid (0.62 mg, 0.0025 mmol) in dry CH_2Cl_2 (0.2 mL), NaI (18.0 mg, 0.12 mmol) and DBU (18 μL, 0.12 mmol). Reaction time for first step: 5h. Reaction time for second step: 72h. R_f = 0.58 (PE/EtOAc 1:1). m.p: 180-186 °C. ^{1}H-NMR (300 MHz, $CDCl_3$): δ 8.40 (d, *J* = 8.1 Hz, 1H), 7.62 – 7.47 (m, 3H), 7.37 (ddd, *J* = 8.1, 6.6, 1.8 Hz, 1H), 7.28 (s, 1H), 7.22 – 7.16 (m, 1H), 6.87 (s, 1H), 5.05 (s, 2H), 2.37 (s, 3H). ^{13}C {^{1}H} NMR (75 MHz, $CDCl_3$): δ 161.3 (C), 142.4 (C), 140.5 (C), 138.2 (C), 138.0 (C), 132.2 (CH), 131.6 (C), 129.5 (CH), 127.6 (CH), 126.4 (CH), 126.0 (CH), 124.7 (C), 124.0 (CH), 120.9 (CH), 97.6 (CH), 52.0 (CH_2), 21.9 (CH_3). IR (ATR, cm^{-1}): 1653 (C=O st), 1585 (C=C st). MS (EI) m/z (%): 247.1 (M^+, 100), 246.1 (67), 232.1 ($M^+ - CH_3$, 33). HRMS (ESI) m/z: $[M+H]^+$ Calcd for $[C_{17}H_{14}NO]^+$ 248.1070; Found 248.1071 for compound: **13h**.

3. NMR Spectra

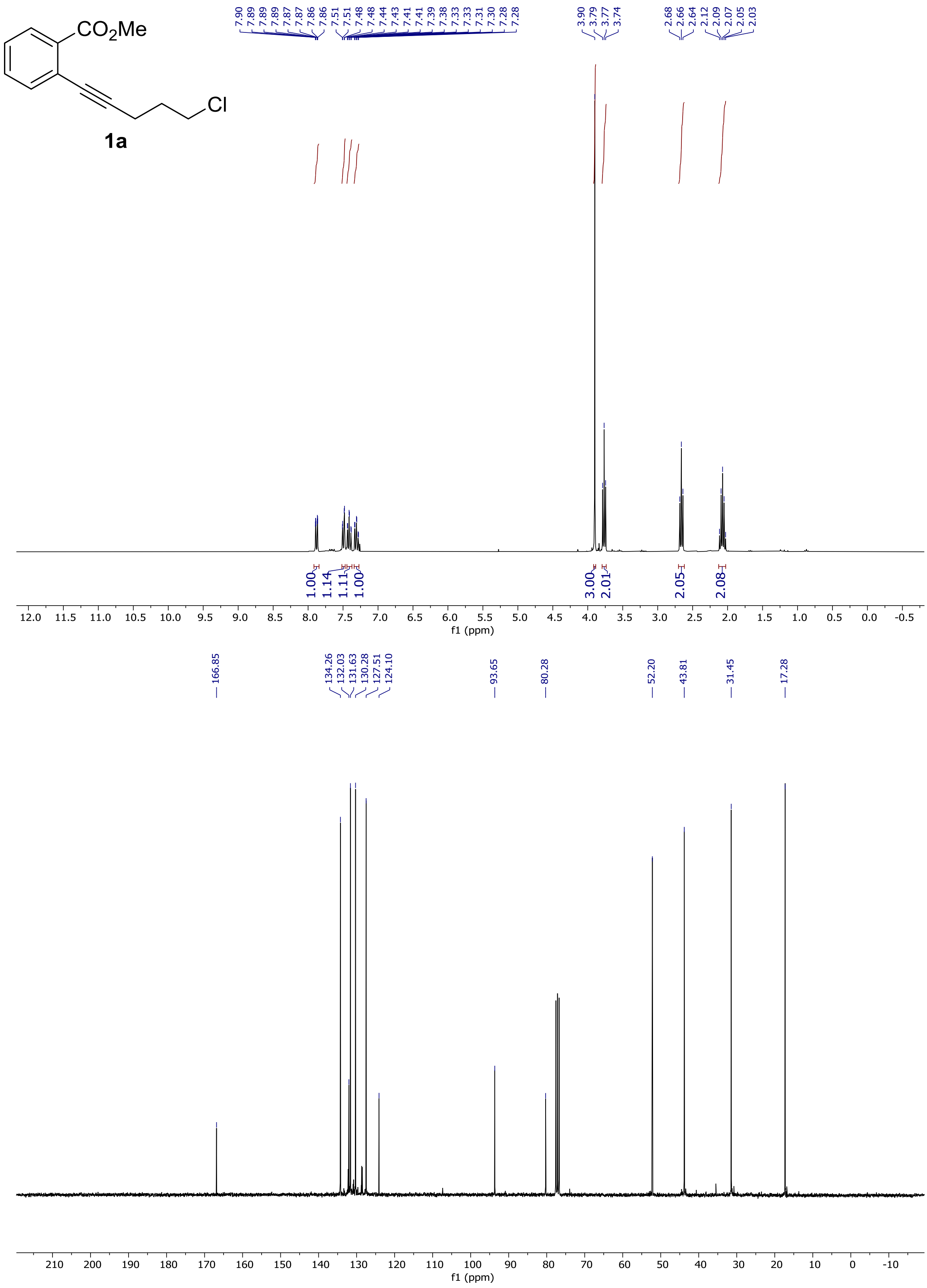

Figure SI-1. ^{1}H-NMR (300 MHz, $CDCl_3$) and ^{13}C {^{1}H} NMR (75 MHz, $CDCl_3$) spectra of compound **1a**

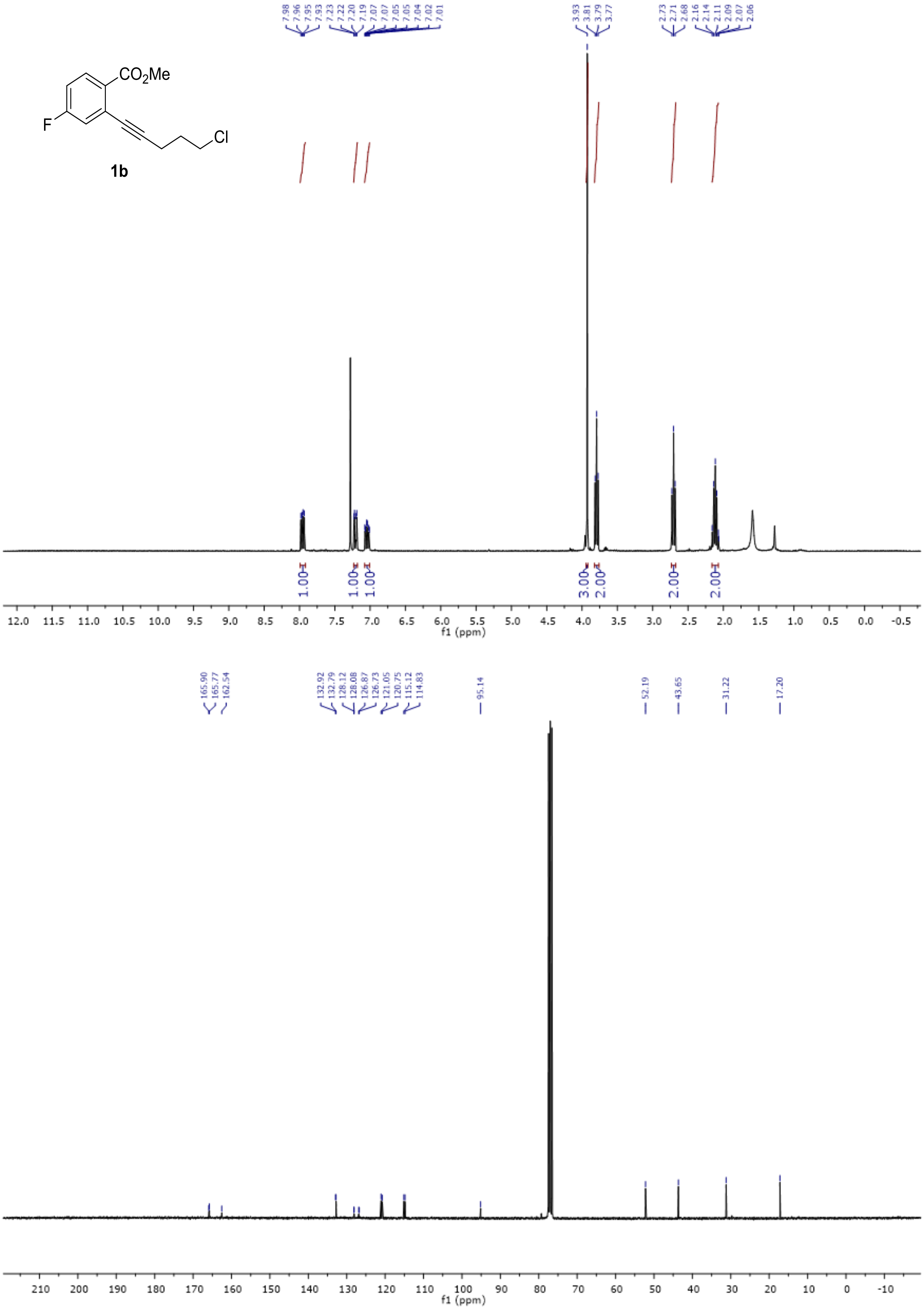

Figure SI-2. ^{1}H-NMR (300 MHz, $CDCl_3$) and ^{13}C {^{1}H} NMR (75 MHz, $CDCl_3$) spectra of compound **1b**

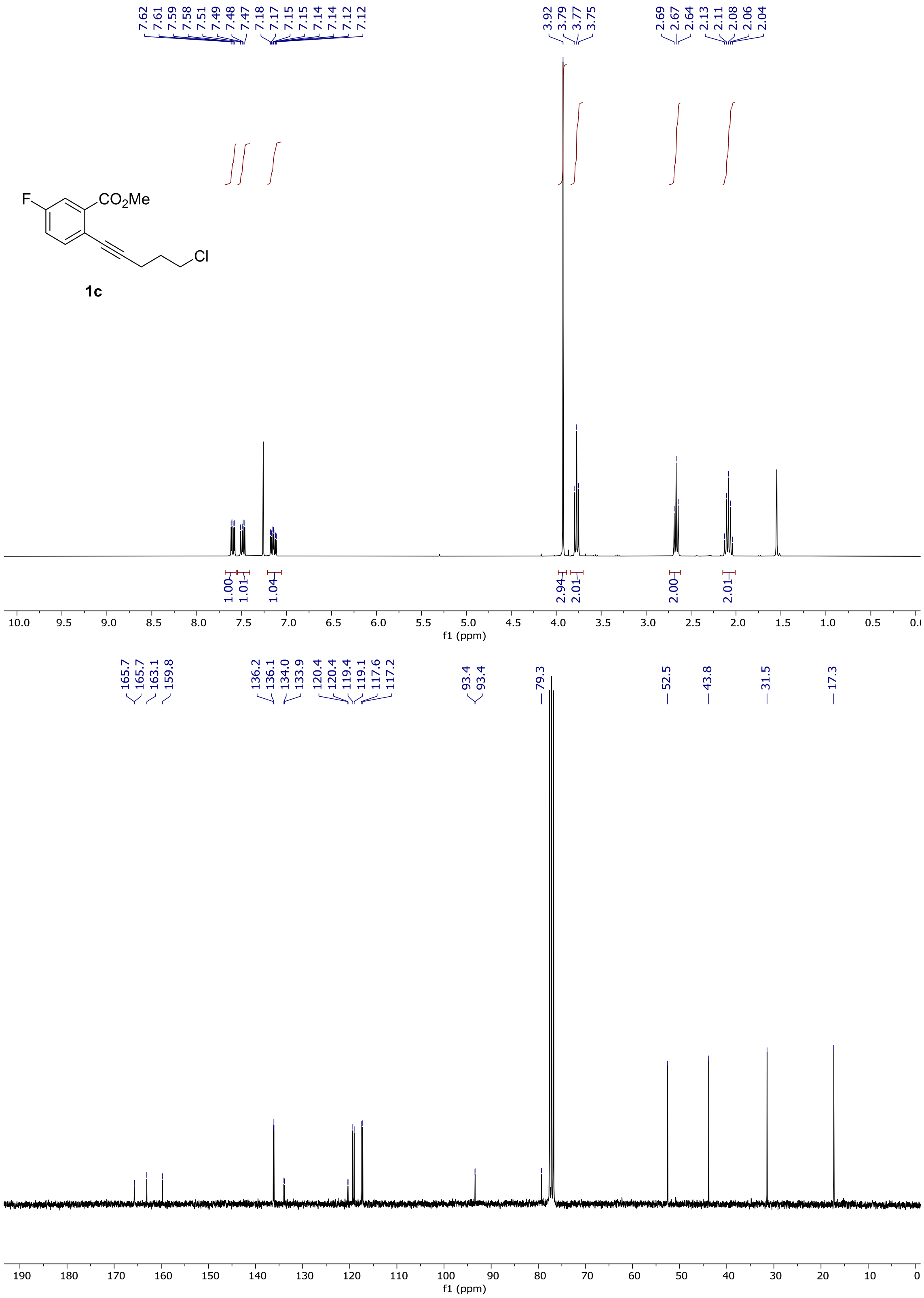

Figure SI-3. ^{1}H-NMR (300 MHz, $CDCl_3$) and ^{13}C {^{1}H} NMR (75 MHz, $CDCl_3$) spectra of compound **1c**

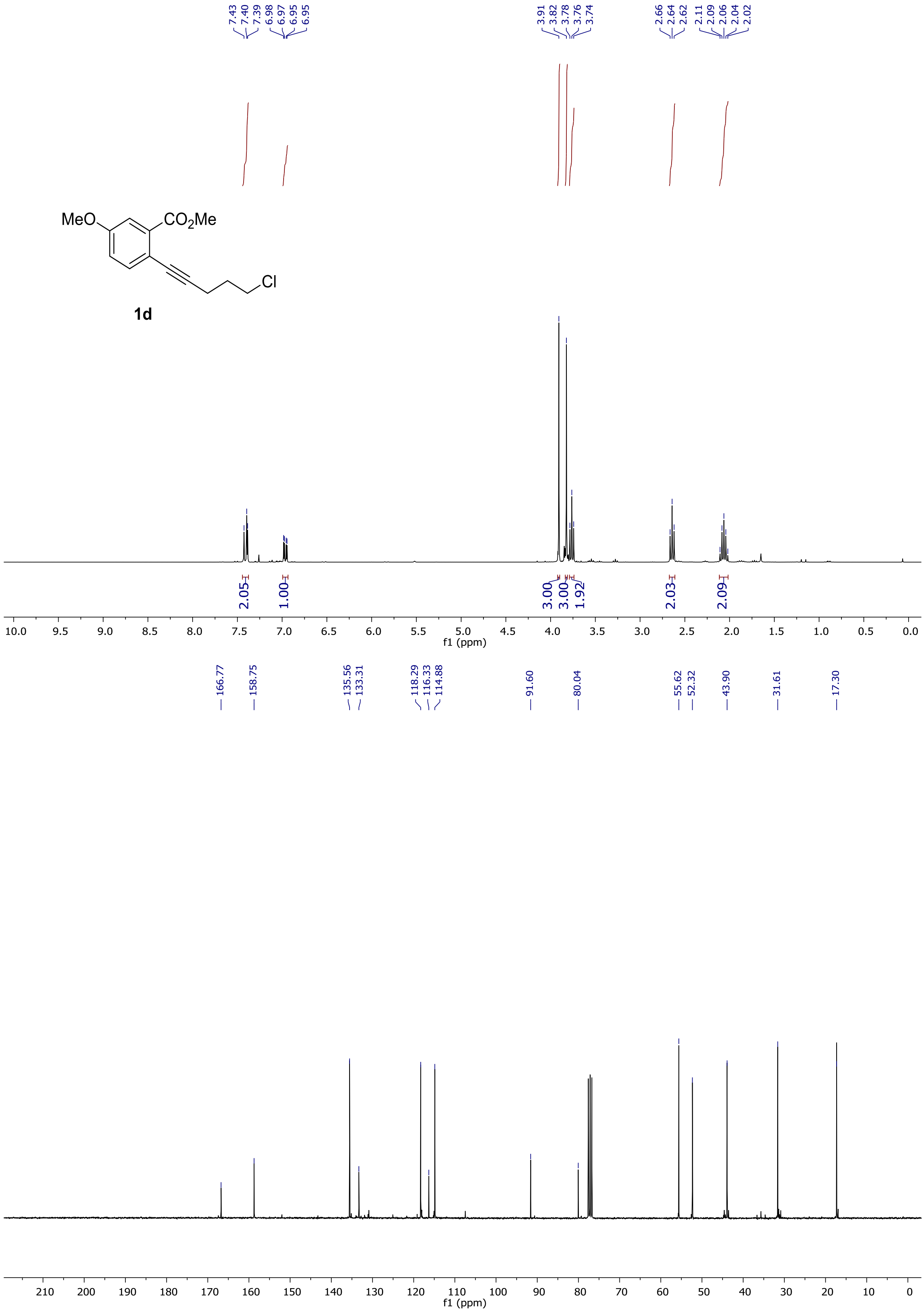

Figure SI-4. ^{1}H-NMR (300 MHz, $CDCl_3$) and ^{13}C {^{1}H} NMR (75 MHz, $CDCl_3$) spectra of compound **1d**

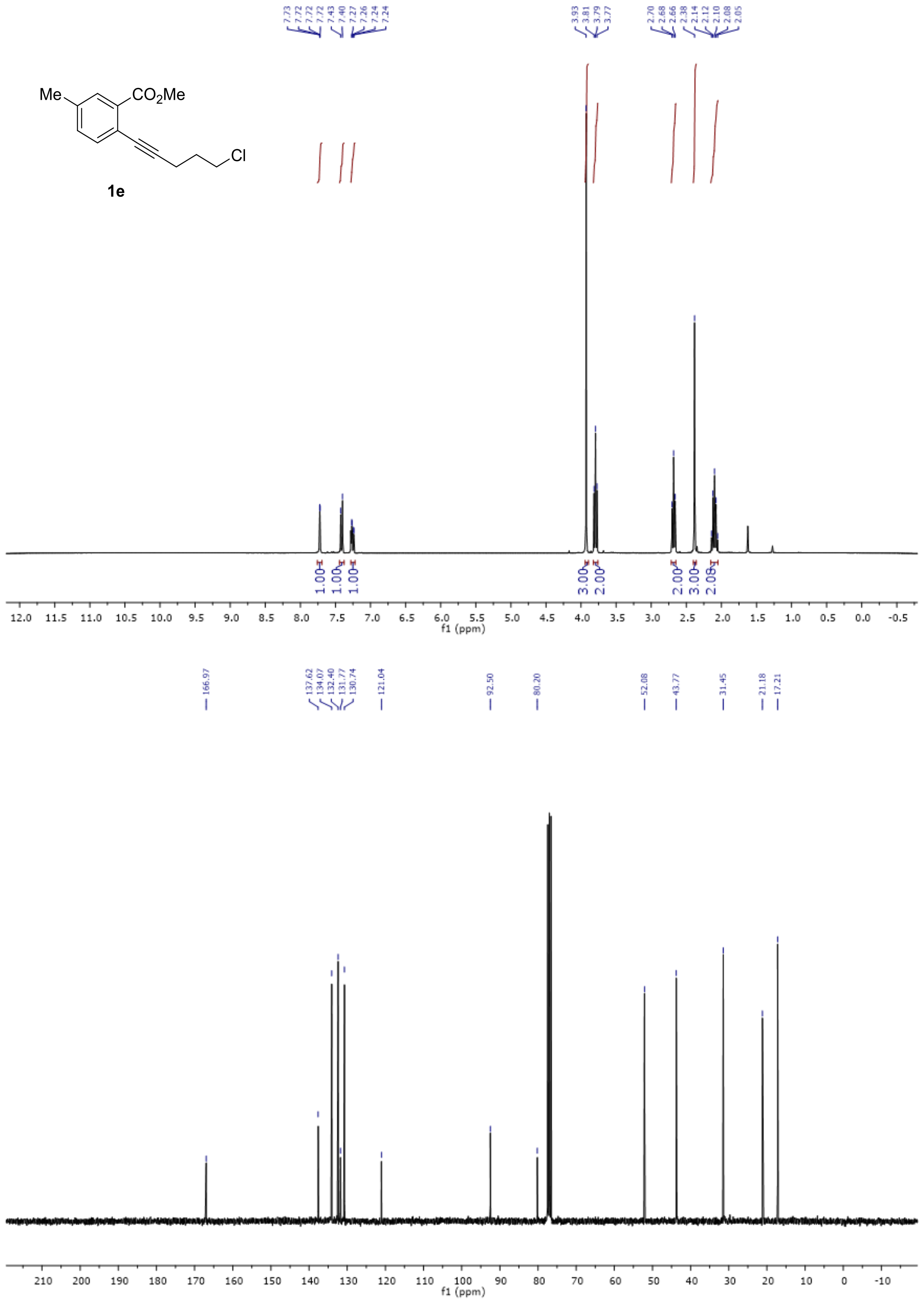

Figure SI-5. ^{1}H-NMR (300 MHz, $CDCl_3$) and ^{13}C {^{1}H} NMR (75 MHz, $CDCl_3$) spectra of compound **1e**

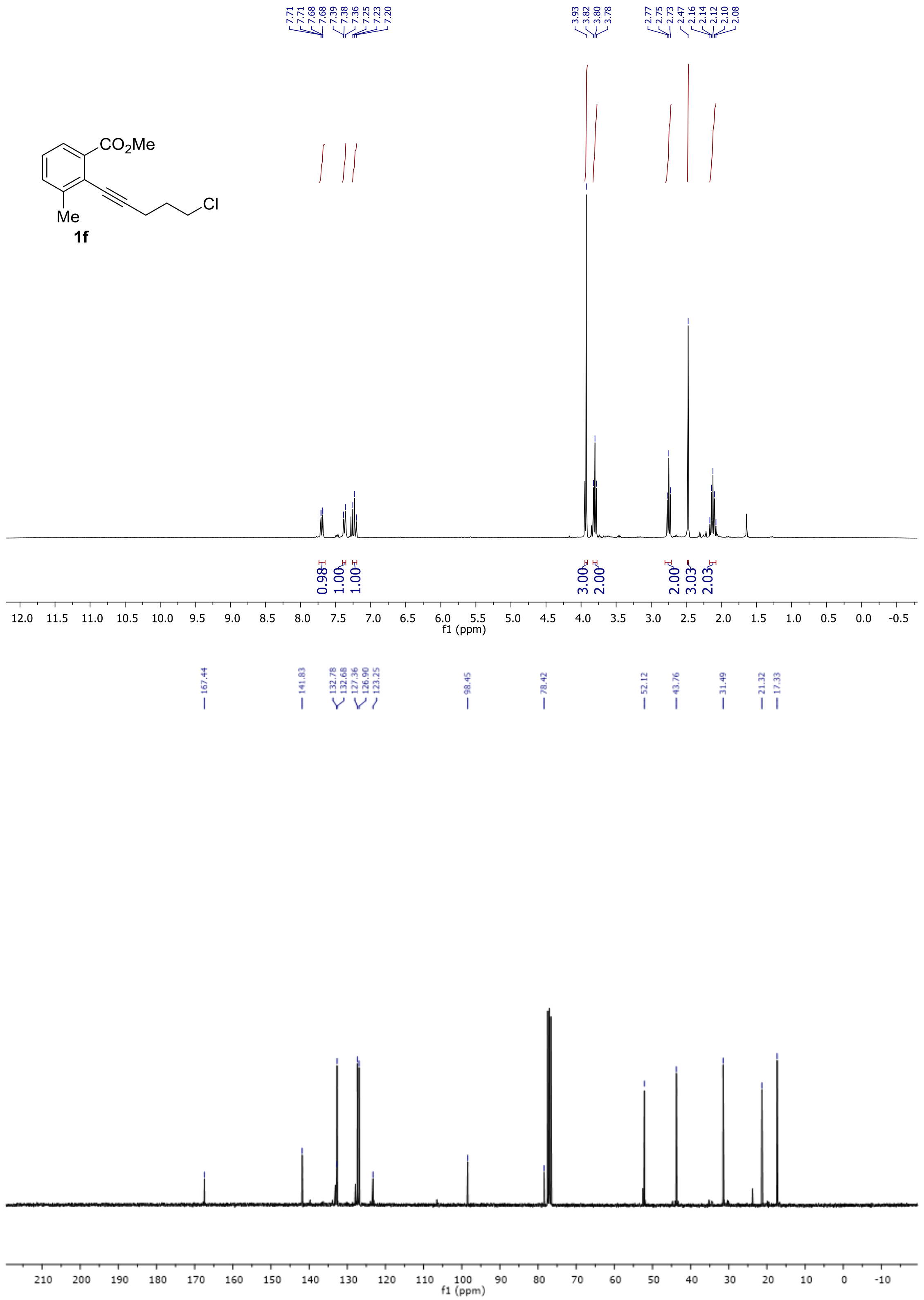

Figure SI-6. ^{1}H-NMR (300 MHz, $CDCl_3$) and ^{13}C {^{1}H} NMR (75 MHz, $CDCl_3$) spectra of compound **1f**

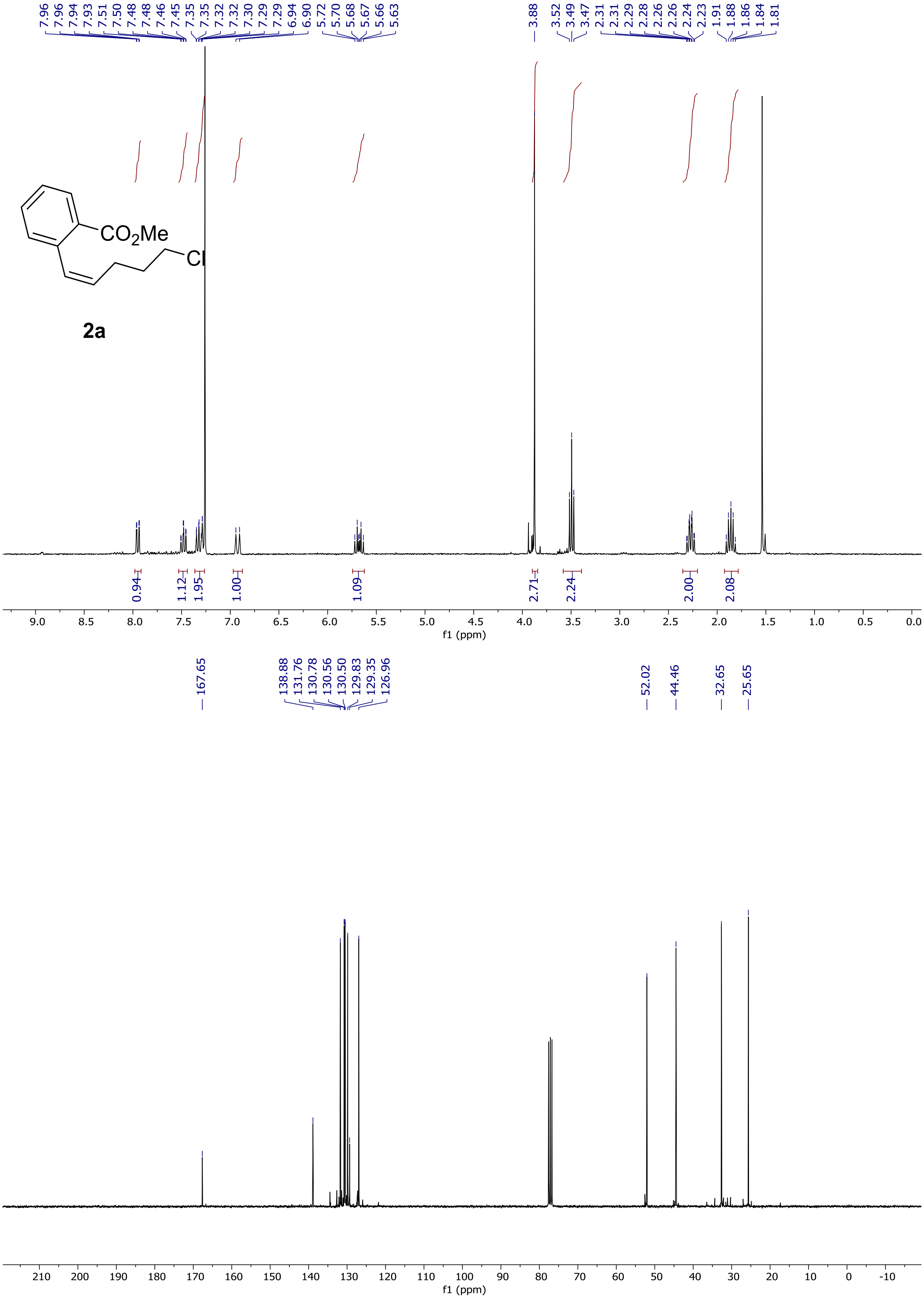

Figure SI-7. ^{1}H-NMR (300 MHz, $CDCl_3$) and ^{13}C {^{1}H} NMR (75 MHz, $CDCl_3$) spectra of compound **2a**

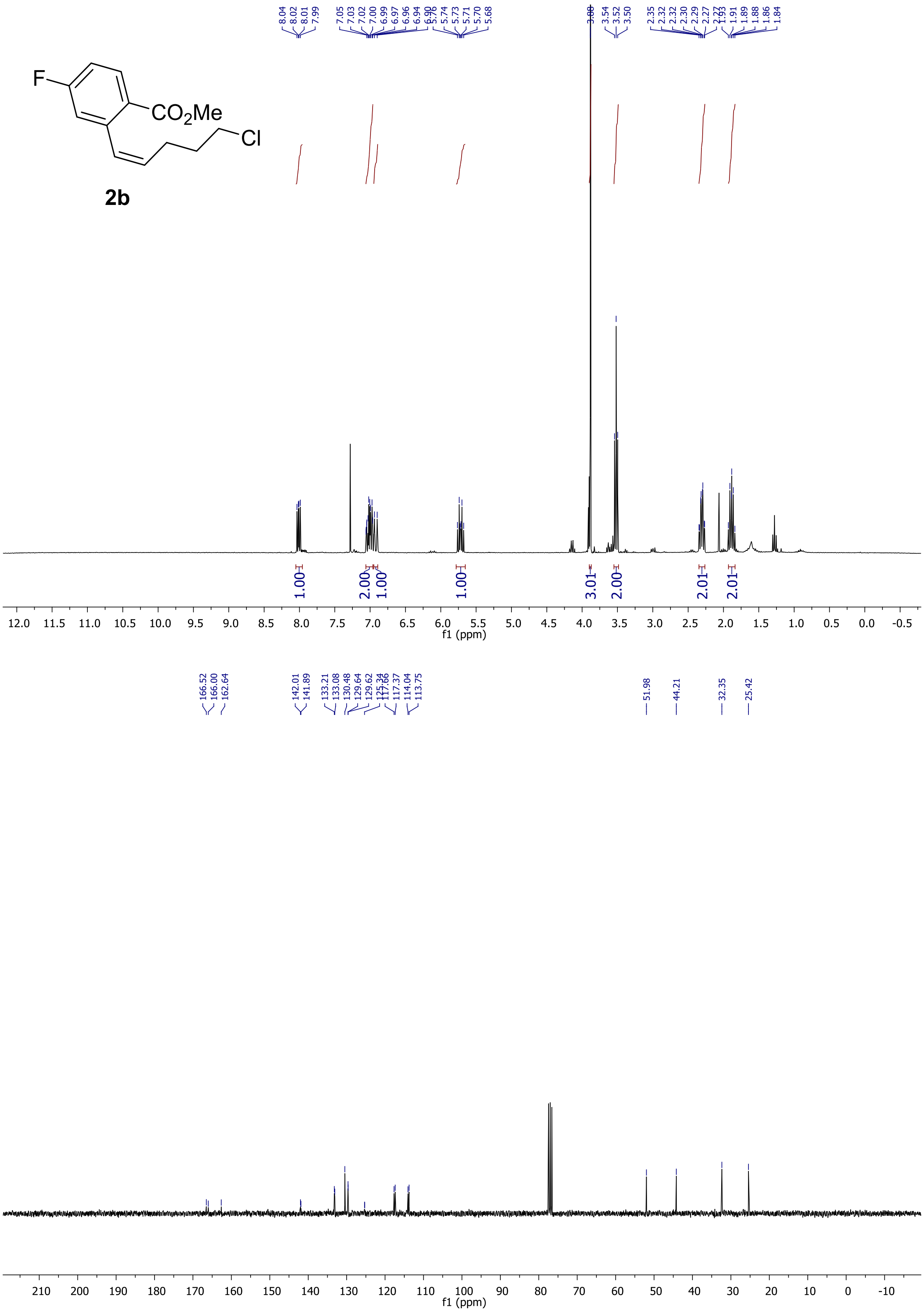

Figure SI-8. ^{1}H-NMR (300 MHz, $CDCl_3$) and ^{13}C {^{1}H} NMR (75 MHz, $CDCl_3$) spectra of compound **2b**

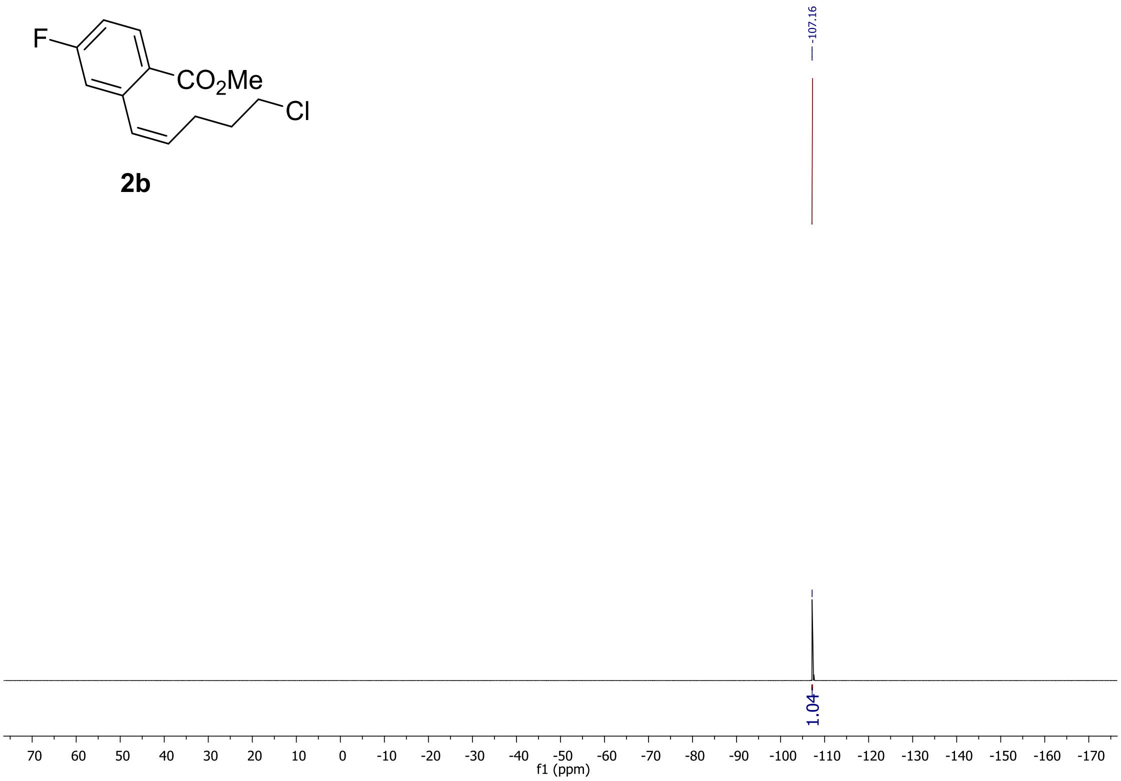

Figure SI-9. ^{19}F NMR (282 MHz, $CDCl_3$) spectrum of compound **2b**

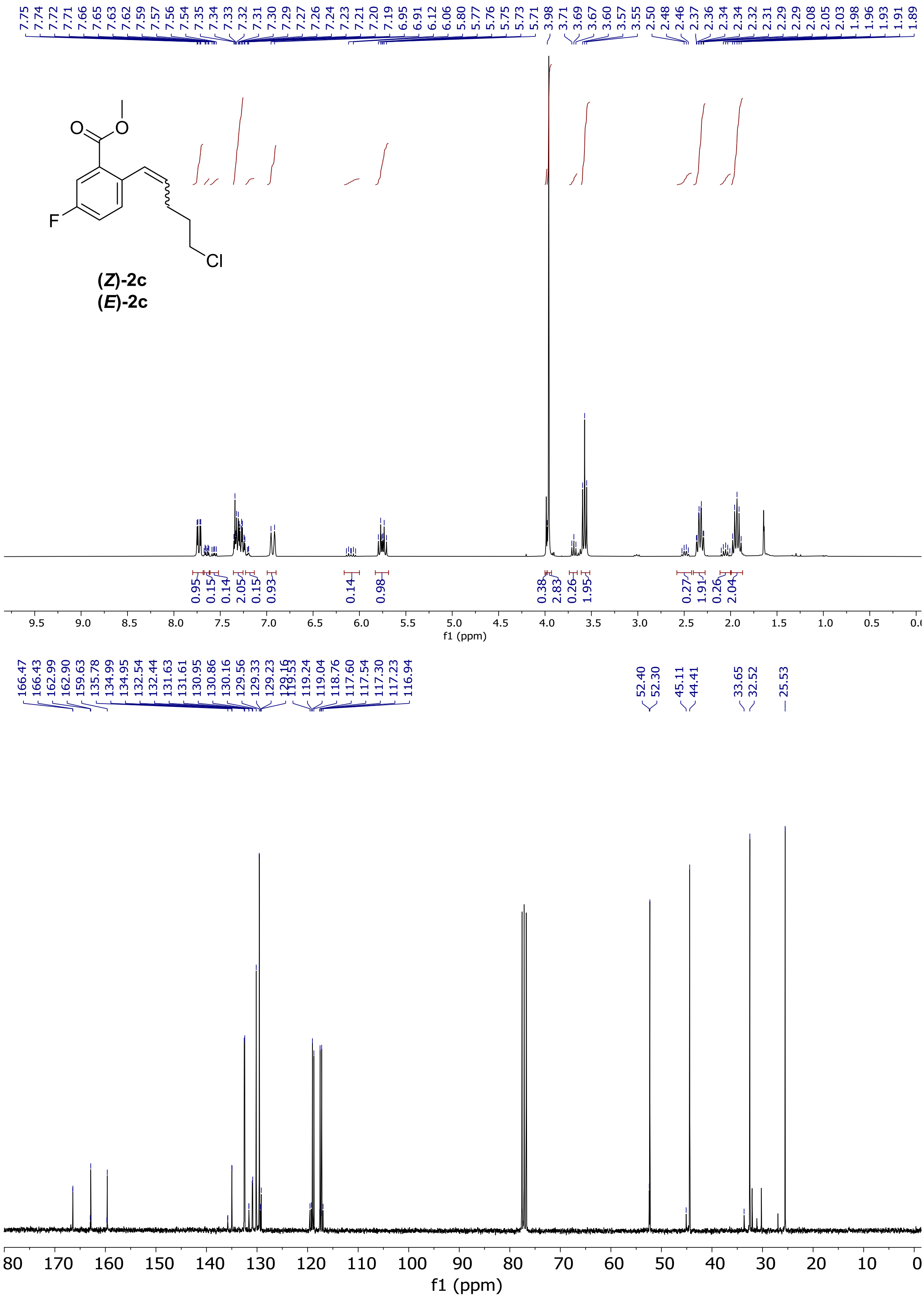

Figure SI-10. ^{1}H-NMR (300 MHz, $CDCl_3$) and ^{13}C {^{1}H} NMR (75 MHz, $CDCl_3$) spectra of compound **2c**

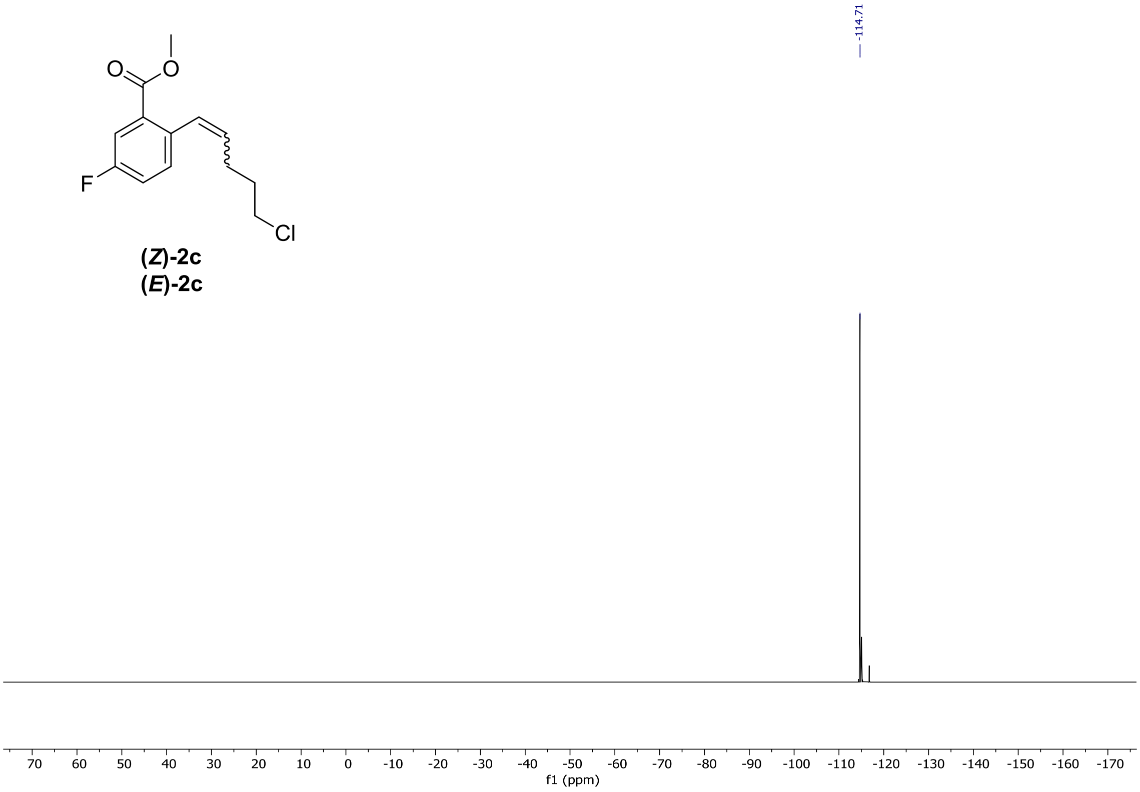

Figure SI-11. ^{19}F NMR (282 MHz, $CDCl_3$) spectrum of compound **2c**

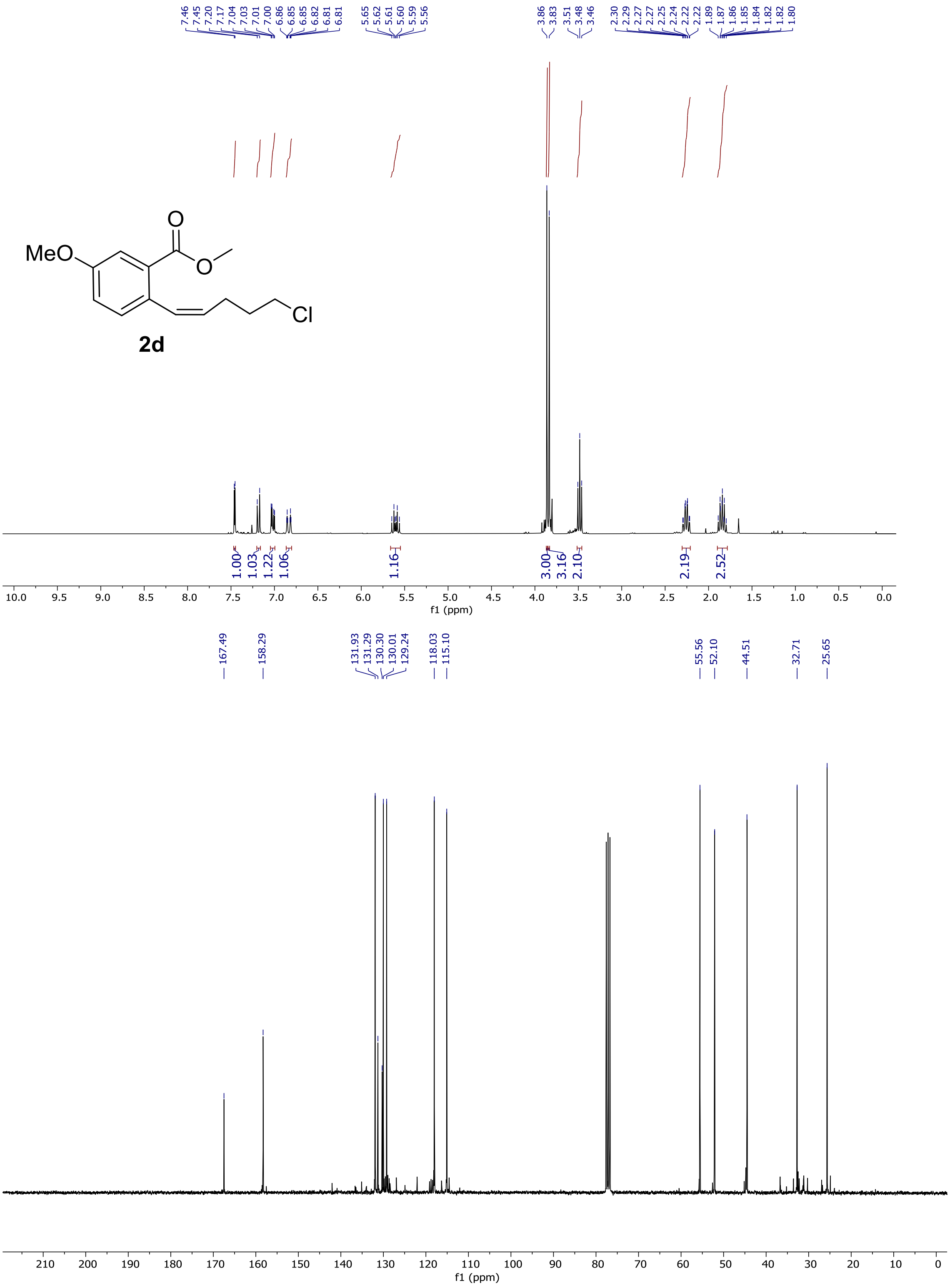

Figure SI-12. ^{1}H-NMR (300 MHz, $CDCl_3$) and ^{13}C {^{1}H} NMR (75 MHz, $CDCl_3$) spectra of compound **2d**

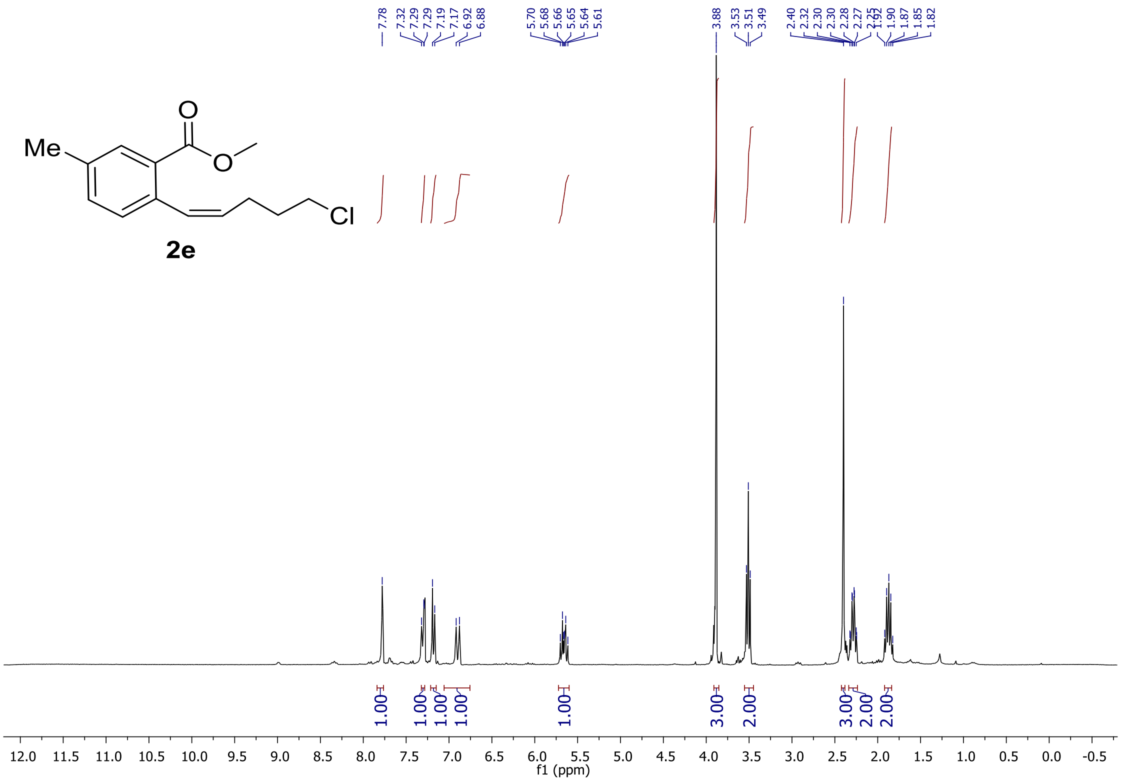

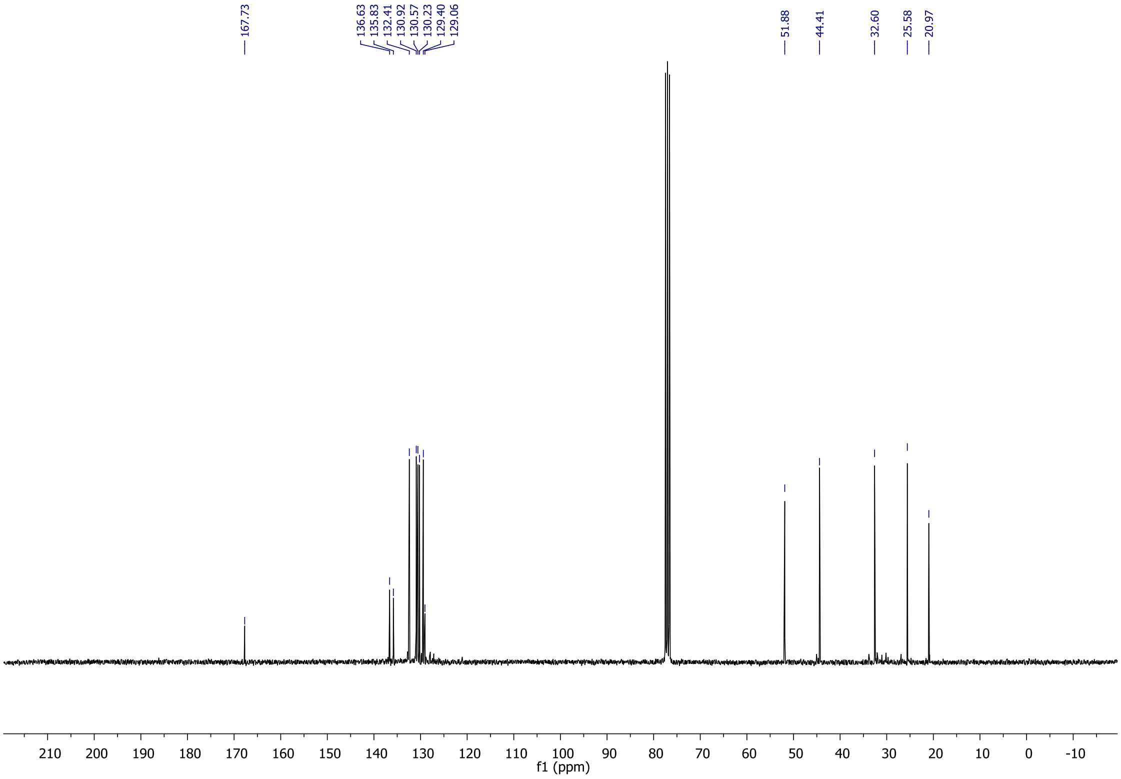

Figure SI-13. ^{1}H-NMR (300 MHz, $CDCl_3$) and ^{13}C {^{1}H} NMR (75 MHz, $CDCl_3$) spectra of compound **2e**

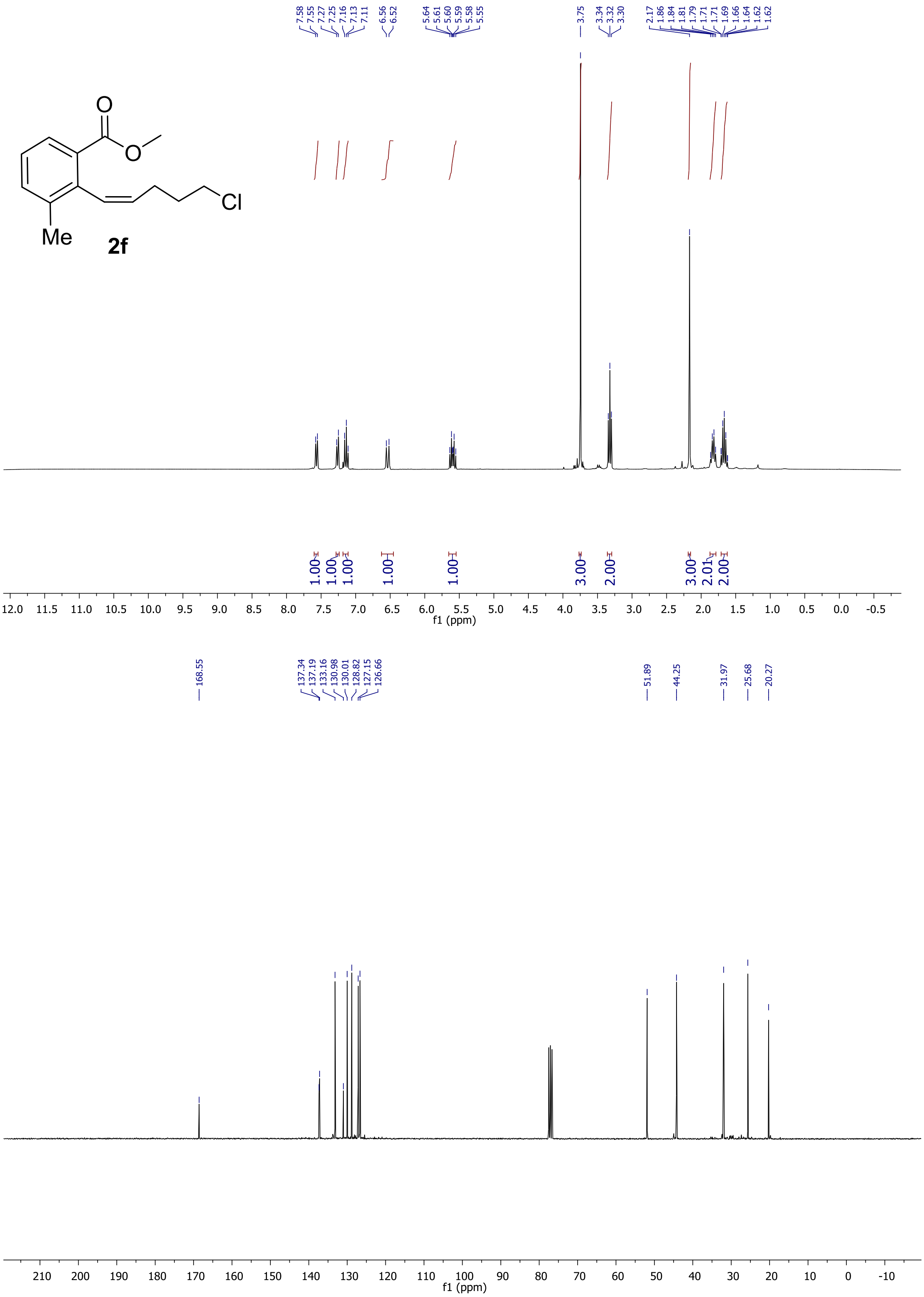

Figure SI-14. ^{1}H-NMR (300 MHz, $CDCl_3$) and ^{13}C {^{1}H} NMR (75 MHz, $CDCl_3$) spectra of compound **2f**

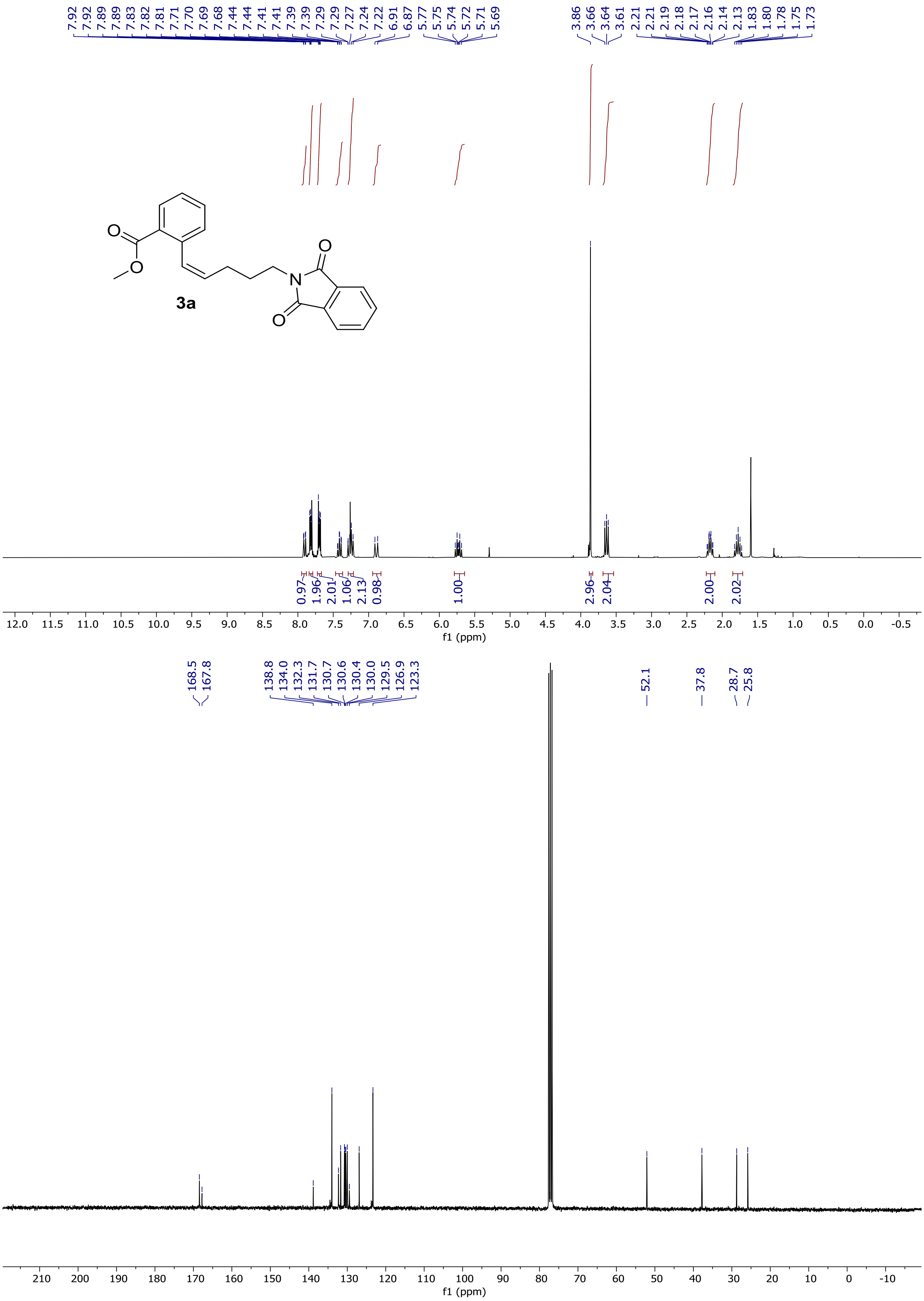

Figure SI-15. ^{1}H-NMR (300 MHz, $CDCl_3$) and ^{13}C {^{1}H} NMR (75 MHz, $CDCl_3$) spectra of compound **3a**

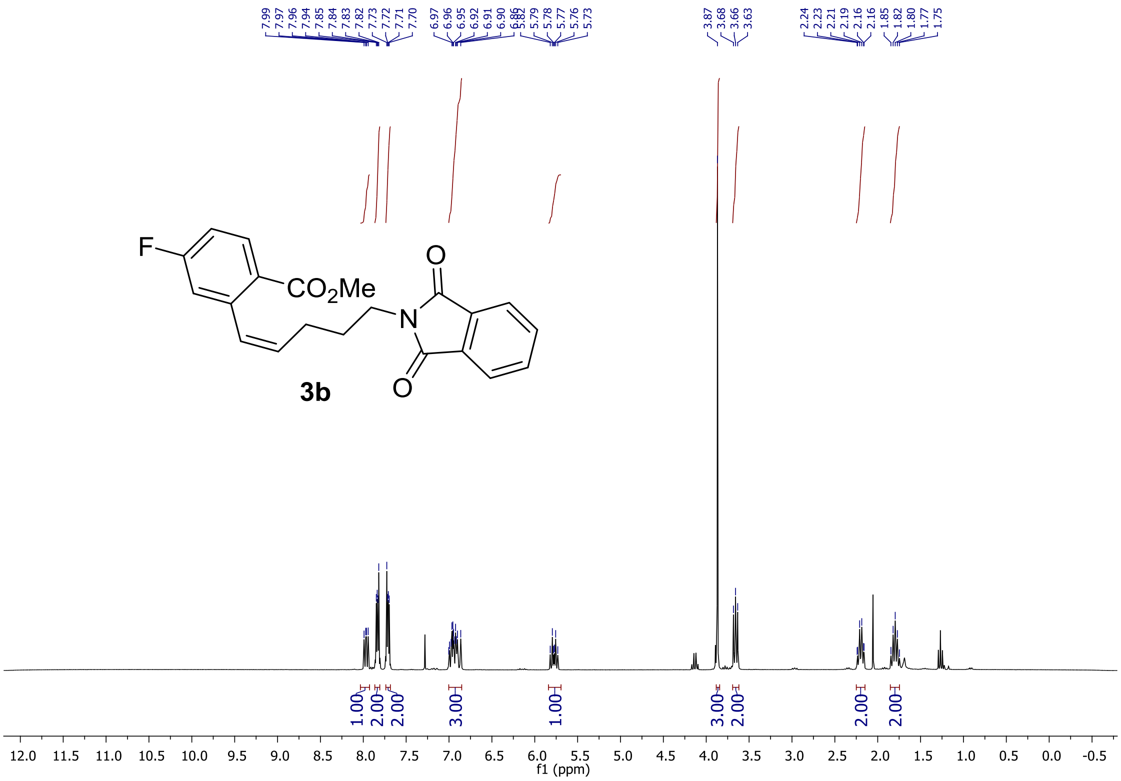

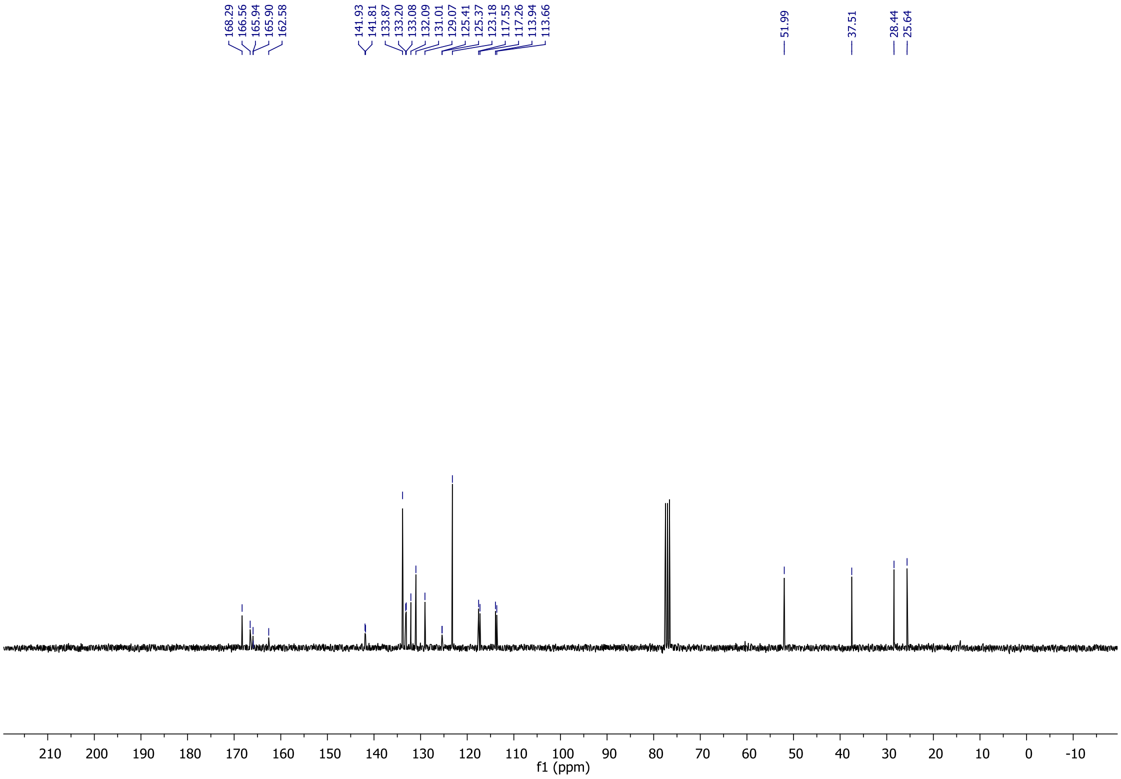

Figure SI-16. ^{1}H-NMR (300 MHz, $CDCl_3$) and ^{13}C {^{1}H} NMR (75 MHz, $CDCl_3$) spectra of compound **3b**

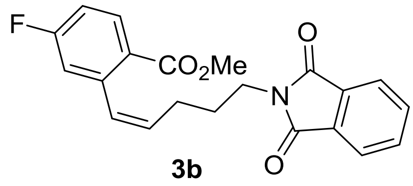

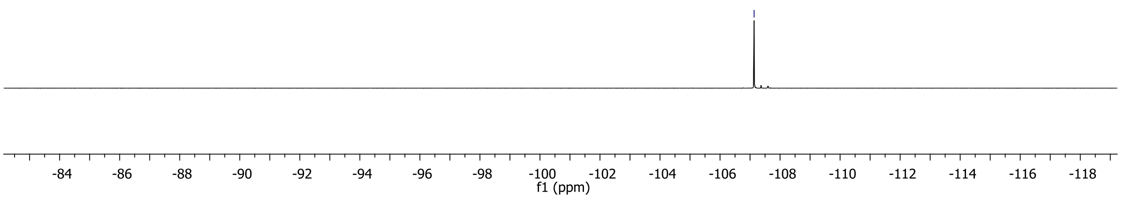

Figure SI-17. ^{19}F NMR **(**282 MHz, $CDCl_3$) spectrum of compound **3b**

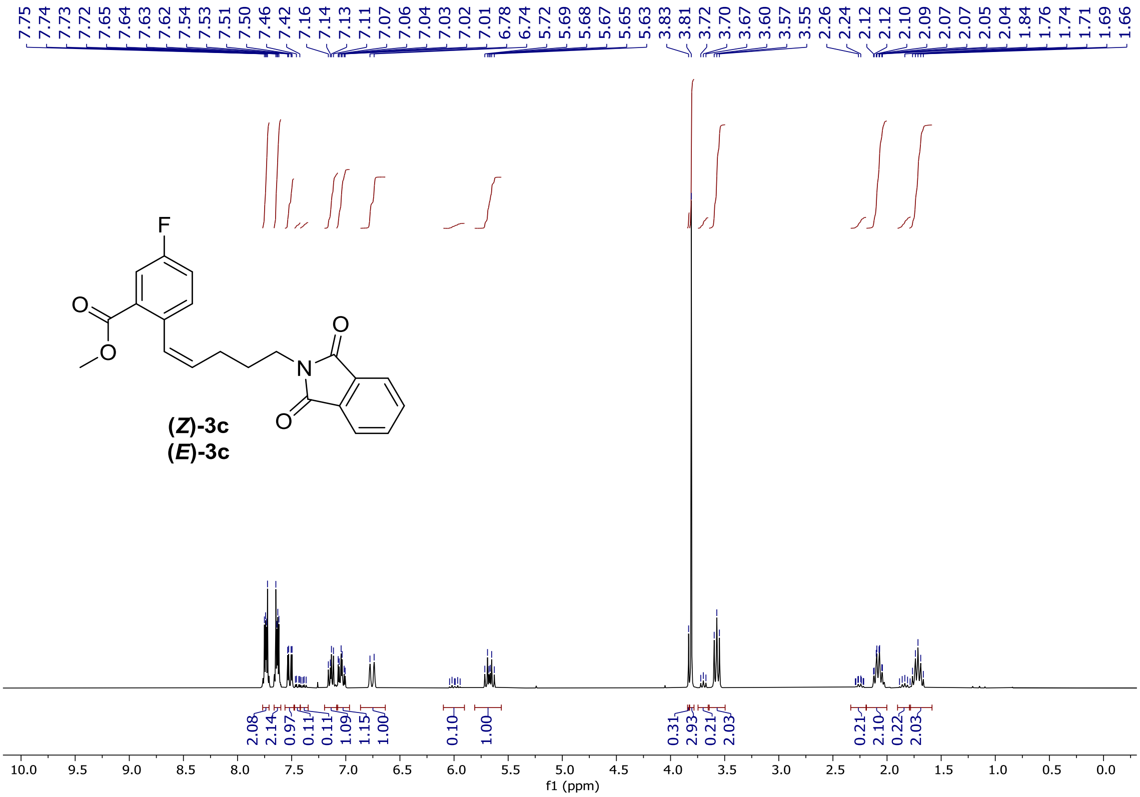

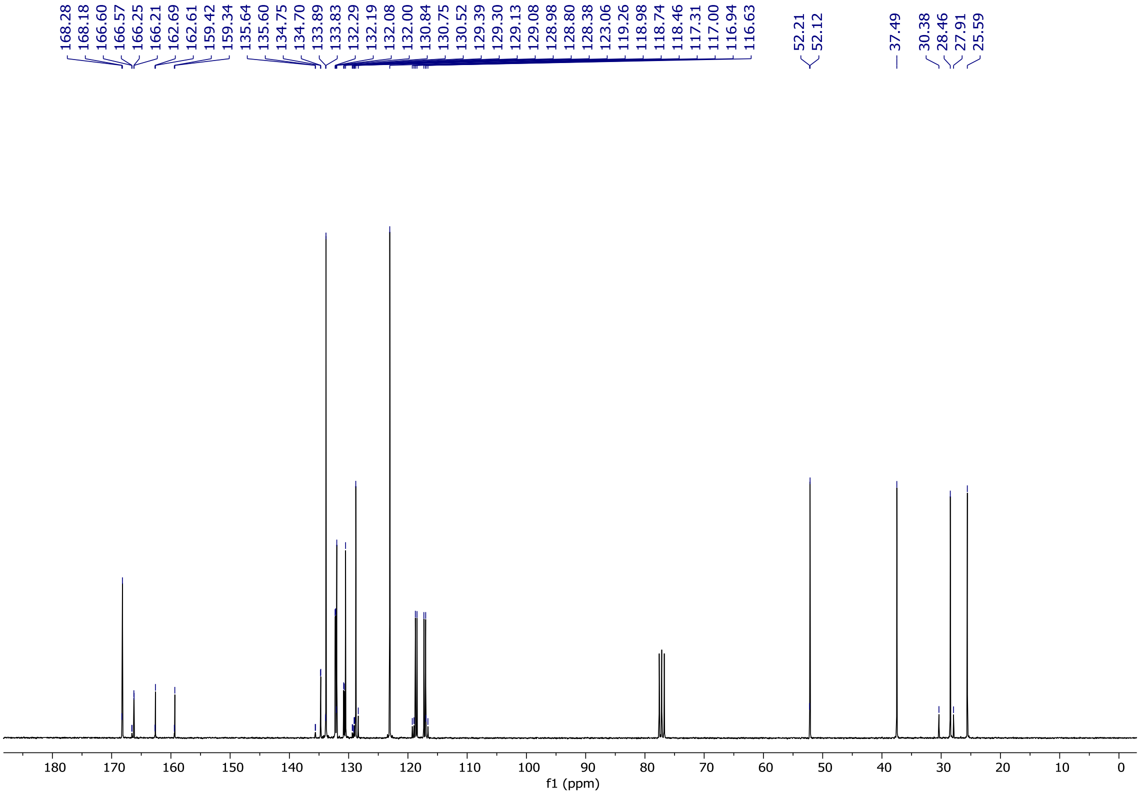

Figure SI-18. ^{1}H-NMR (300 MHz, $CDCl_3$) and ^{13}C {^{1}H} NMR (75 MHz, $CDCl_3$) spectra of compound **3c**

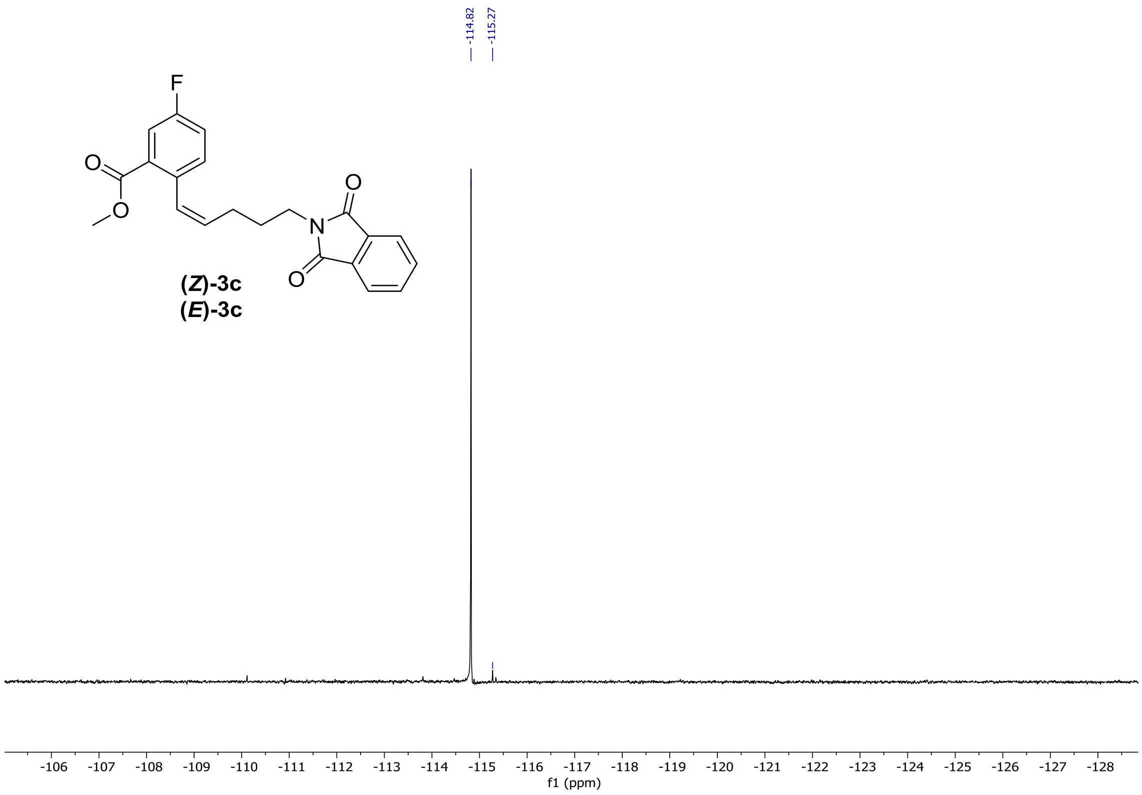

Figure SI-19. ^{19}F NMR (282 MHz, $CDCl_3$) spectrum of compound **3c**

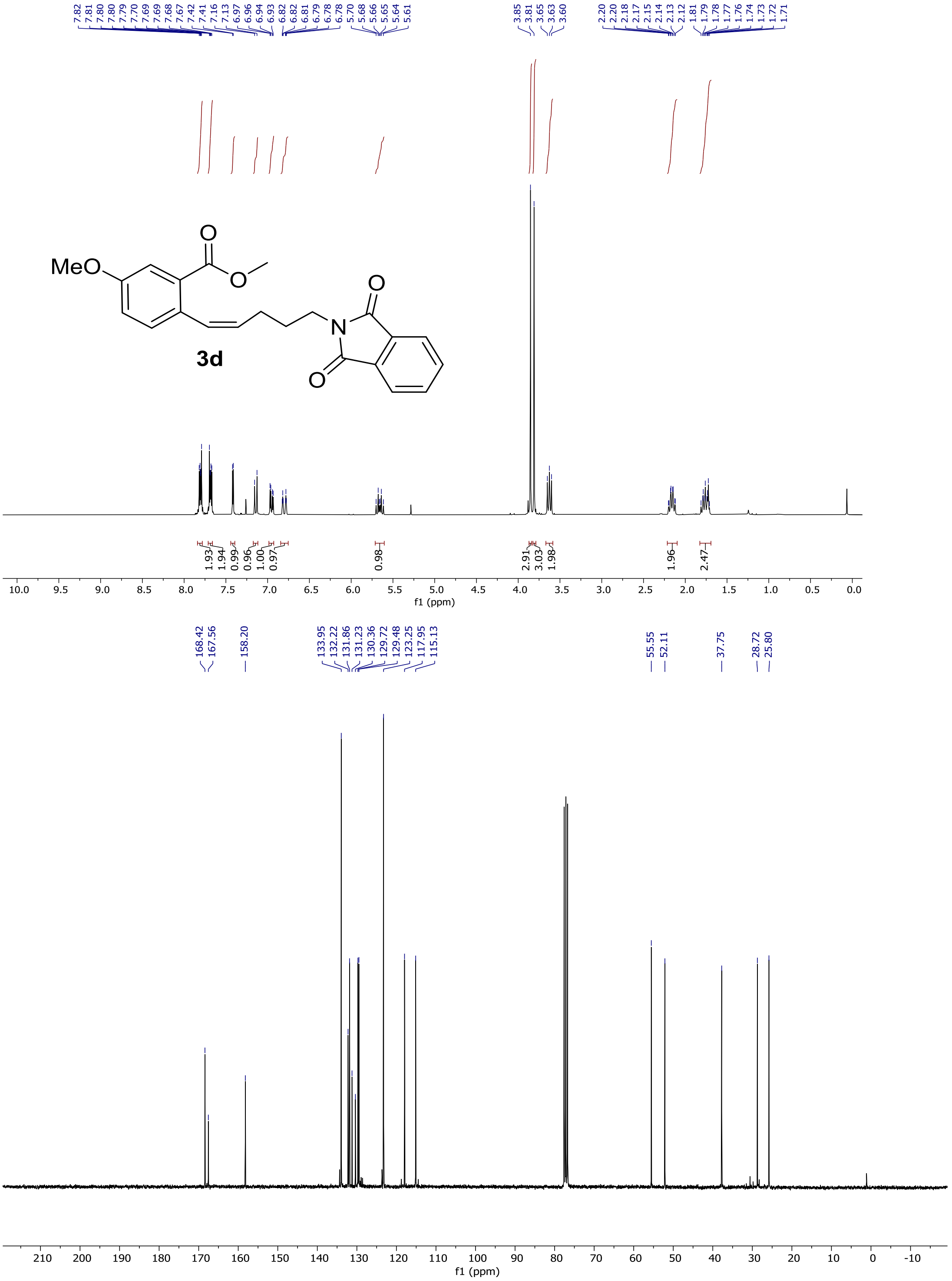

Figure SI-20. ^{1}H-NMR (300 MHz, $CDCl_3$) and ^{13}C {^{1}H} NMR (75 MHz, $CDCl_3$) spectra of compound **3d**

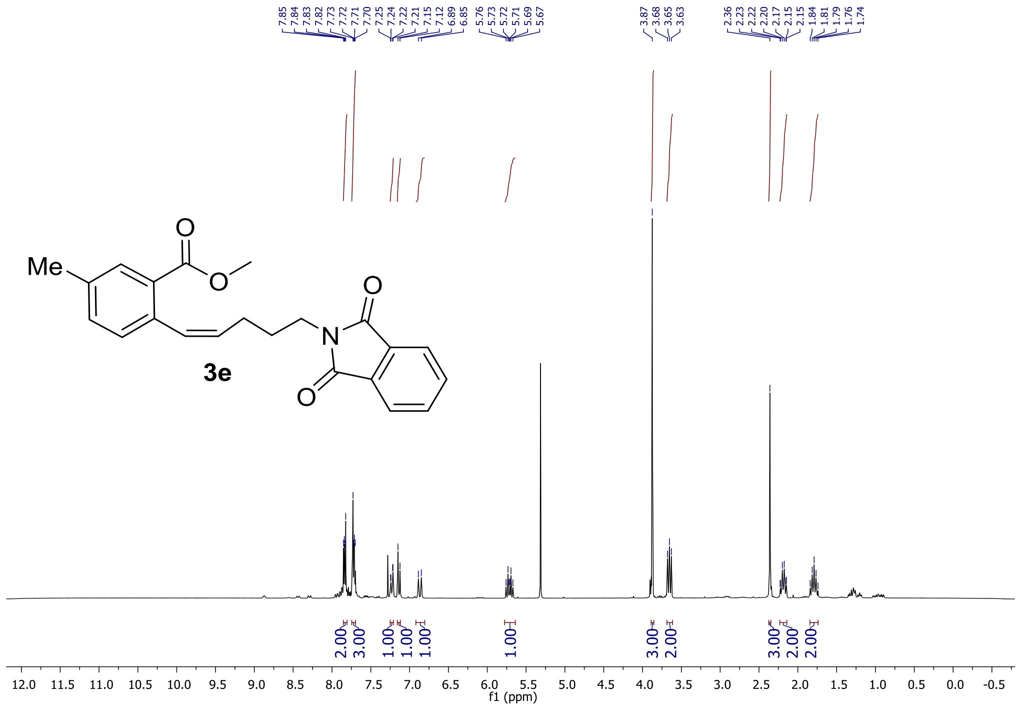

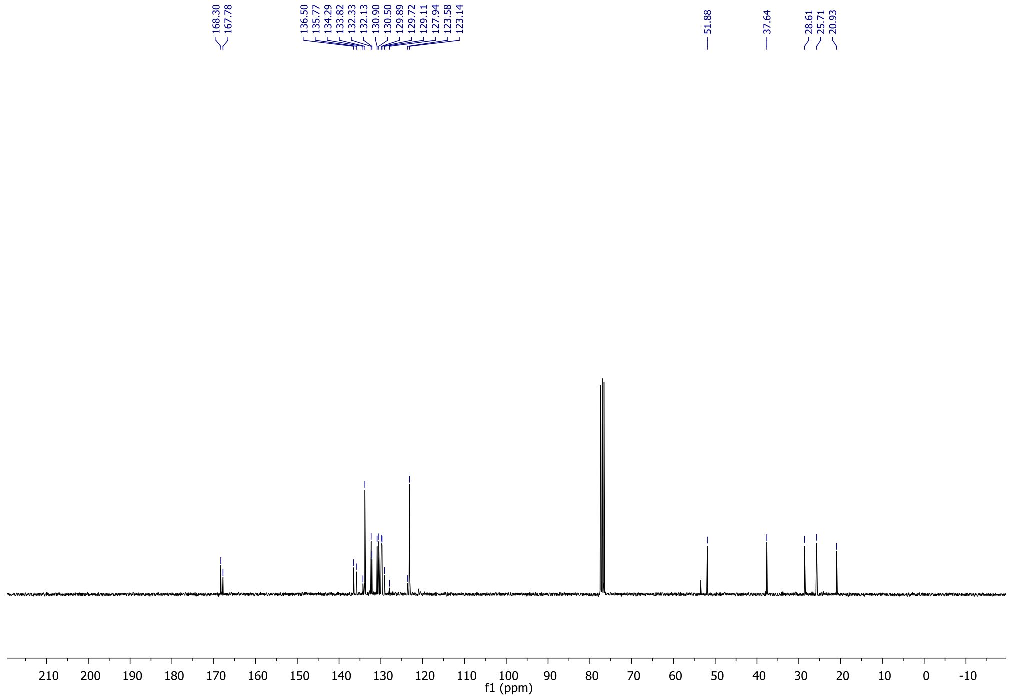

Figure SI-21. ^{1}H-NMR (300 MHz, CDCl$_3$) and ^{13}C {^{1}H} NMR (75 MHz, CDCl$_3$) spectra of compound **3e**

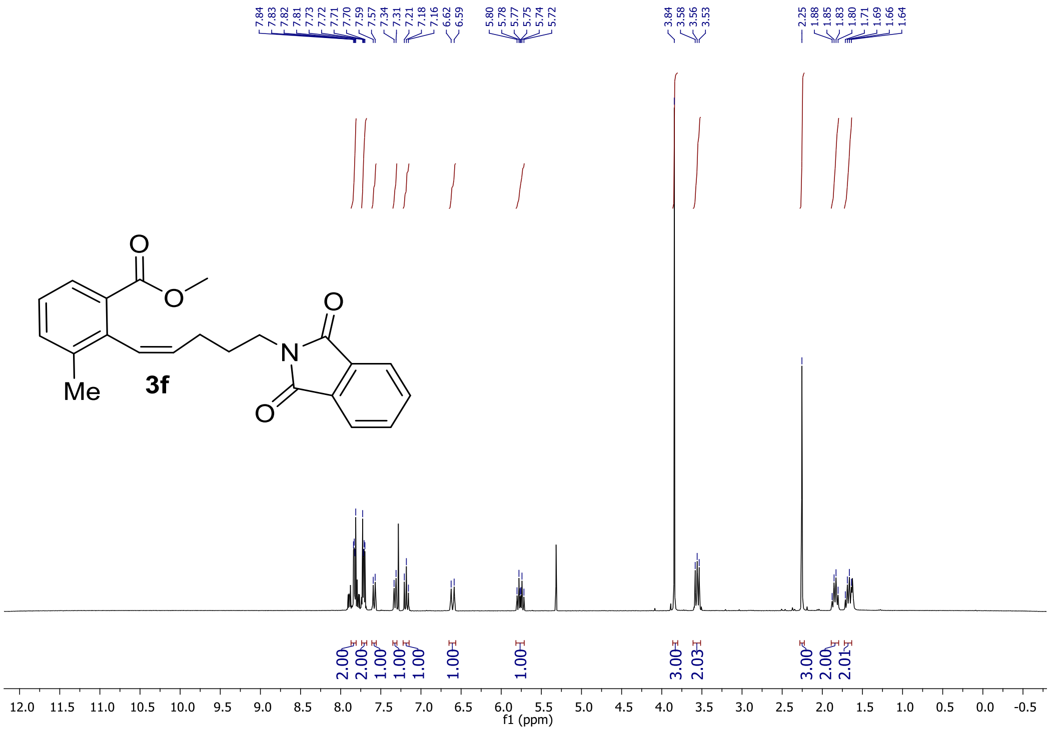

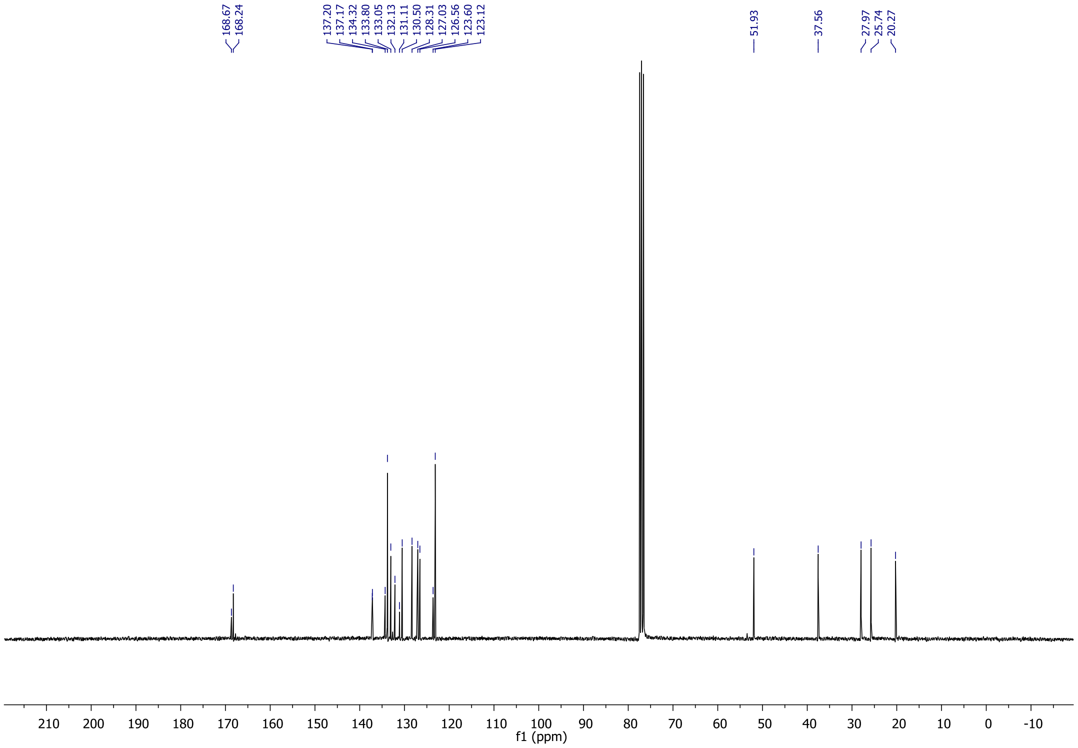

Figure SI-22. ^{1}H-NMR (300 MHz, $CDCl_3$) and ^{13}C {^{1}H} NMR (75 MHz, $CDCl_3$) spectra of compound **3f**

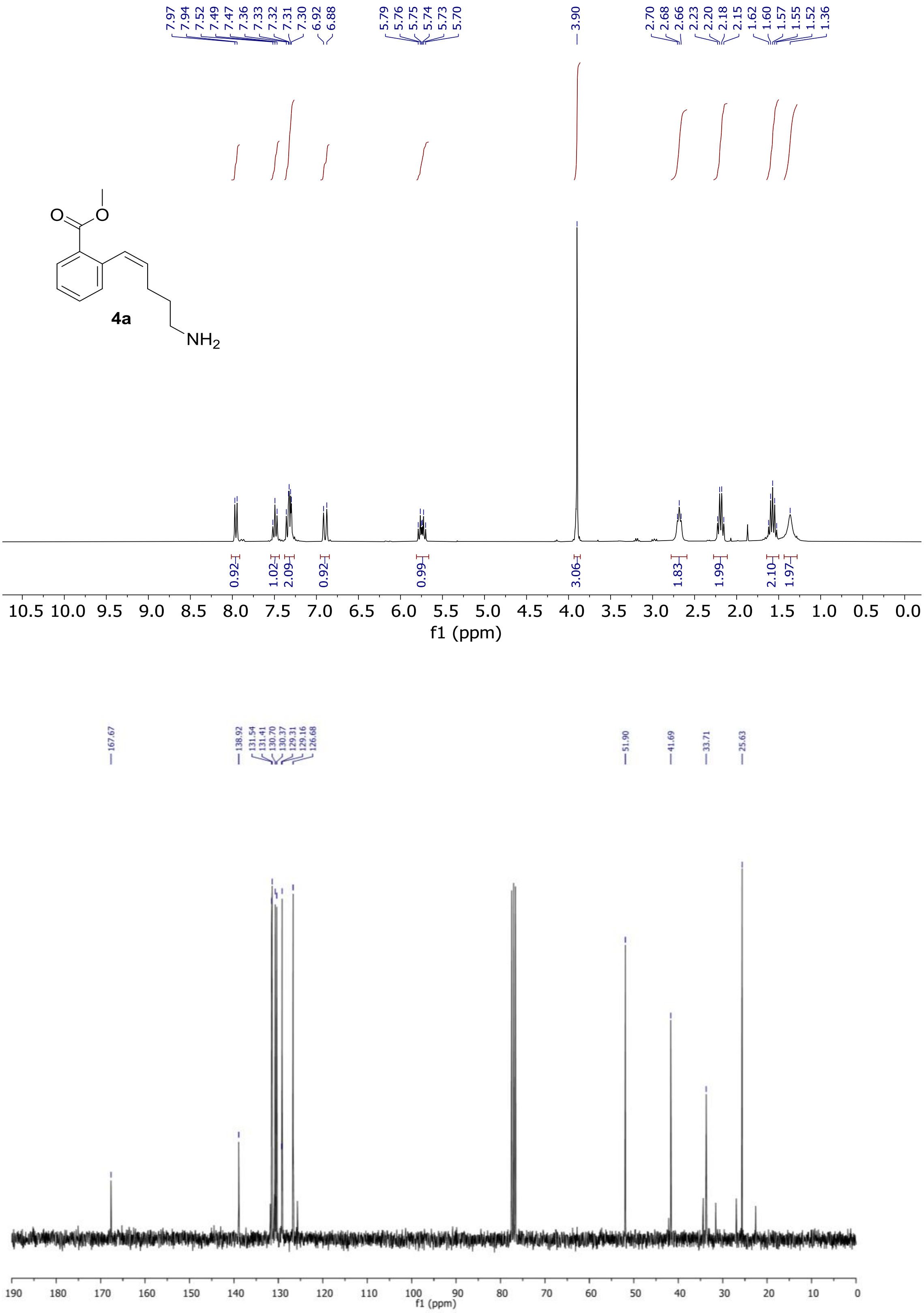

Figure SI-23. ^{1}H-NMR (300 MHz, $CDCl_3$) and $^{13}C\{^{1}H\}$ NMR (75 MHz, $CDCl_3$) spectra of compound **4a**

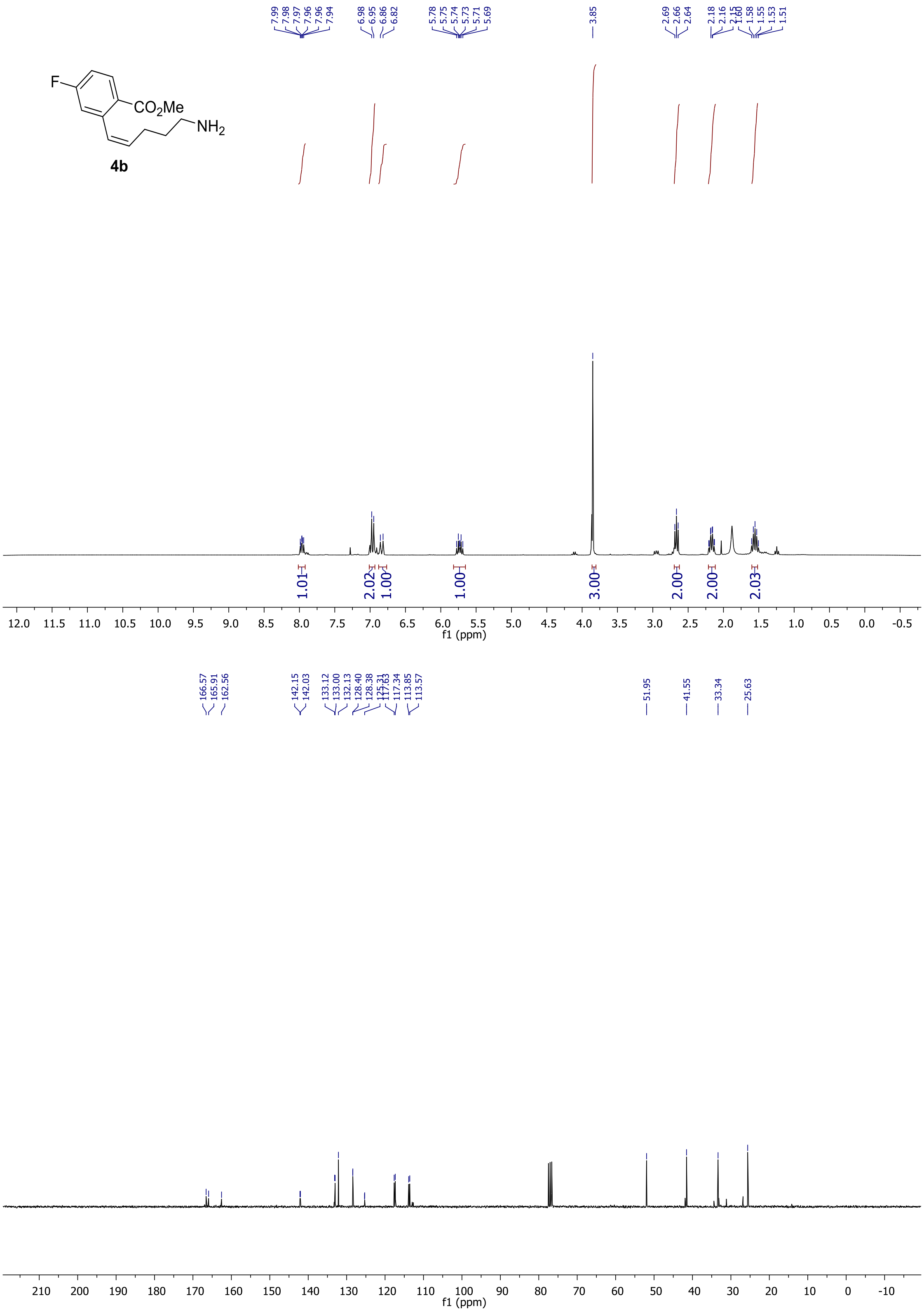

Figure SI-24. ^{1}H-NMR (300 MHz, $CDCl_3$) and ^{13}C {^{1}H} NMR (75 MHz, $CDCl_3$) spectra of compound **4b**

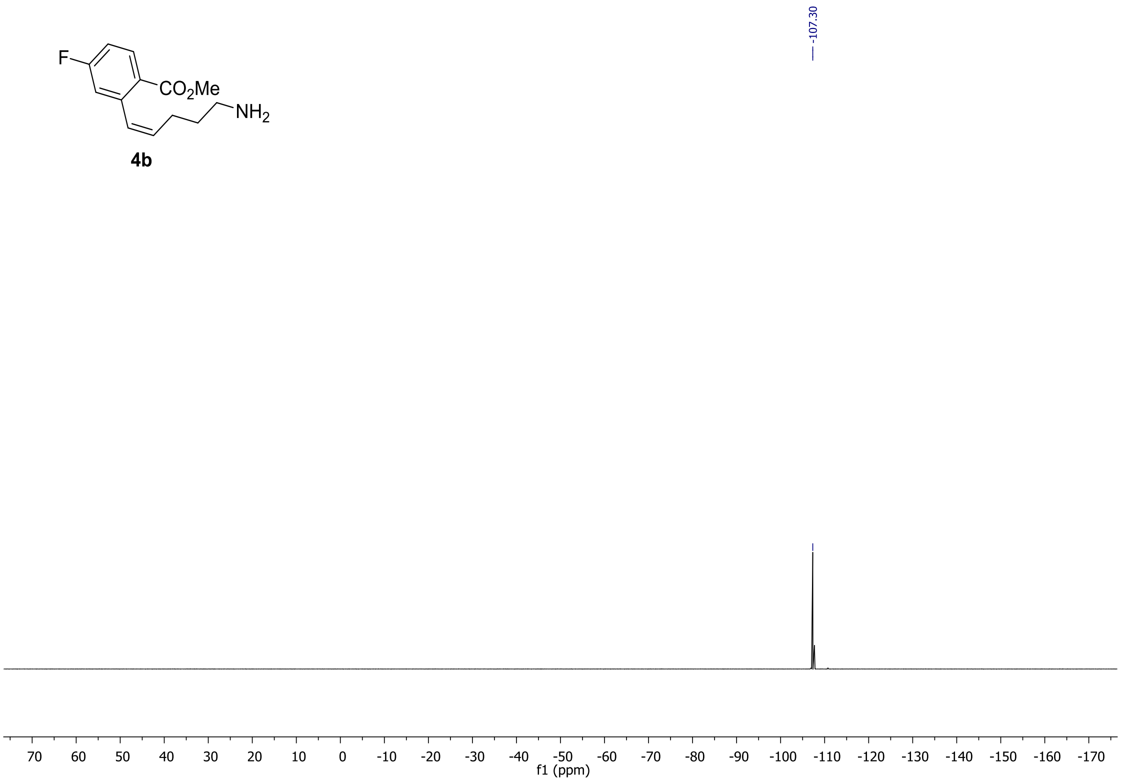

Figure SI-25. ^{19}F NMR (282 MHz, $CDCl_3$) spectrum of compound **4b**

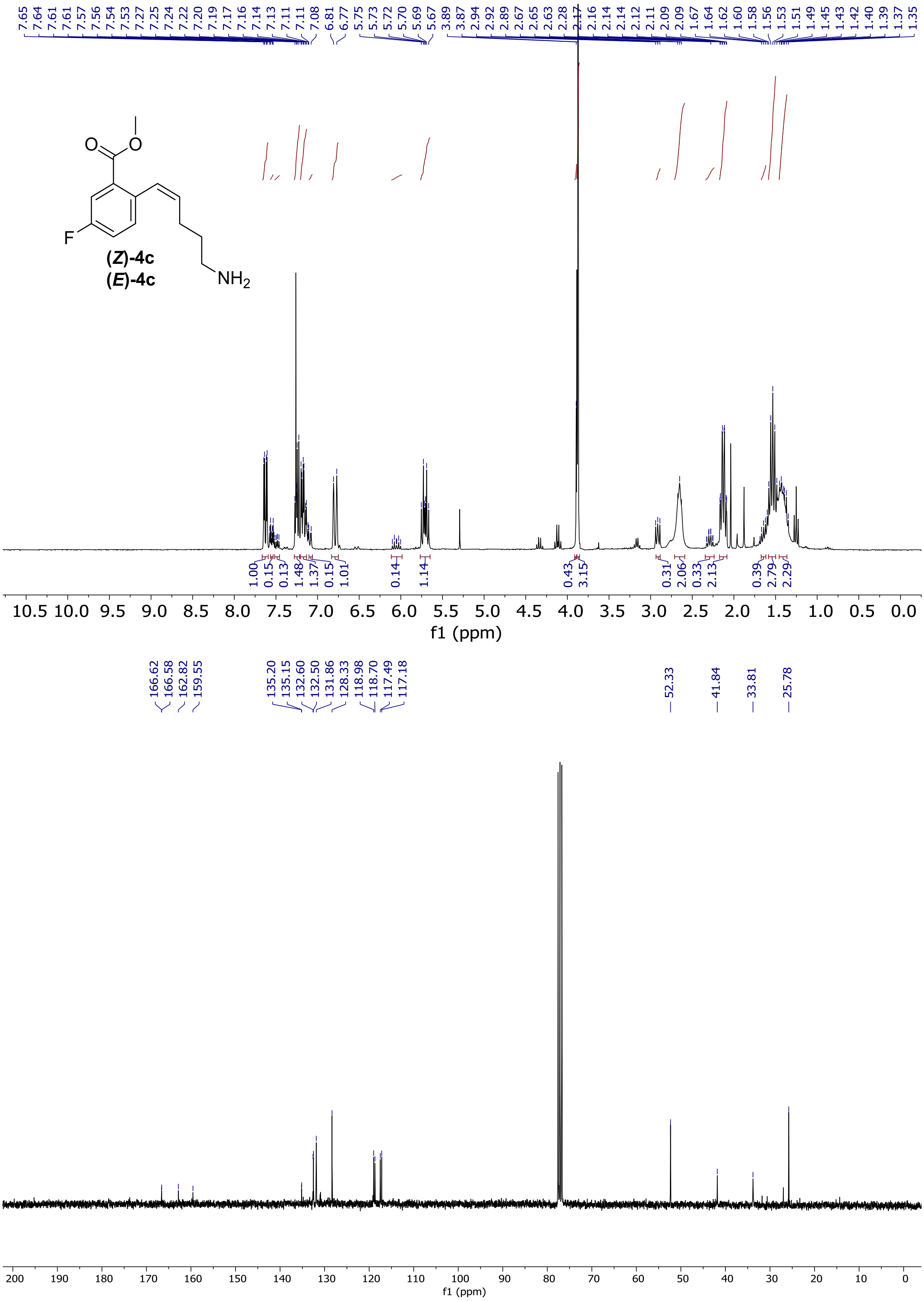

Figure SI-26. ^{1}H-NMR (300 MHz, $CDCl_3$) and ^{13}C {^{1}H} NMR (75 MHz, $CDCl_3$) spectra of compound **4c**

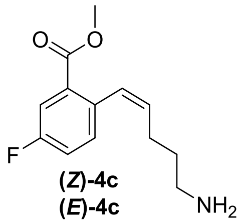

(*Z*)-4c
(*E*)-4c

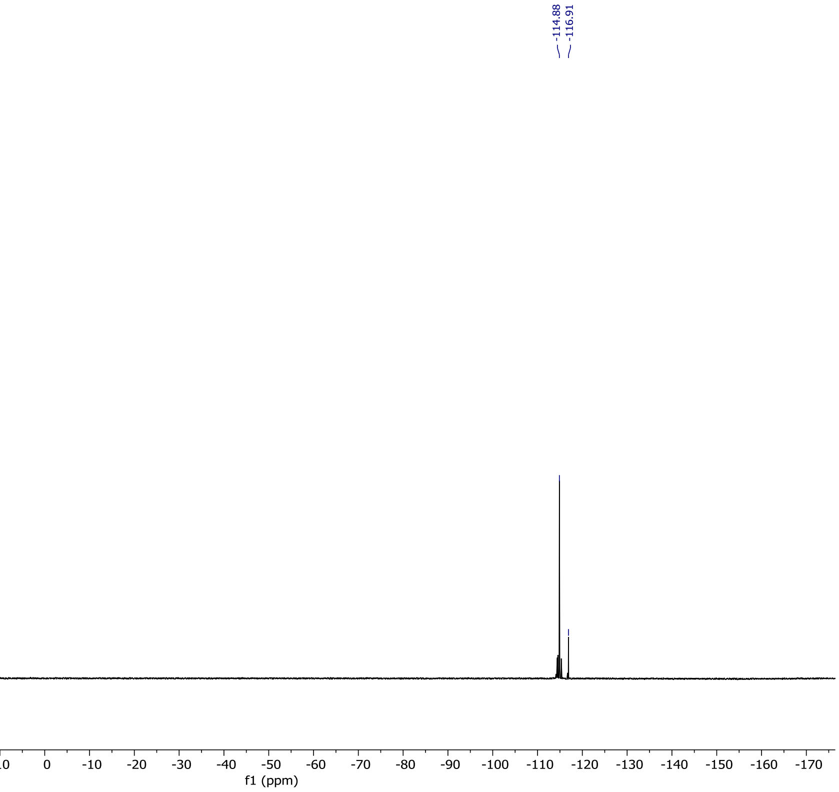

Figure SI-27. ^{19}F NMR (282 MHz, $CDCl_3$) spectrum of compound **4c**

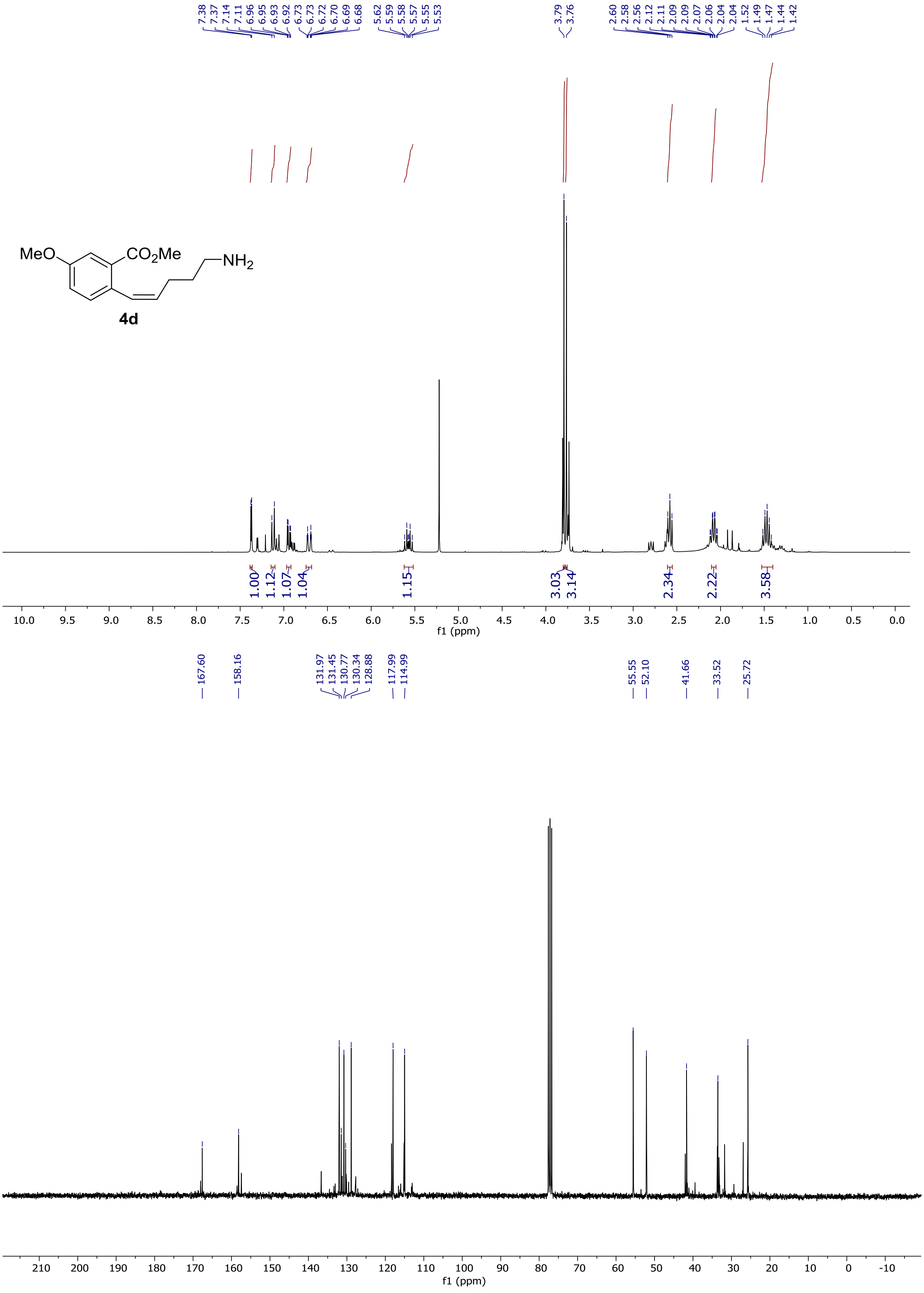

Figure SI-28. ^{1}H-NMR (300 MHz, $CDCl_3$) and ^{13}C {^{1}H} NMR (75 MHz, $CDCl_3$) spectra of compound **4d**

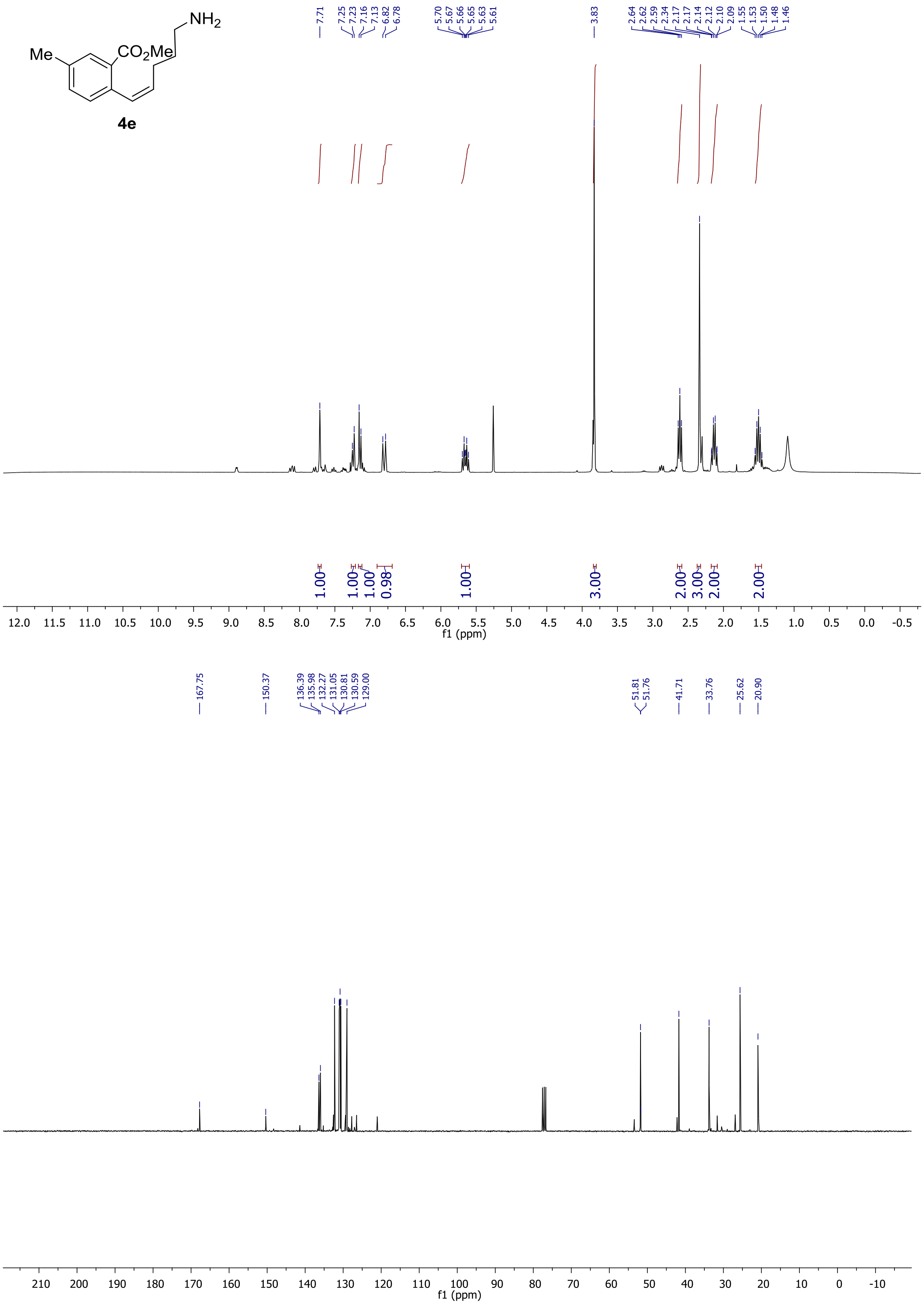

Figure SI-29. ^{1}H-NMR (300 MHz, $CDCl_3$) and ^{13}C {^{1}H} NMR (75 MHz, $CDCl_3$) spectra of compound **4e**

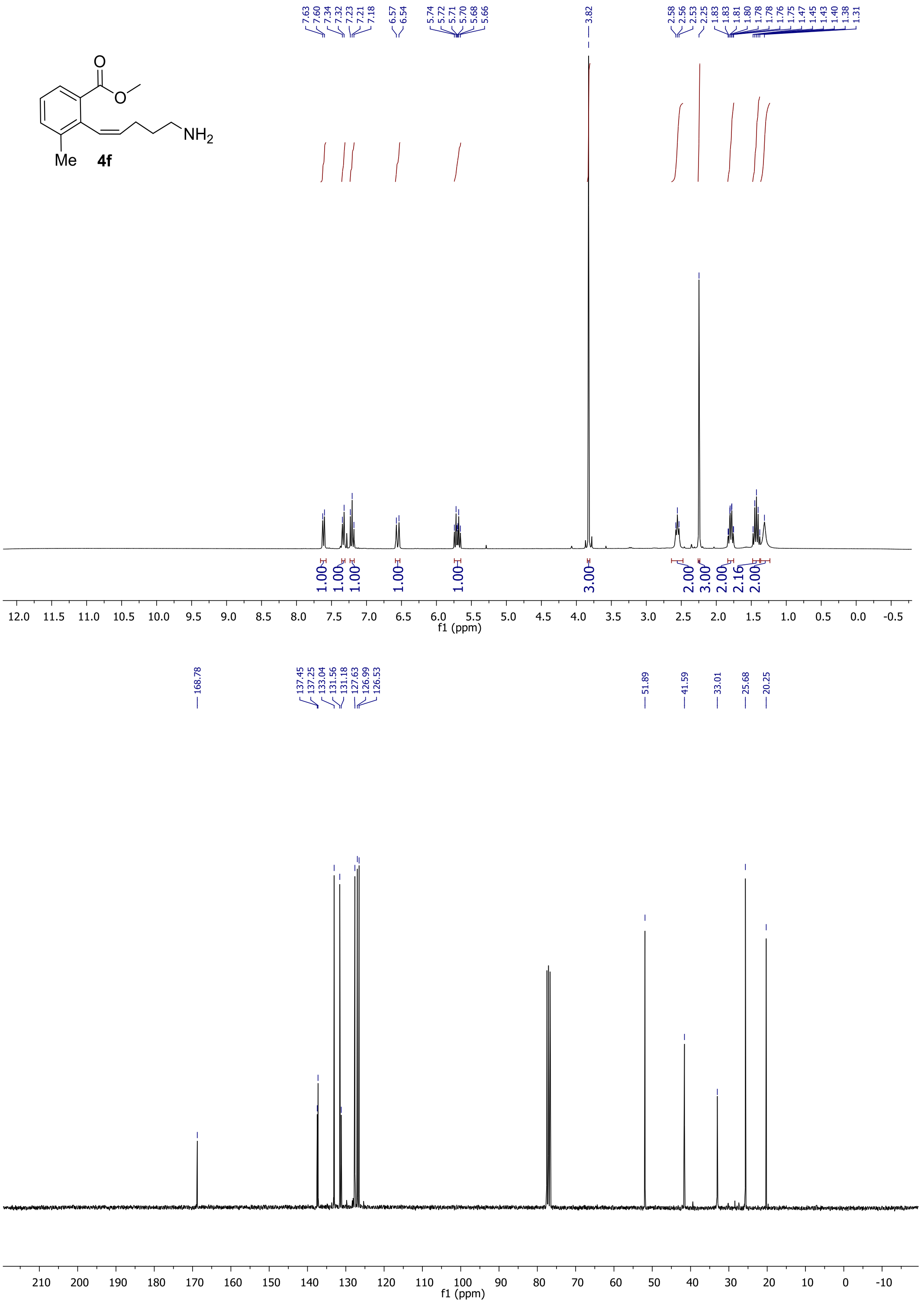

Figure SI-30. ^{1}H-NMR (300 MHz, $CDCl_3$) and ^{13}C {^{1}H} NMR (75 MHz, $CDCl_3$) spectra of compound **4f**

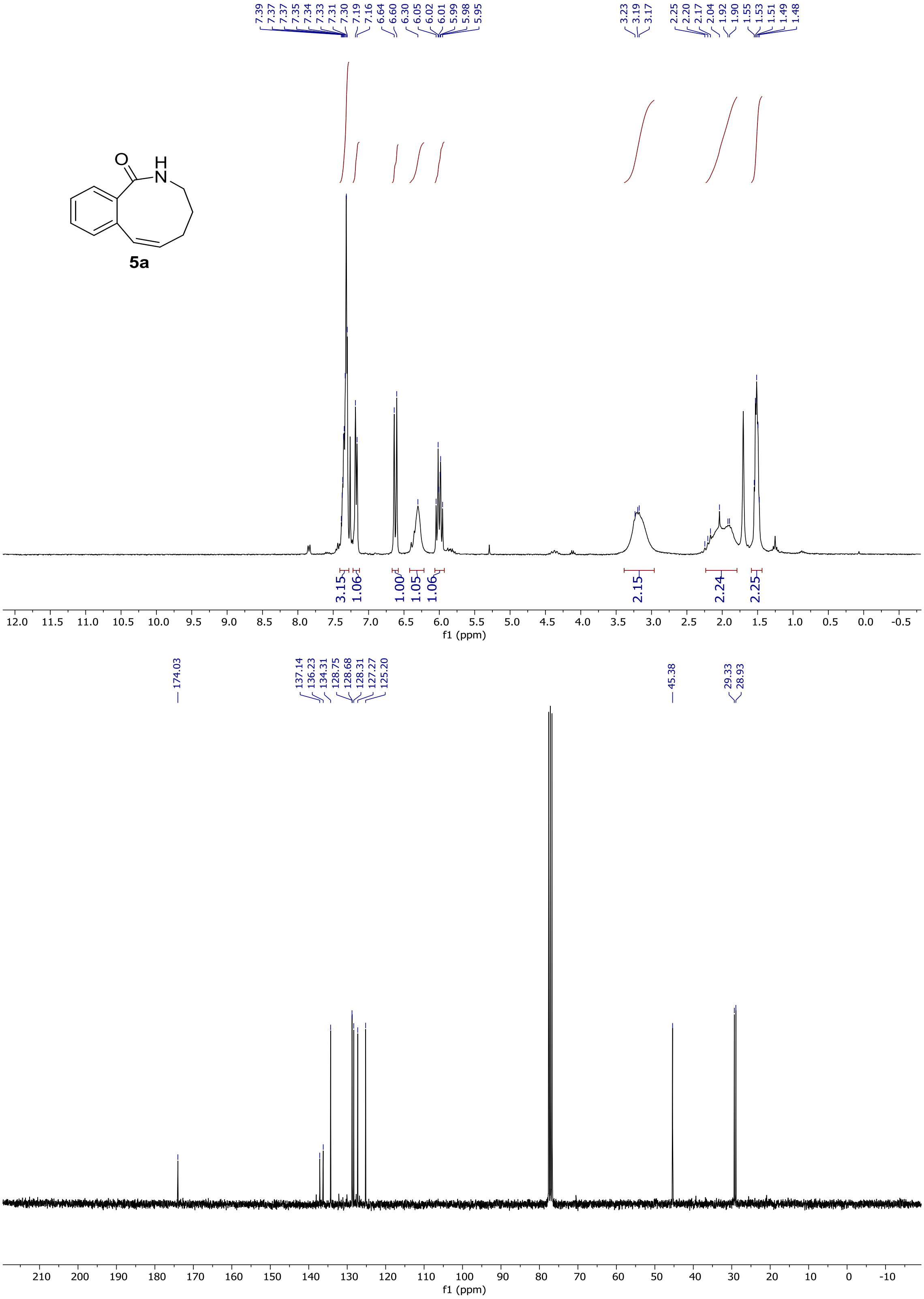

Figure SI-31. ^{1}H-NMR (300 MHz, $CDCl_3$) and ^{13}C {^{1}H} NMR (75 MHz, $CDCl_3$) spectra of compound **5a**

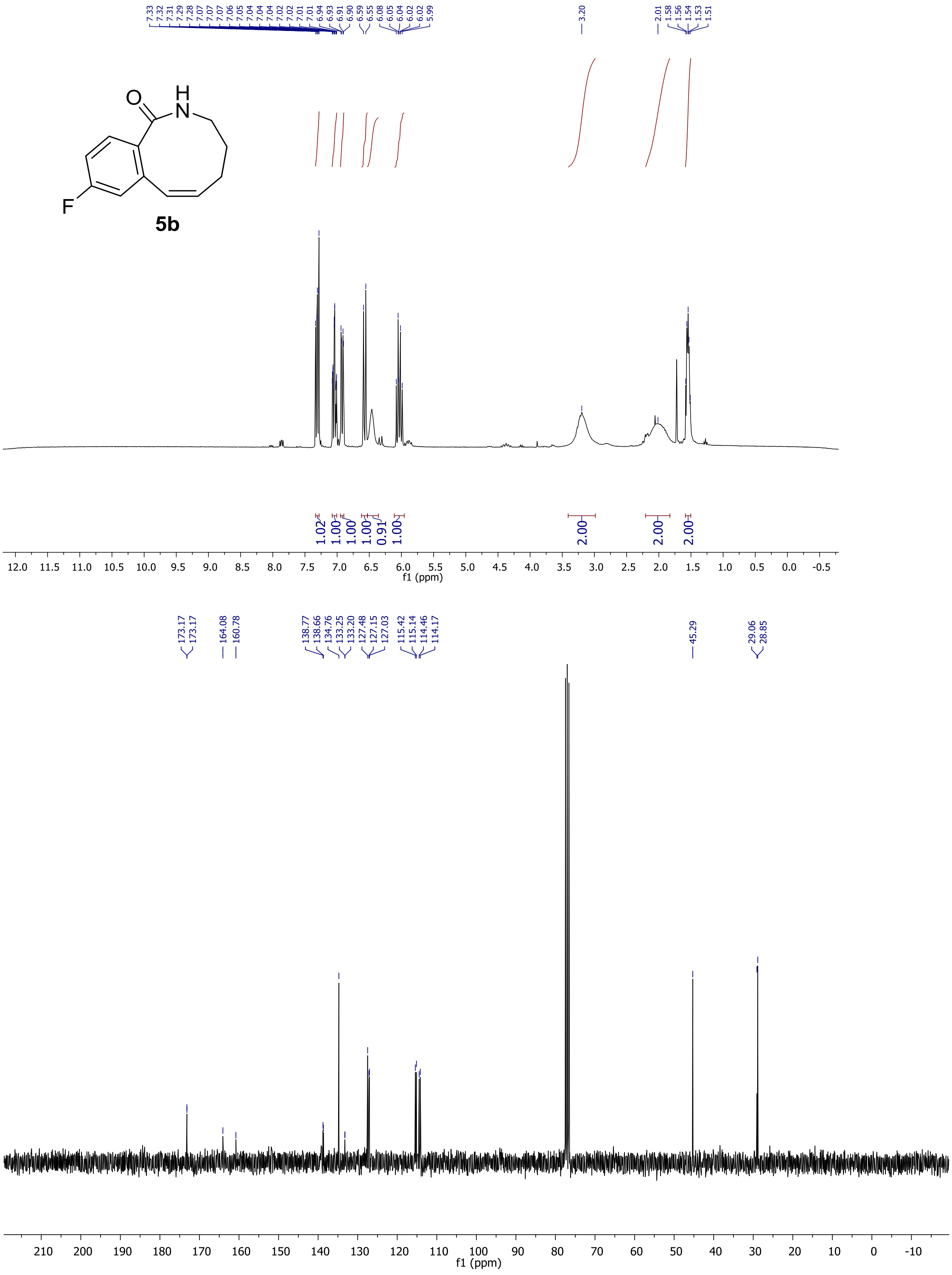

Figure SI-32. ^{1}H-NMR (300 MHz, $CDCl_3$) and $^{13}C\ \{^{1}H\}$ NMR (75 MHz, $CDCl_3$) spectra of compound **5b**

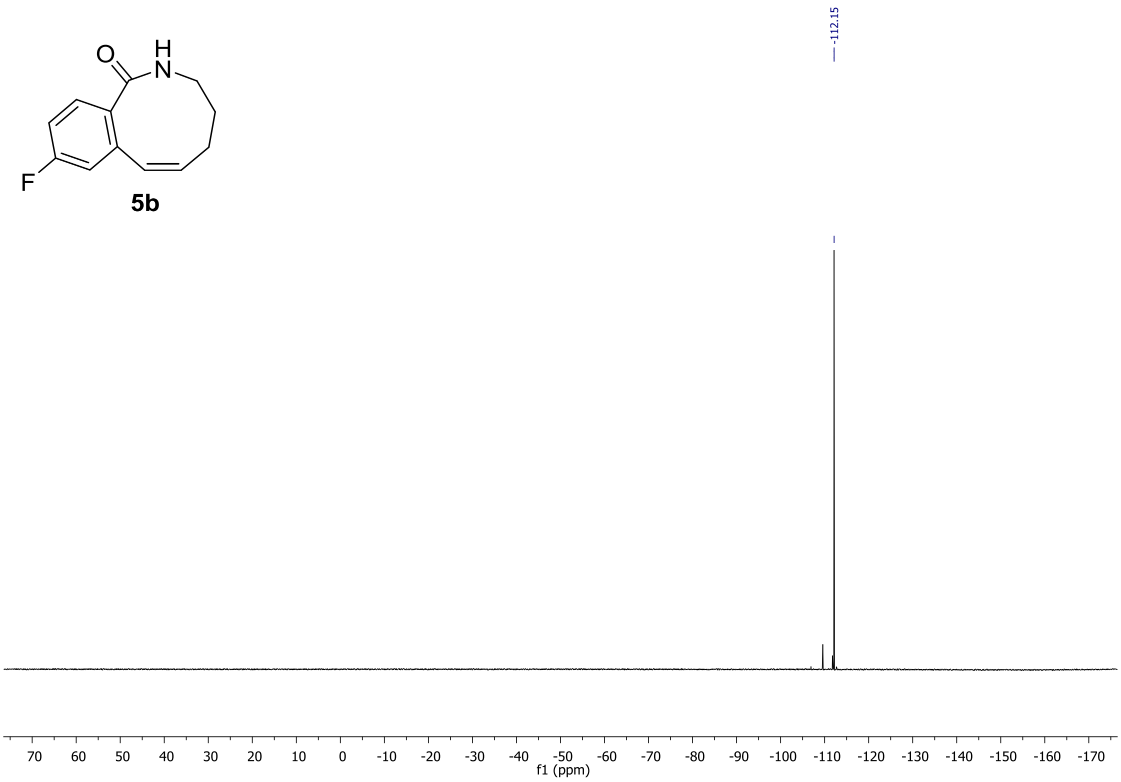

5b

Figure SI-33. ^{19}F NMR (282 MHz, $CDCl_3$) spectrum of compound **5b**

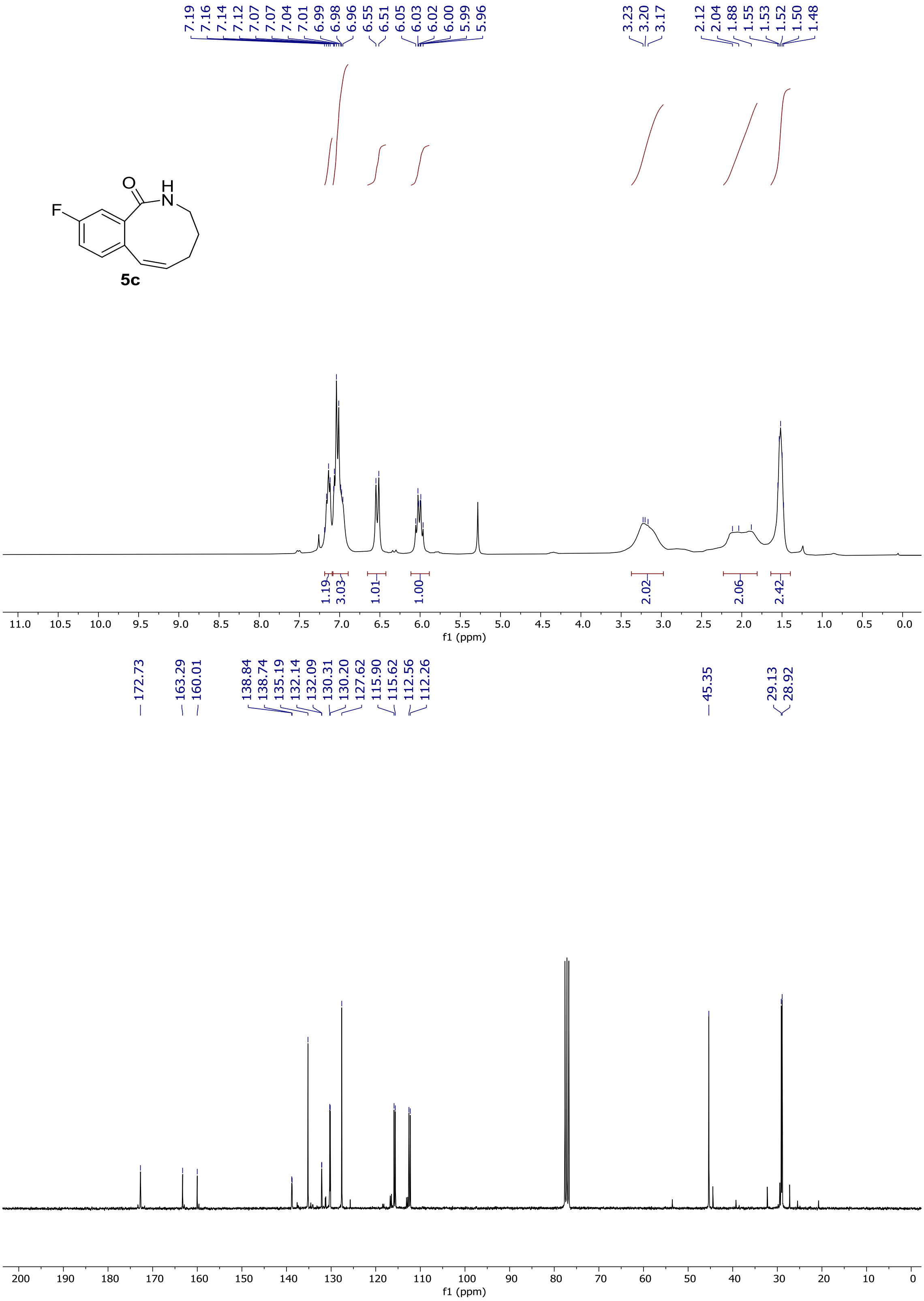

Figure SI-34. ^{1}H-NMR (300 MHz, $CDCl_3$) and ^{13}C {^{1}H} NMR (75 MHz, $CDCl_3$) spectra of compound **5c**

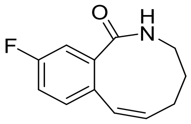

5c

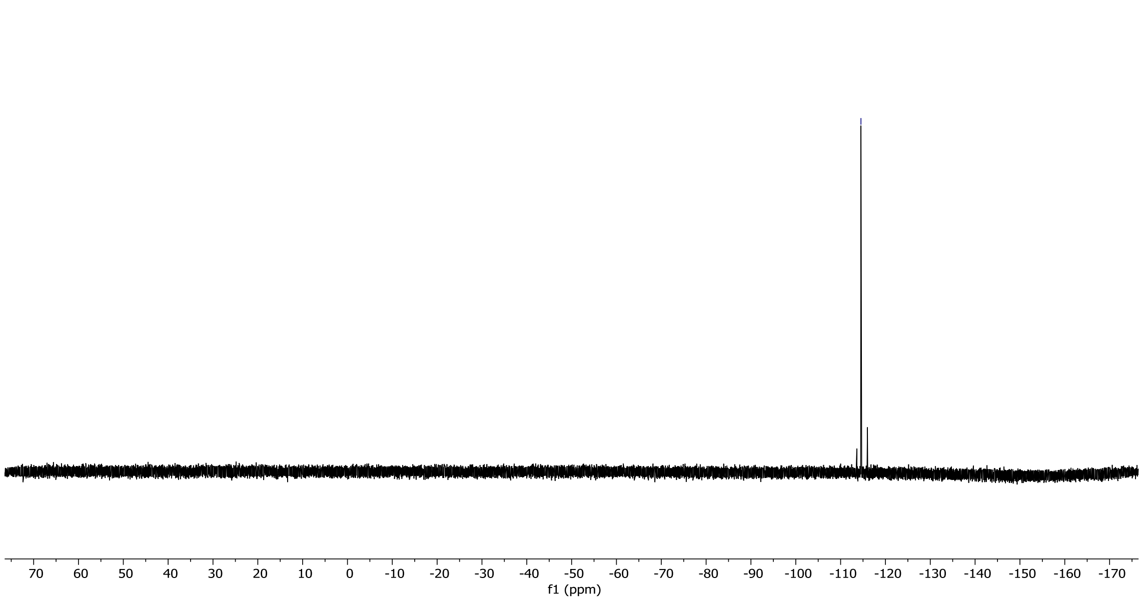

Figure SI-35. ^{19}F NMR (282 MHz, $CDCl_3$) spectrum of compound **5c**

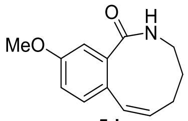

5d

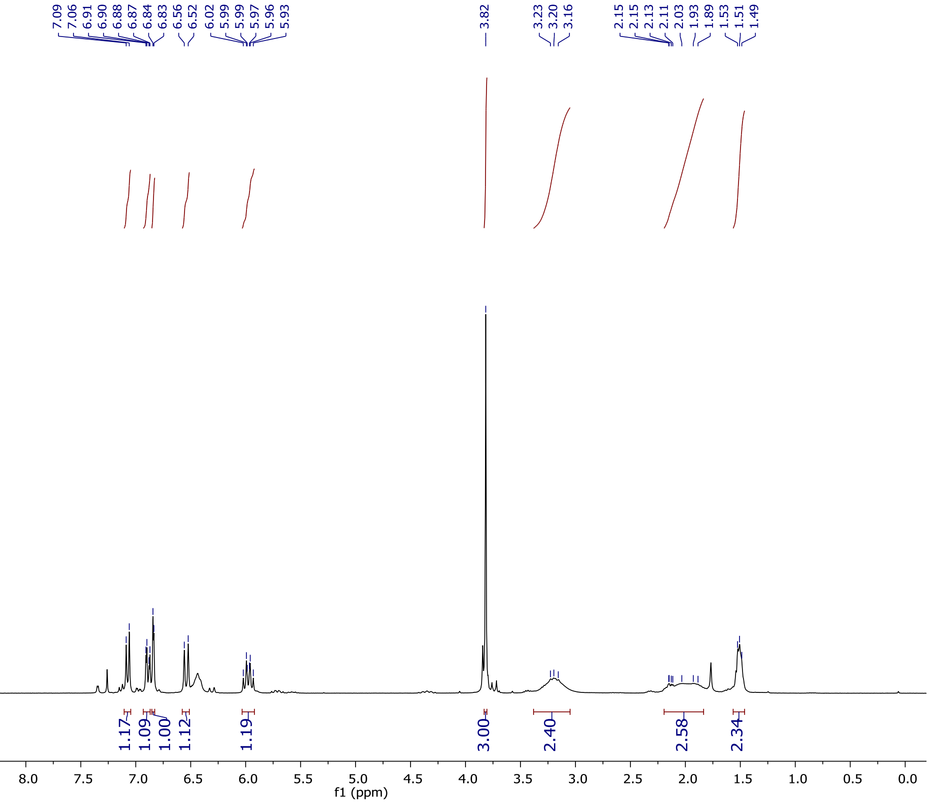

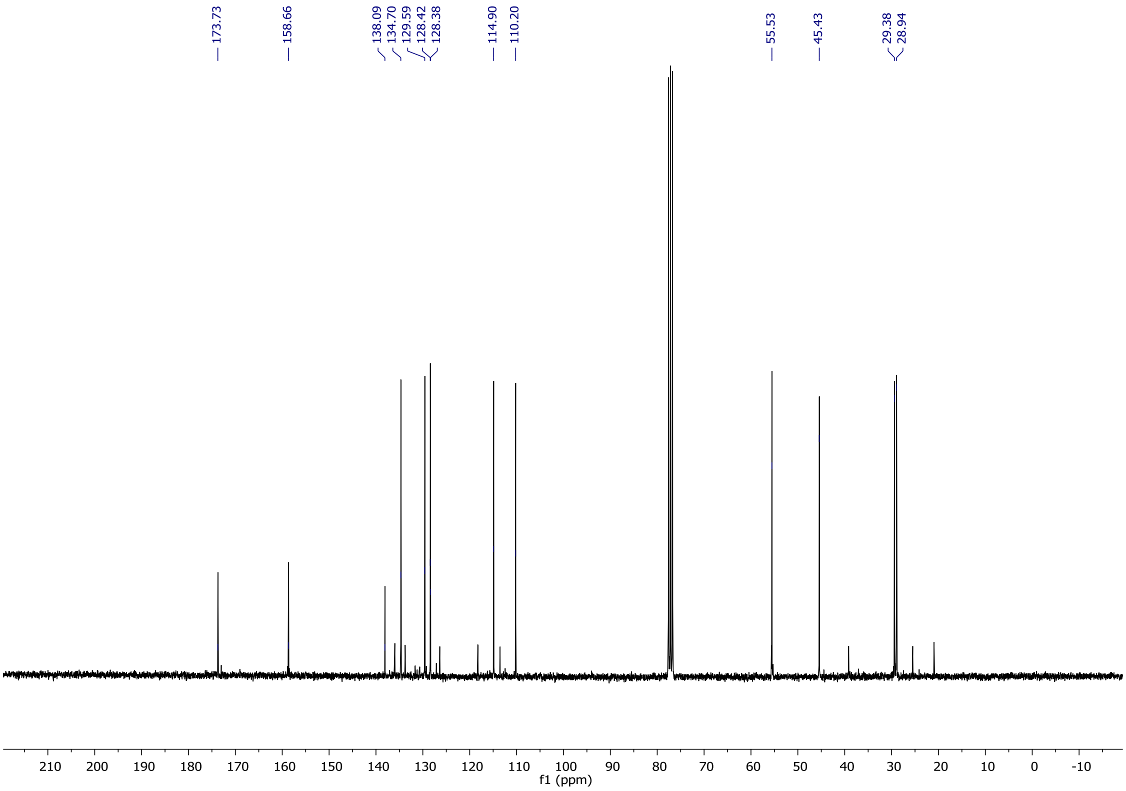

Figure SI-36. ^{1}H-NMR (300 MHz, $CDCl_3$) and ^{13}C {^{1}H} NMR (75 MHz, $CDCl_3$) spectra of compound **5d**

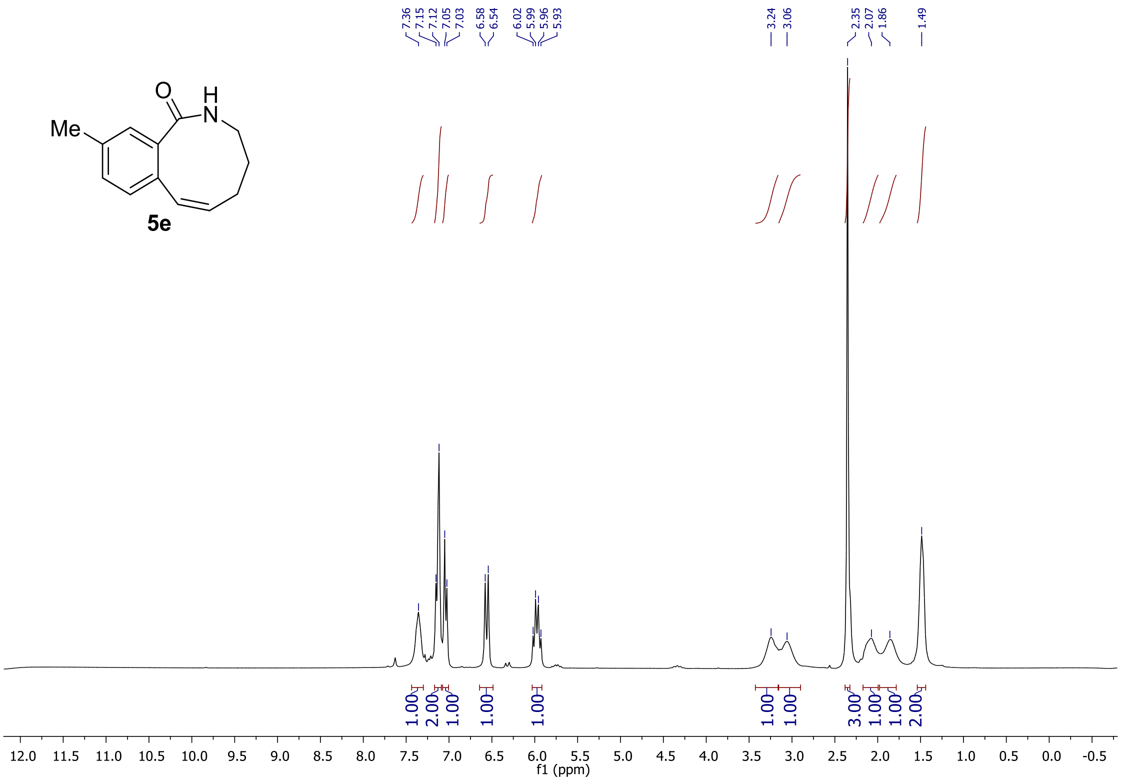

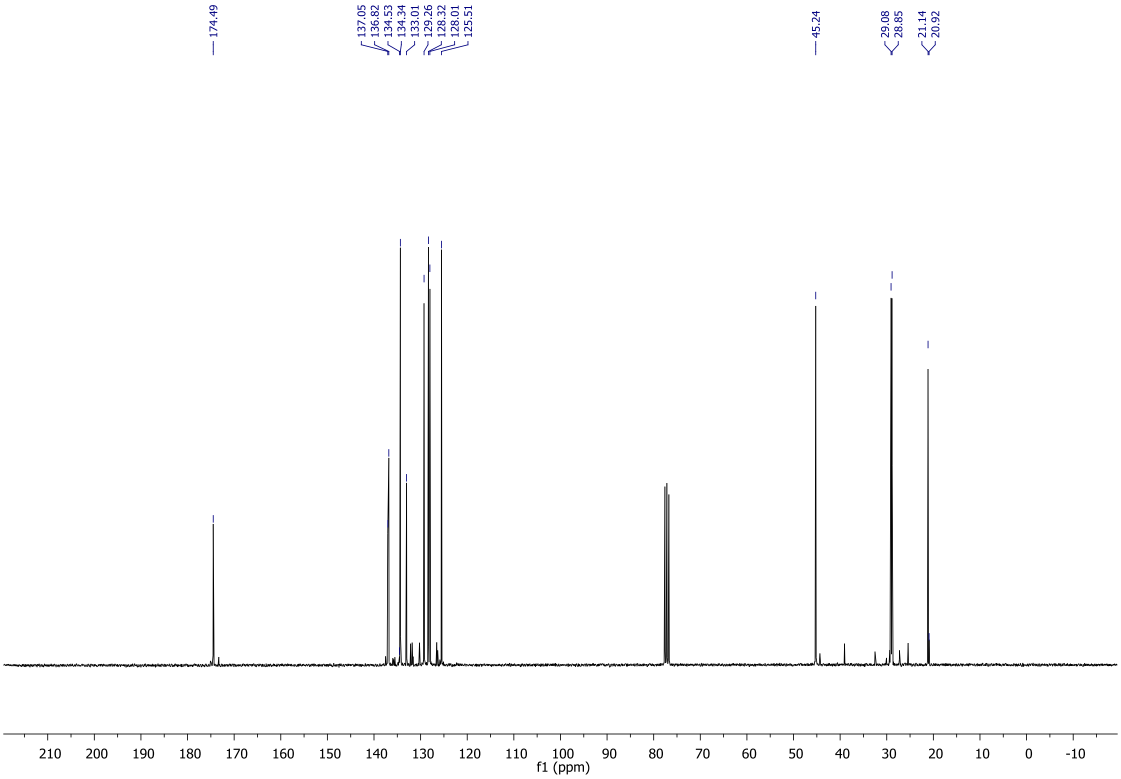

Figure SI-37. ^{1}H-NMR (300 MHz, $CDCl_3$) and ^{13}C {^{1}H} NMR (75 MHz, $CDCl_3$) spectra of compound **5e**

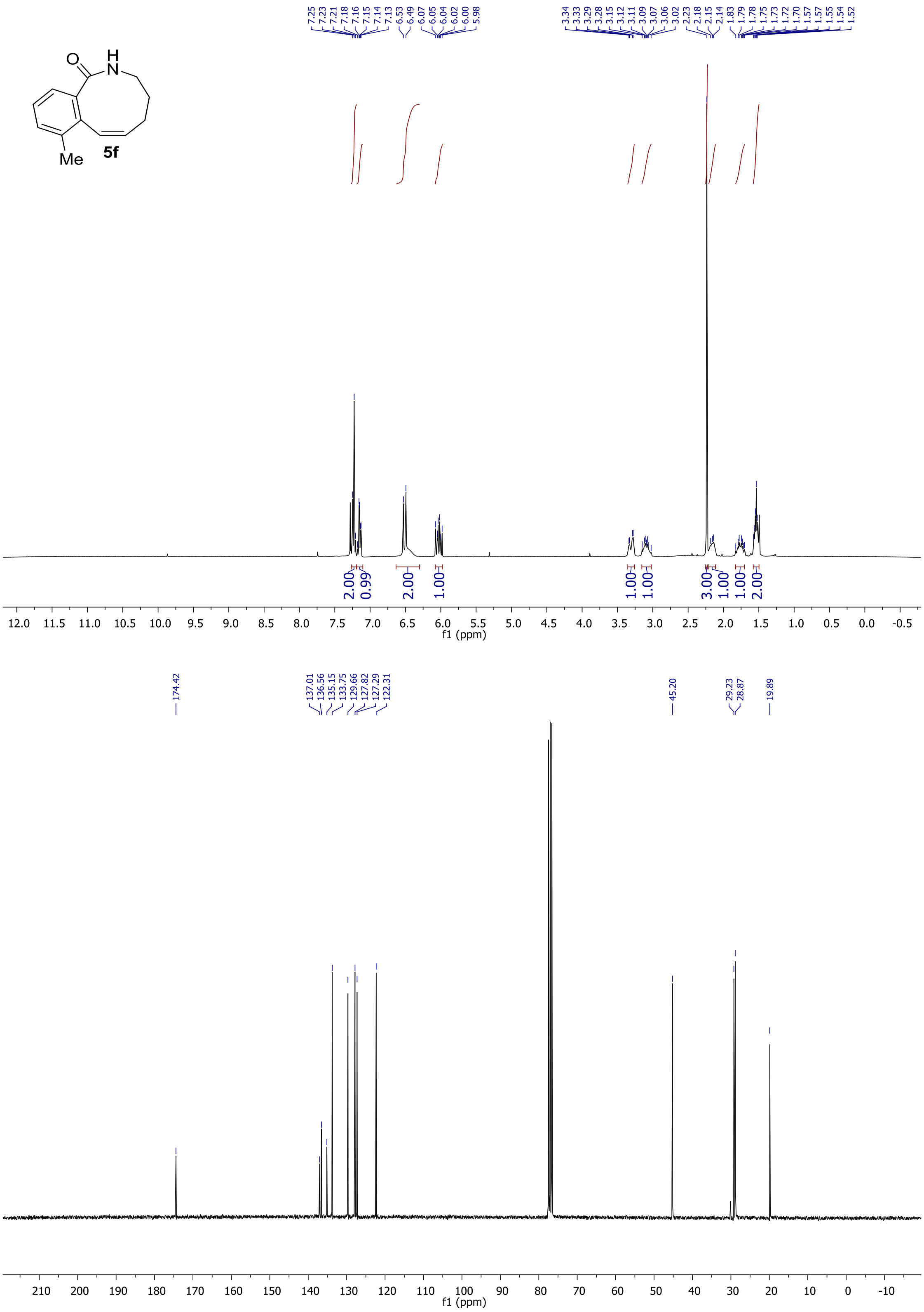

Figure SI-38. ^{1}H-NMR (300 MHz, $CDCl_3$) and ^{13}C {^{1}H} NMR (75 MHz, $CDCl_3$) spectra of compound **5f**

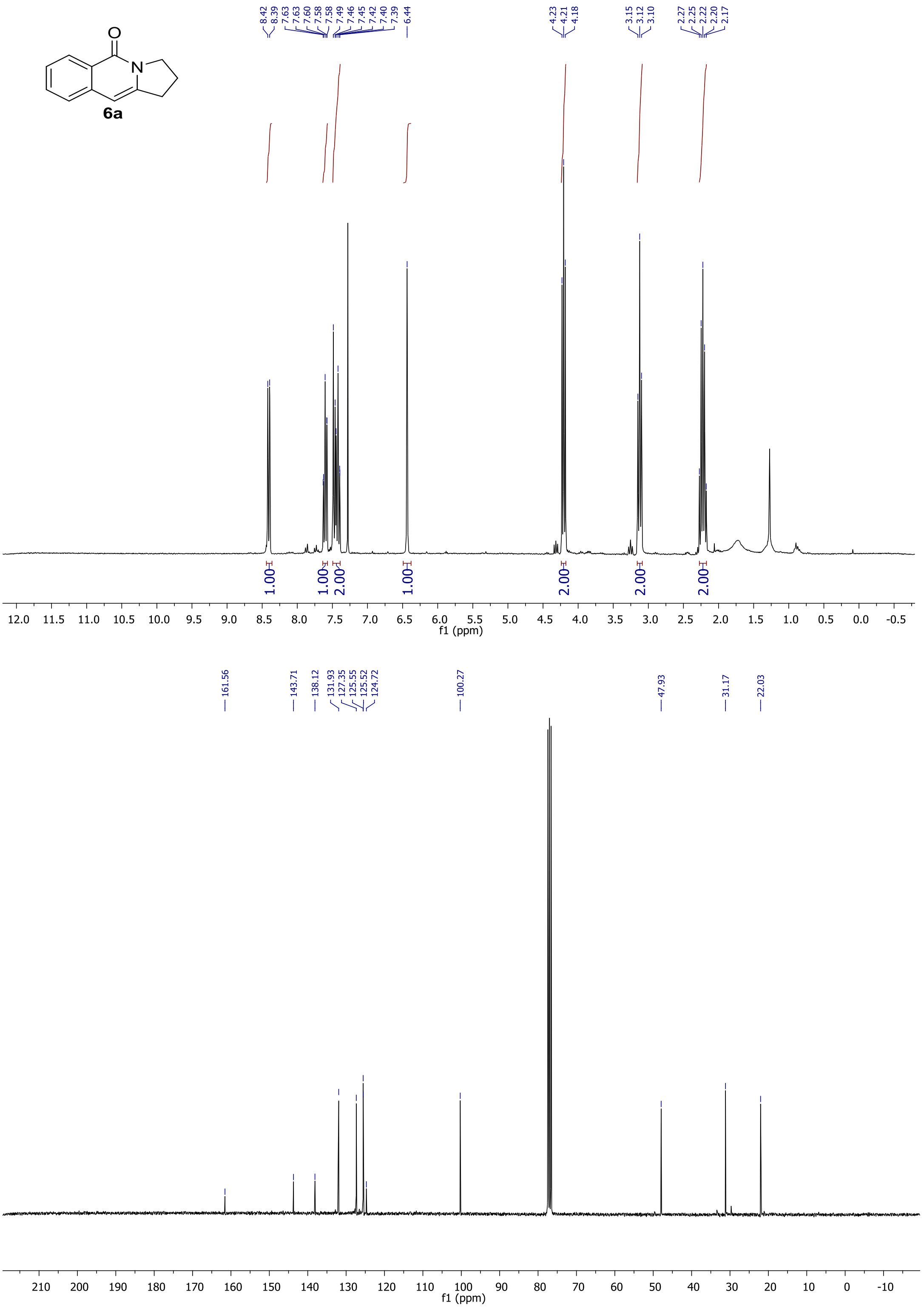

Figure SI-39. ^{1}H-NMR (300 MHz, $CDCl_3$) and ^{13}C {^{1}H} NMR (75 MHz, $CDCl_3$) spectra of compound **6a**

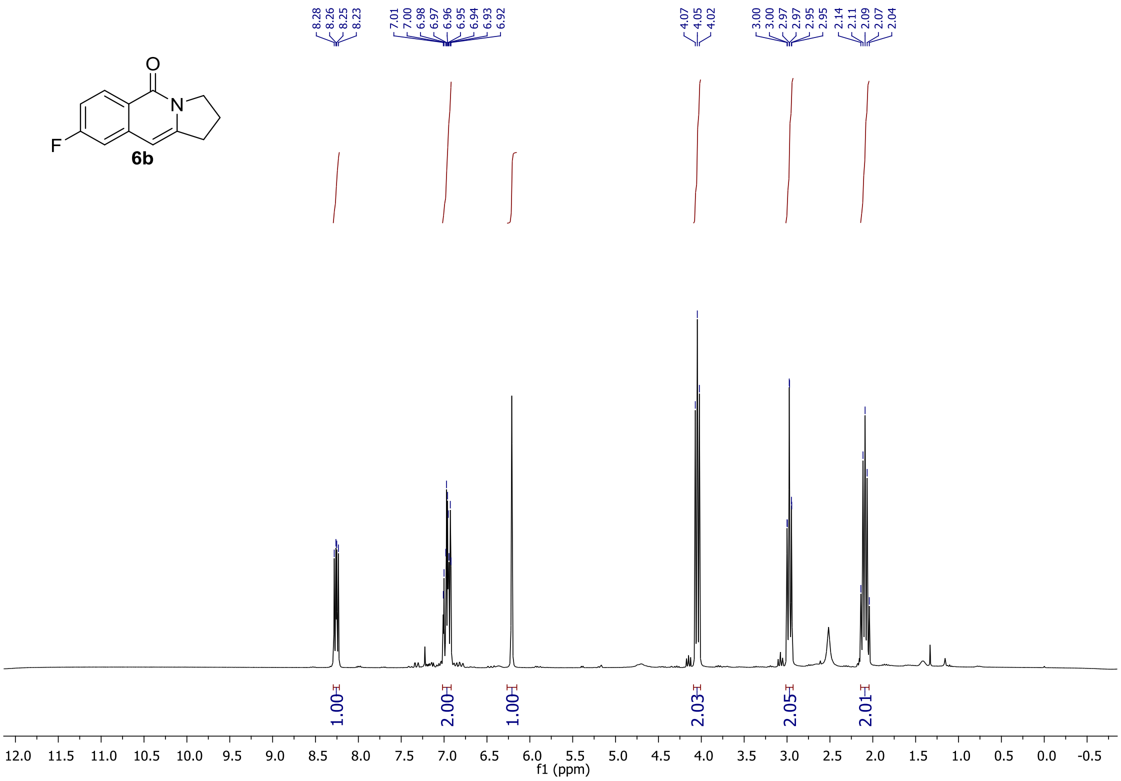

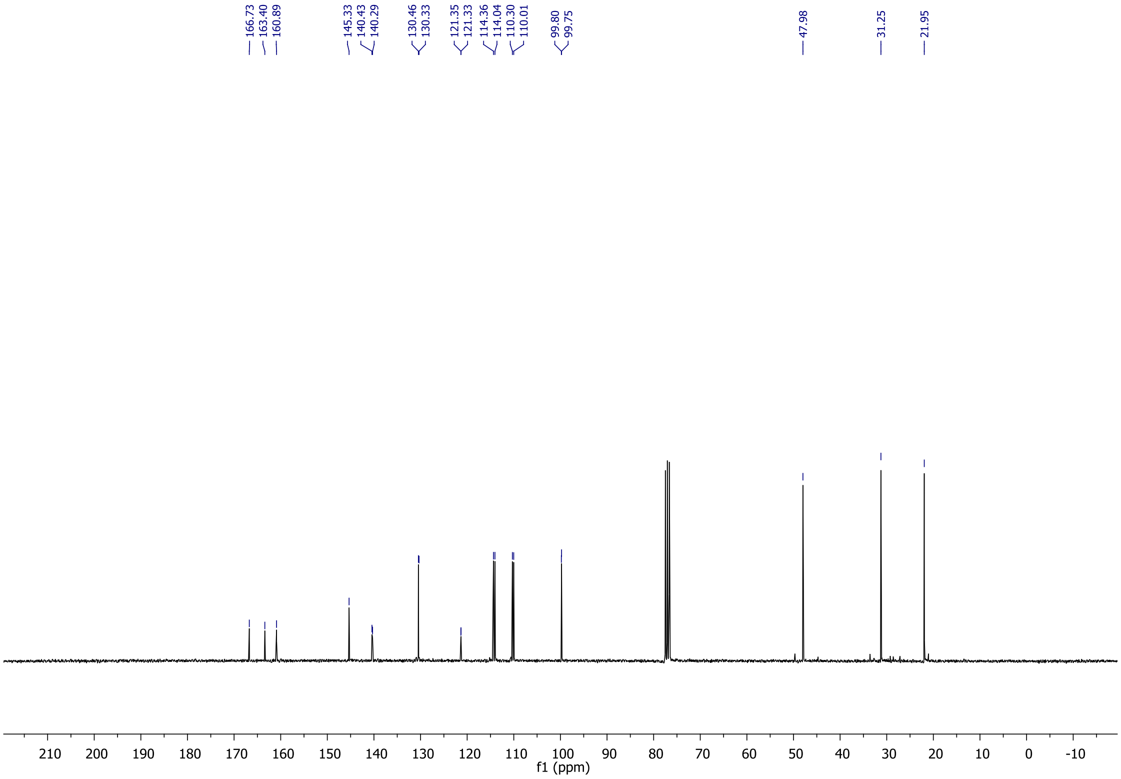

Figure SI-40. ^{1}H-NMR (300 MHz, $CDCl_3$) and ^{13}C {^{1}H} NMR (75 MHz, $CDCl_3$) spectra of compound **6b**

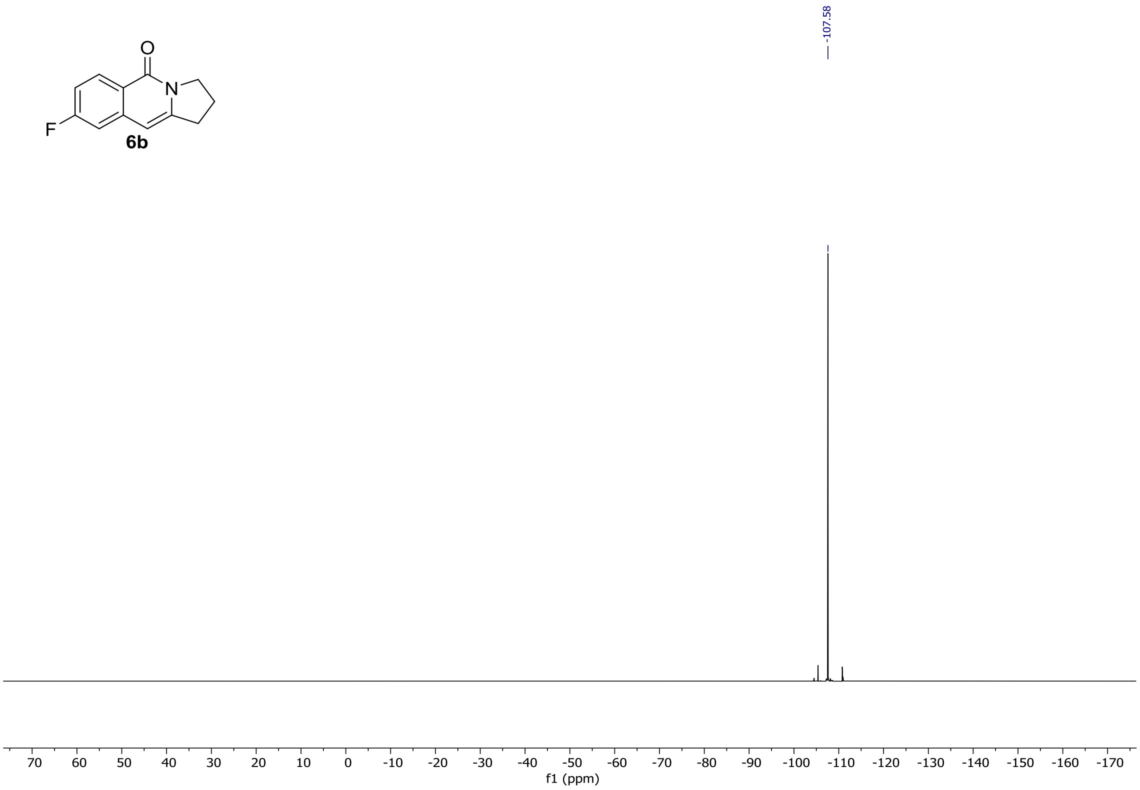

Figure SI-41. ^{19}F NMR (282 MHz, $CDCl_3$) spectrum of compound **6b**

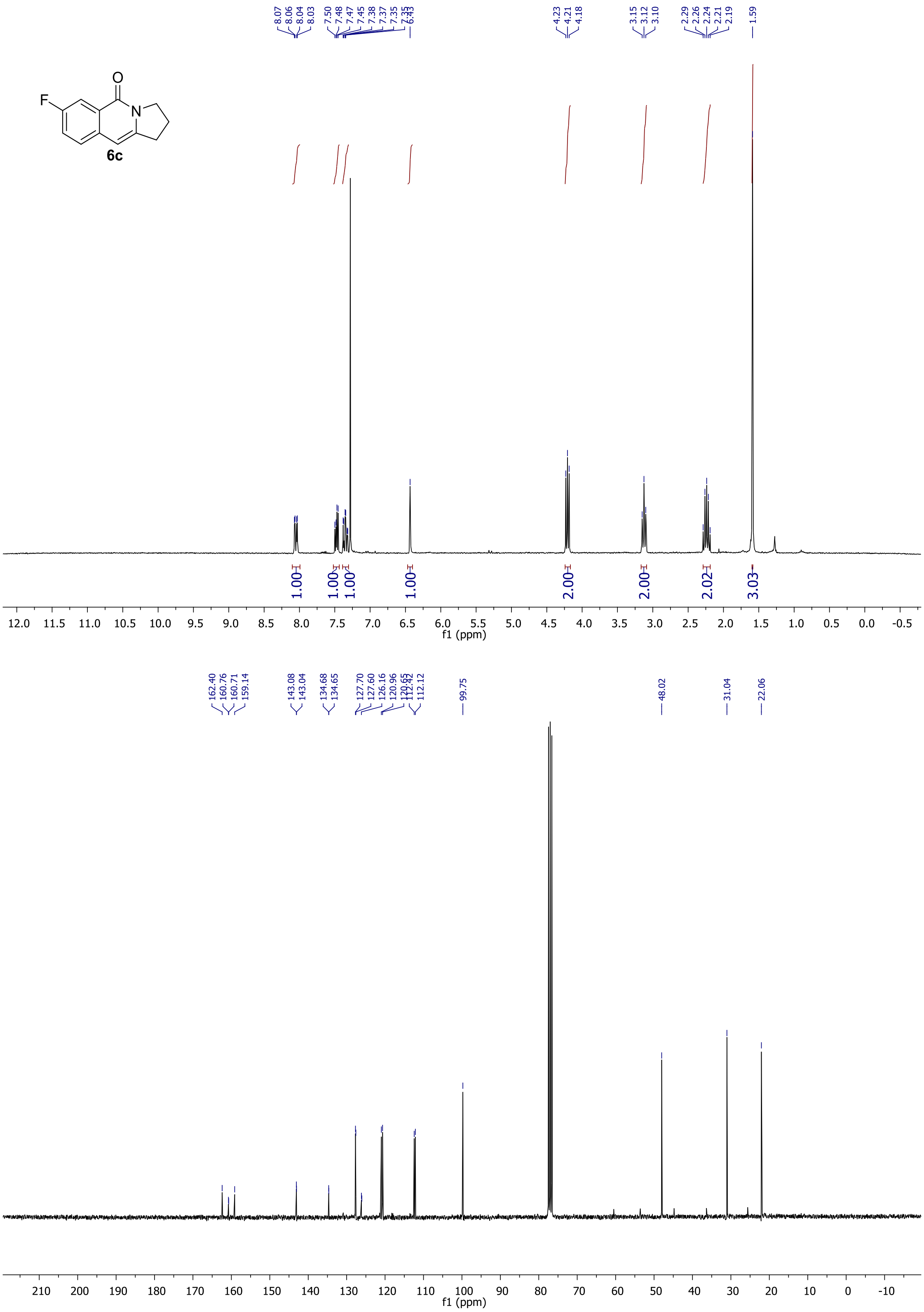

Figure SI-42. ^{1}H-NMR (300 MHz, $CDCl_3$) and ^{13}C {^{1}H} NMR (75 MHz, $CDCl_3$) spectra of compound **6c**

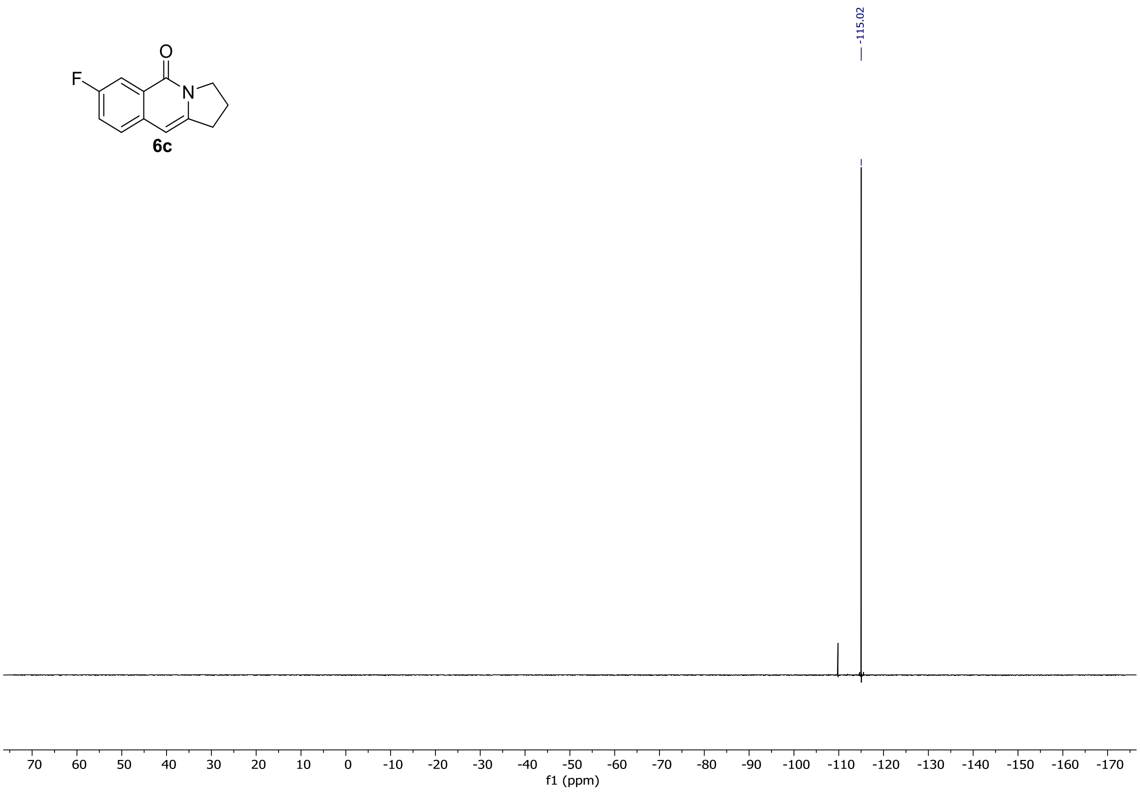

Figure SI-43. ^{19}F NMR (282 MHz, $CDCl_3$) spectrum of compound **6c**

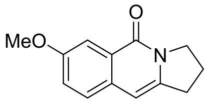

6d

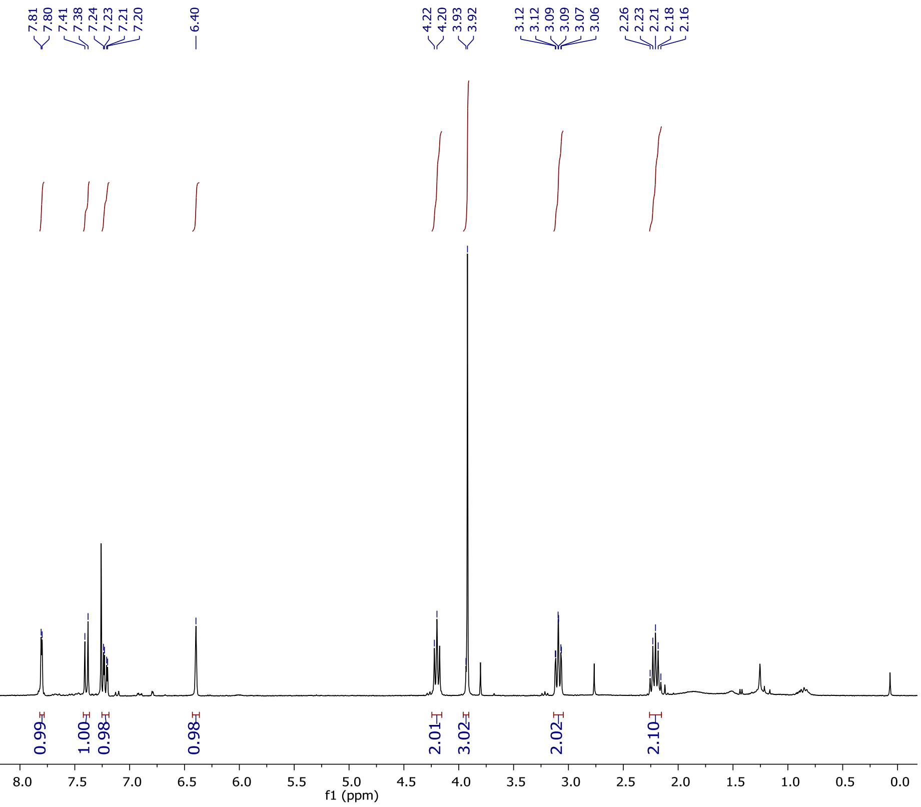

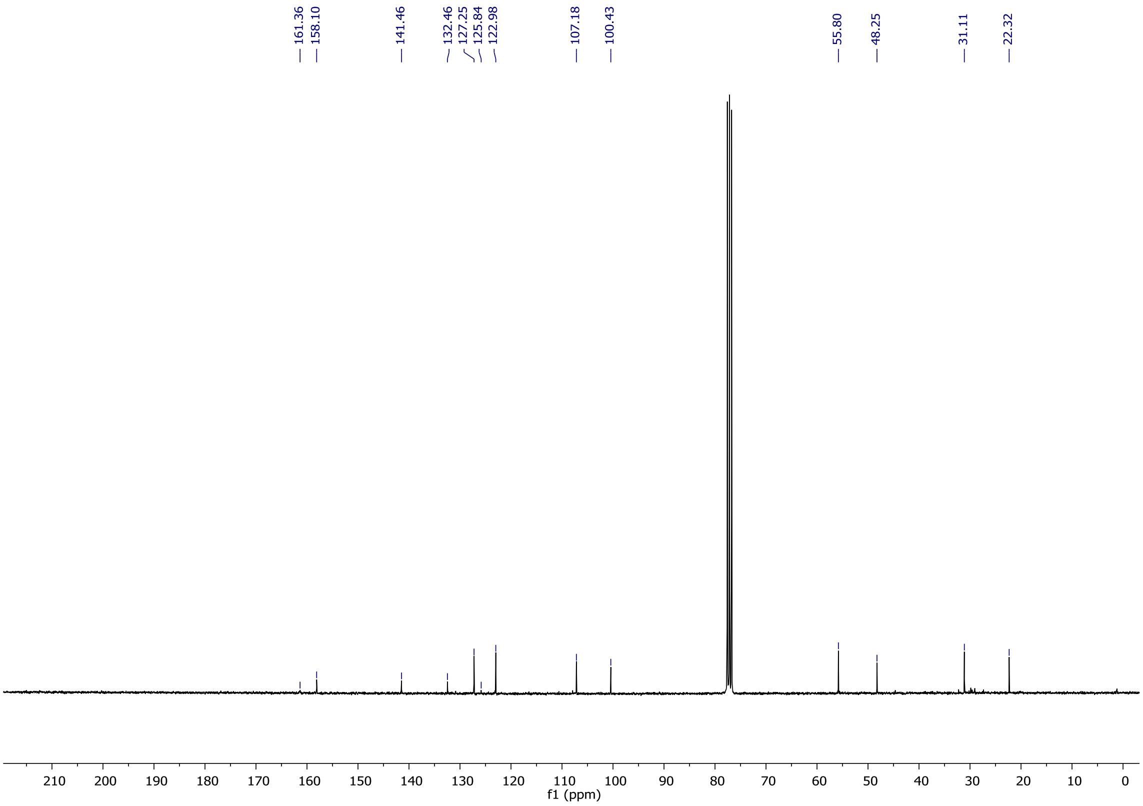

Figure SI-44. ^{1}H-NMR (300 MHz, $CDCl_3$) and ^{13}C {^{1}H} NMR (75 MHz, $CDCl_3$) spectra of compound **6d**

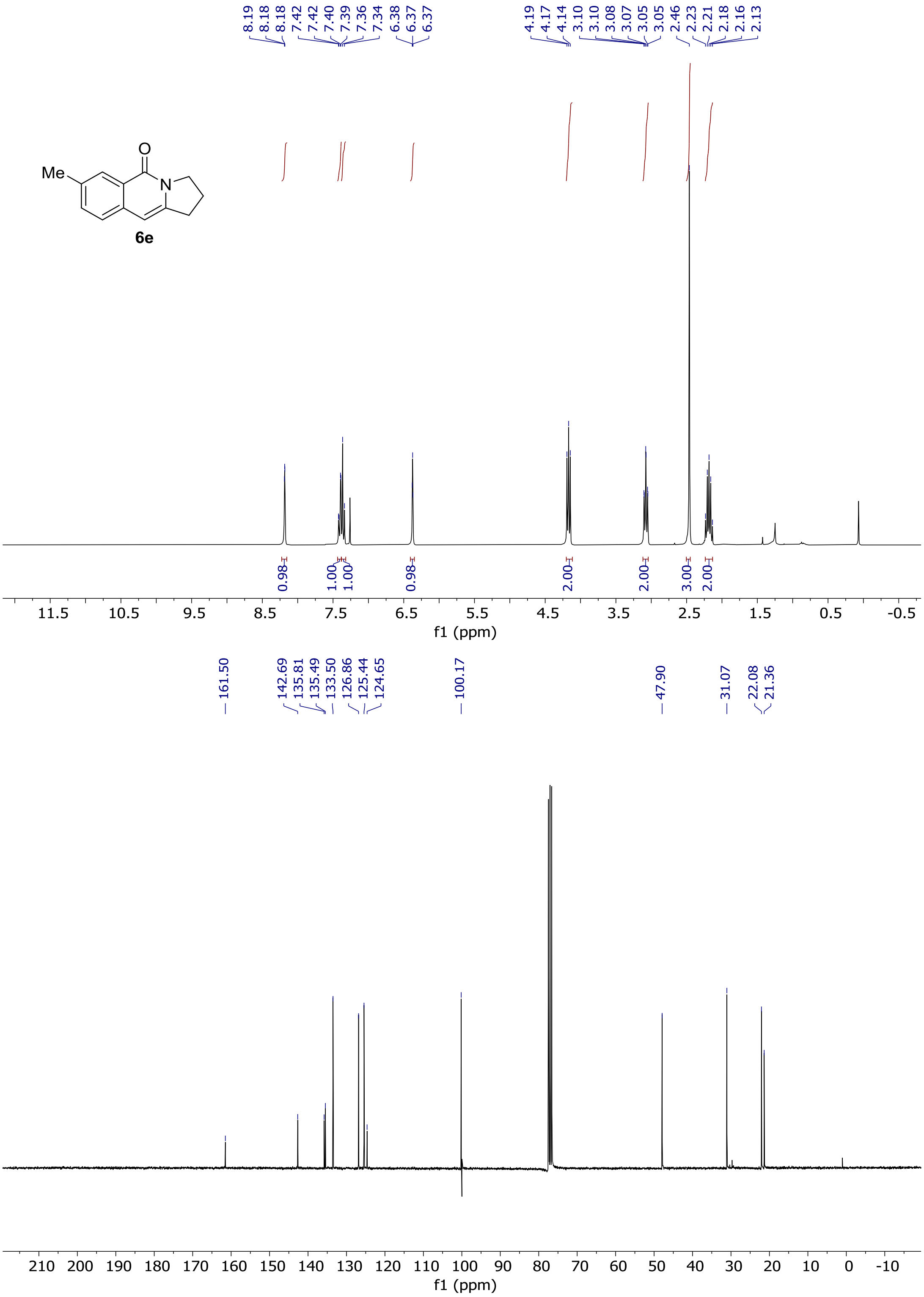

Figure SI-45. ^{1}H-NMR (300 MHz, $CDCl_3$) and ^{13}C {^{1}H} NMR (75 MHz, $CDCl_3$) spectra of compound **6e**

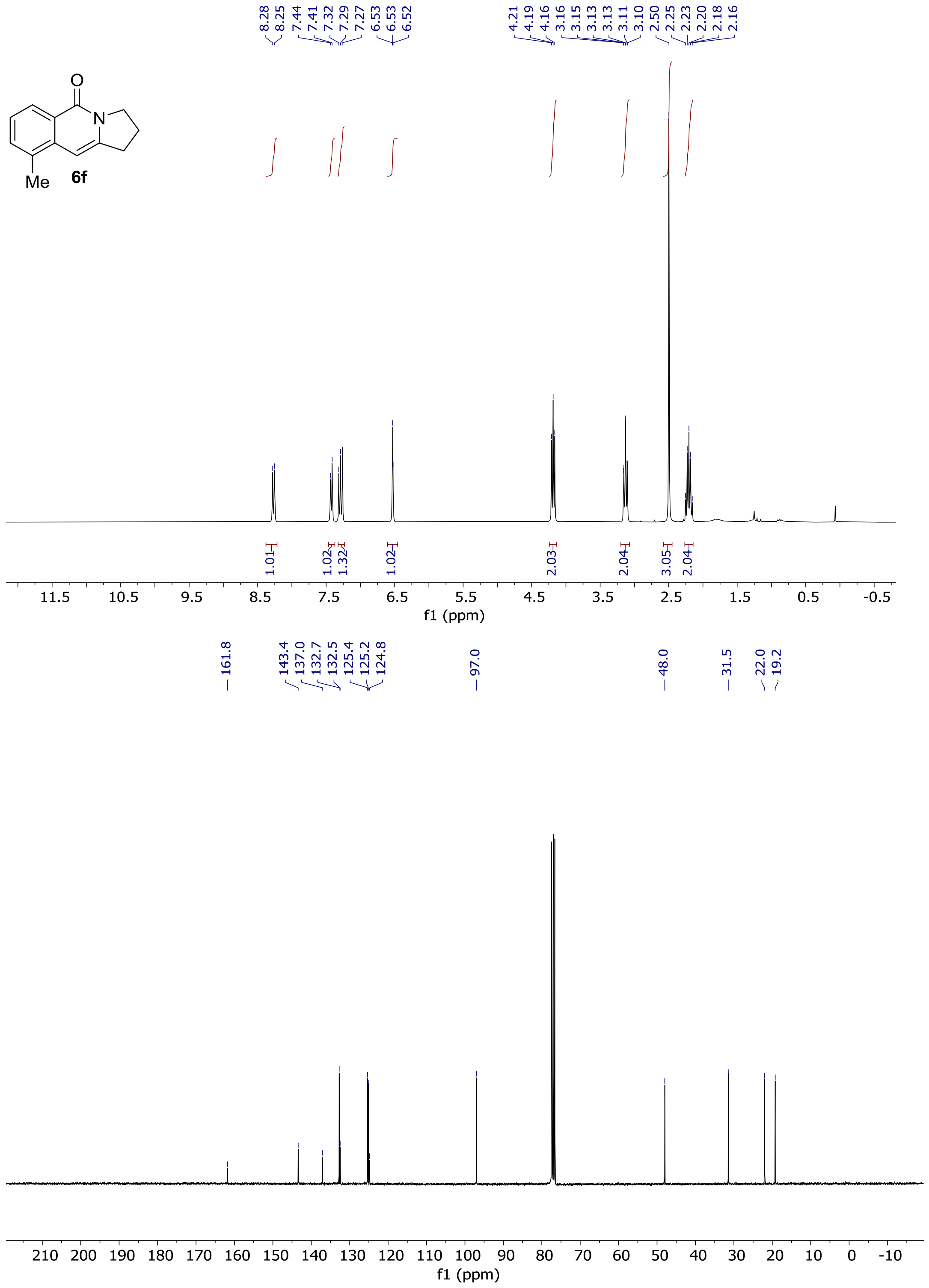

Figure SI-46. ^{1}H-NMR (300 MHz, $CDCl_3$) and ^{13}C {^{1}H} NMR (75 MHz, $CDCl_3$) spectra of compound **6f**

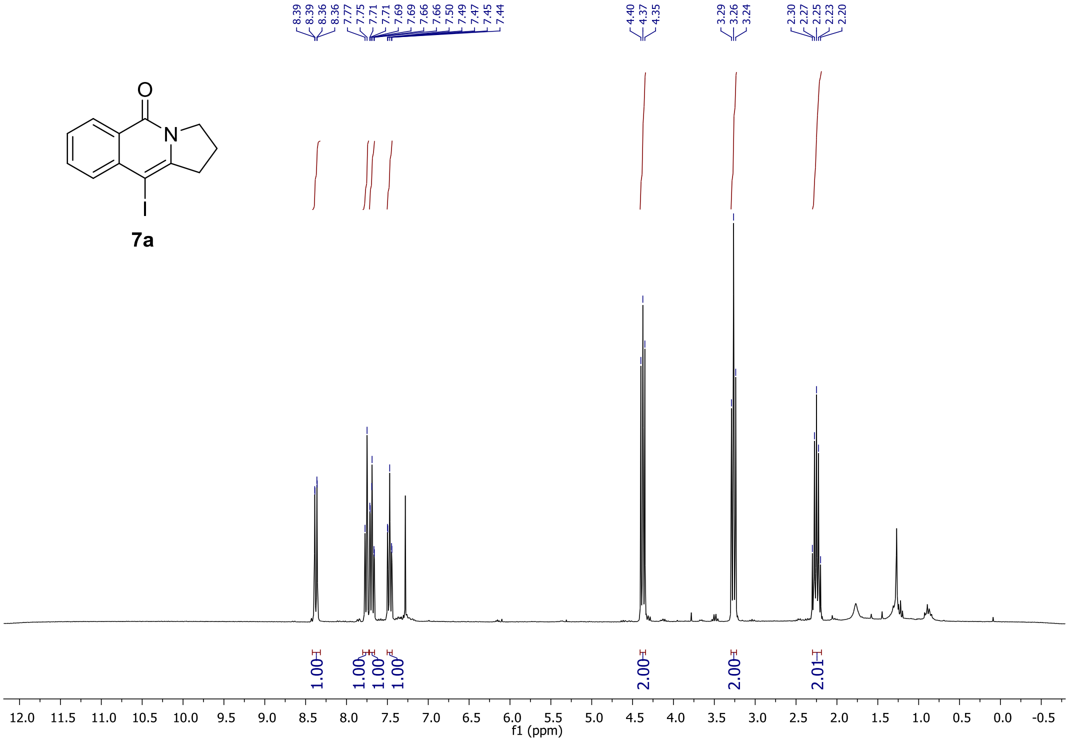

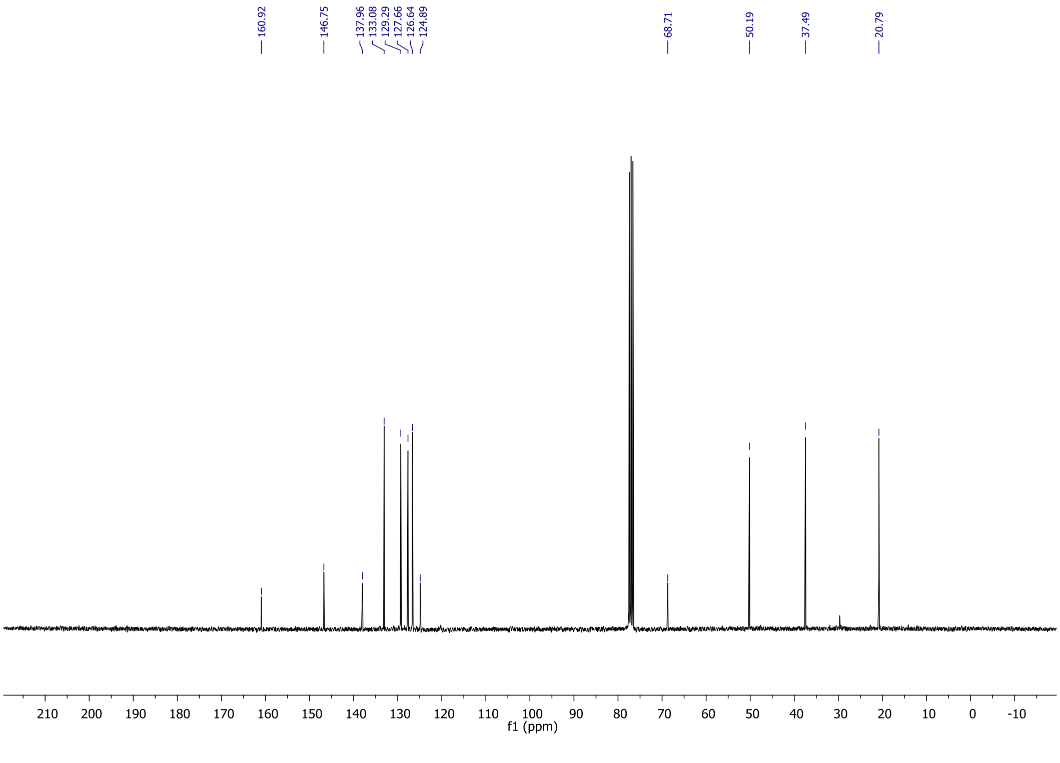

Figure SI-47. ^{1}H-NMR (300 MHz, $CDCl_3$) and ^{13}C {^{1}H} NMR (75 MHz, $CDCl_3$) spectra of compound **7a**

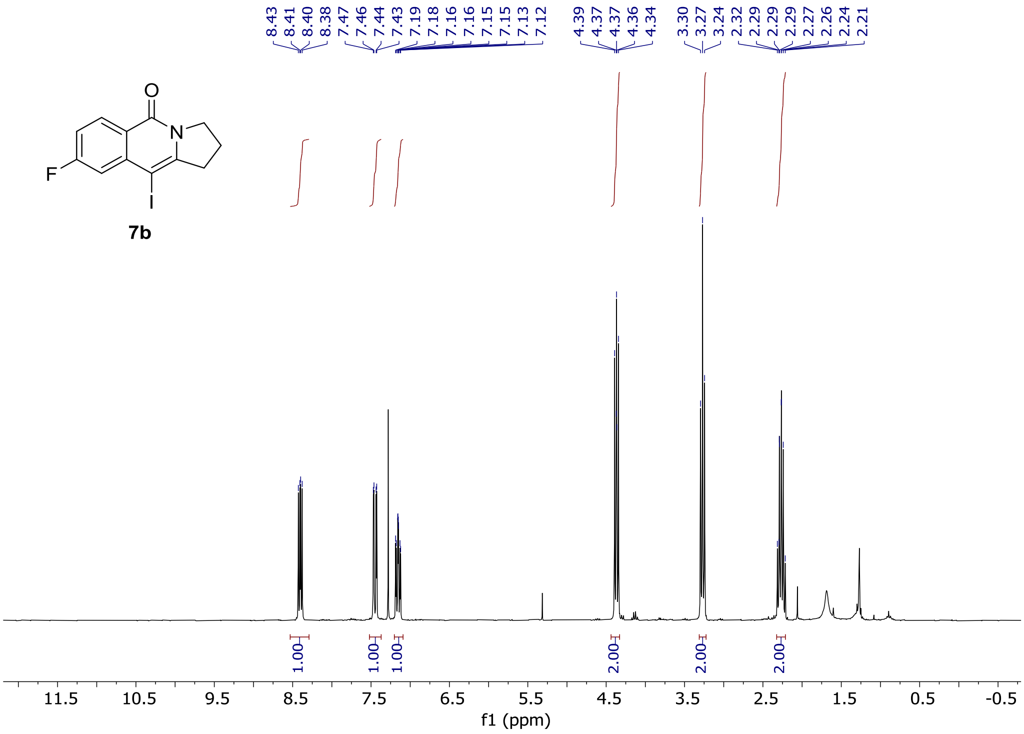

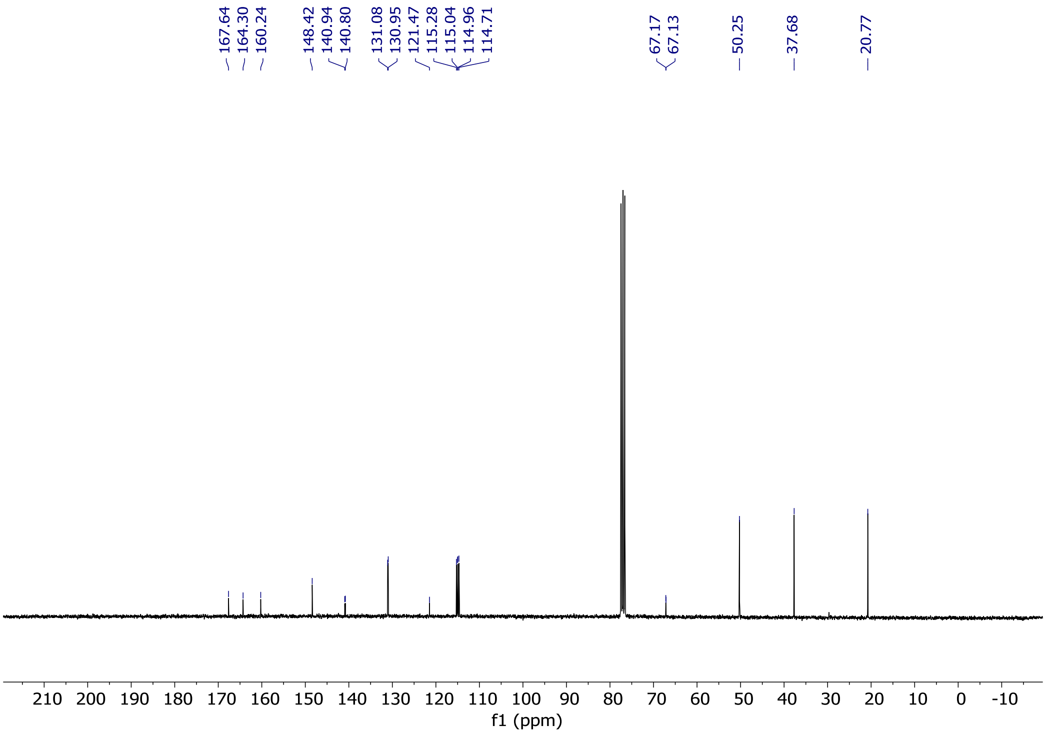

Figure SI-48. ^{1}H-NMR (300 MHz, $CDCl_3$) and ^{13}C {^{1}H} NMR (75 MHz, $CDCl_3$) spectra of compound **7b**

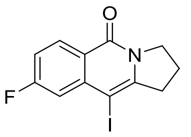

7b

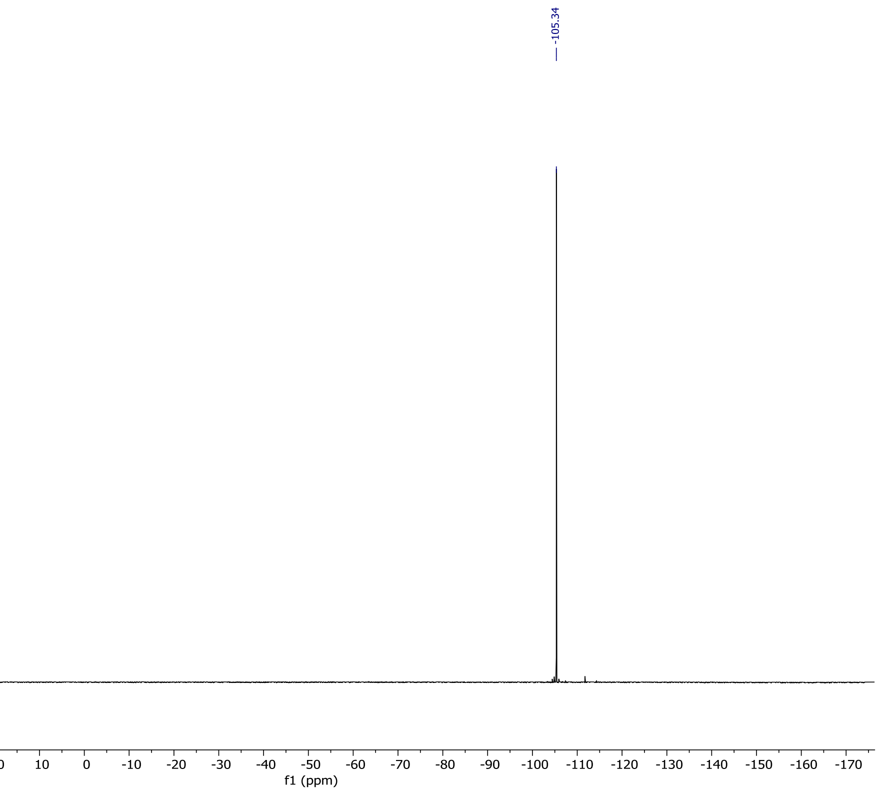

Figure SI-49. ^{19}F NMR (282 MHz, $CDCl_3$) spectrum of compound **7b**

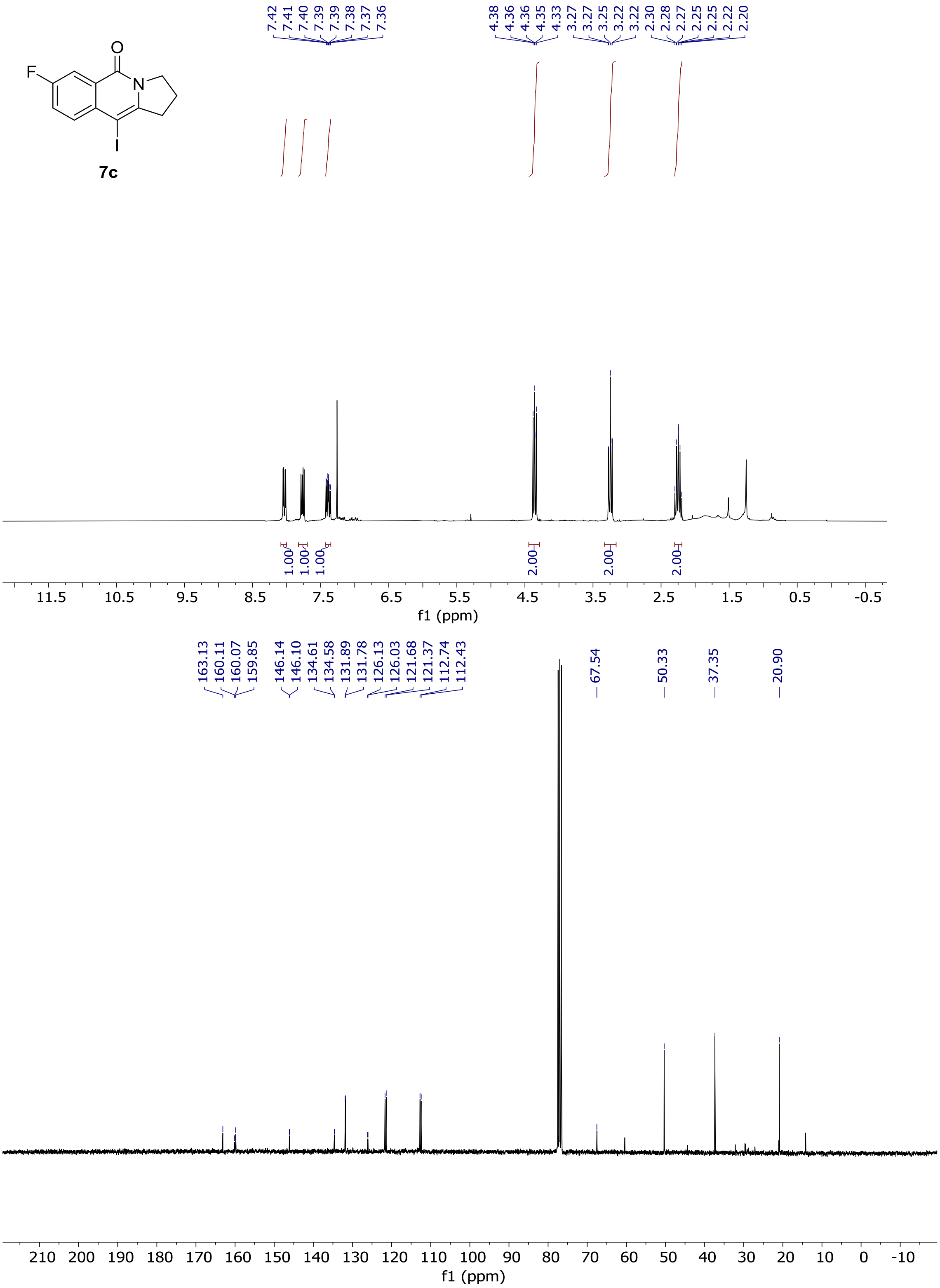

Figure SI-50. ^{1}H-NMR (300 MHz, $CDCl_3$) and ^{13}C {^{1}H} NMR (75 MHz, $CDCl_3$) spectra of compound **7c**

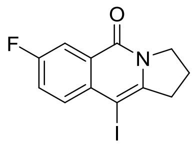

7c

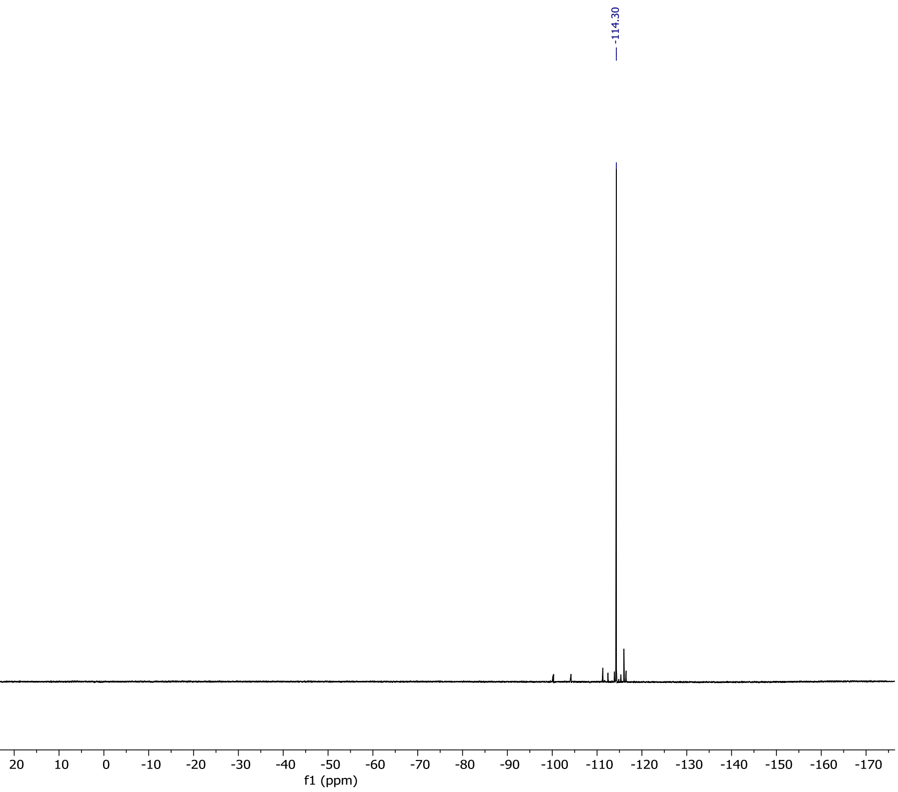

Figure SI-51. ^{19}F NMR (282 MHz, $CDCl_3$) spectrum of compound **7c**

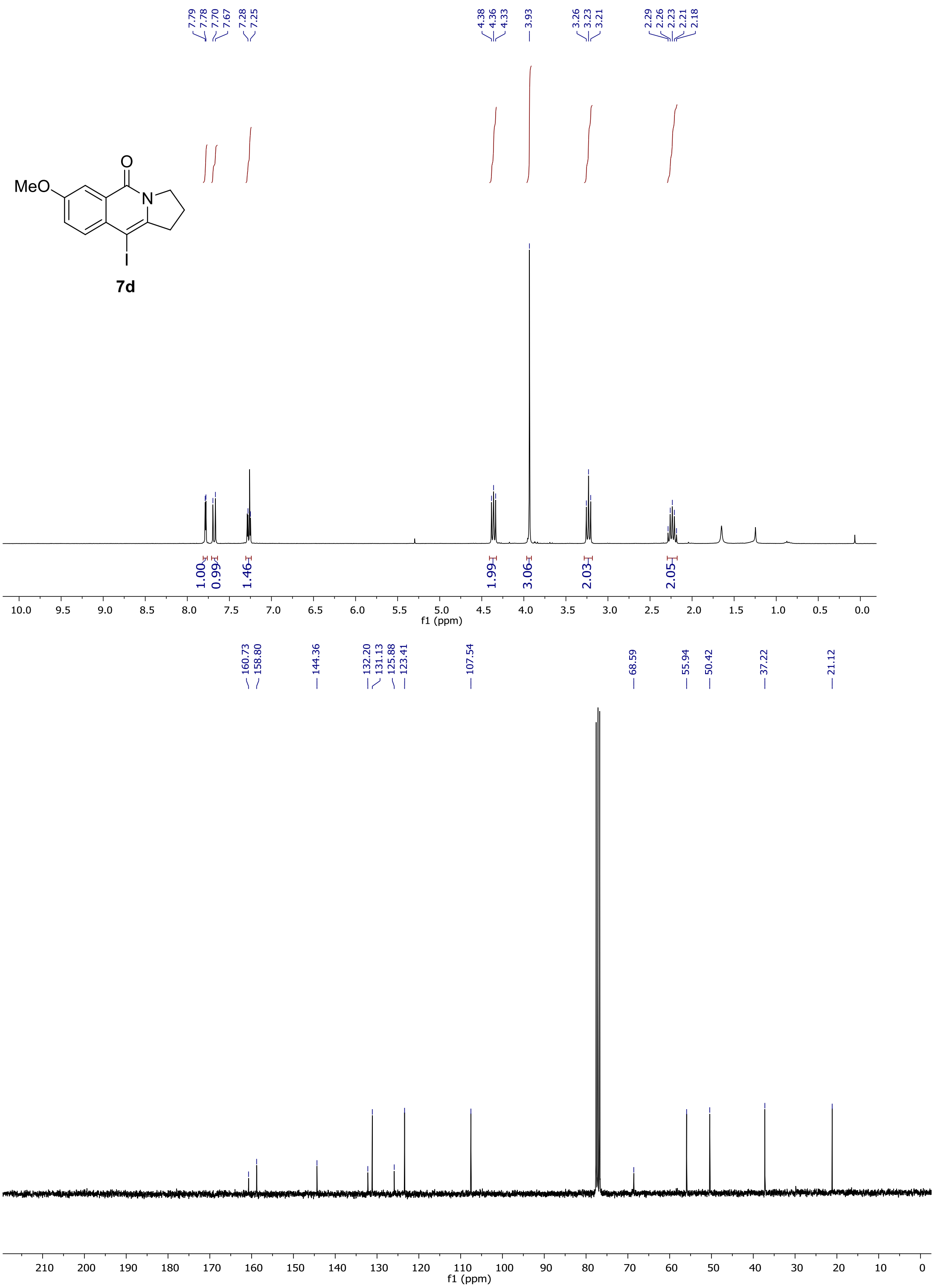

Figure SI-52. ^{1}H-NMR (300 MHz, $CDCl_3$) and ^{13}C {^{1}H} NMR (75 MHz, $CDCl_3$) spectra of compound **7d**

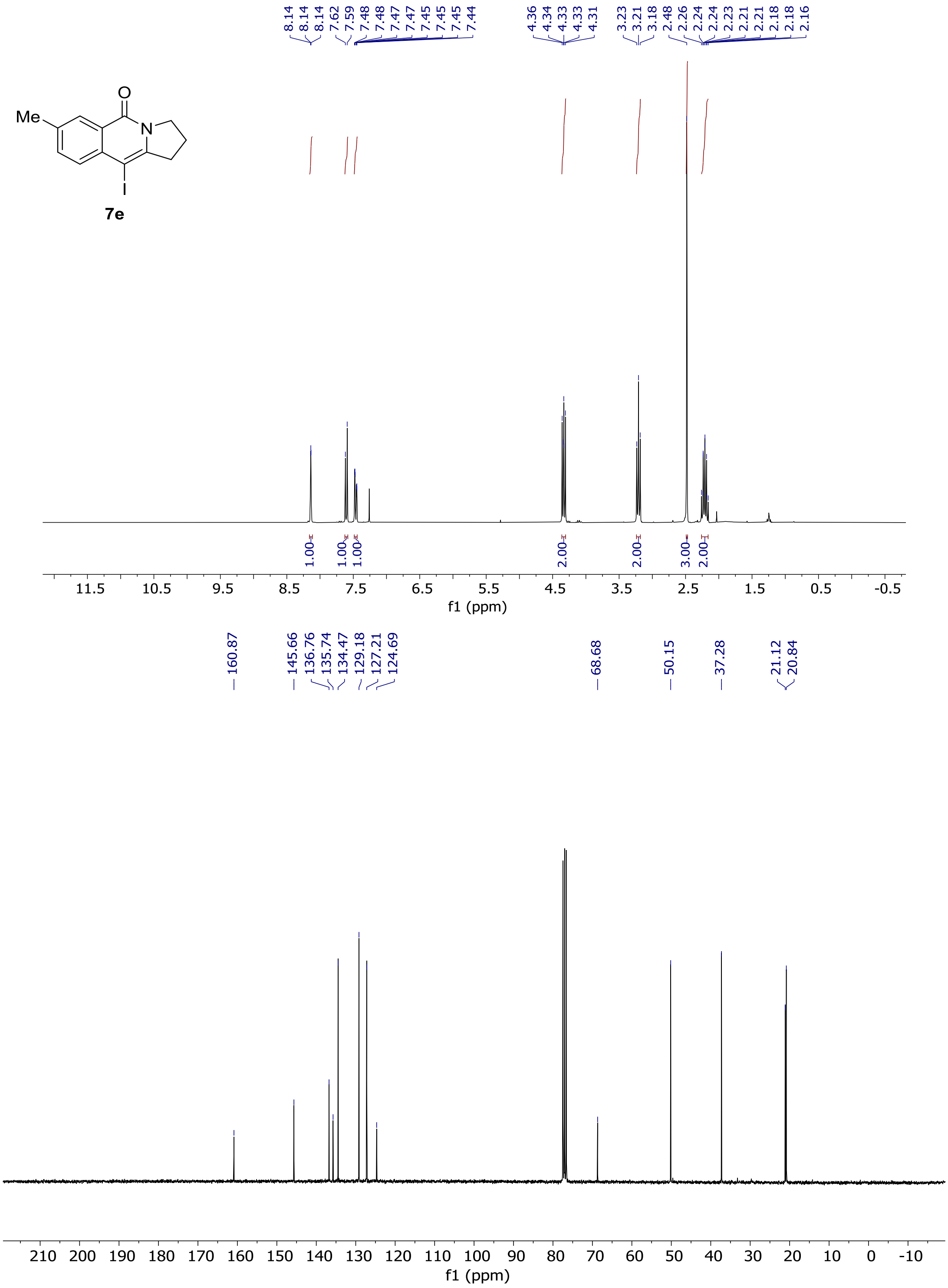

Figure SI-53. ^{1}H-NMR (300 MHz, $CDCl_3$) and ^{13}C {^{1}H} NMR (75 MHz, $CDCl_3$) spectra of compound **7e**

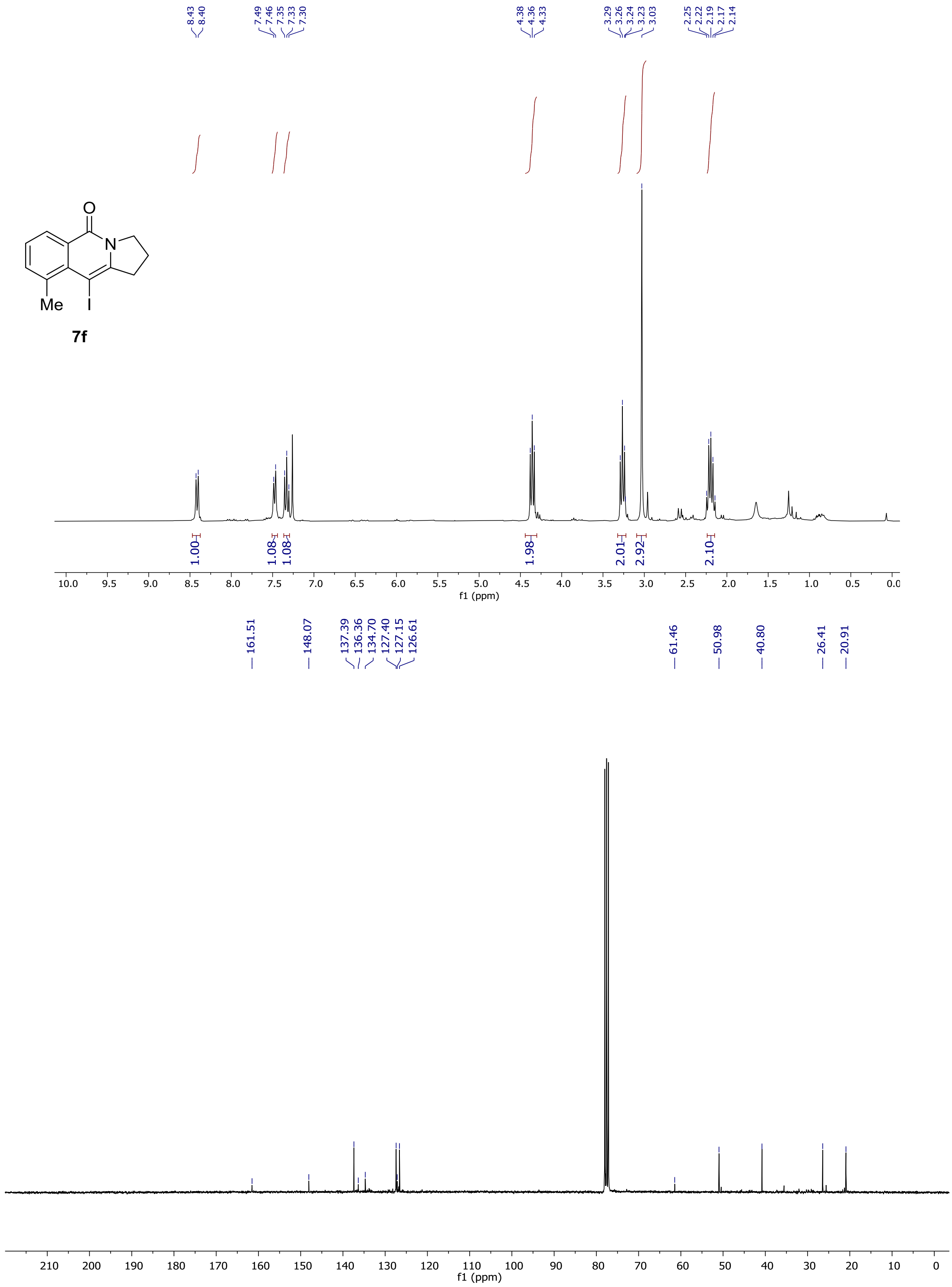

Figure SI-54. ^{1}H-NMR (300 MHz, $CDCl_3$) and ^{13}C {^{1}H} NMR (75 MHz, $CDCl_3$) spectra of compound **7f**

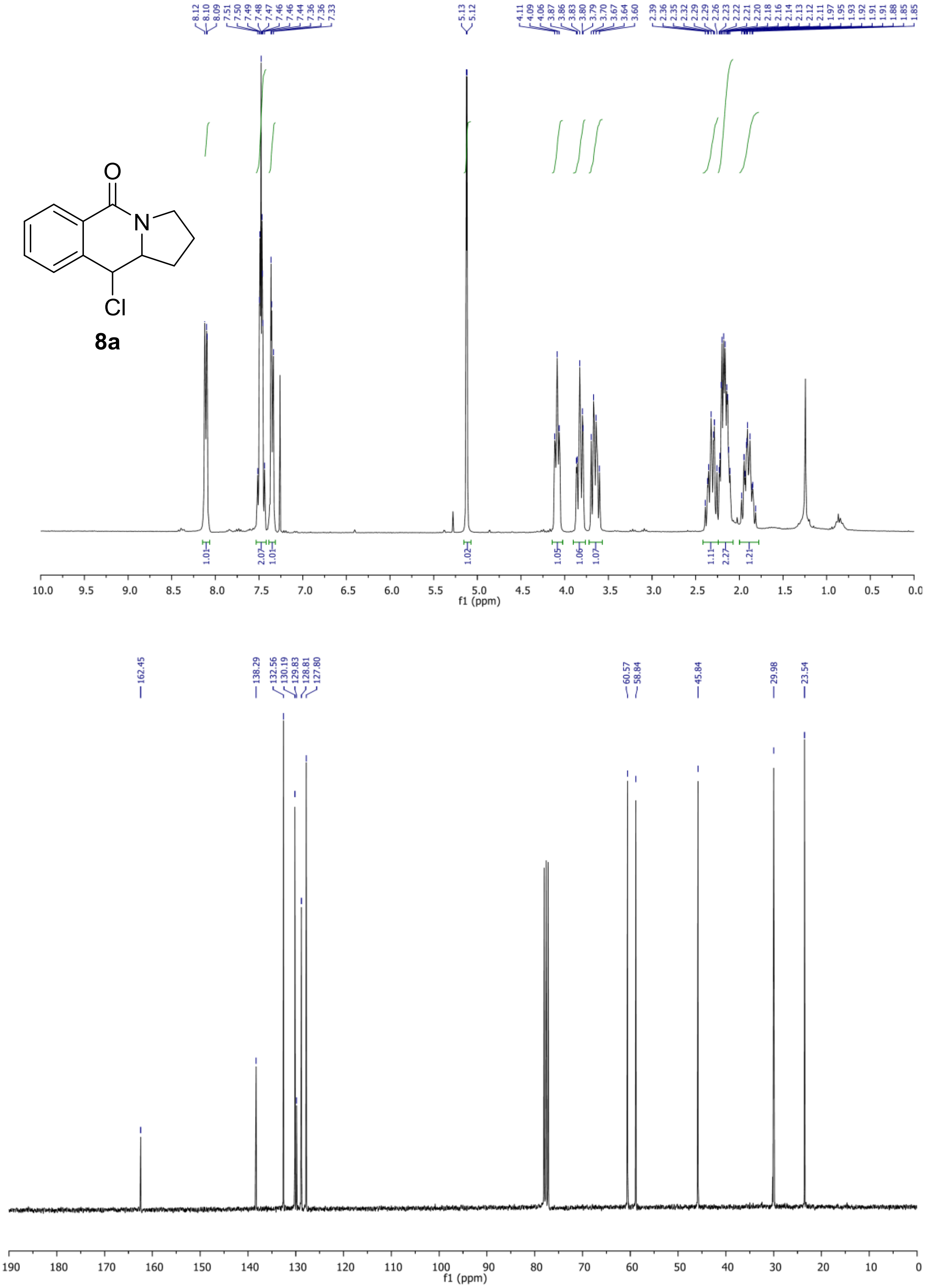

Figure SI-55. ^{1}H-NMR (300 MHz, $CDCl_3$) and ^{13}C {^{1}H} NMR (75 MHz, $CDCl_3$) spectra of compound **8a**

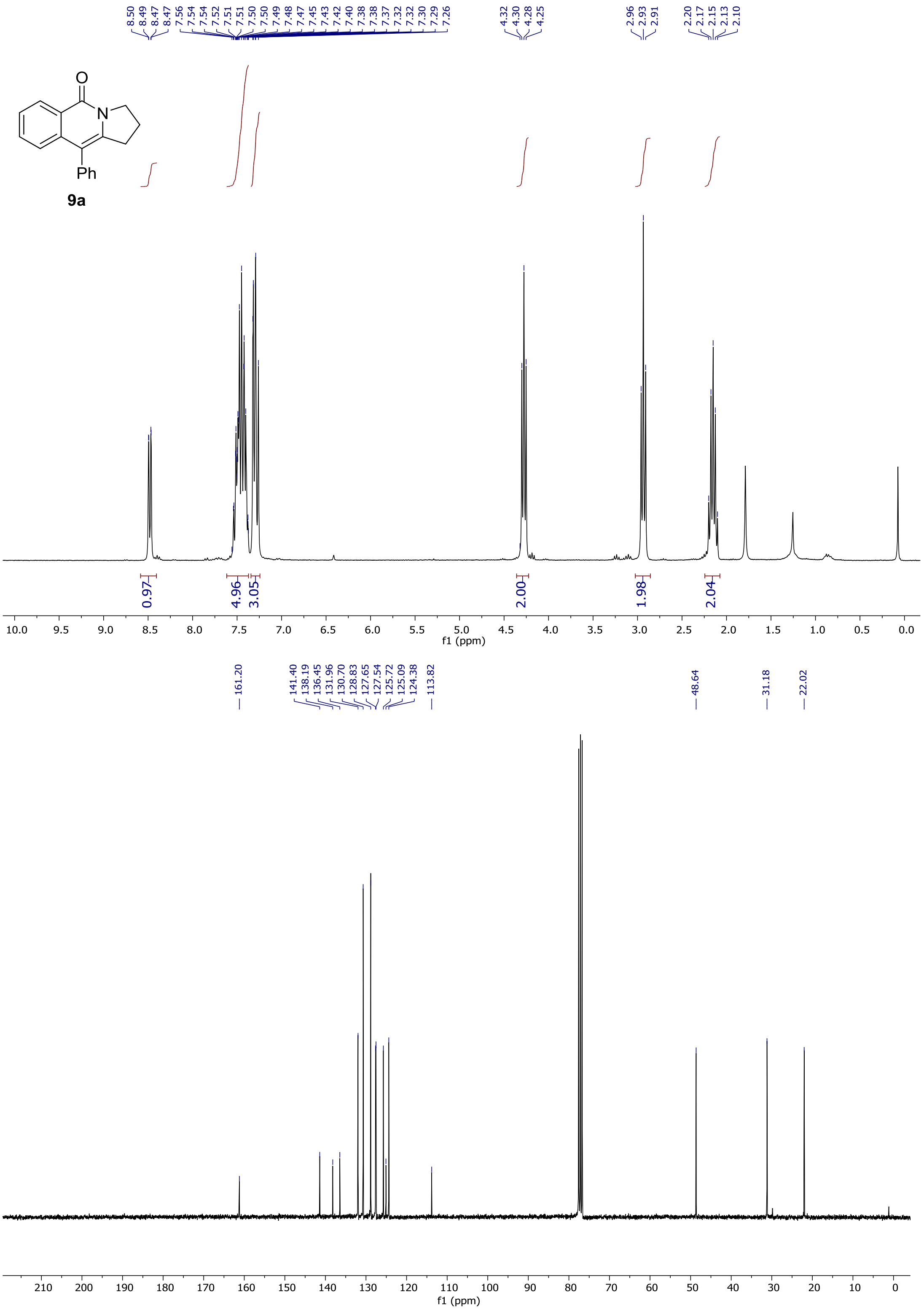

Figure SI-56. ¹H-NMR (300 MHz, $CDCl_3$) and ^{13}C {¹H} NMR (75 MHz, $CDCl_3$) spectra of compound **9a**

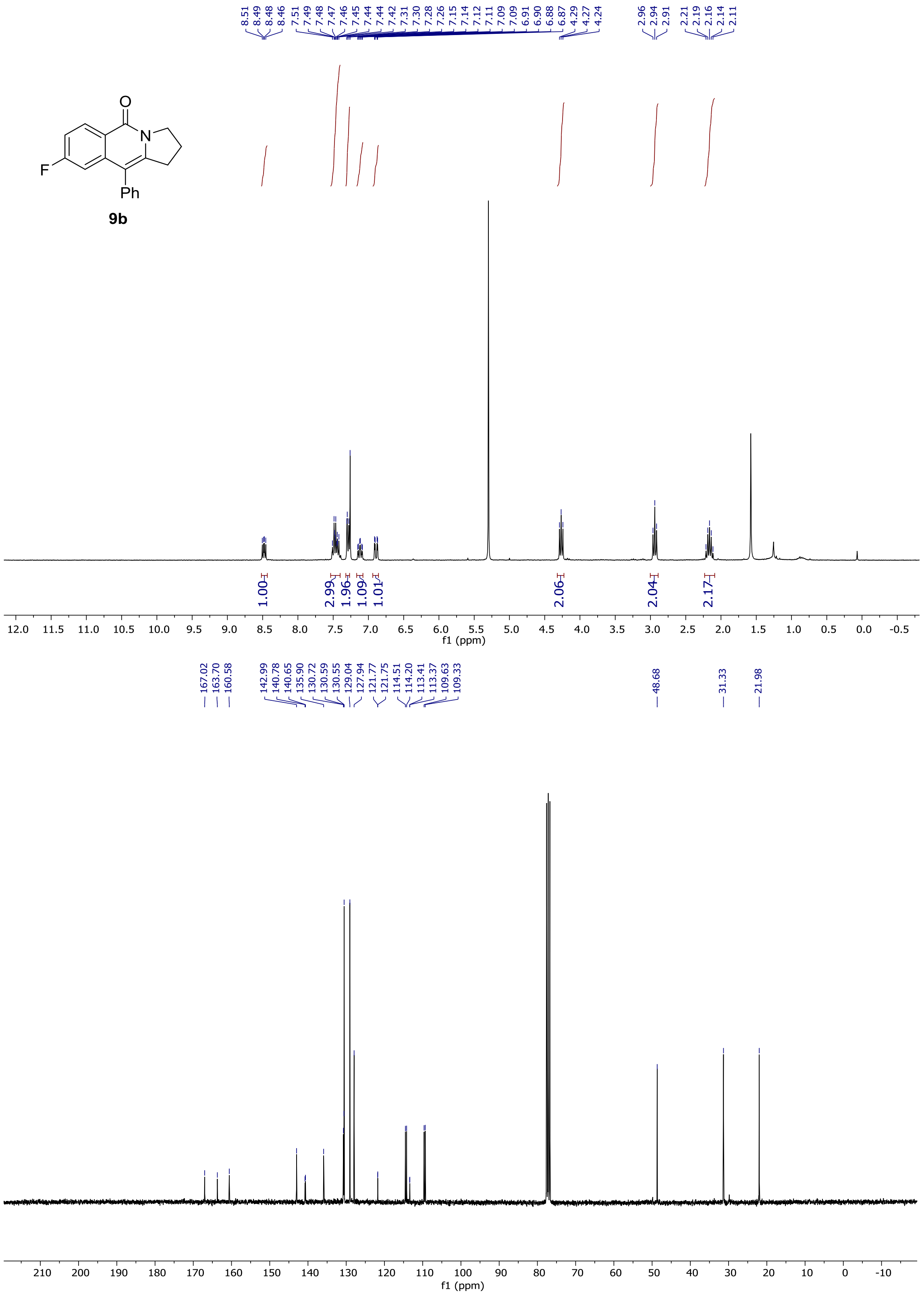

Figure SI-57. ^{1}H-NMR (300 MHz, $CDCl_3$) and ^{13}C {^{1}H} NMR (75 MHz, $CDCl_3$) spectra of compound **9b**

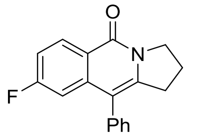

9b

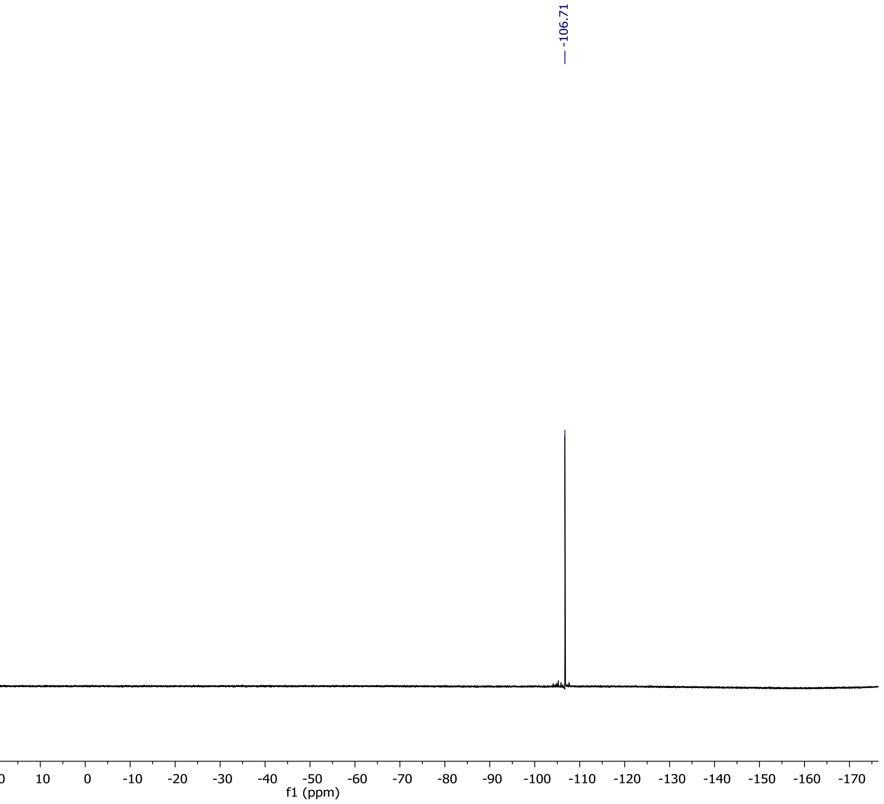

Figure SI-58. ^{19}F NMR (282 MHz, $CDCl_3$) spectrum of compound **9b**

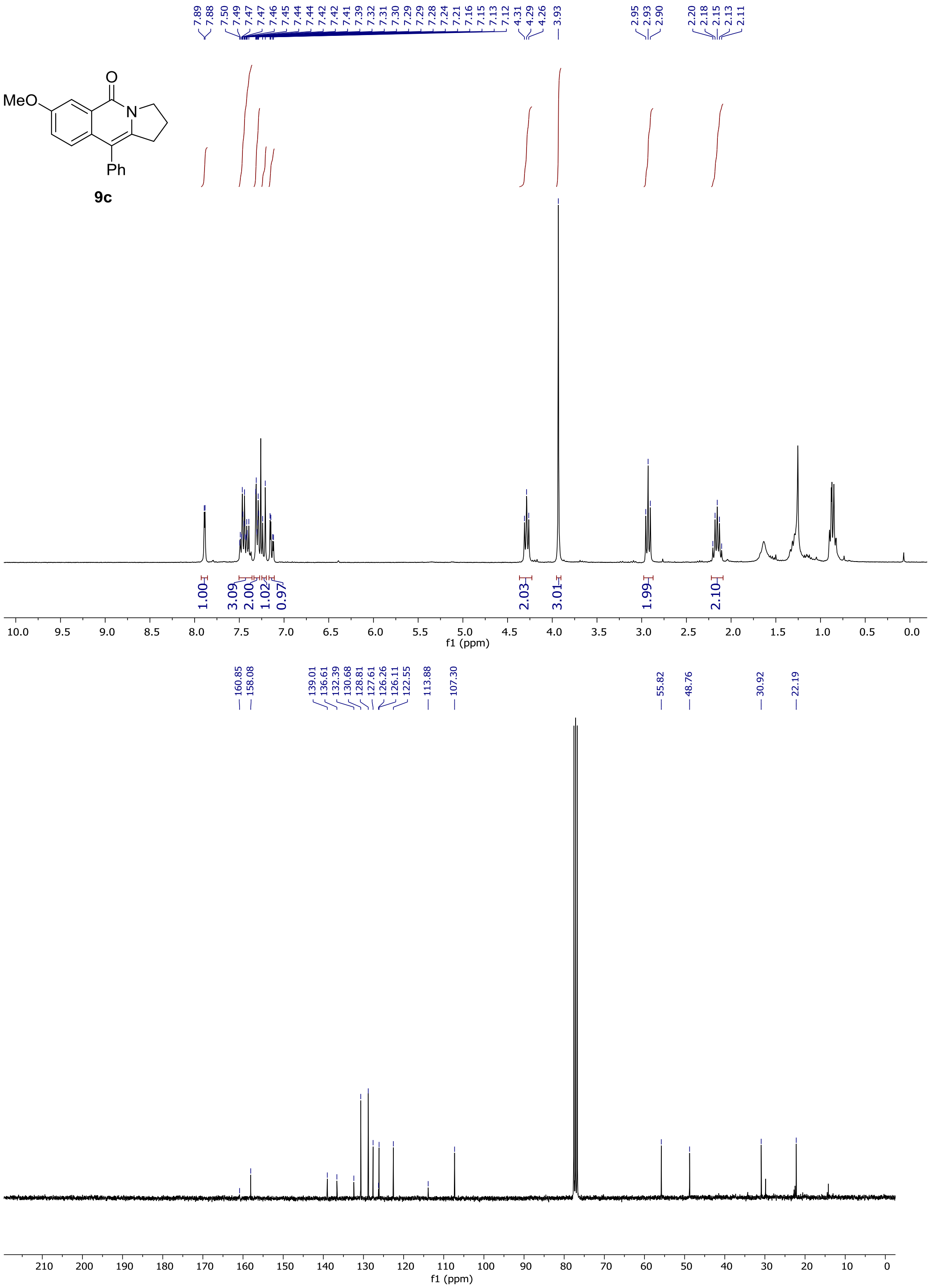

Figure SI-59. ^{1}H-NMR (300 MHz, $CDCl_3$) and ^{13}C {^{1}H} NMR (75 MHz, $CDCl_3$) spectra of compound **9c**

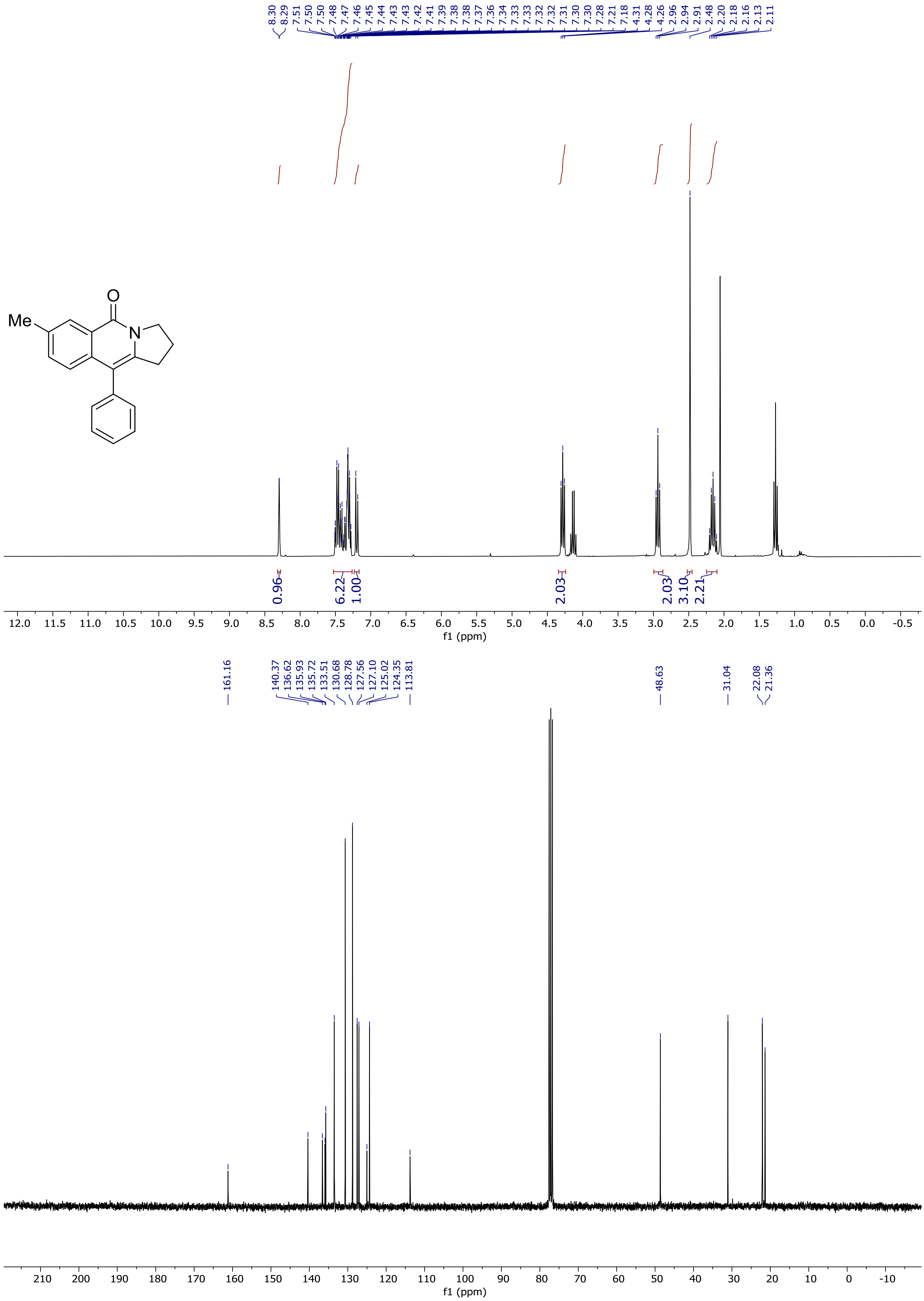

Figure SI-60. ^{1}H-NMR (300 MHz, $CDCl_3$) and ^{13}C $\{^{1}H\}$ NMR (75 MHz, $CDCl_3$) spectra of compound **9d**

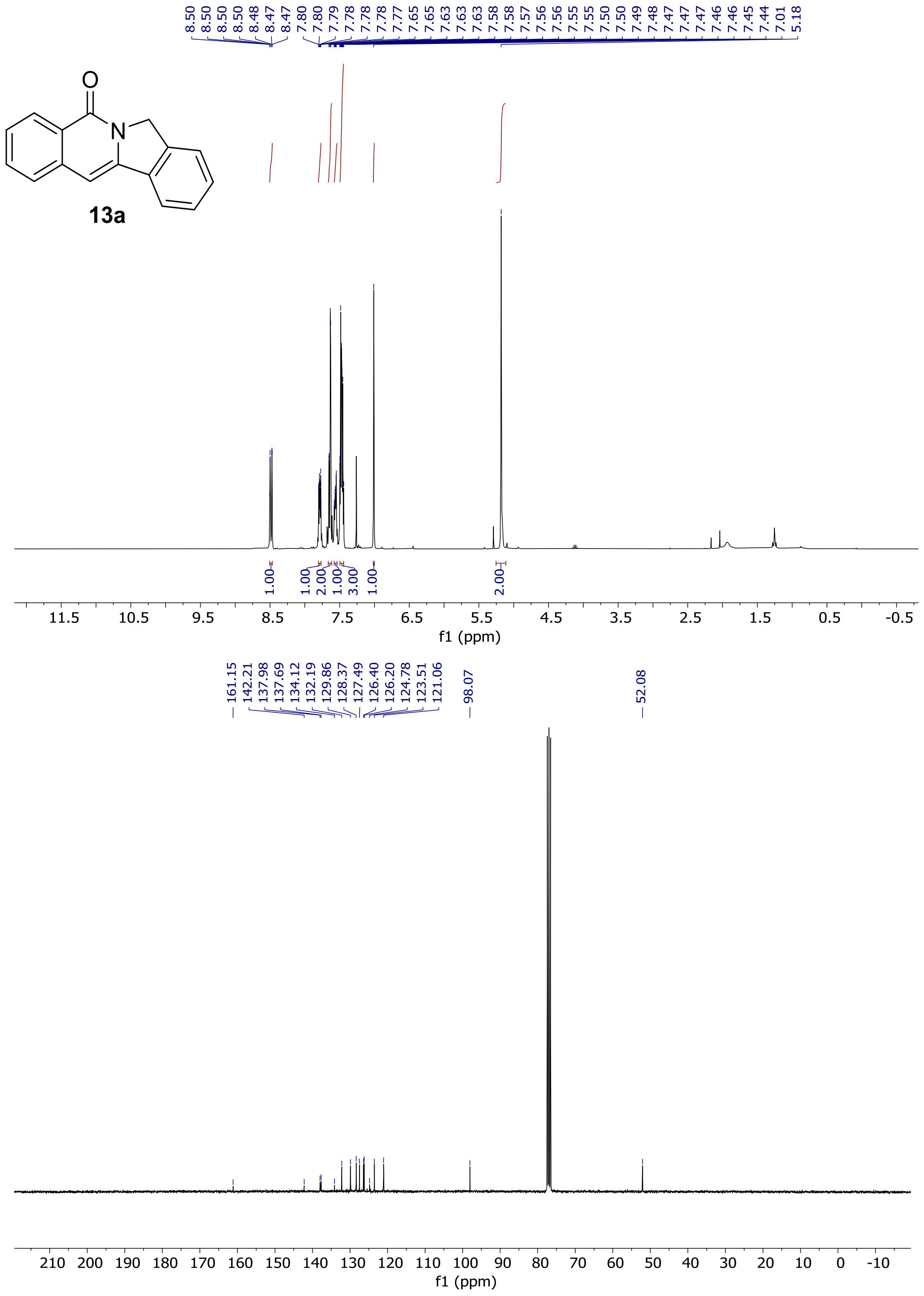

Figure SI-61. ^{1}H-NMR (300 MHz, $CDCl_3$) and ^{13}C {^{1}H} NMR (75 MHz, $CDCl_3$) spectra of compound **13a**

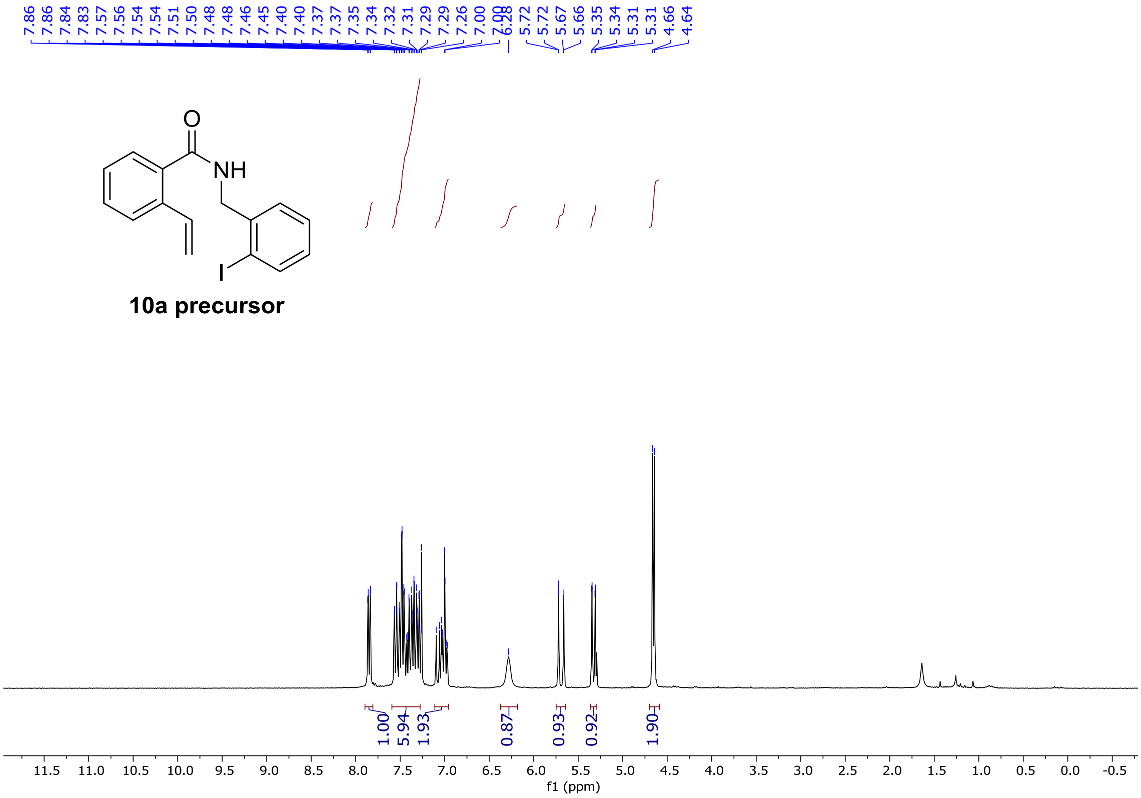

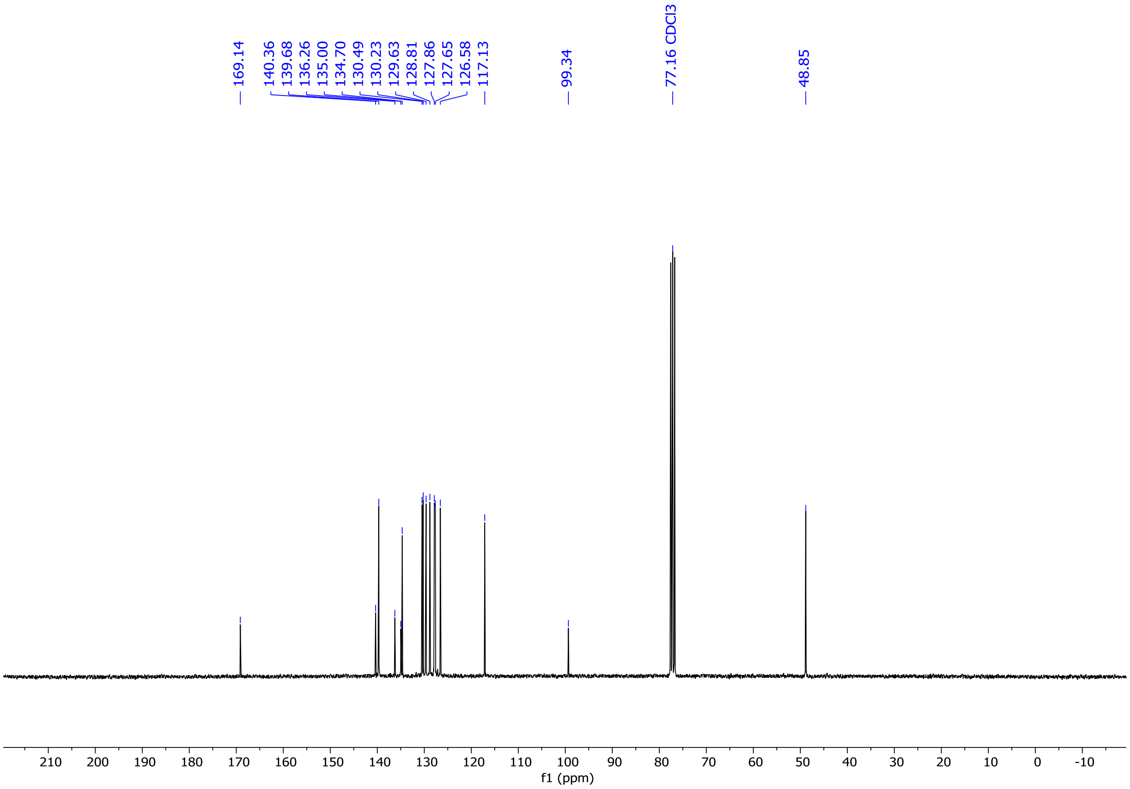

Figure SI-62. ^{1}H- NMR (300 MHz, $CDCl_3$) and ^{13}C {^{1}H} NMR (75 MHz, $CDCl_3$) spectra of *N*-(2-bromobenzyl)-2-vinylbenzamide (**10a precursor**)

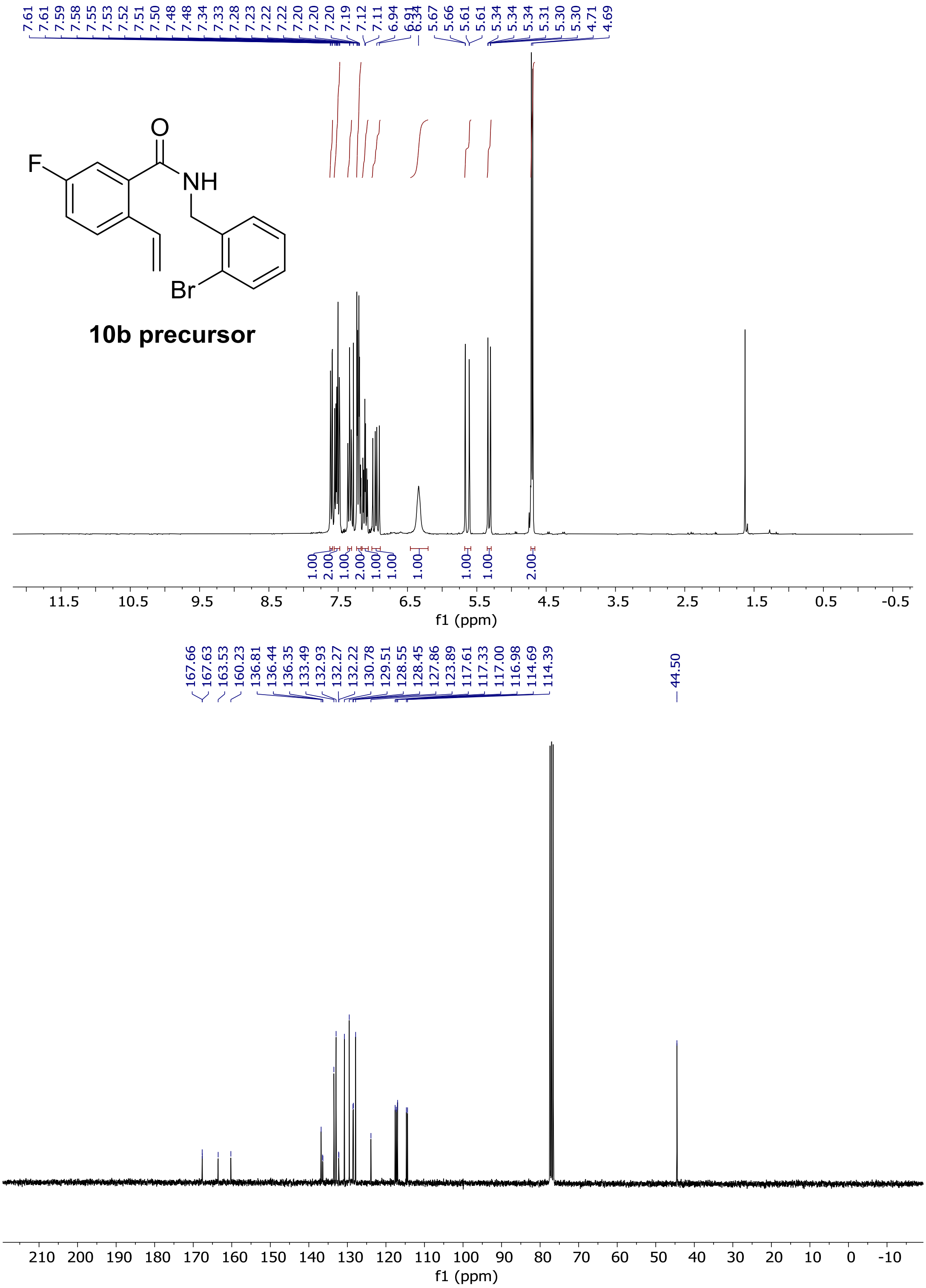

Figure SI-63. ^{1}H- NMR (300 MHz, $CDCl_3$) and ^{13}C {^{1}H} NMR (75 MHz, $CDCl_3$) spectra of *N*-(2-bromobenzyl)-5-fluoro-2-vinylbenzamide (**10b precursor**)

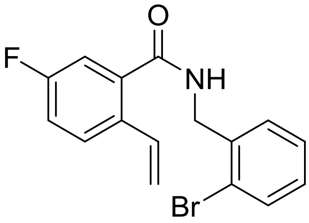

10b precursor

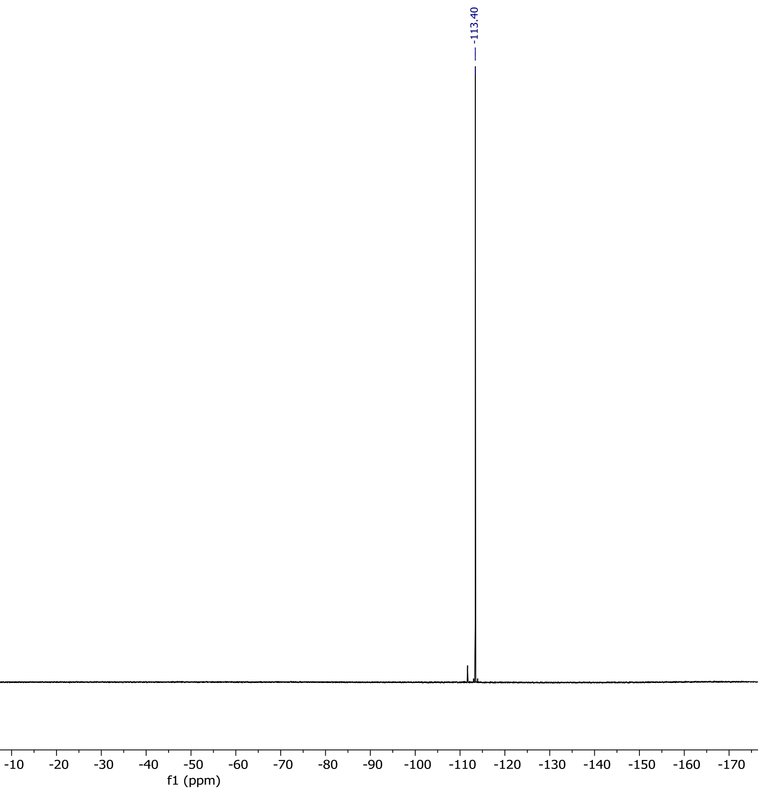

Figure SI-64. ^{19}F NMR (282 MHz, $CDCl_3$) spectrum of *N*-(2-bromobenzyl)-5-fluoro-2-vinylbenzamide (**10b precursor**)

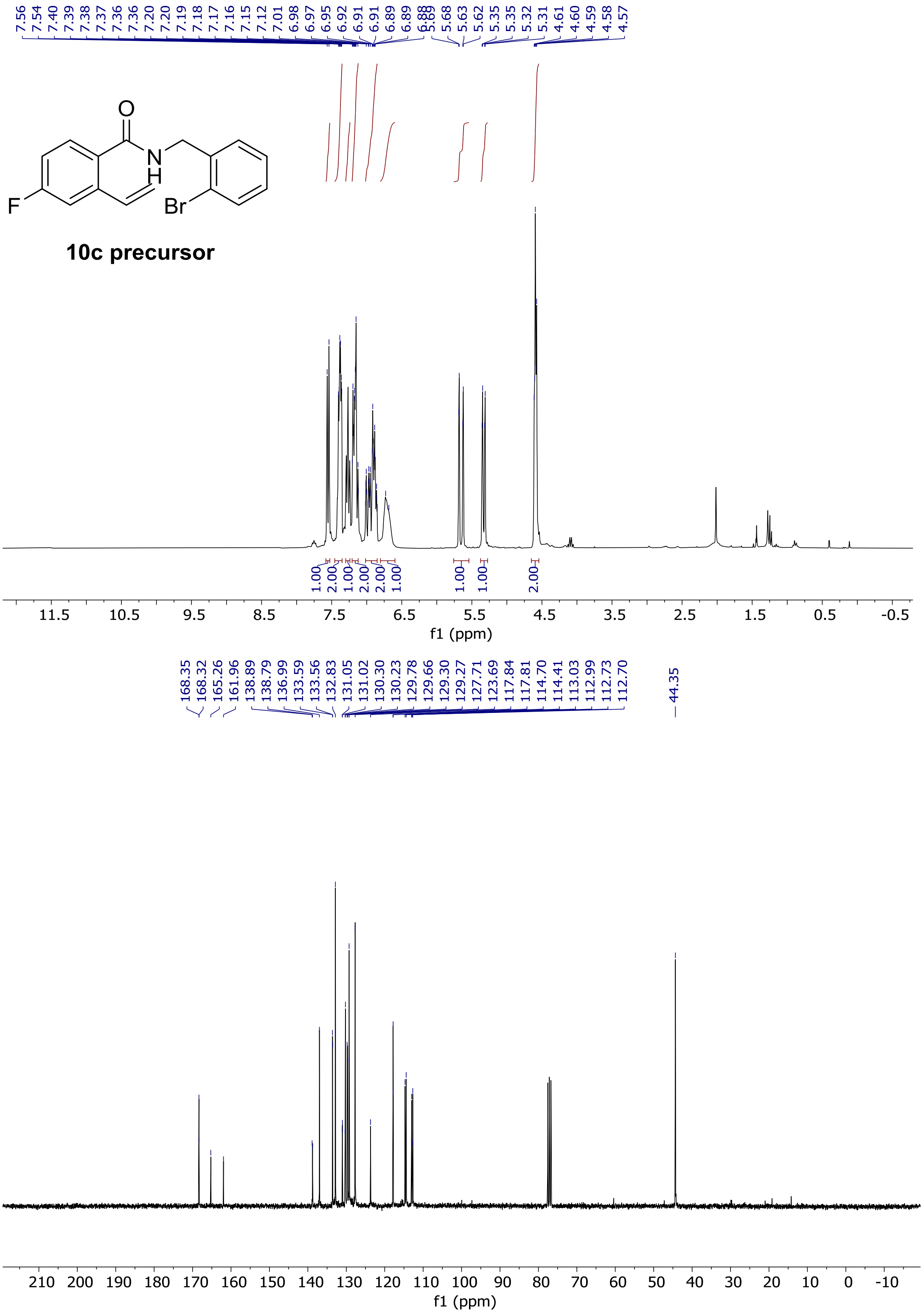

Figure SI-65. ^{1}H- NMR (300 MHz, $CDCl_3$) and ^{13}C {^{1}H} NMR (75 MHz, $CDCl_3$) spectra of *N*-(2-bromobenzyl)-4-fluoro-2-vinylbenzamide (**10c precursor**)

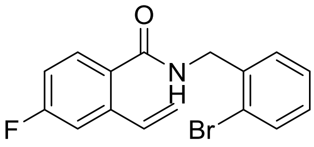

10c precursor

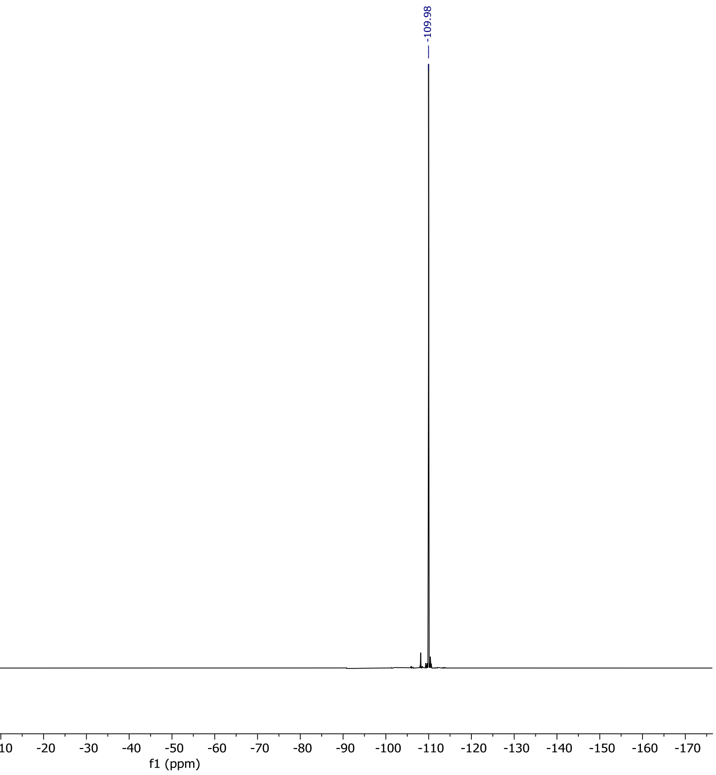

Figure SI-66. ^{19}F NMR (282 MHz, $CDCl_3$) spectrum of *N*-(2-bromobenzyl)-4-fluoro-2-vinylbenzamide (**10c precursor**)

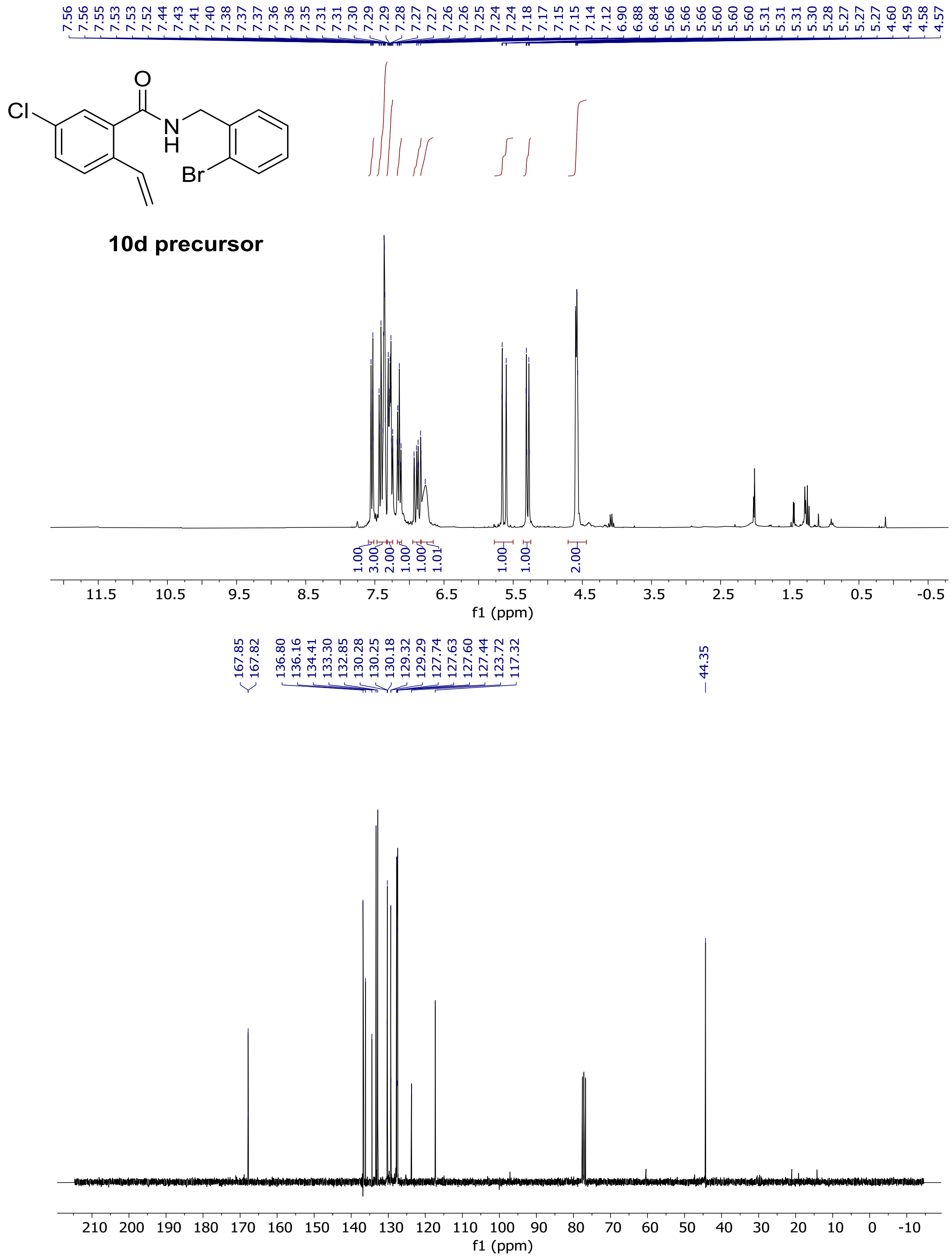

Figure SI-67. ^{1}H- NMR (300 MHz, $CDCl_3$) and ^{13}C {^{1}H} NMR (75 MHz, $CDCl_3$) spectra of *N*-(2-bromobenzyl)-5-chloro- 2-vinylbenzamide (**10d precursor**)

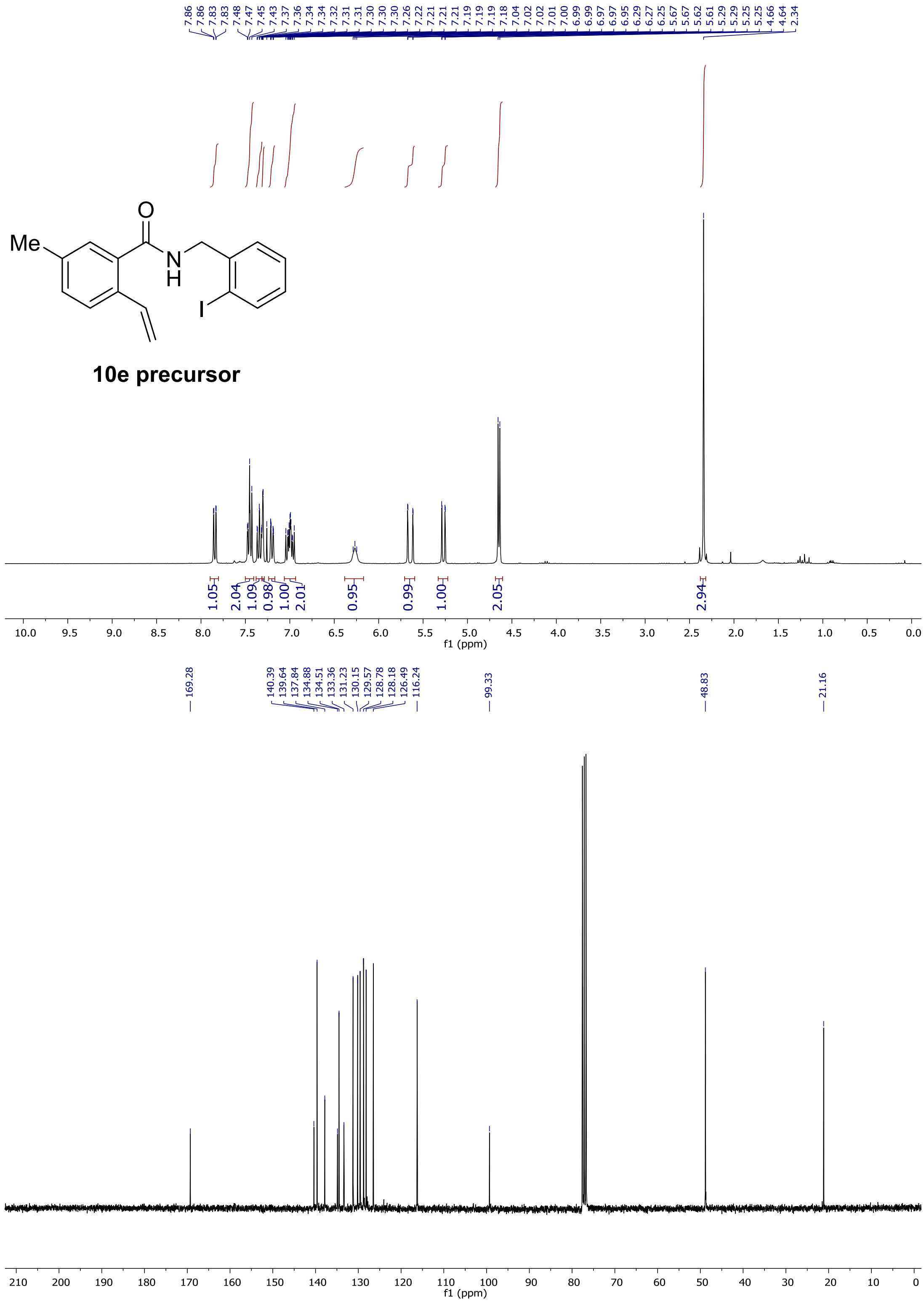

Figure SI-68. ^{1}H- NMR (300 MHz, $CDCl_3$) and ^{13}C {^{1}H} NMR (75 MHz, $CDCl_3$) spectra of *N*-(2-iodobenzyl)-5-methyl-2-vinylbenzamide (**10e precursor**)

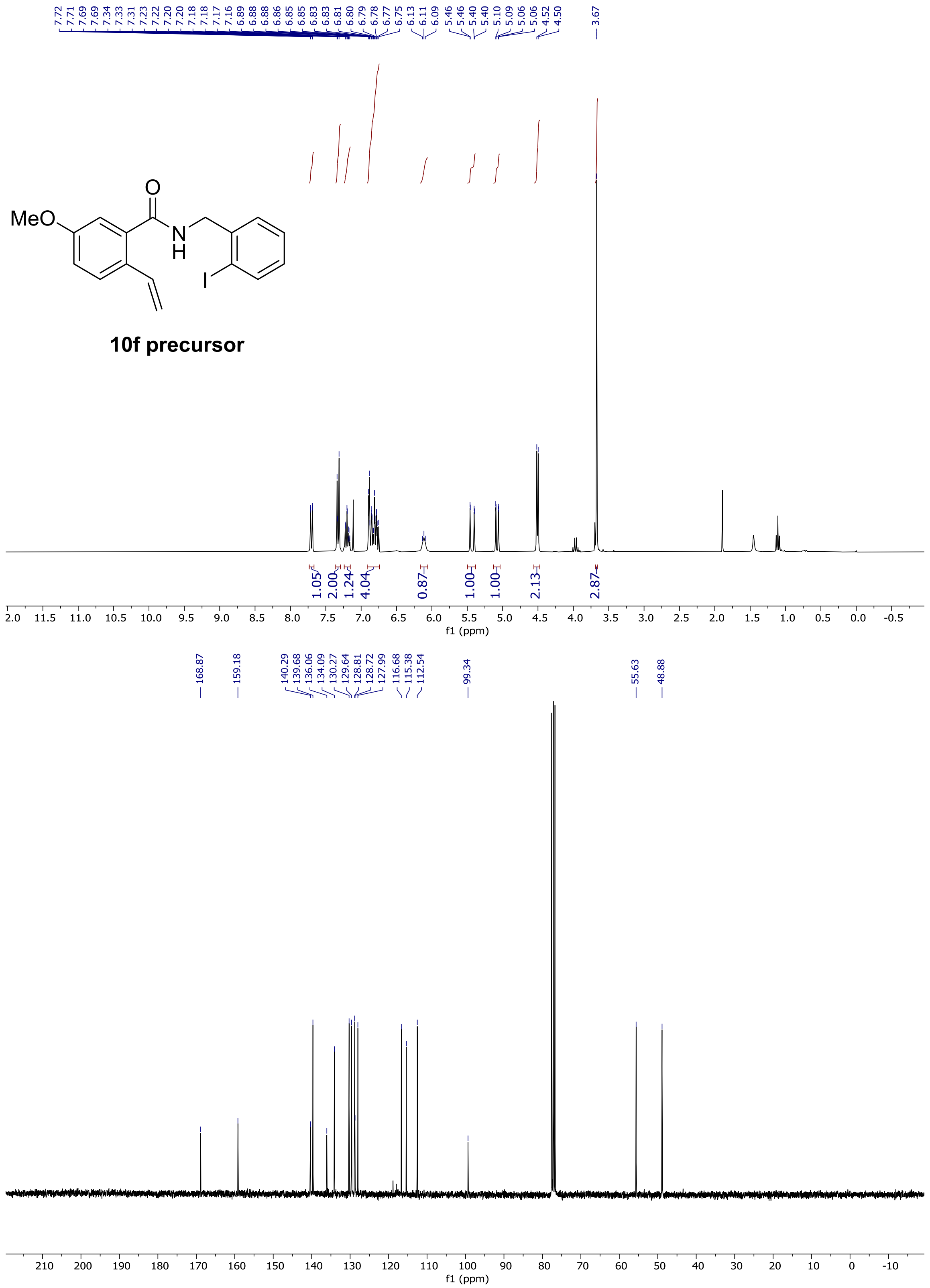

Figure SI-69. ^{1}H- NMR (300 MHz, $CDCl_3$) and ^{13}C {^{1}H} NMR (75 MHz, $CDCl_3$) spectra of *N*-(2-iodobenzyl)-5-methoxy-2-vinylbenzamide (**10f precursor**)

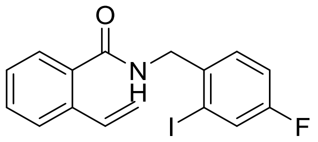

10g precursor

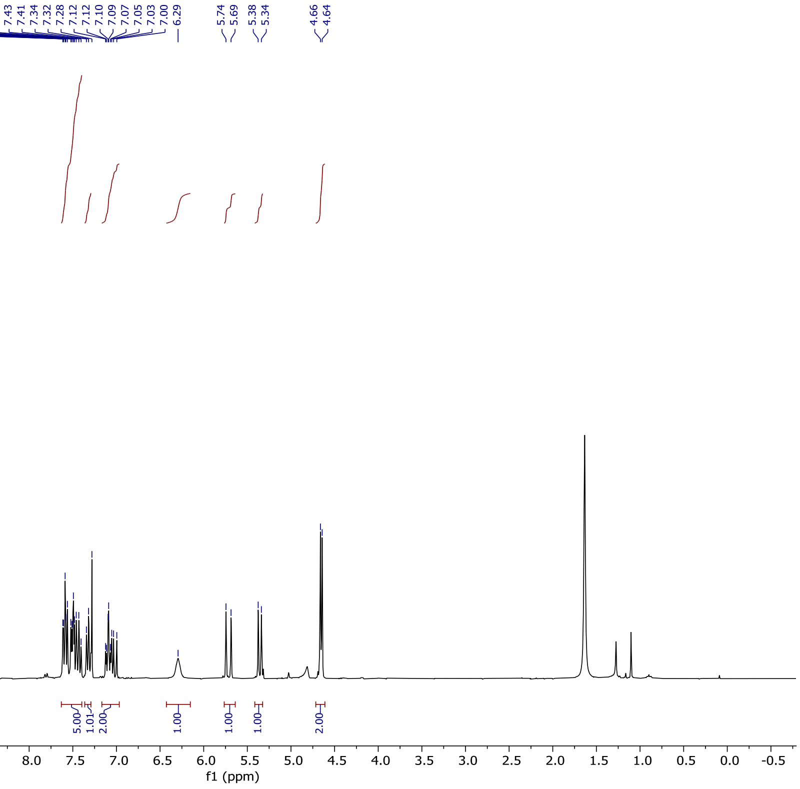

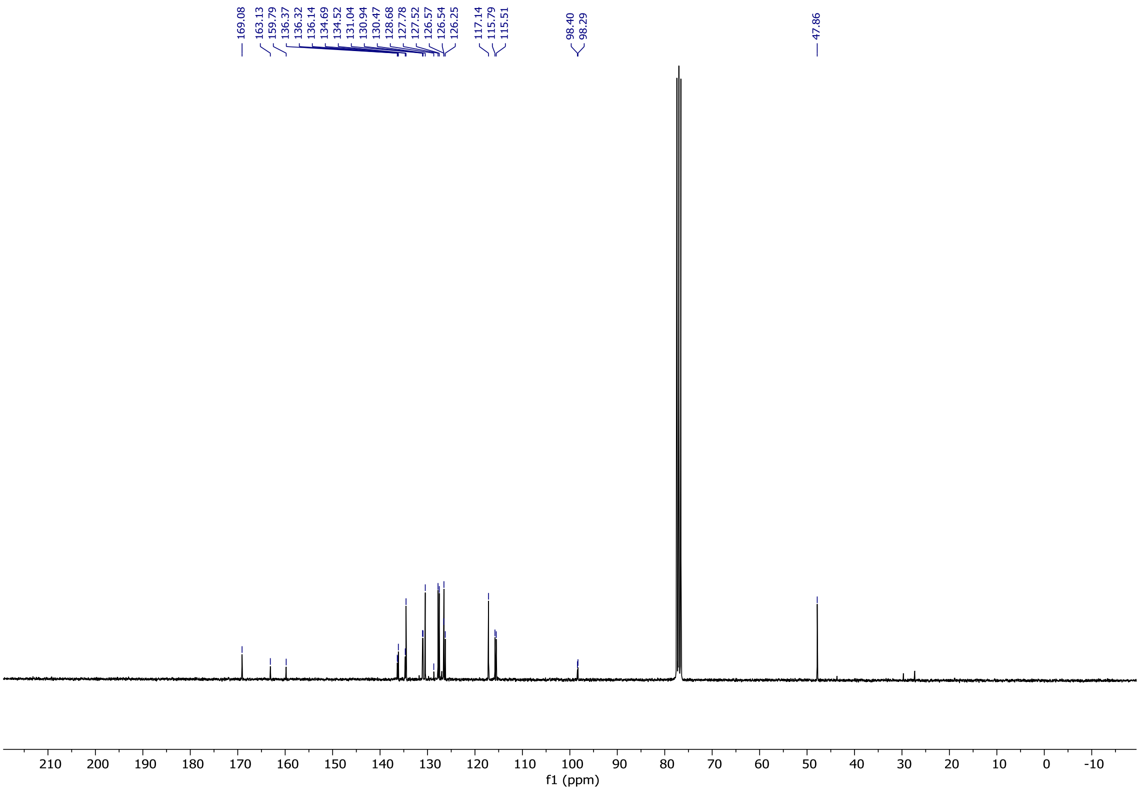

Figure SI-70. ^{1}H- NMR (300 MHz, $CDCl_3$) and $^{13}C\ \{^{1}H\}$ NMR (75 MHz, $CDCl_3$) spectra of *N*-(4-fluoro-2-iodobenzyl)-2-vinylbenzamide (**10g precursor**)

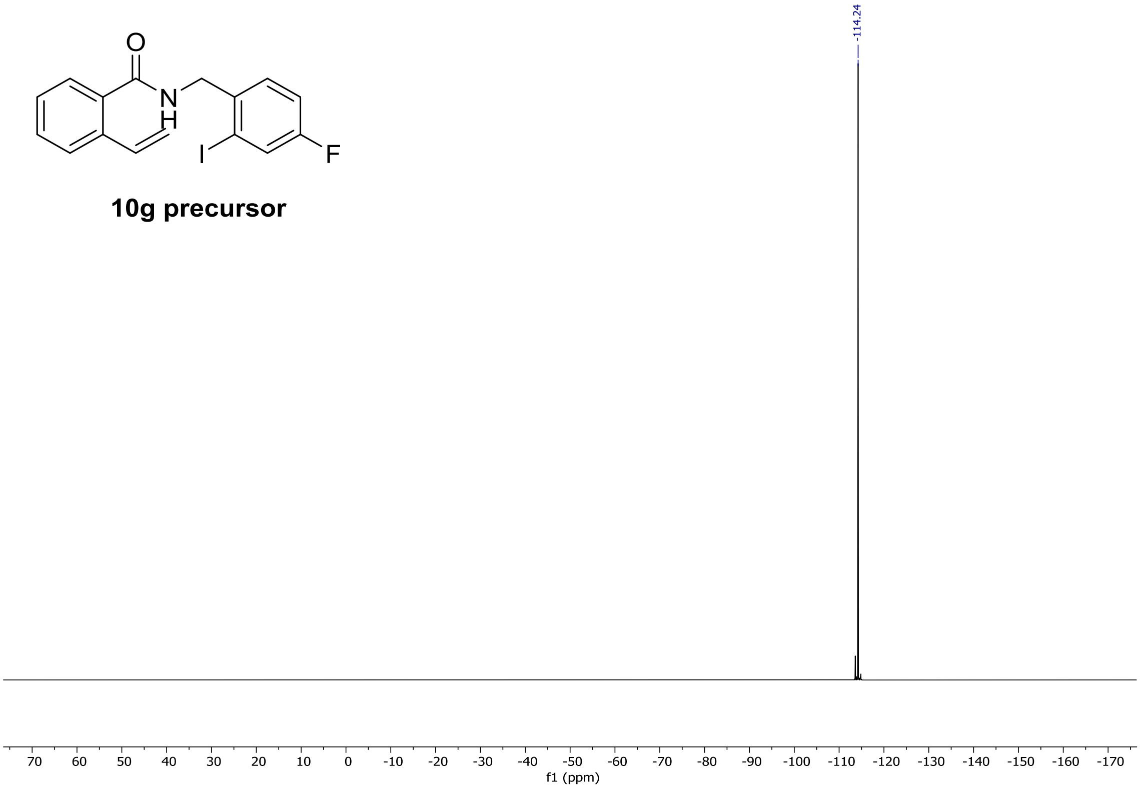

Figure SI-71. ^{19}F NMR (282 MHz, $CDCl_3$) spectrum of *N*-(4-fluoro-2-iodobenzyl)-2-vinylbenzamide (**10g precursor**)

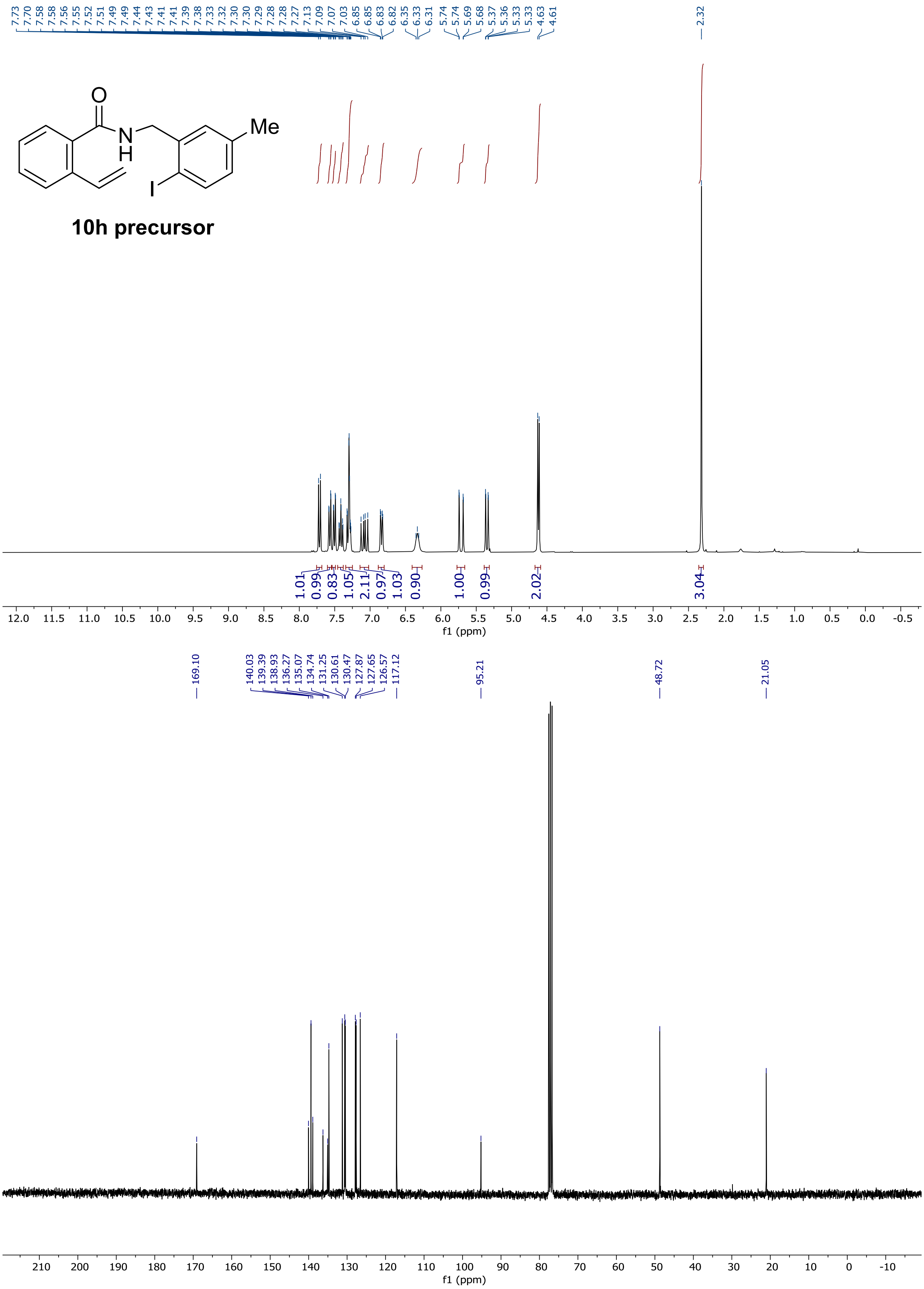

Figure SI-72. ^{1}H- NMR (300 MHz, $CDCl_3$) and ^{13}C {^{1}H} NMR (75 MHz, $CDCl_3$) spectra of *N*-(2-Iodo-5-methylbenzyl)-2-vinylbenzamide (**10h precursor**)

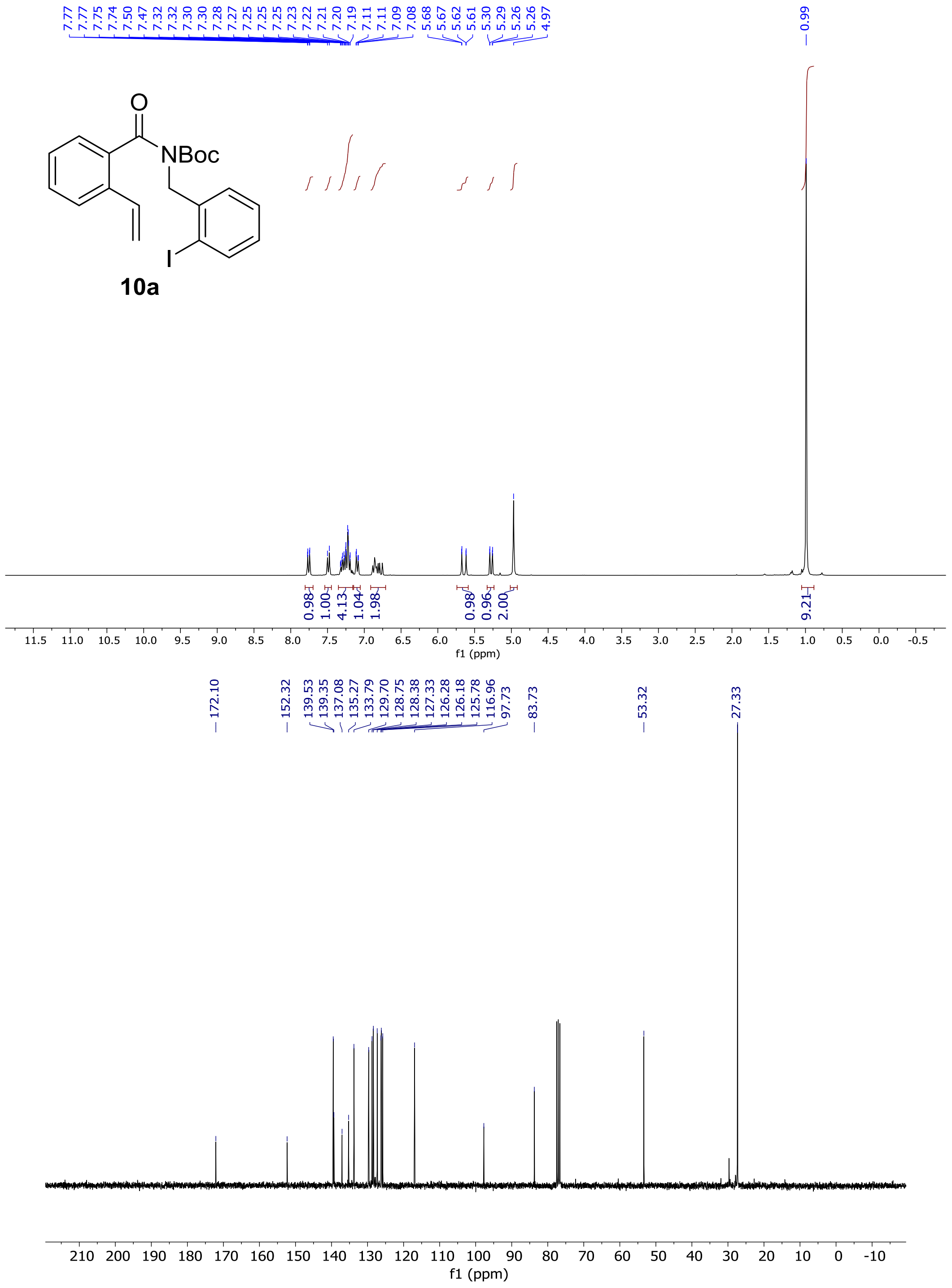

Figure SI-73. ^{1}H- NMR (300 MHz, $CDCl_3$) and ^{13}C {^{1}H} NMR (75 MHz, $CDCl_3$) spectra of compound **10a**

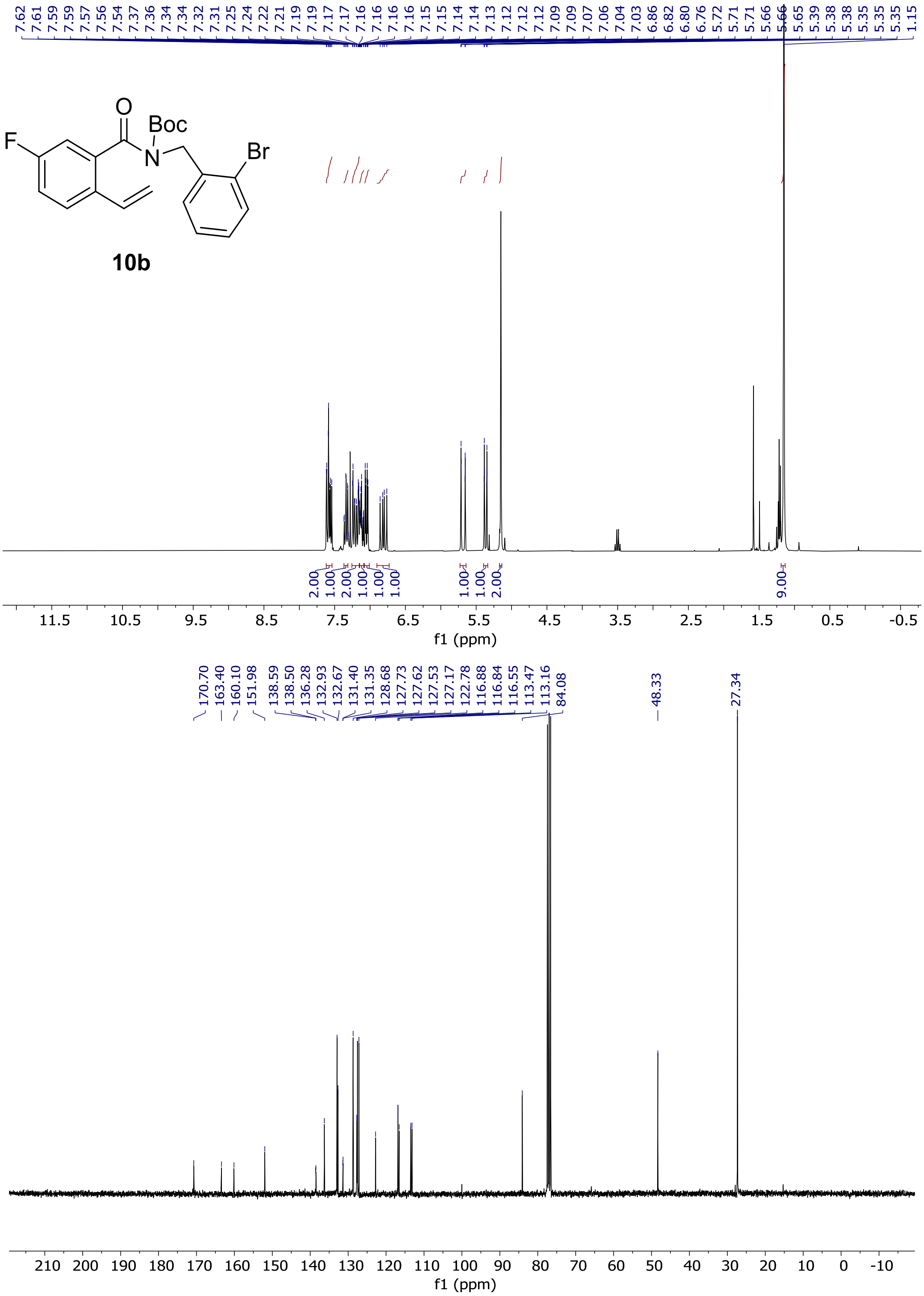

Figure SI-74. ^{1}H- NMR (300 MHz, $CDCl_3$) and ^{13}C {^{1}H} NMR (75 MHz, $CDCl_3$) spectra of compound **10b**

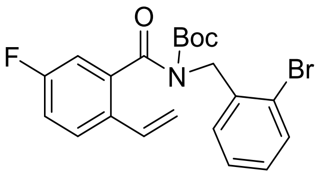

10b

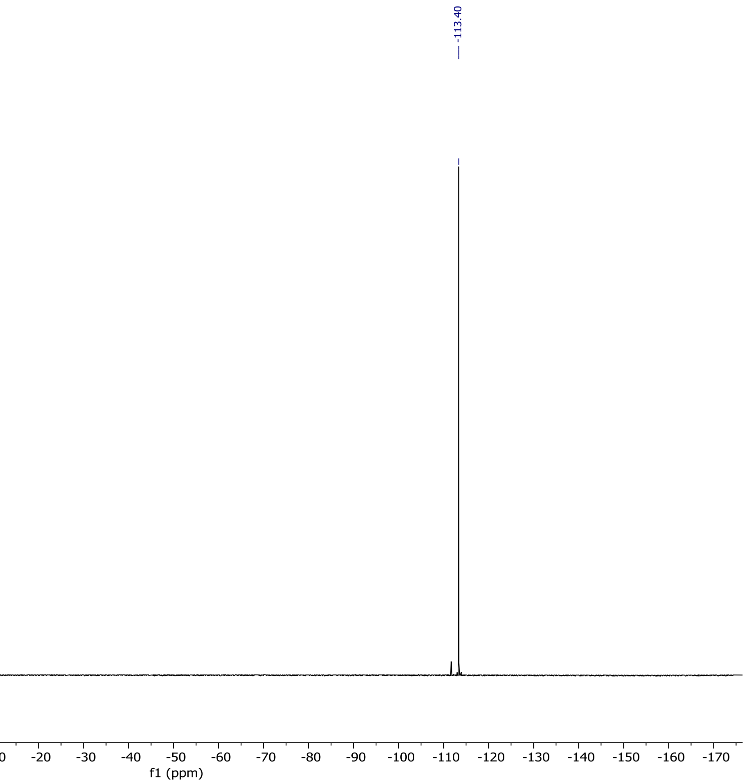

Figure SI-75. ^{19}F NMR (282 MHz, $CDCl_3$) spectrum of compound **10b**

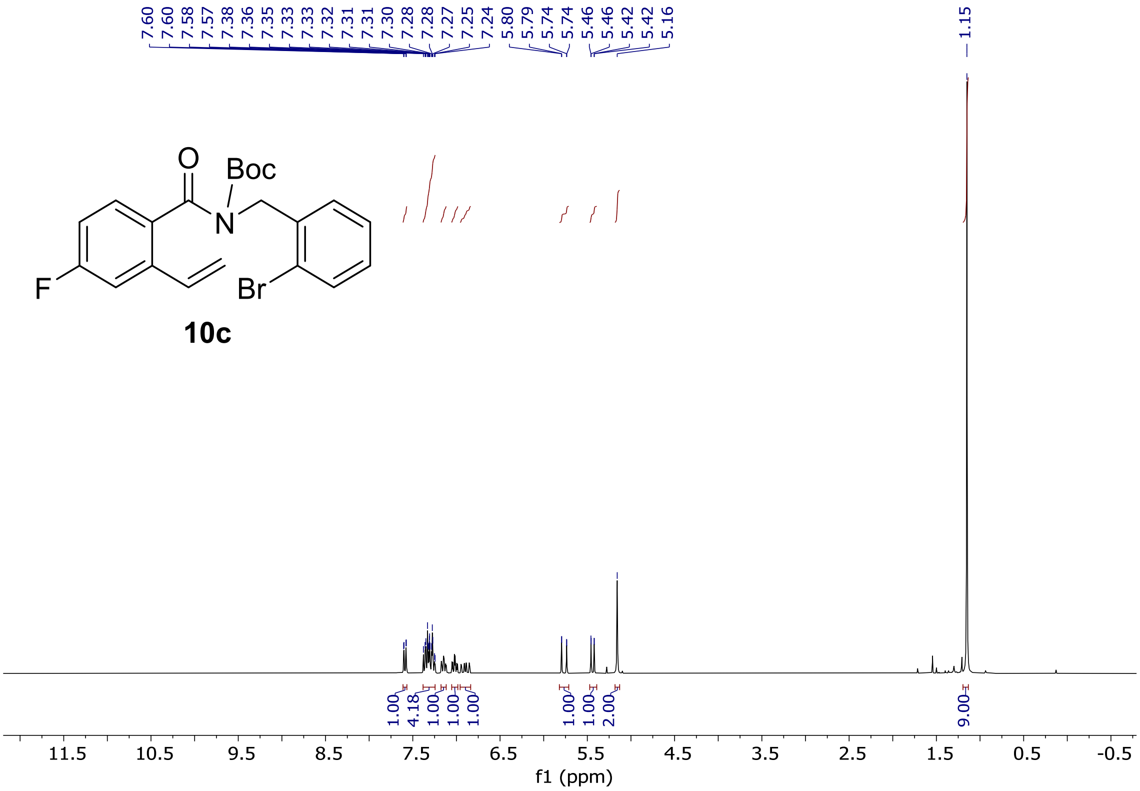

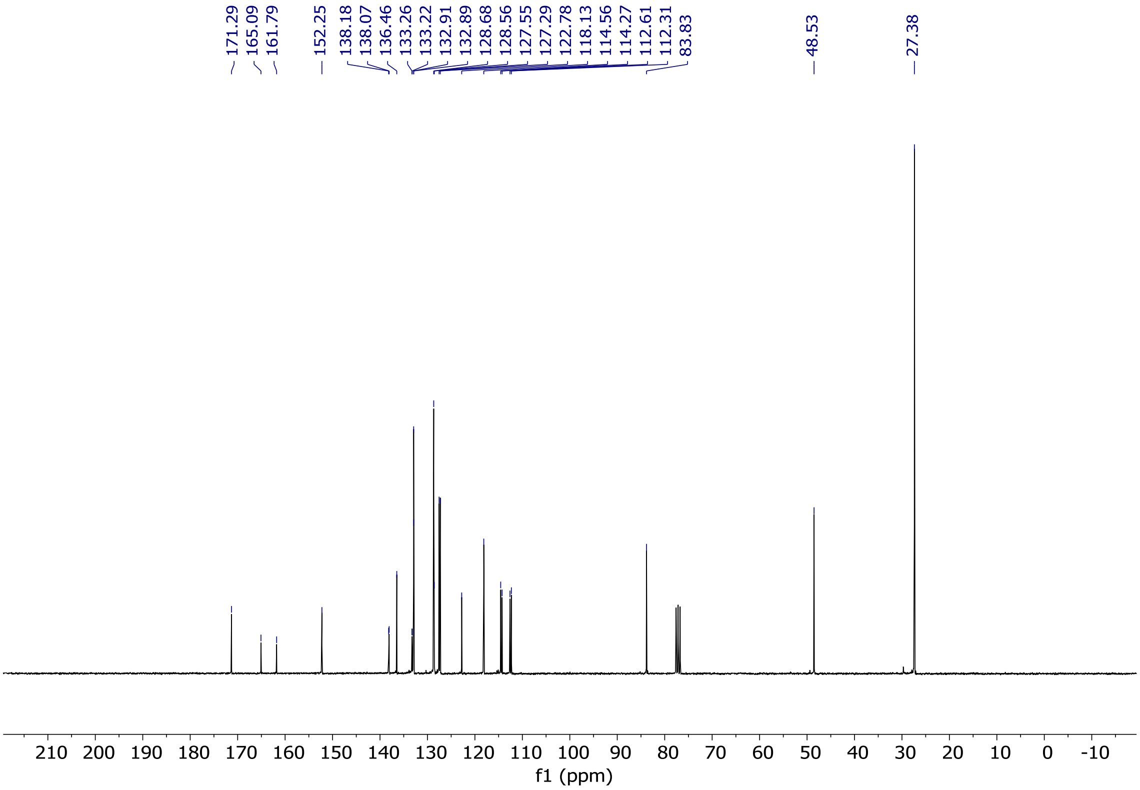

Figure SI-76. ^{1}H- NMR (300 MHz, $CDCl_3$) and ^{13}C {^{1}H} NMR (75 MHz, $CDCl_3$) spectra of compound **10c**

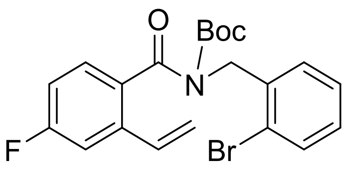

10c

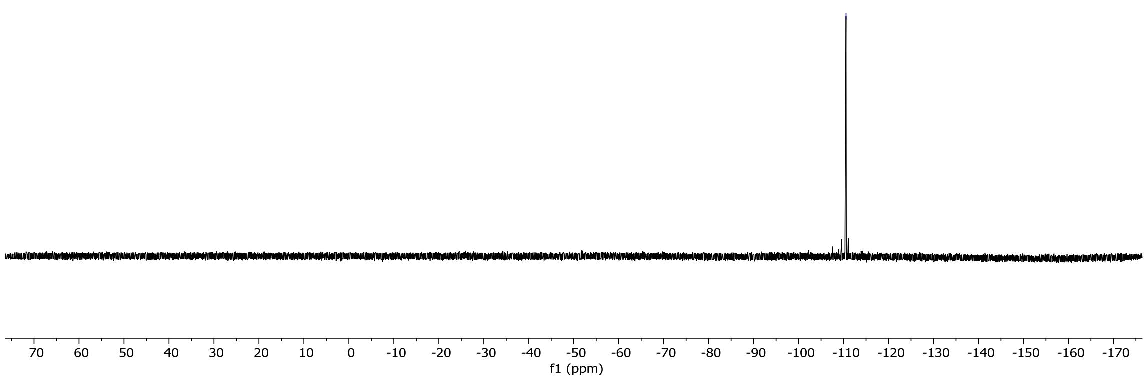

Figure SI-77. ^{19}F NMR (282 MHz, $CDCl_3$) spectrum of compound **10c**

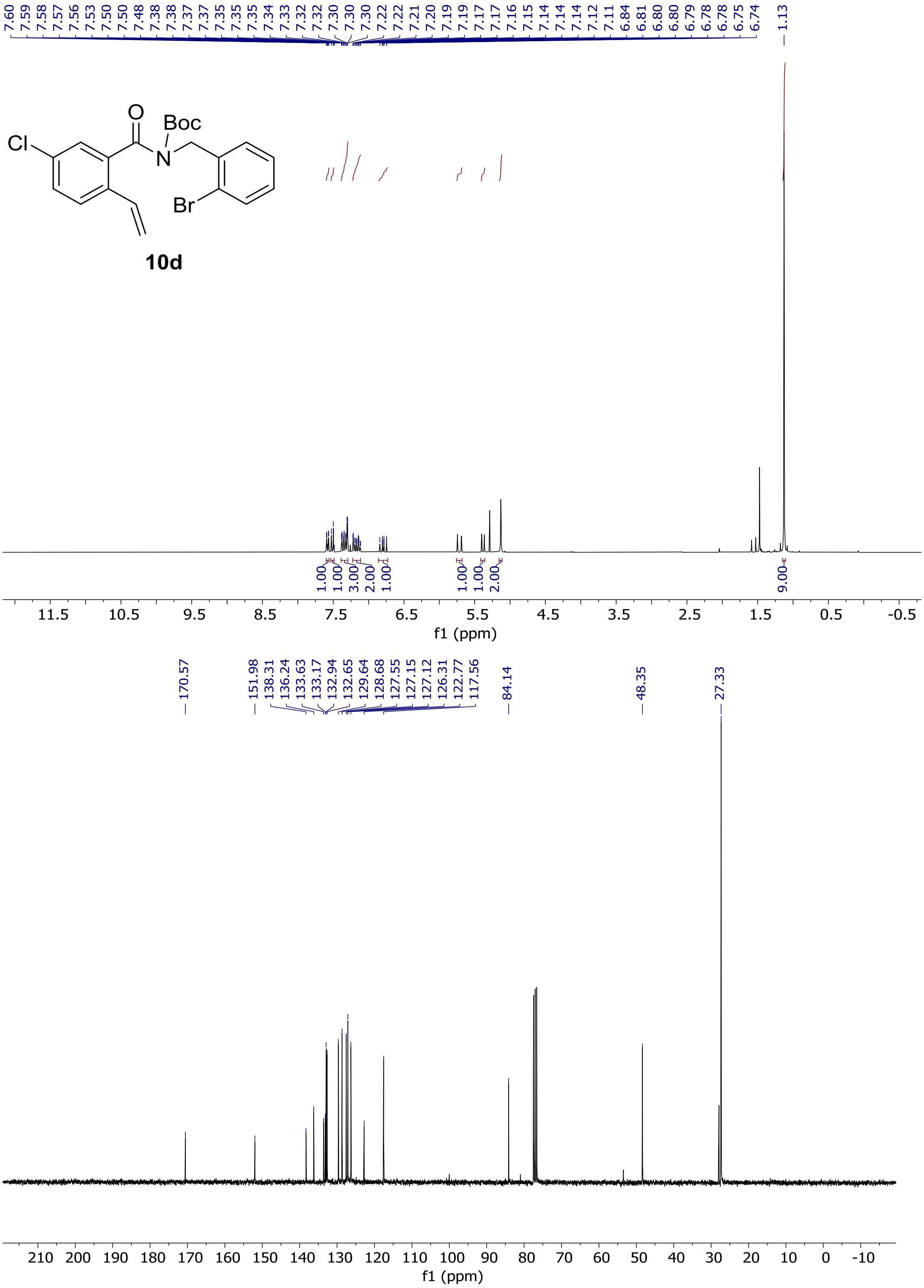

Figure SI-78. ^{1}H- NMR (300 MHz, $CDCl_3$) and ^{13}C {^{1}H} NMR (75 MHz, $CDCl_3$) spectra of compound **10d**

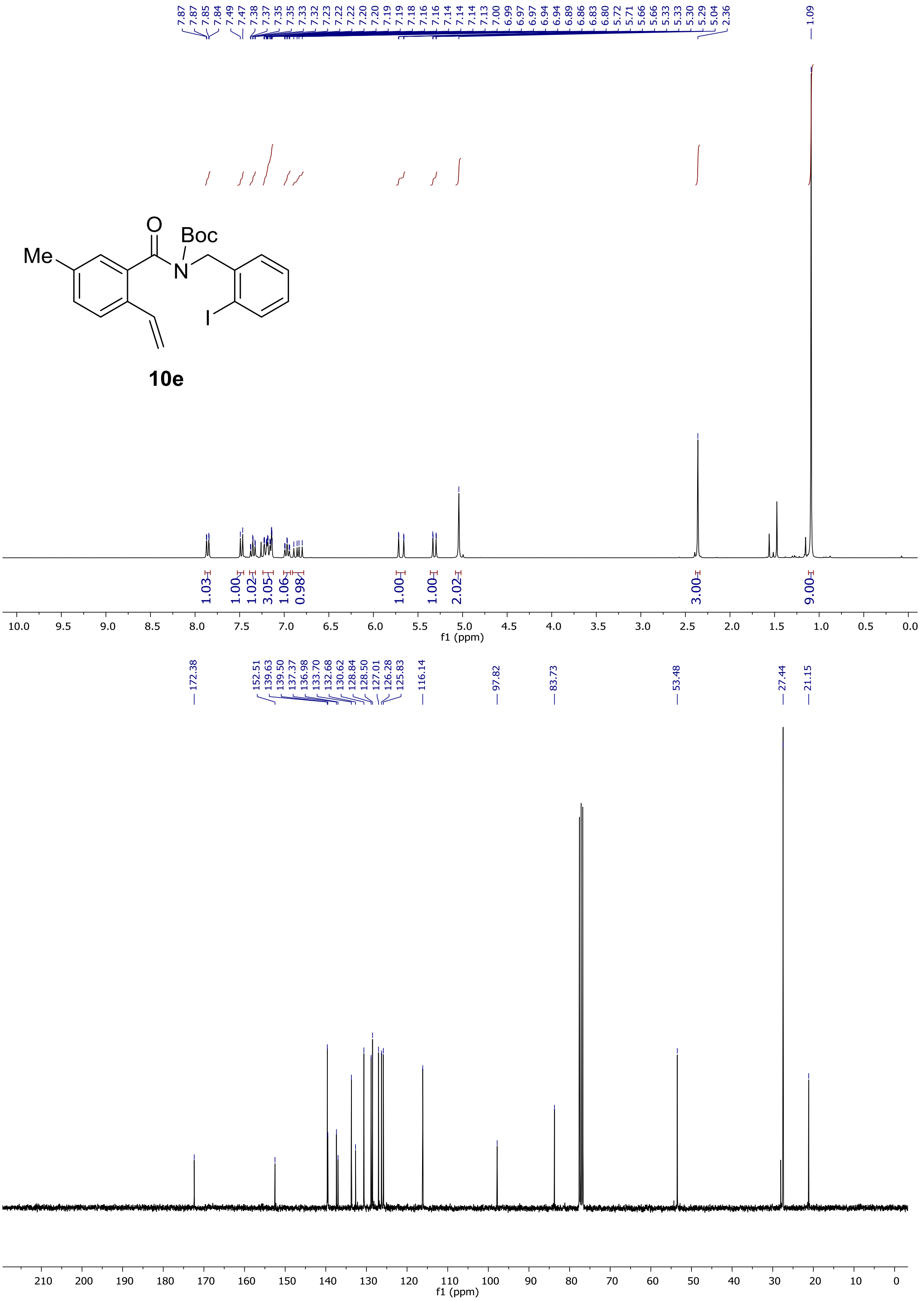

Figure SI-79. ^{1}H- NMR (300 MHz, $CDCl_3$) and ^{13}C {^{1}H} NMR (75 MHz, $CDCl_3$) spectra of compound **10e**

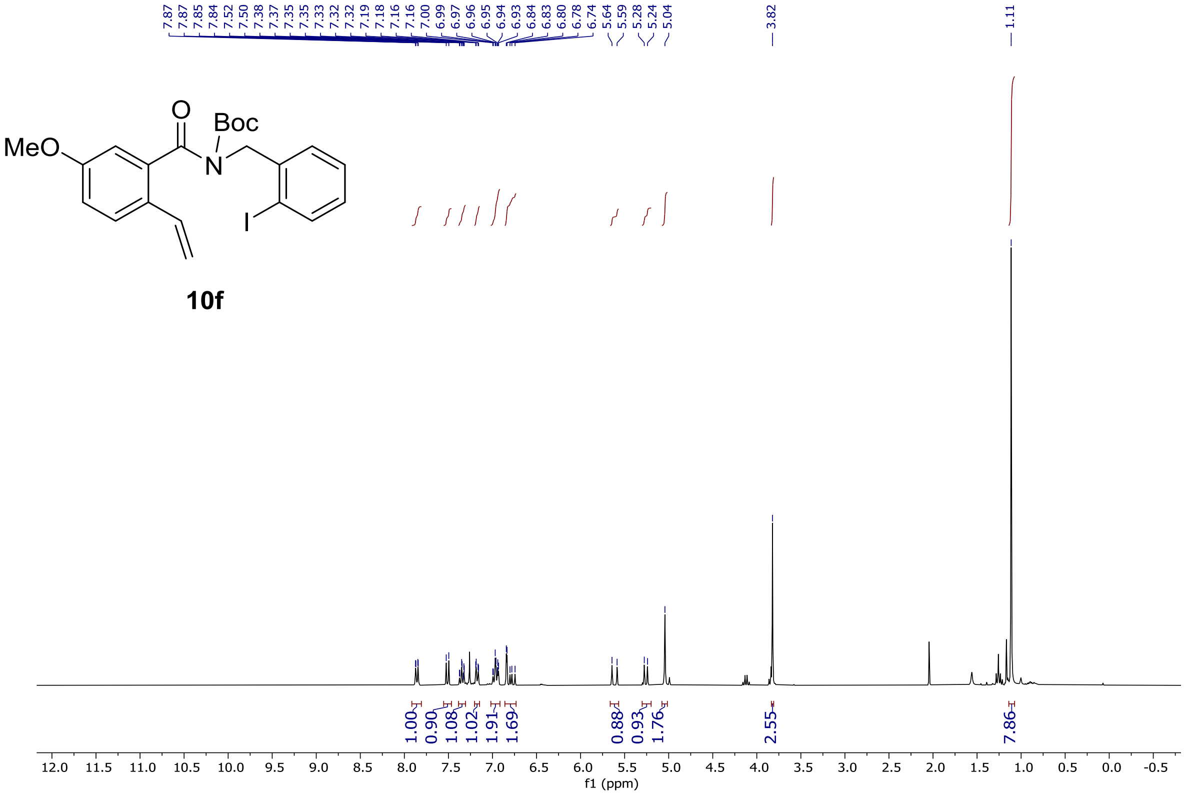

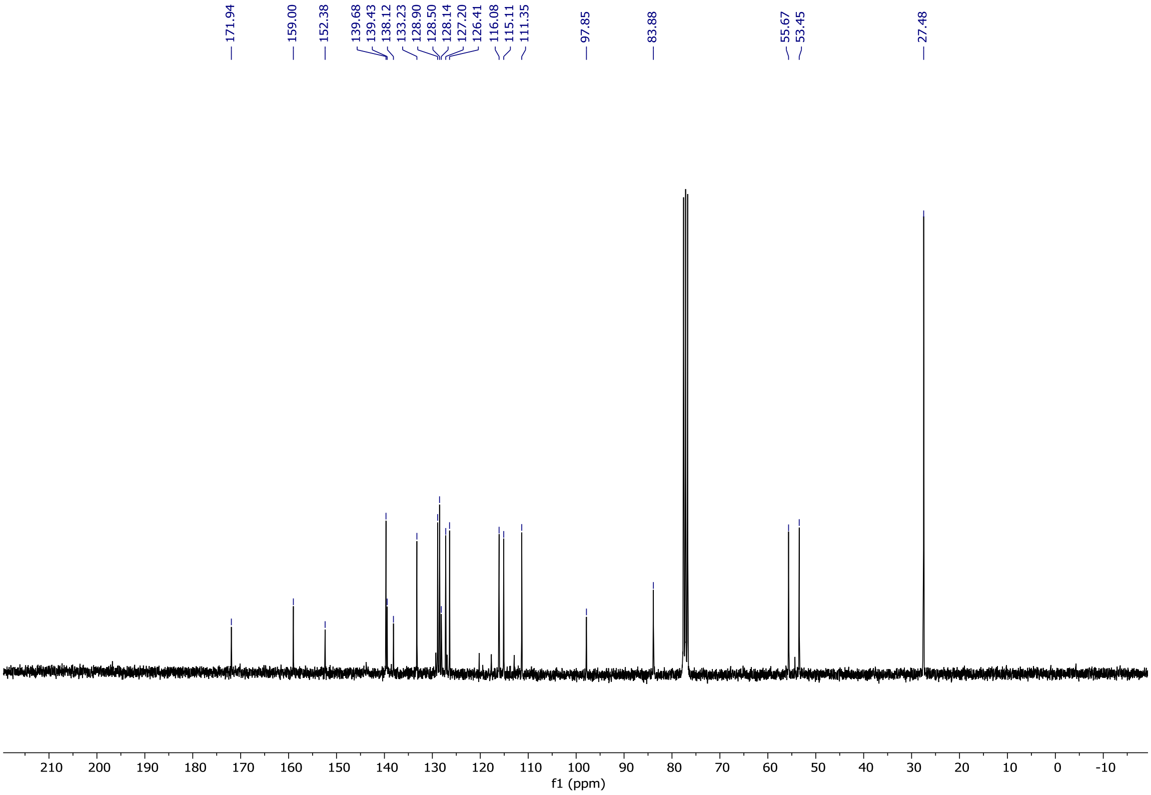

Figure SI-80. ^{1}H- NMR (300 MHz, $CDCl_3$) and ^{13}C {^{1}H} NMR (75 MHz, $CDCl_3$) spectra of compound **10f**

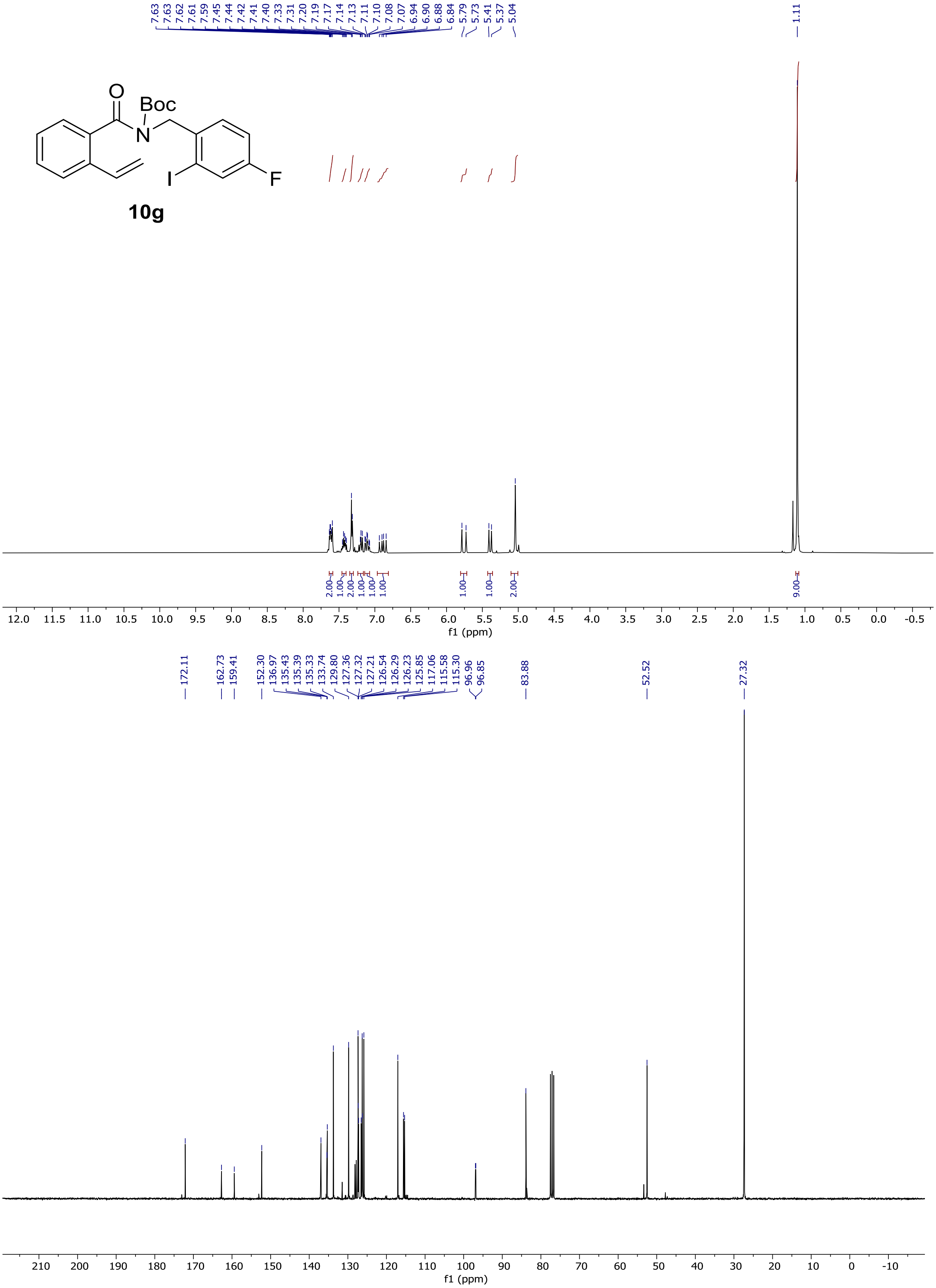

Figure SI-81. ^{1}H- NMR (300 MHz, $CDCl_3$) and ^{13}C {^{1}H} NMR (75 MHz, $CDCl_3$) spectra of compound **10g**

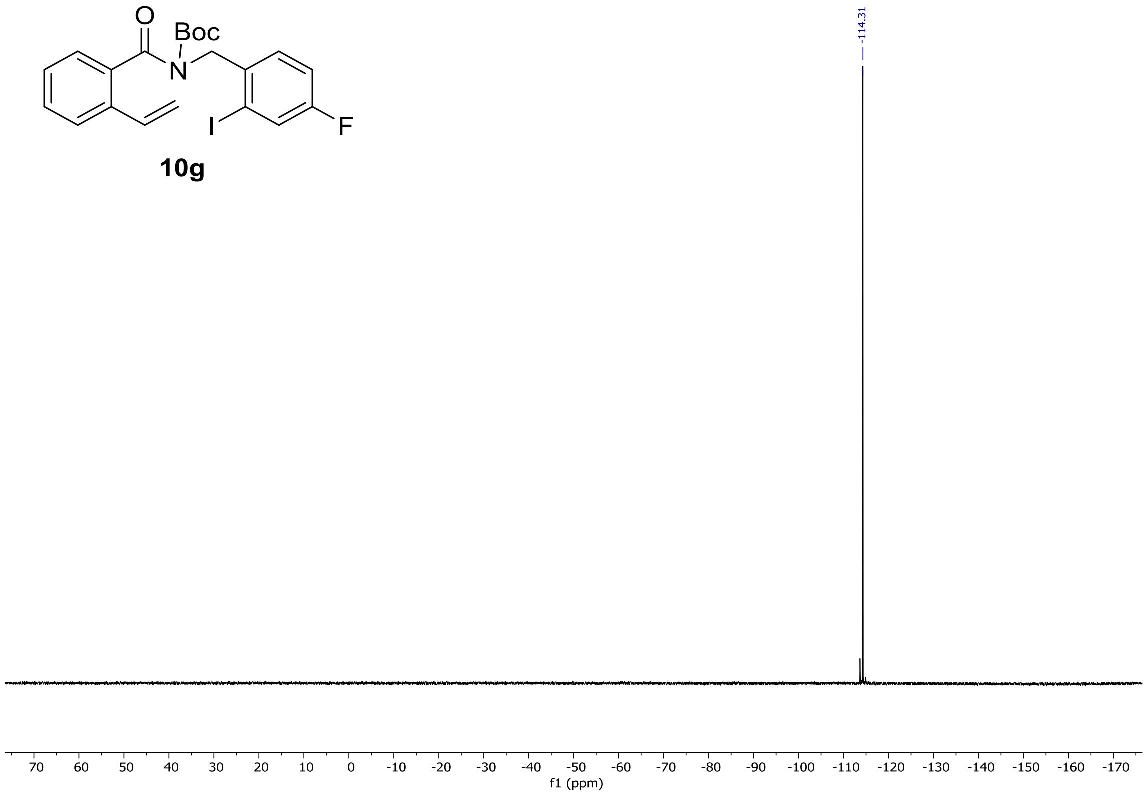

Figure SI-82. ^{19}F NMR (282 MHz, $CDCl_3$) spectrum of compound **10g**

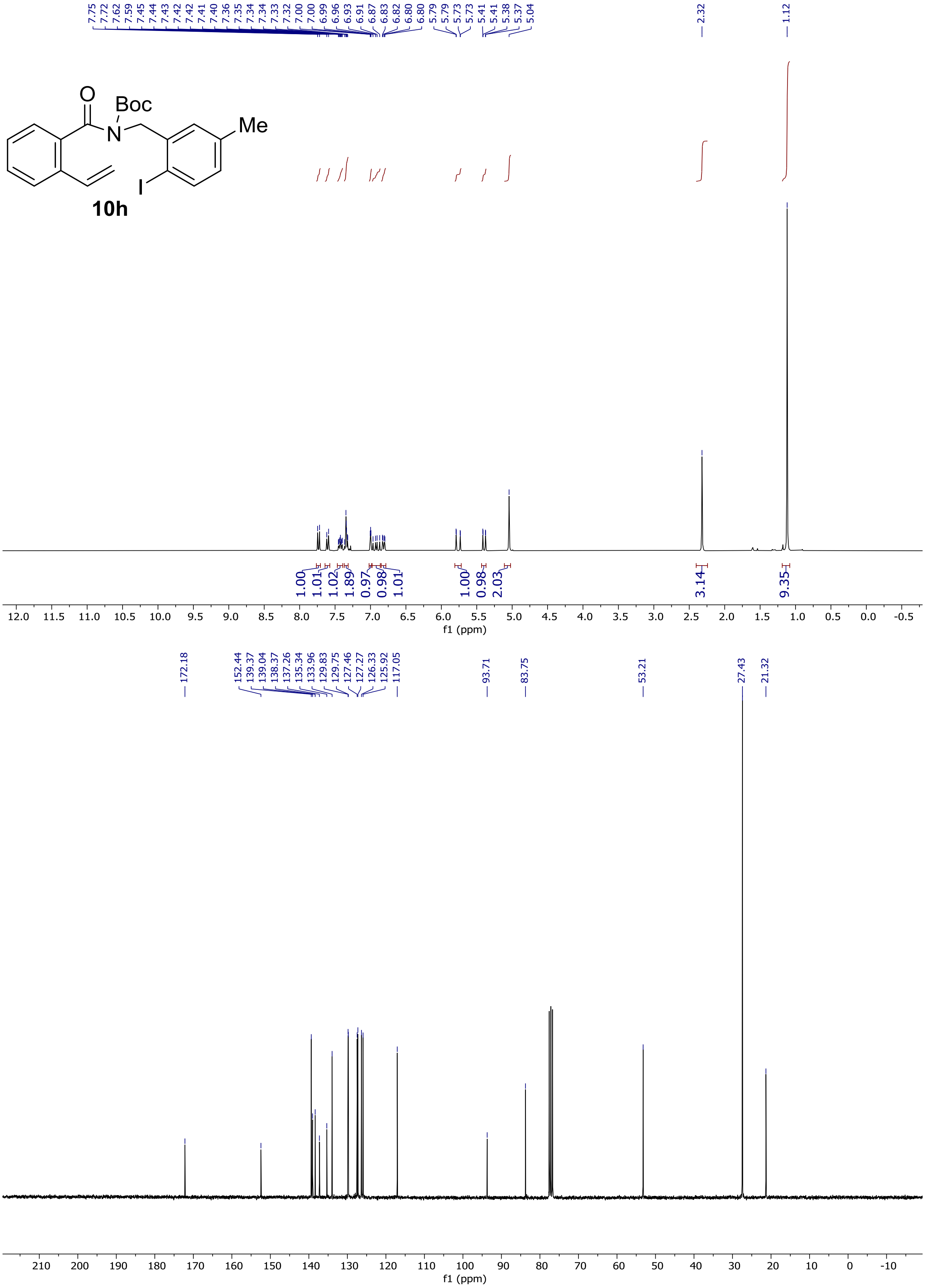

Figure SI-83. ^{1}H- NMR (300 MHz, $CDCl_3$) and ^{13}C {^{1}H} NMR (75 MHz, $CDCl_3$) spectra of compound **10h**

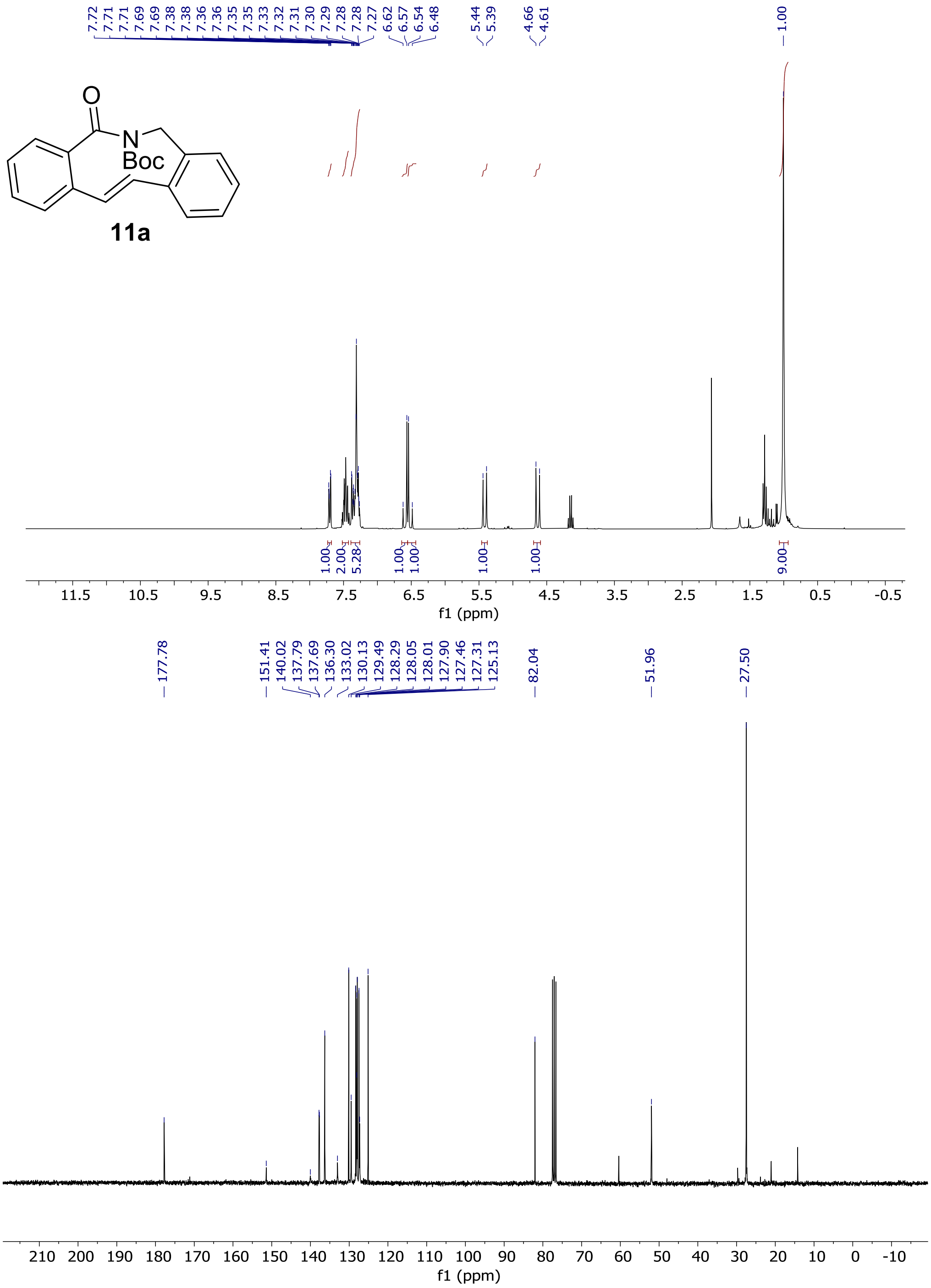

Figure SI-84. ^{1}H- NMR (300 MHz, $CDCl_3$) and ^{13}C {^{1}H} NMR (75 MHz, $CDCl_3$) spectra of compound **11a**

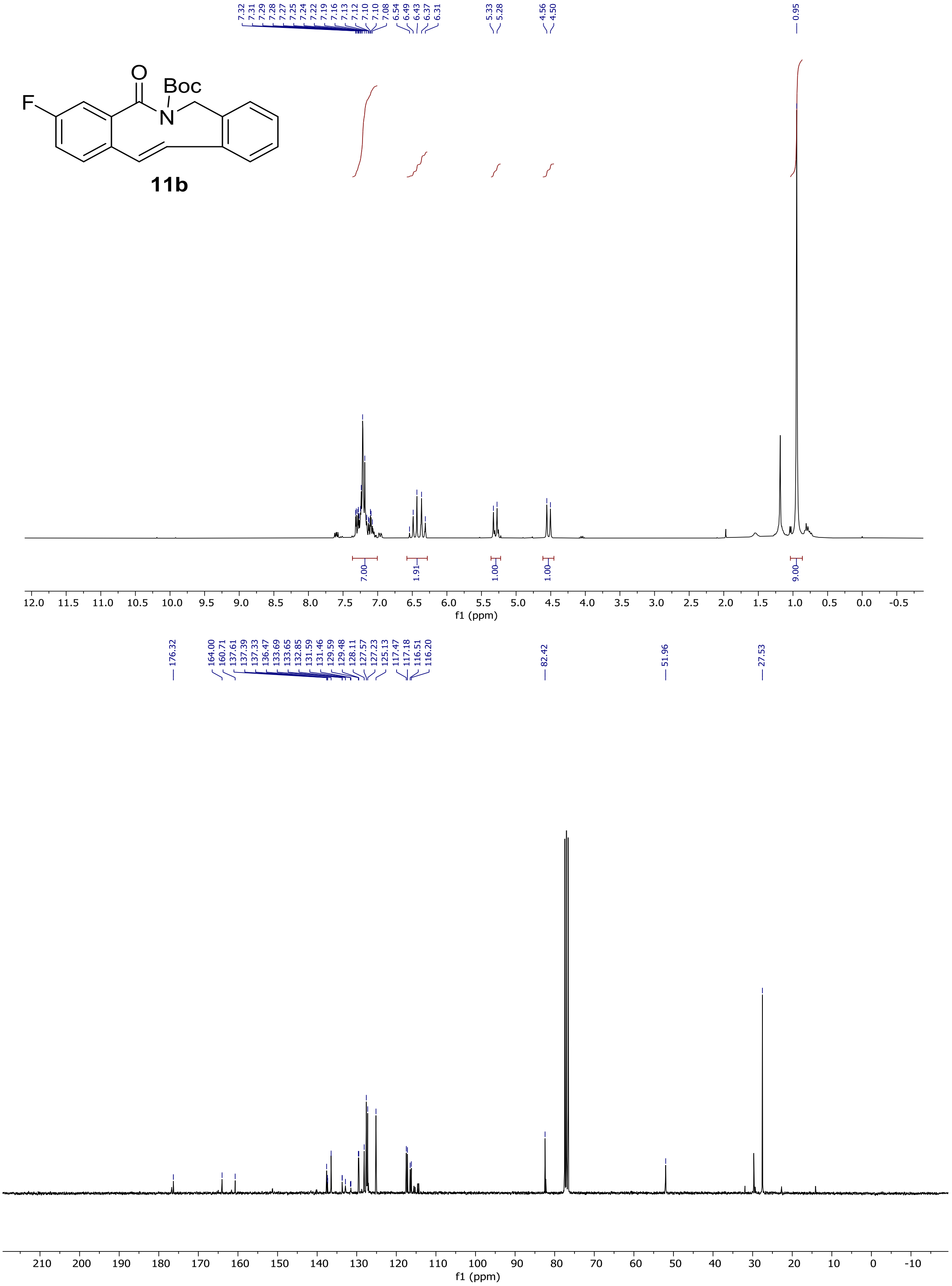

Figure SI-85. ^{1}H- NMR (300 MHz, $CDCl_3$) and ^{13}C {^{1}H} NMR (75 MHz, $CDCl_3$) spectra of compound **11b**

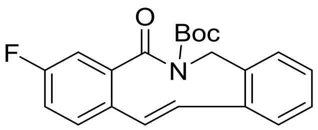

11b

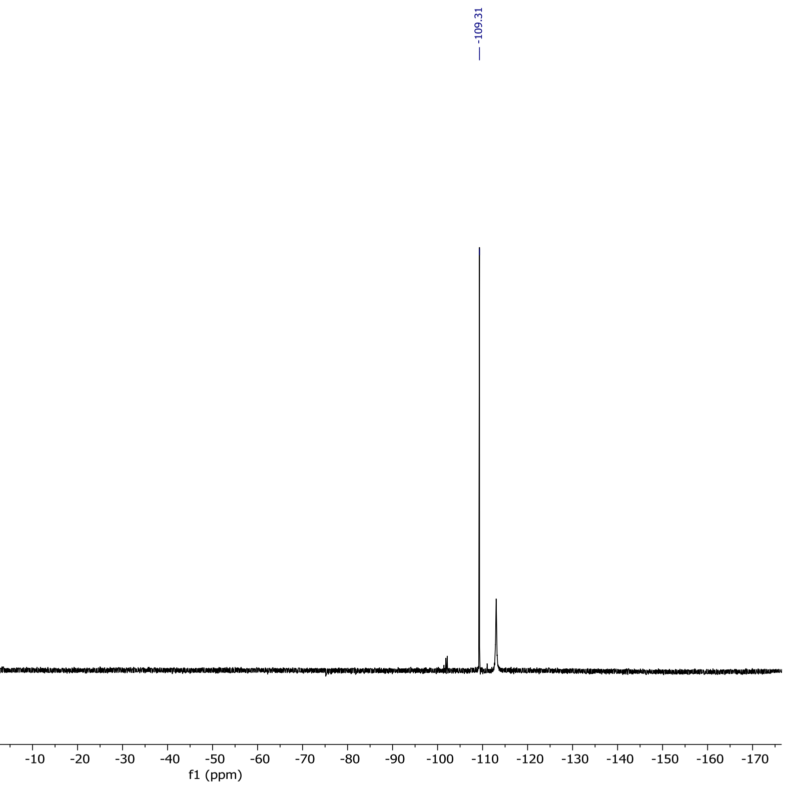

Figure SI-86. ^{19}F NMR (282 MHz, $CDCl_3$) spectrum of compound **11b**

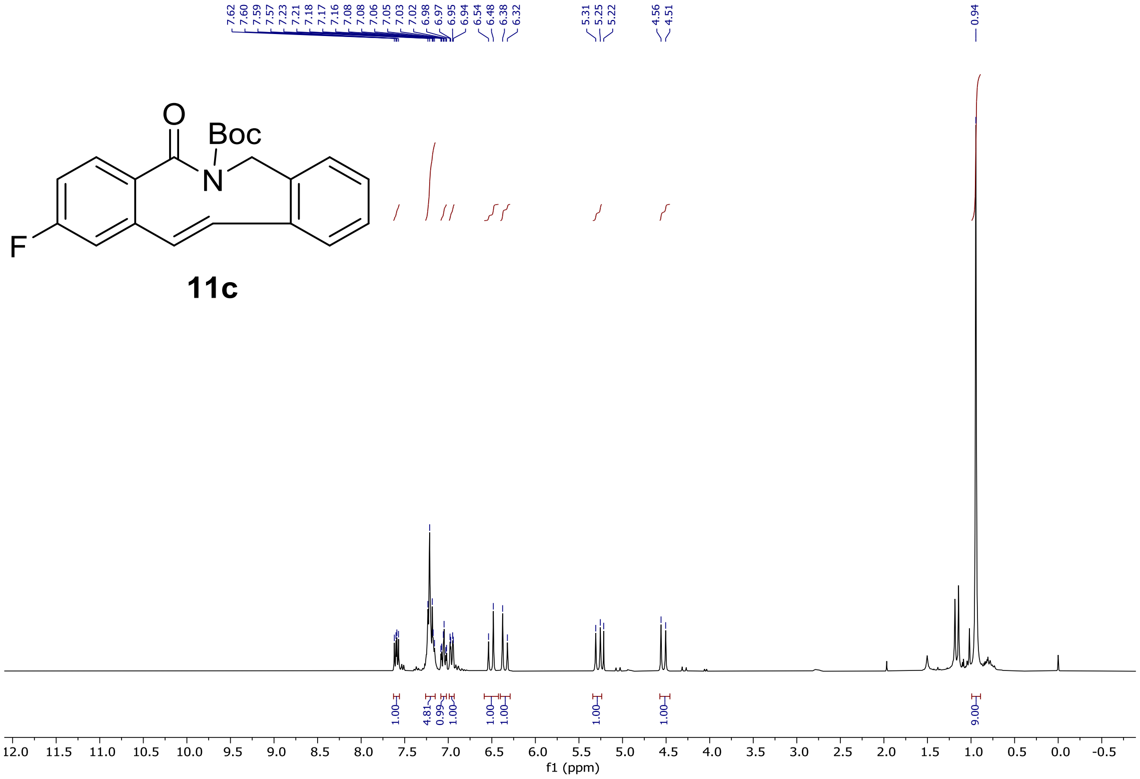

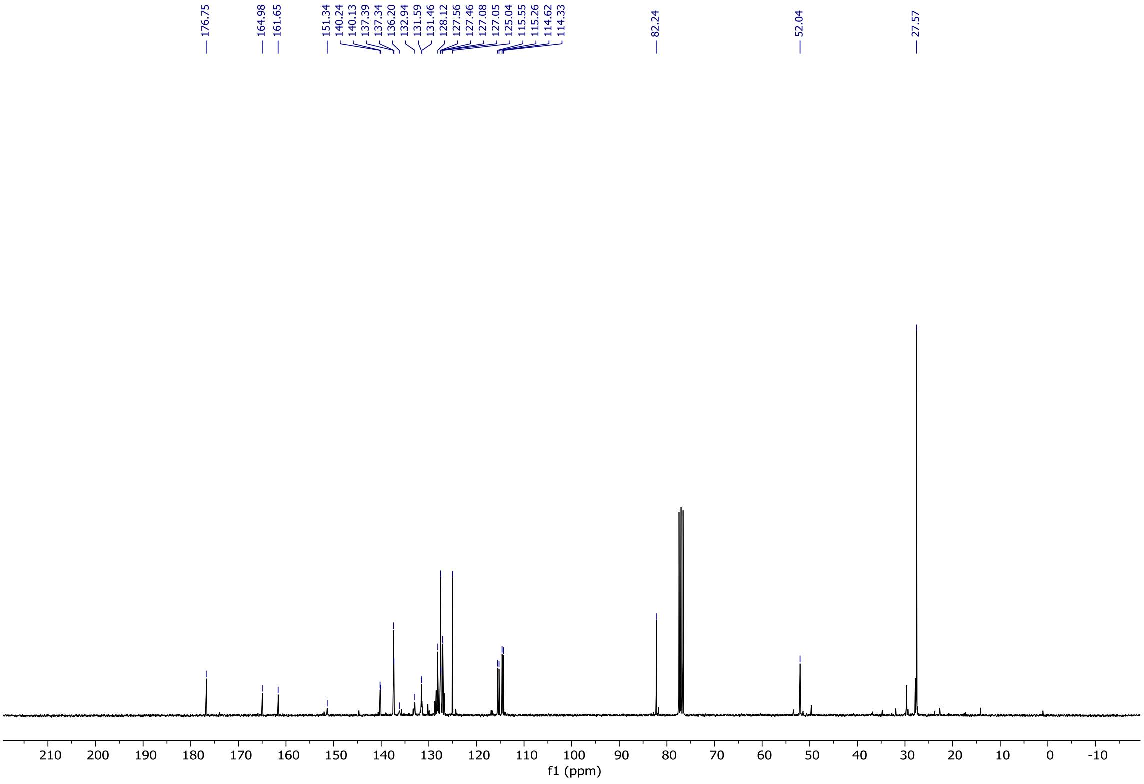

Figure SI-87. ^{1}H- NMR (300 MHz, $CDCl_3$) and ^{13}C {^{1}H} NMR (75 MHz, $CDCl_3$) spectra of compound **11c**

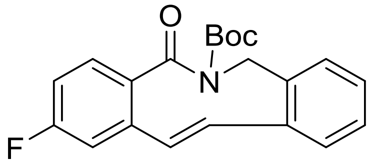

11c

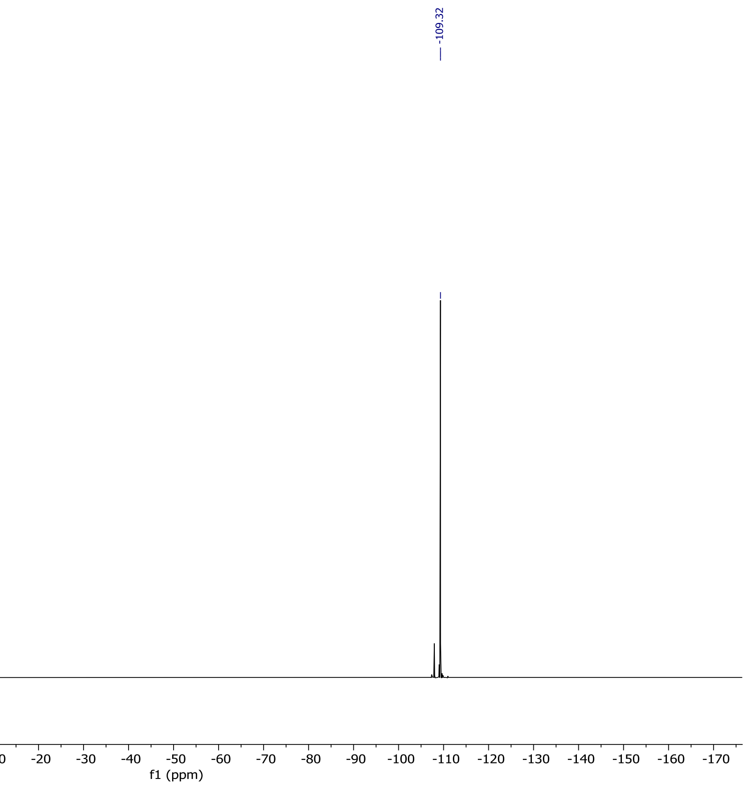

Figure SI-88. ^{19}F NMR (282 MHz, $CDCl_3$) spectrum of compound **11c**

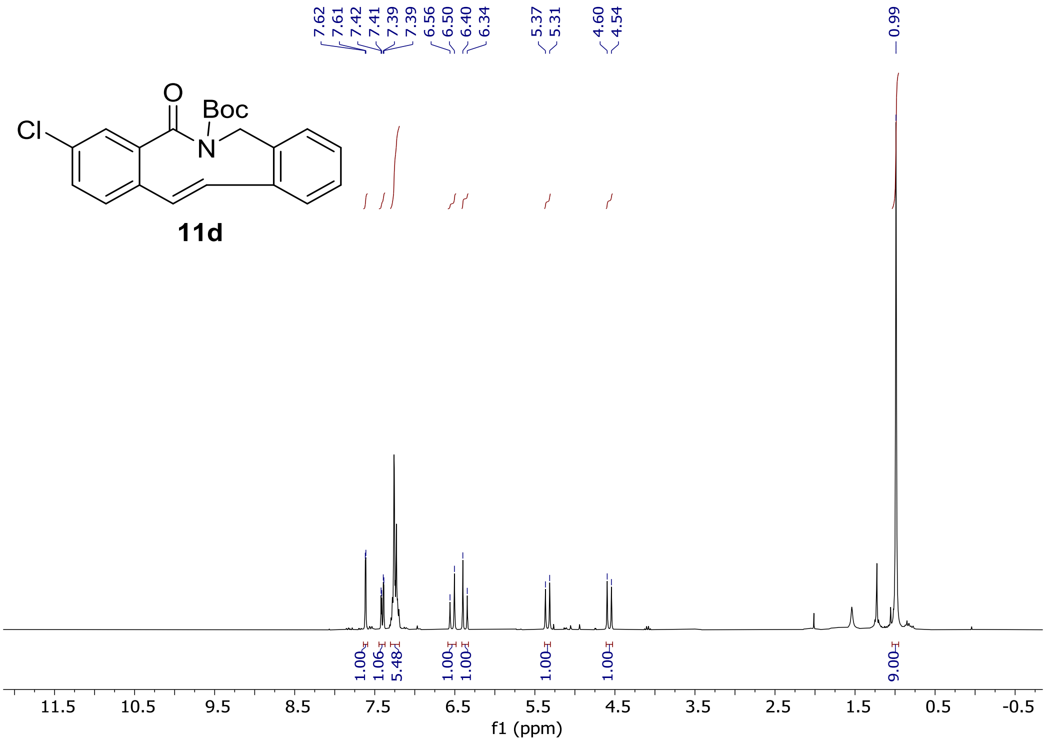

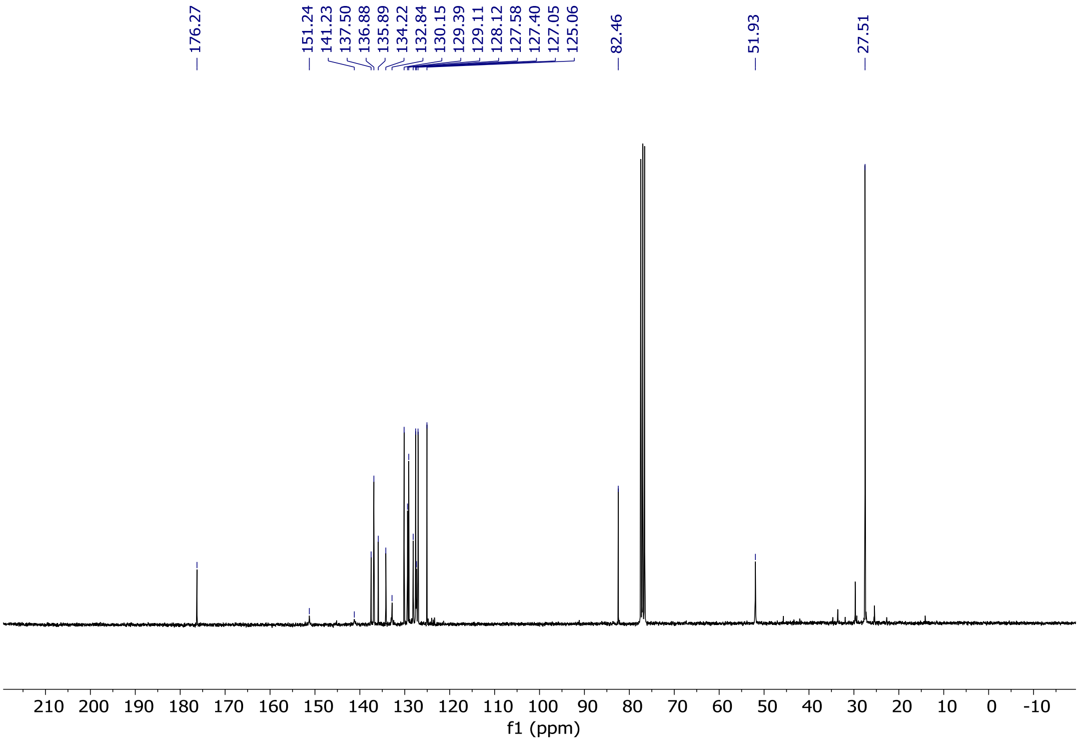

Figure SI-89. ^{1}H- NMR (300 MHz, $CDCl_3$) and ^{13}C {^{1}H} NMR (75 MHz, $CDCl_3$) spectra of compound **11d**

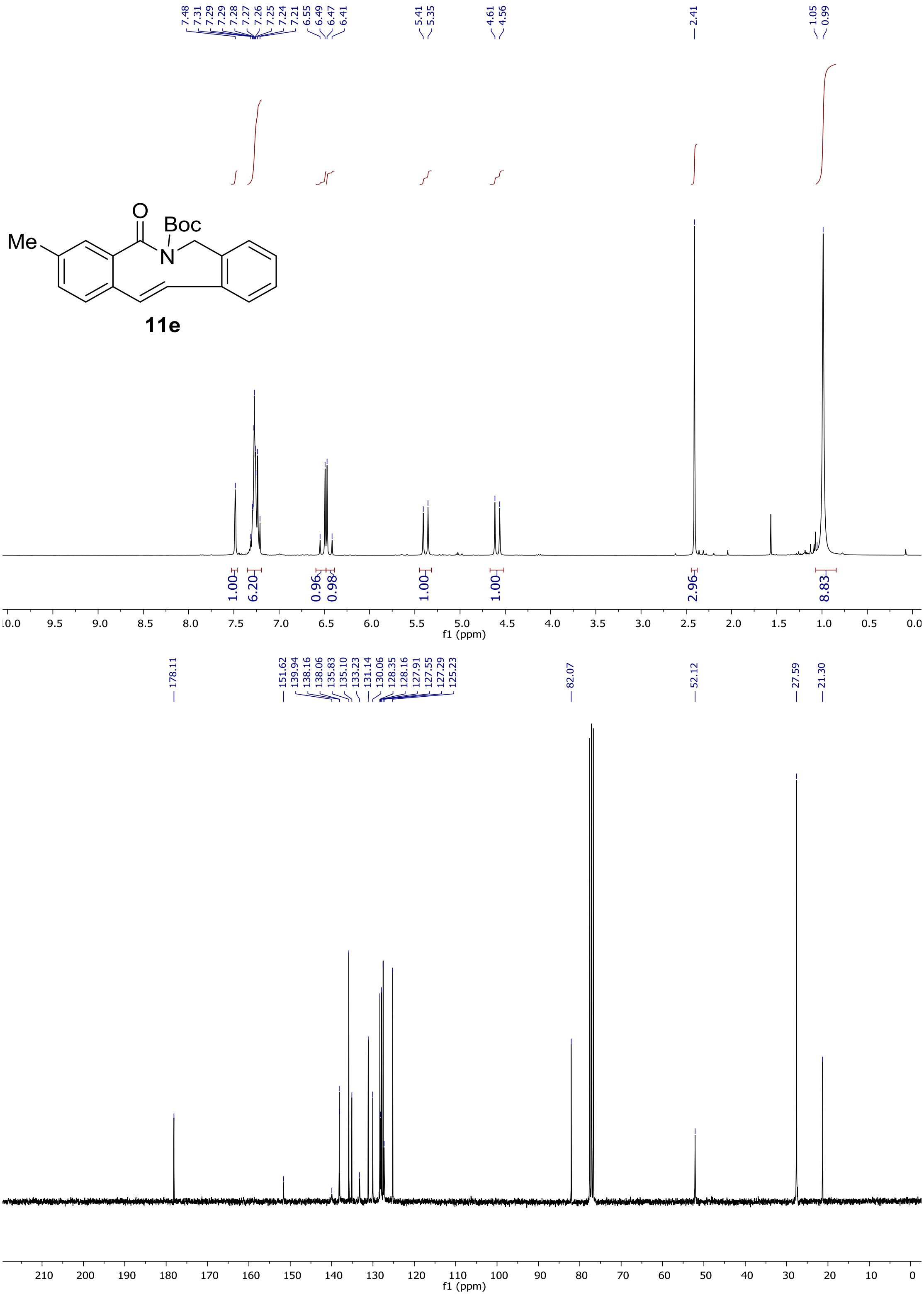

Figure SI-90. ^{1}H- NMR (300 MHz, $CDCl_3$) and ^{13}C {^{1}H} NMR (75 MHz, $CDCl_3$) spectra of compound **11e**

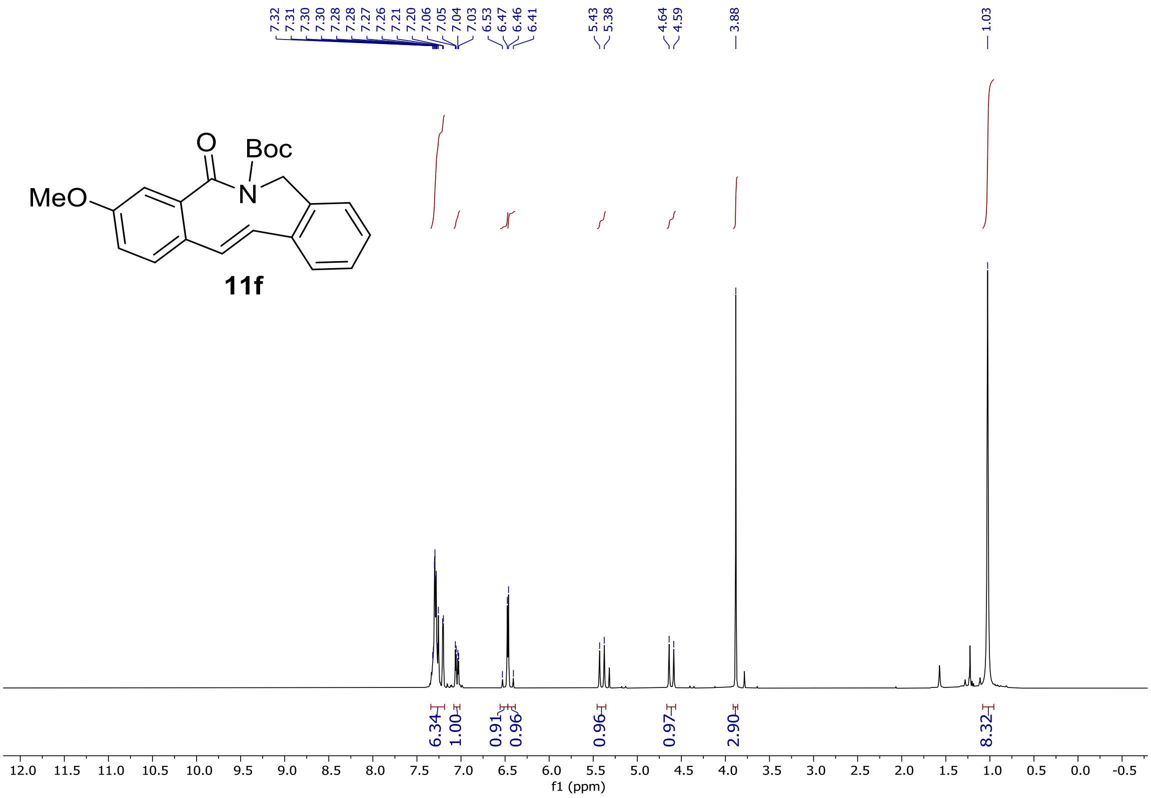

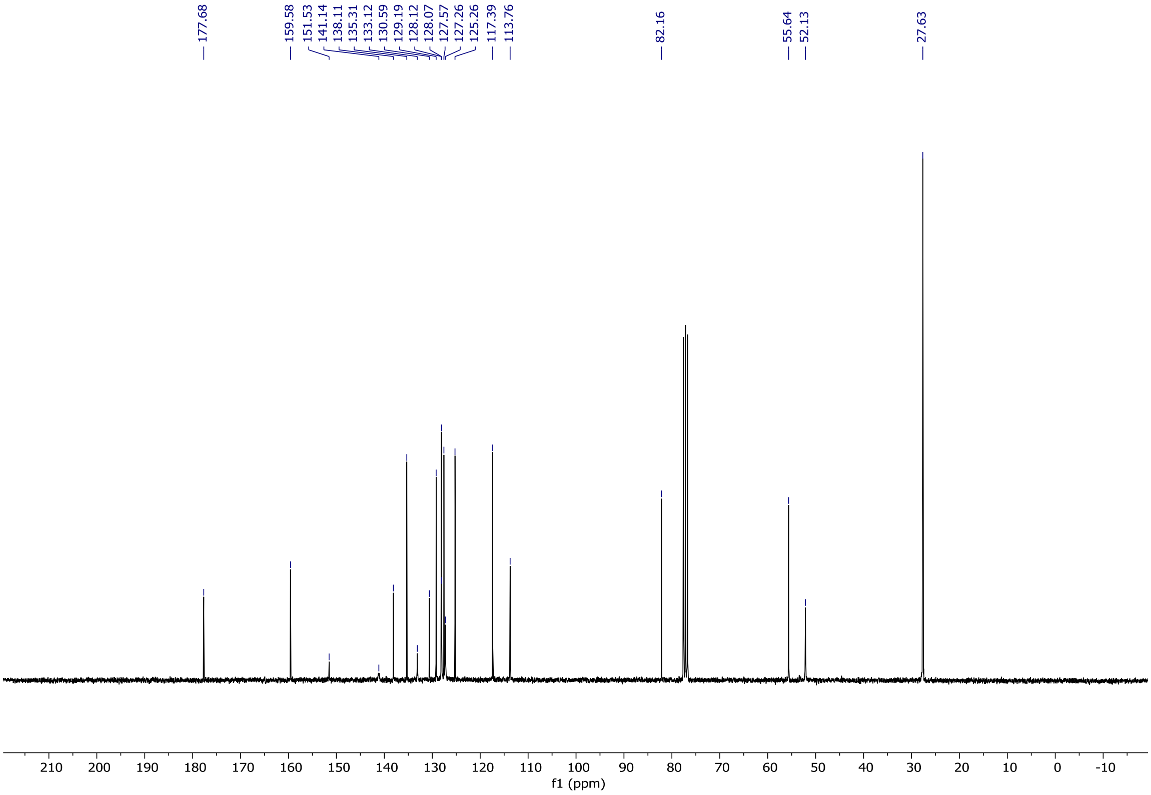

Figure SI-91. ^{1}H- NMR (300 MHz, $CDCl_3$) and $^{13}C\{^{1}H\}$ NMR (75 MHz, $CDCl_3$) spectra of compound **11f**

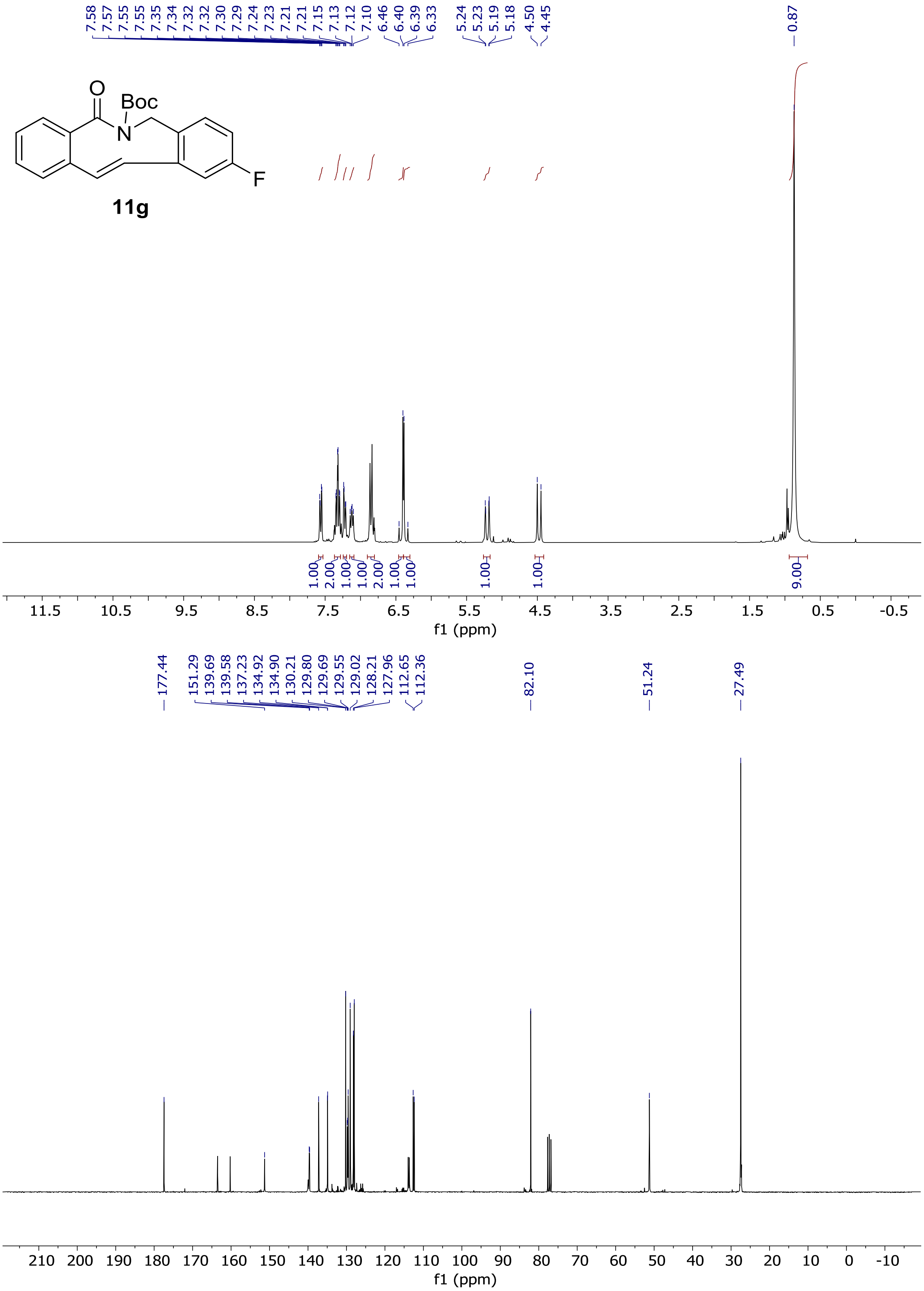

Figure SI-92. ^{1}H- NMR (300 MHz, $CDCl_3$) and ^{13}C {^{1}H} NMR (75 MHz, $CDCl_3$) spectra of compound **11g**

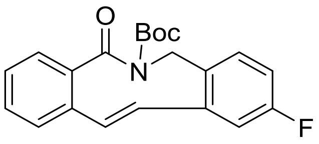

11g

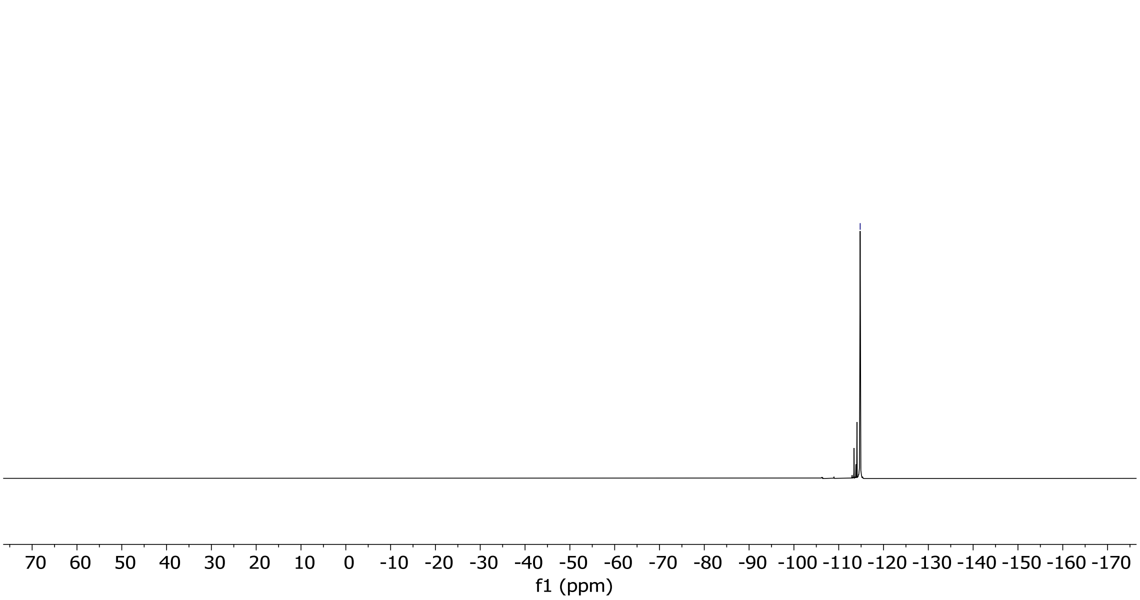

Figure SI-93. ^{19}F NMR (282 MHz, $CDCl_3$) spectrum of compound **11g**

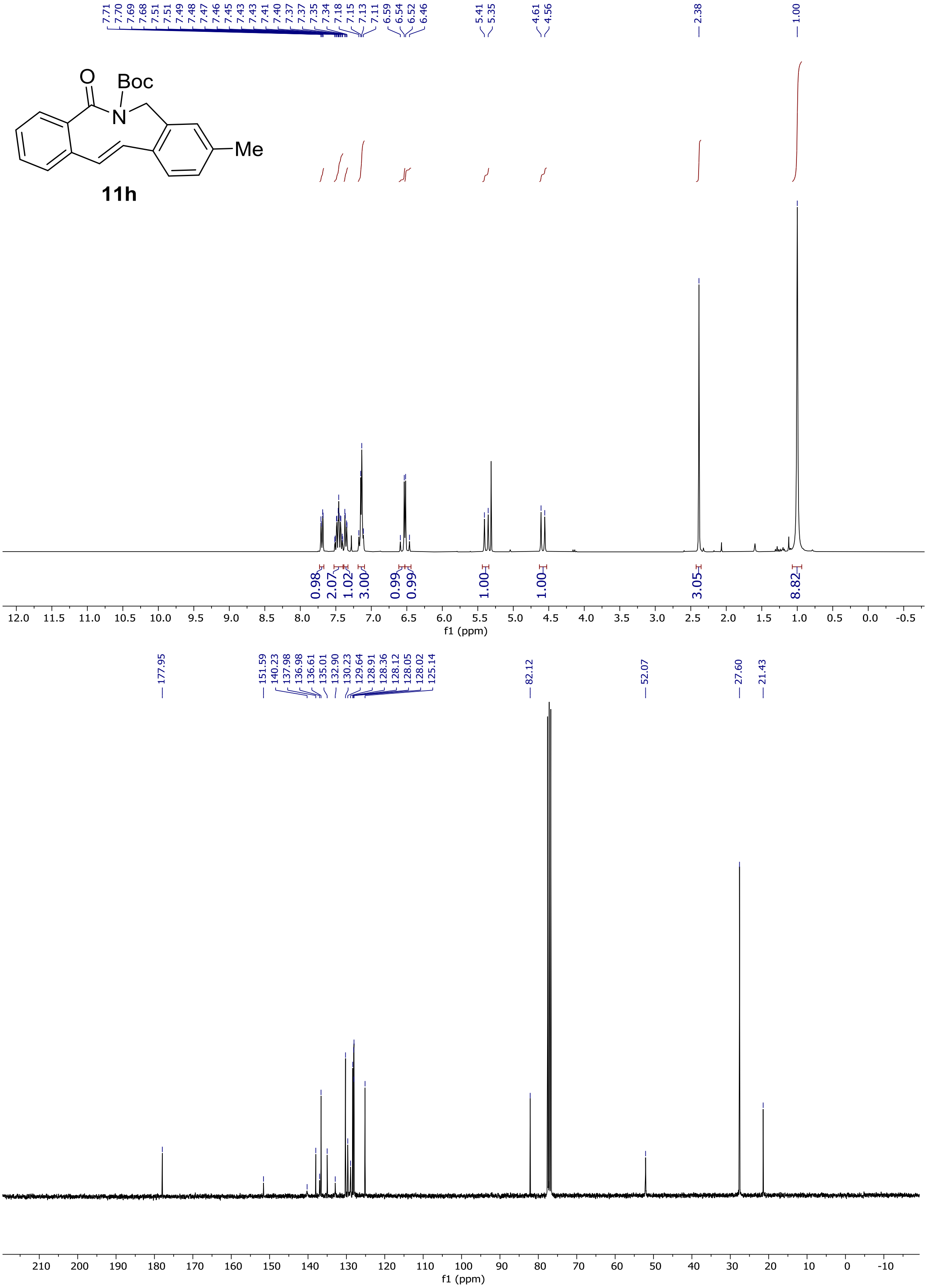

Figure SI-94. ^{1}H- NMR (300 MHz, $CDCl_3$) and ^{13}C {^{1}H} NMR (75 MHz, $CDCl_3$) spectra of compound **11h**

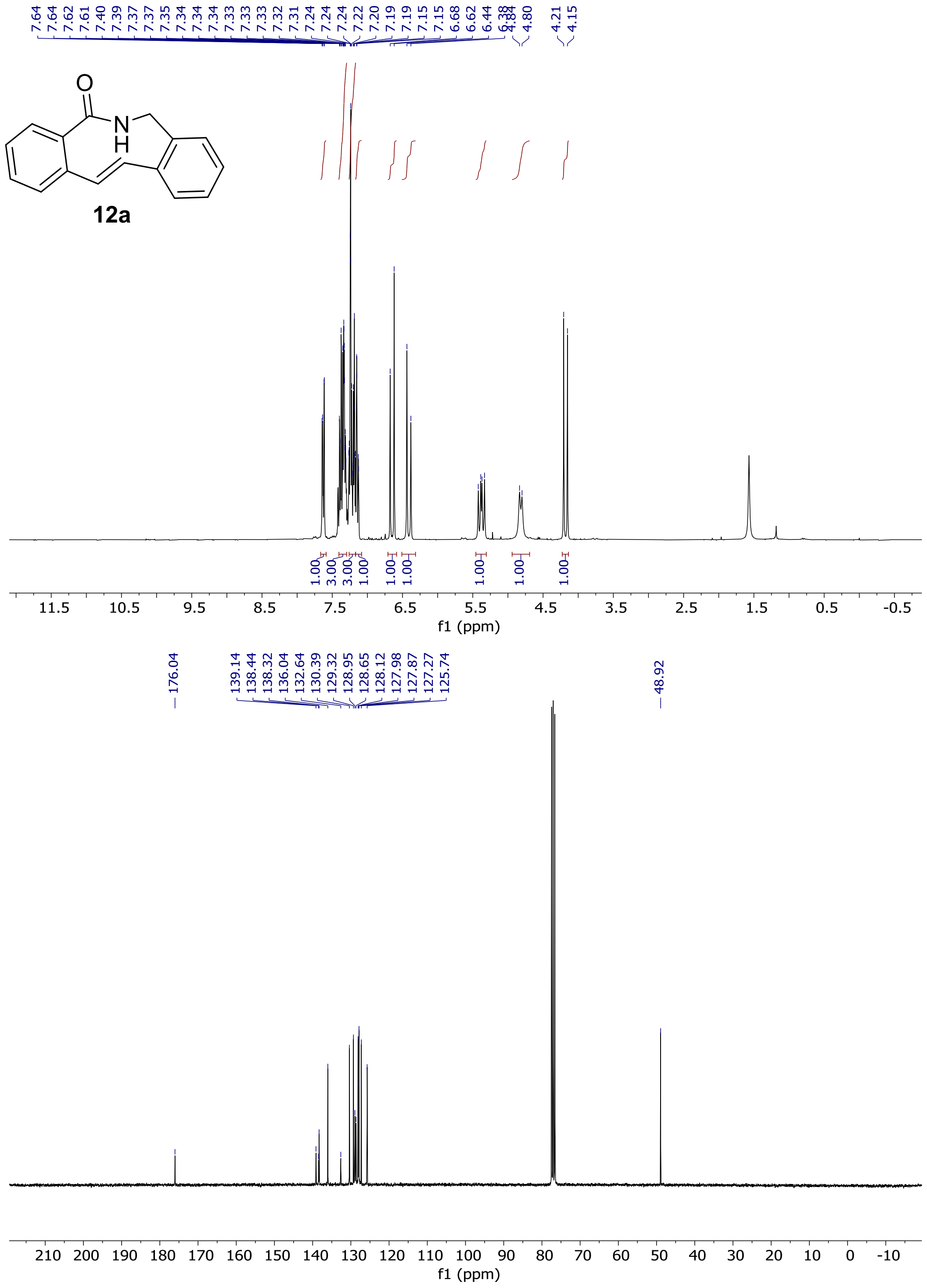

Figure SI-95. ^{1}H- NMR (300 MHz, $CDCl_3$) and ^{13}C {^{1}H} NMR (75 MHz, $CDCl_3$) spectra of compound **12a**

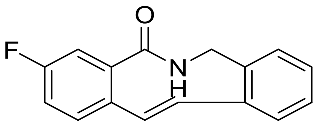

12b

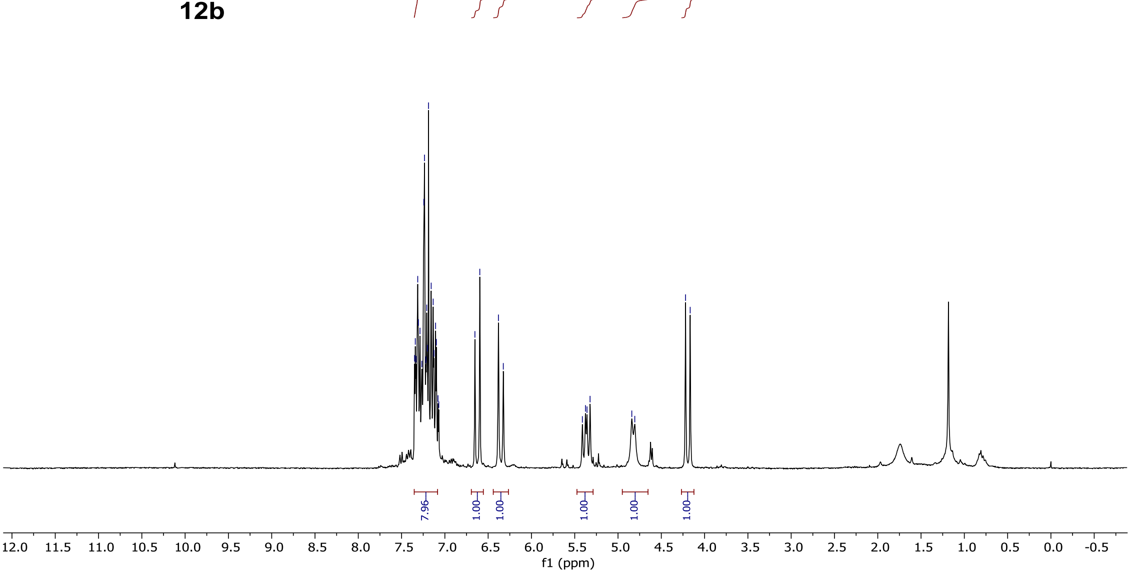

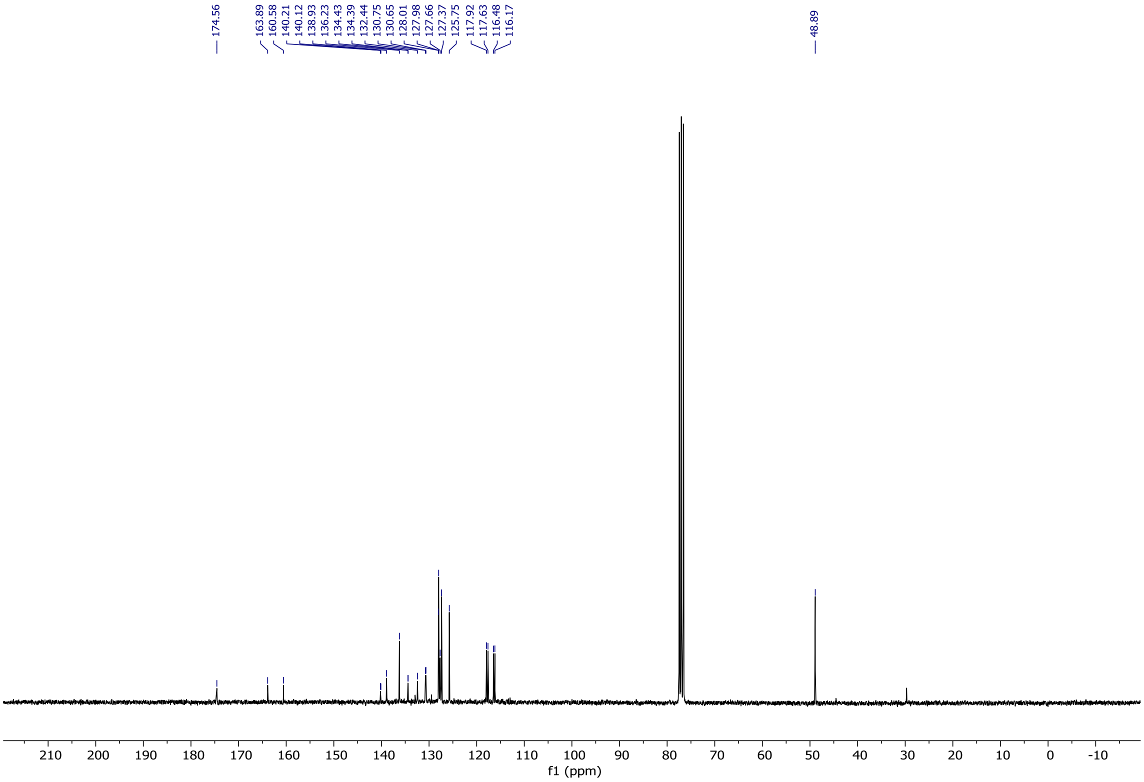

Figure SI-96. ^{1}H- NMR (300 MHz, $CDCl_3$) and ^{13}C {^{1}H} NMR (75 MHz, $CDCl_3$) spectra of compound **12b**

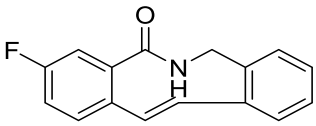

12b

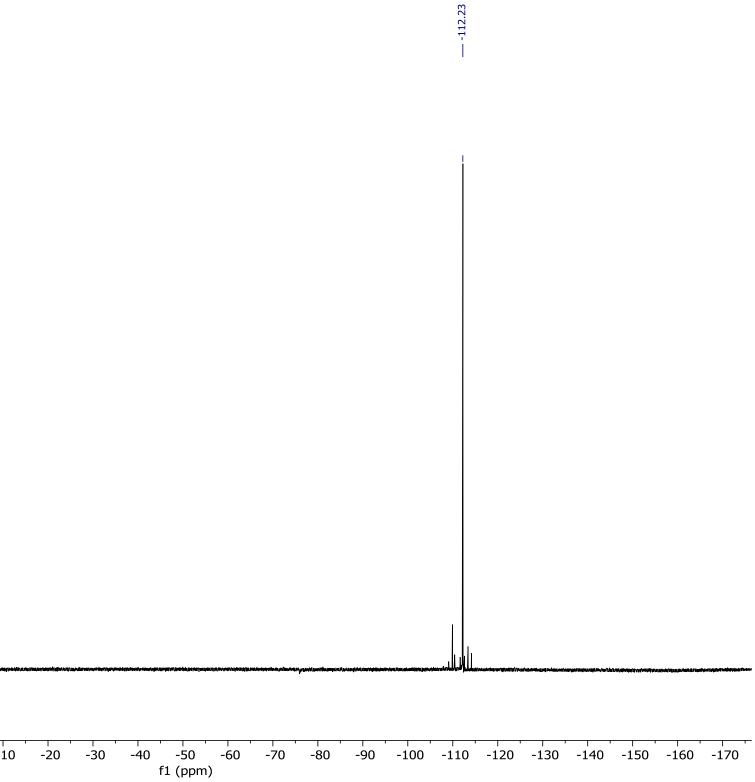

Figure SI-97. ^{19}F NMR (282 MHz, $CDCl_3$) spectrum of compound **12b**

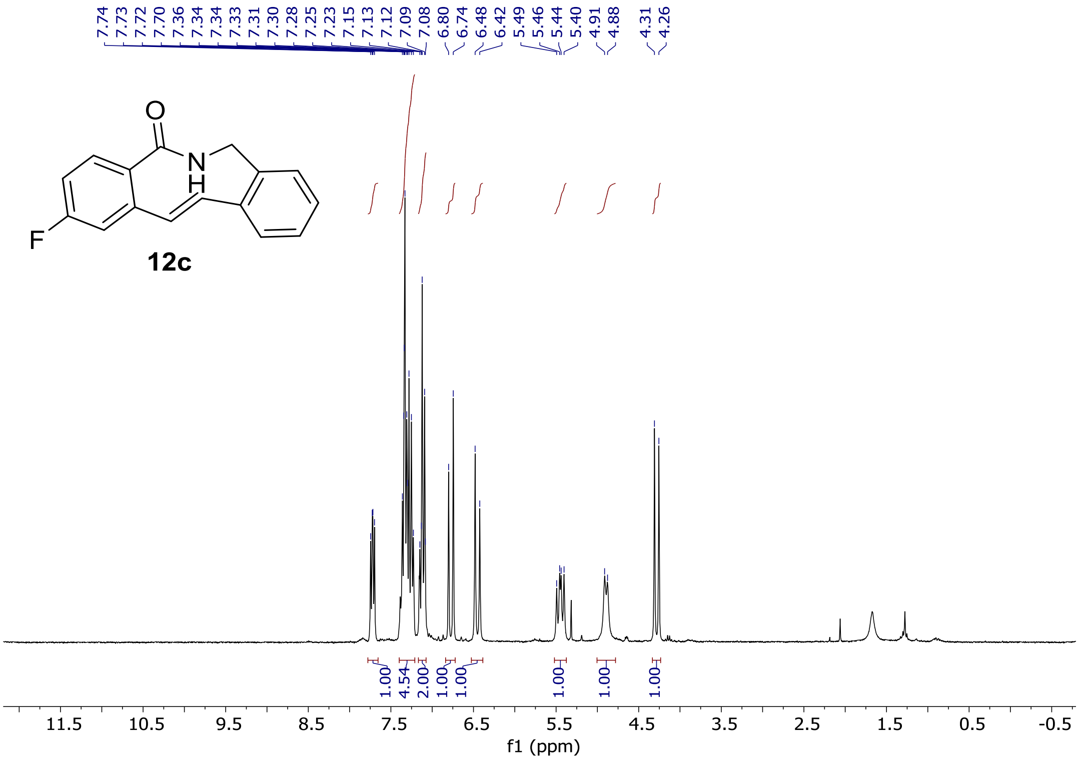

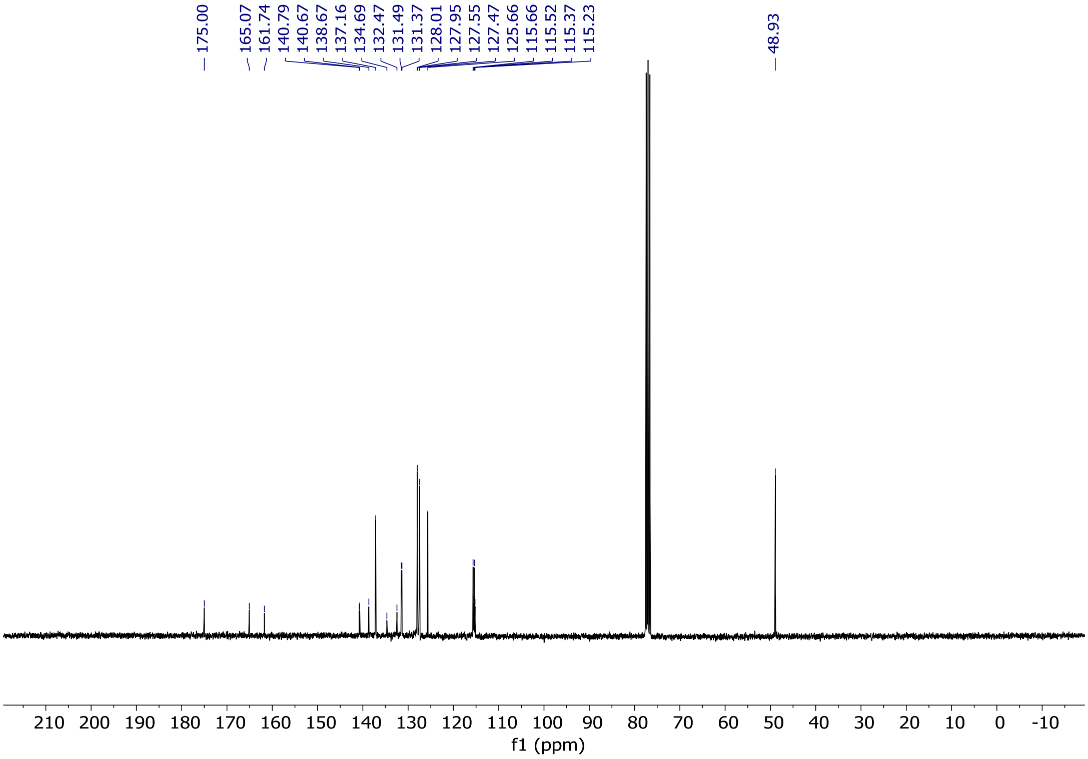

Figure SI-98. ^{1}H- NMR (300 MHz, $CDCl_3$) and ^{13}C {^{1}H} NMR (75 MHz, $CDCl_3$) spectra of compound **12c**

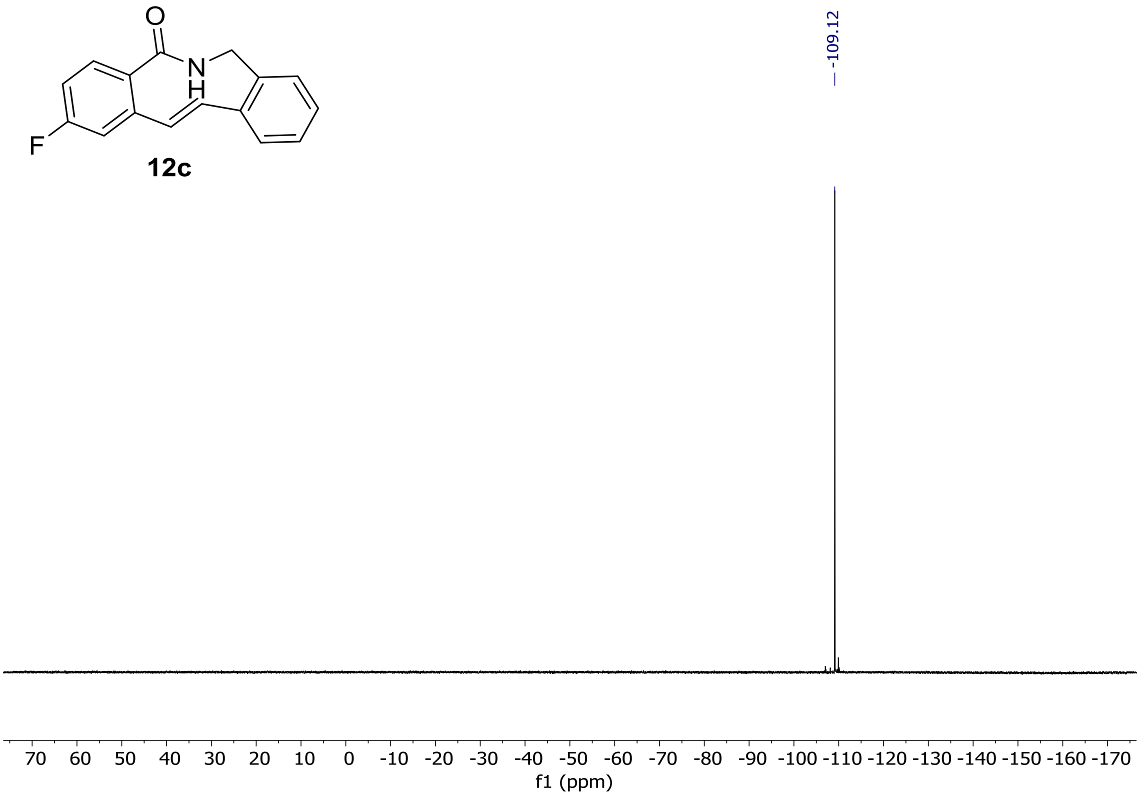

Figure SI-99. ^{19}F NMR (282 MHz, $CDCl_3$) spectrum of compound **12c**

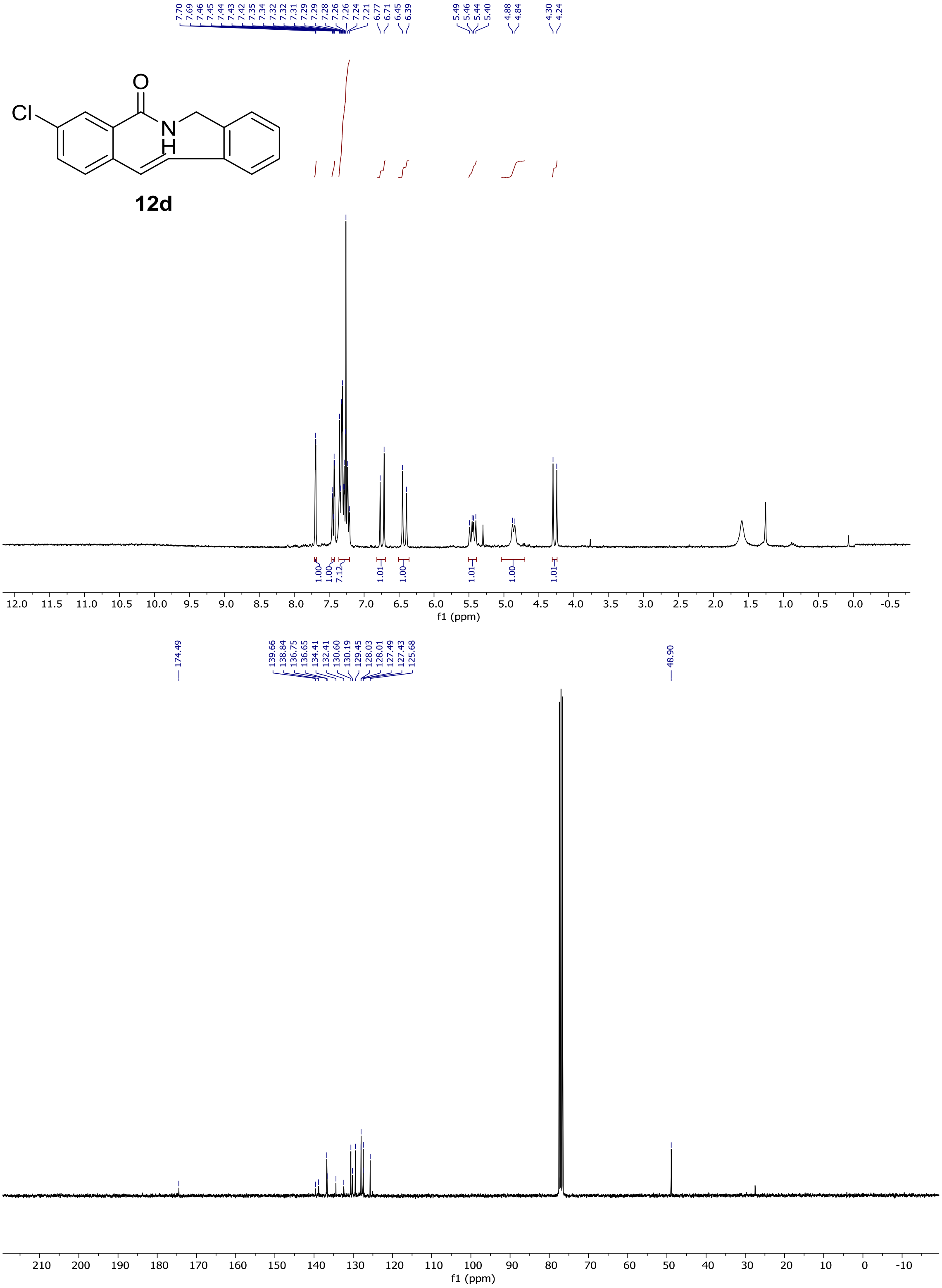

Figure SI-100. ^{1}H- NMR (300 MHz, $CDCl_3$) and ^{13}C {^{1}H} NMR (75 MHz, $CDCl_3$) spectra of compound **12d**

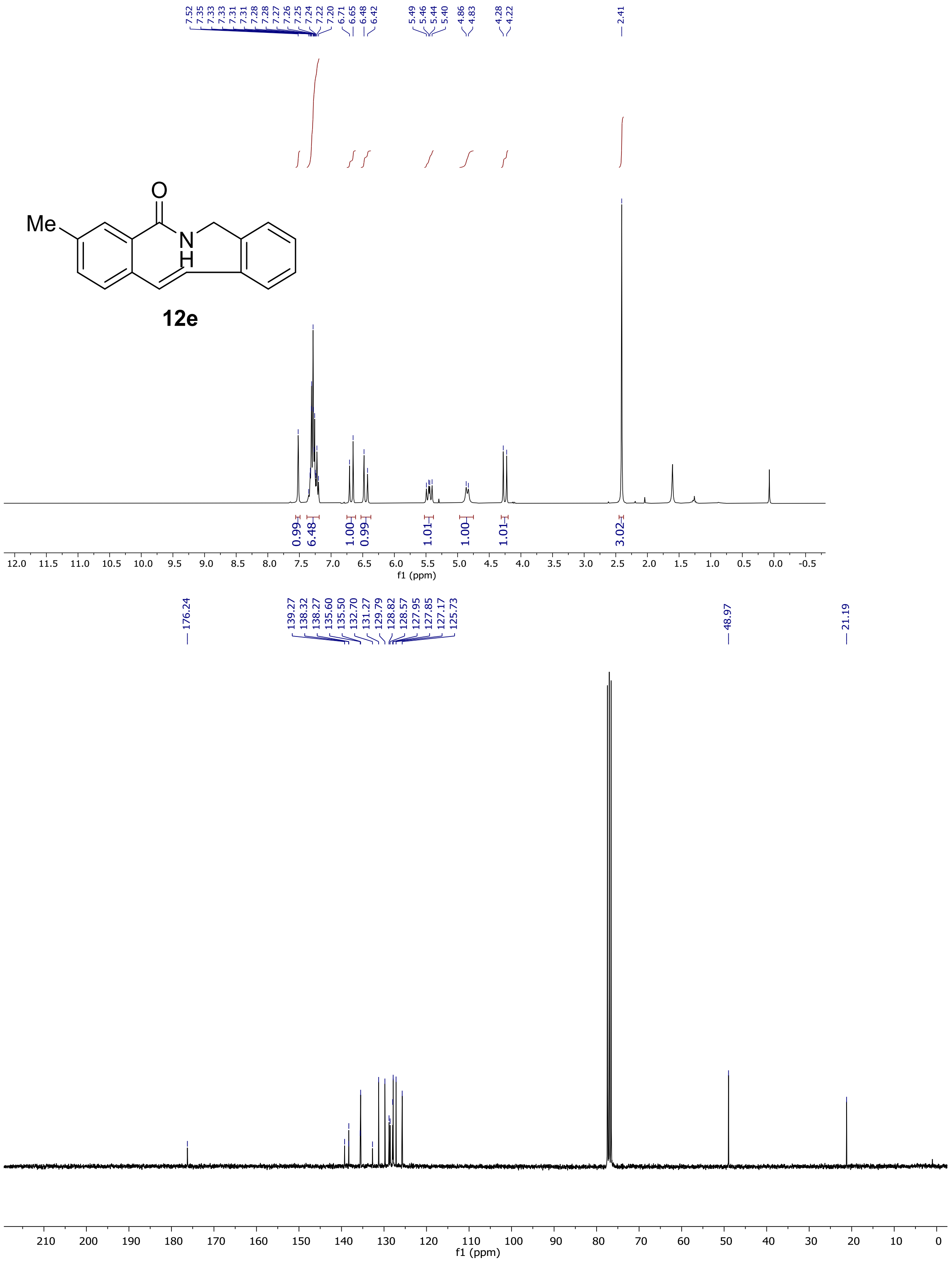

Figure SI-101. ^{1}H- NMR (300 MHz, $CDCl_3$) and ^{13}C {^{1}H} NMR (75 MHz, $CDCl_3$) spectra of compound **12e**

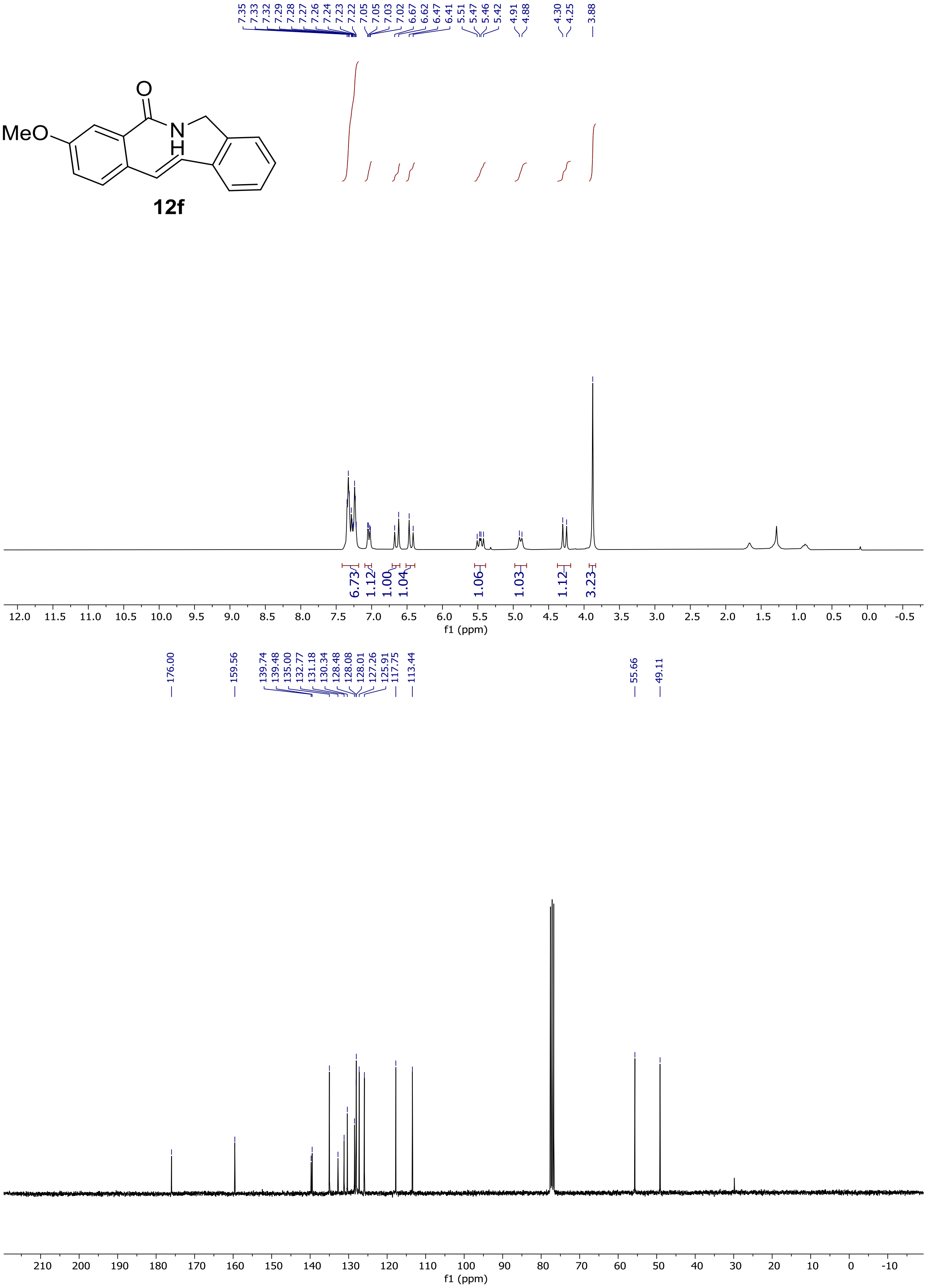

Figure SI-102. ^{1}H- NMR (300 MHz, $CDCl_3$) and ^{13}C {^{1}H} NMR (75 MHz, $CDCl_3$) spectra of compound **12f**

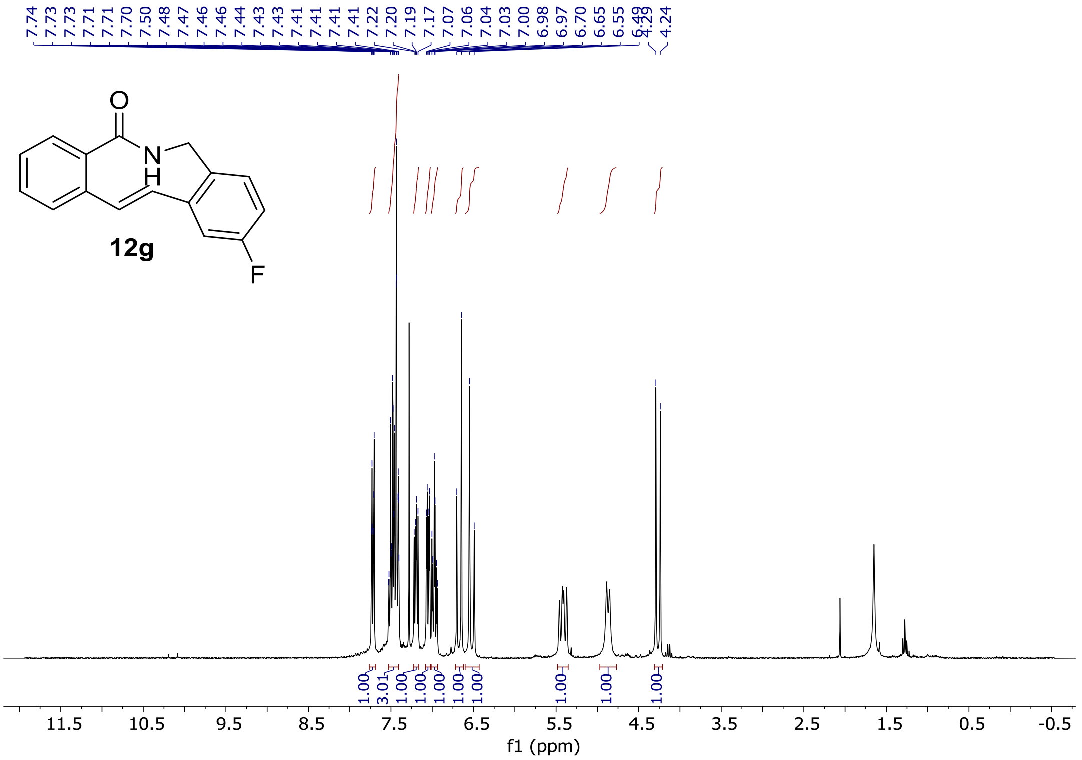

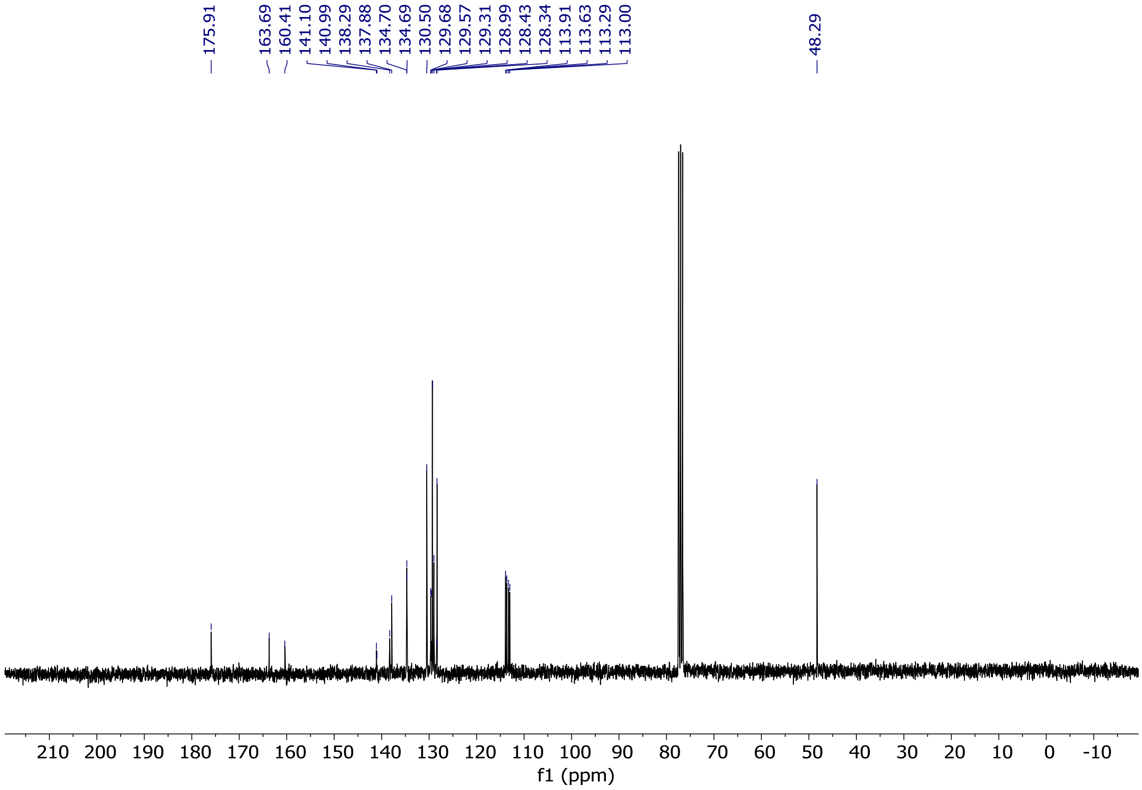

Figure SI-103. ^{1}H- NMR (300 MHz, $CDCl_3$) and ^{13}C {^{1}H} NMR (75 MHz, $CDCl_3$) spectra of compound **12g**

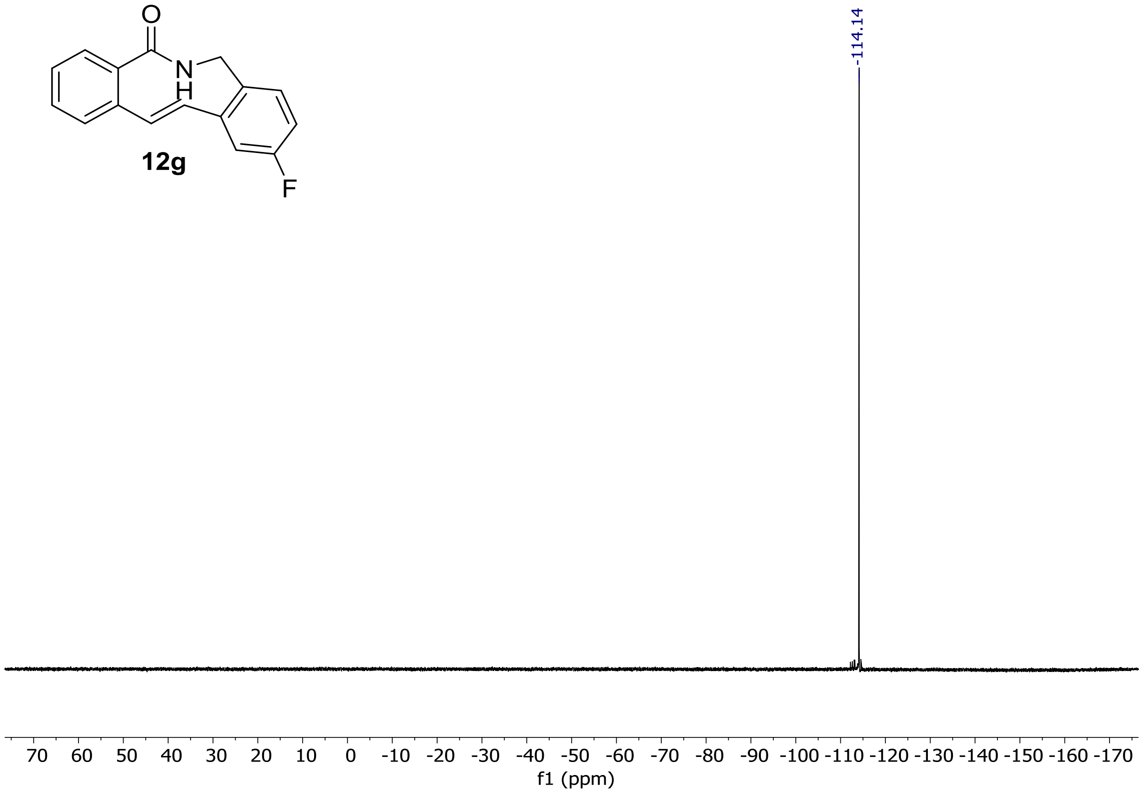

Figure SI-104. ^{19}F NMR (282 MHz, $CDCl_3$) spectrum of compound **12g**

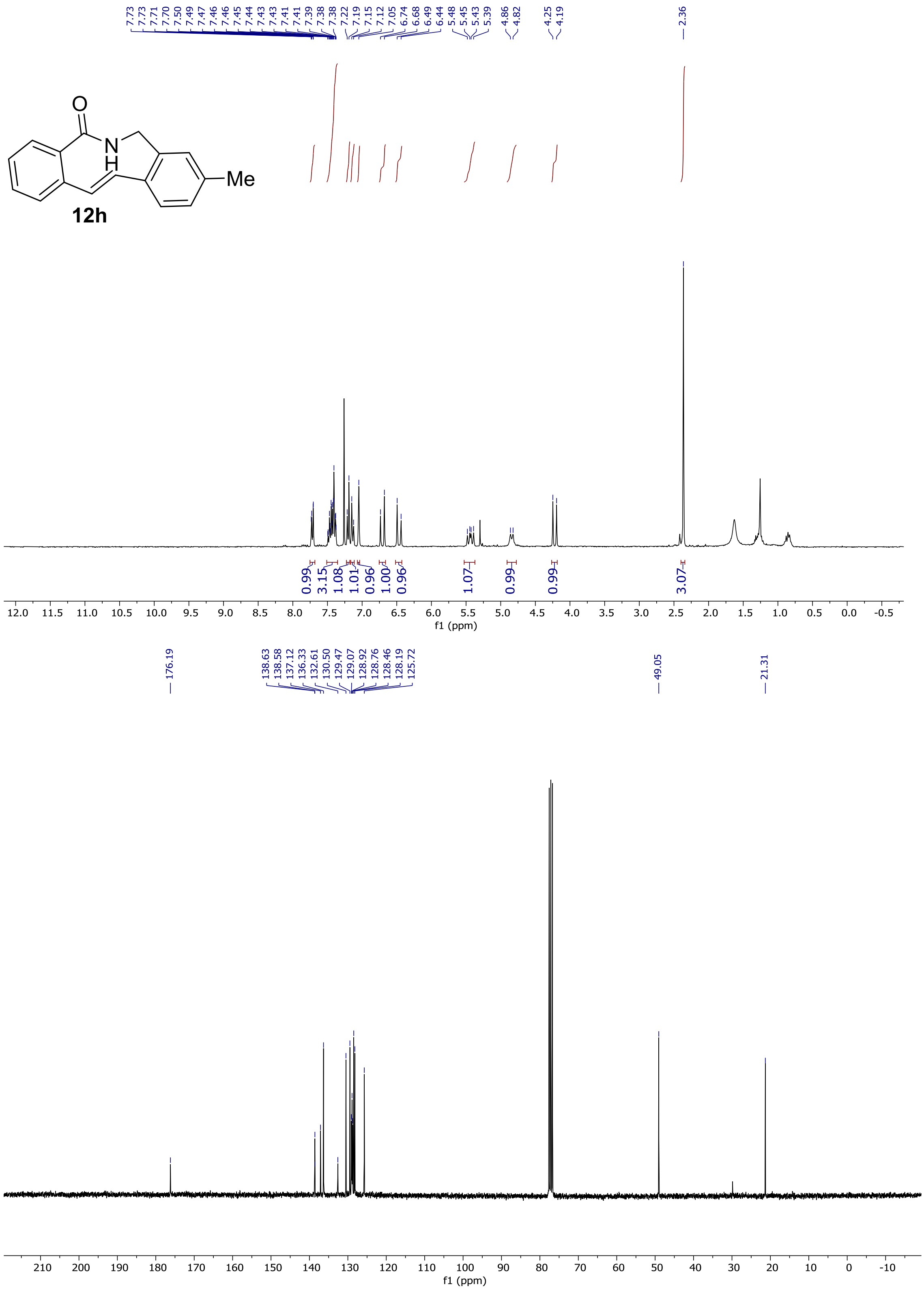

Figure SI-105. ^{1}H- NMR (300 MHz, $CDCl_3$) and ^{13}C {^{1}H} NMR (75 MHz, $CDCl_3$) spectra of compound **12h**

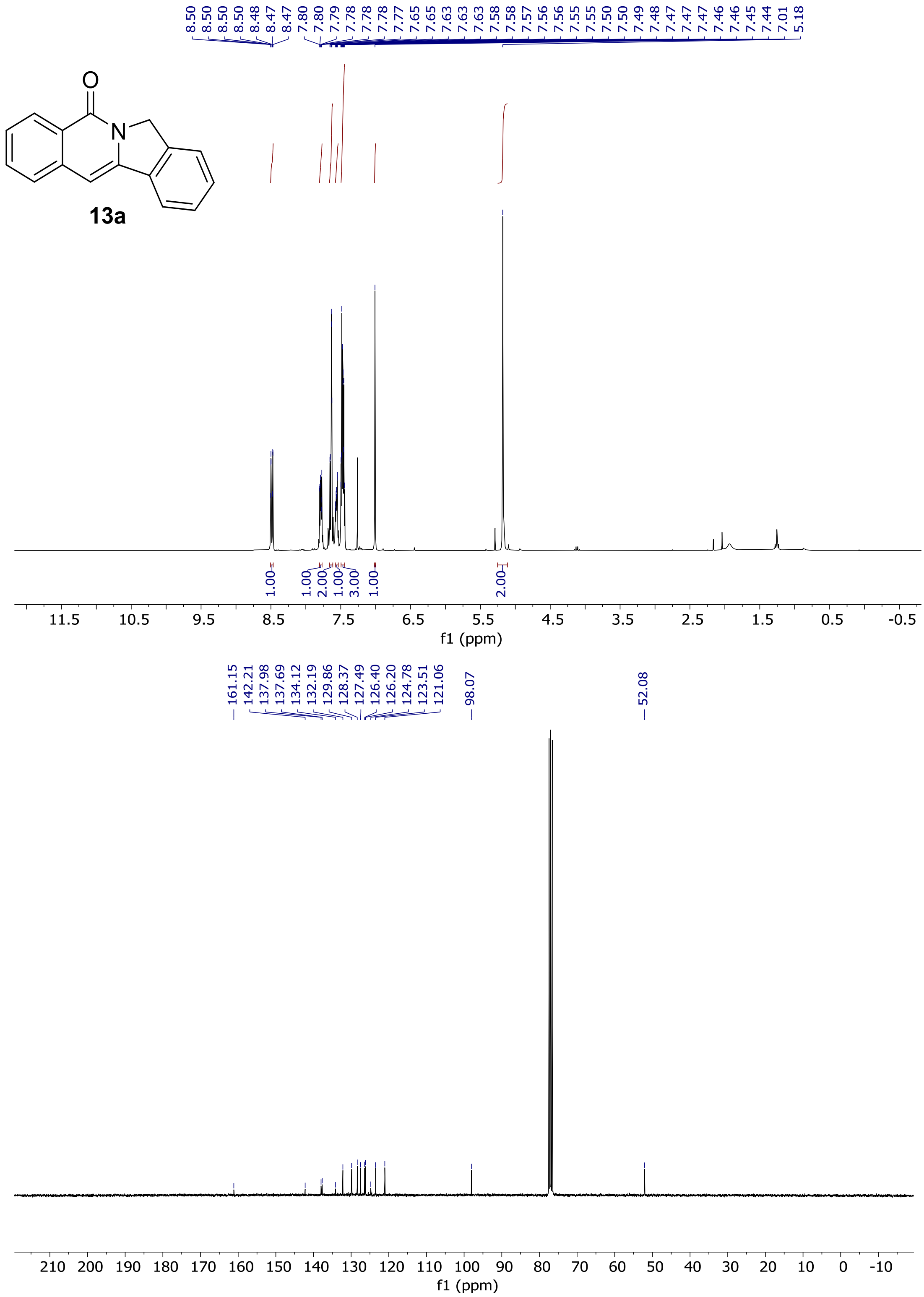

Figure SI-106. ^{1}H- NMR (300 MHz, $CDCl_3$) and ^{13}C {^{1}H} NMR (75 MHz, $CDCl_3$) spectra of compound **13a**

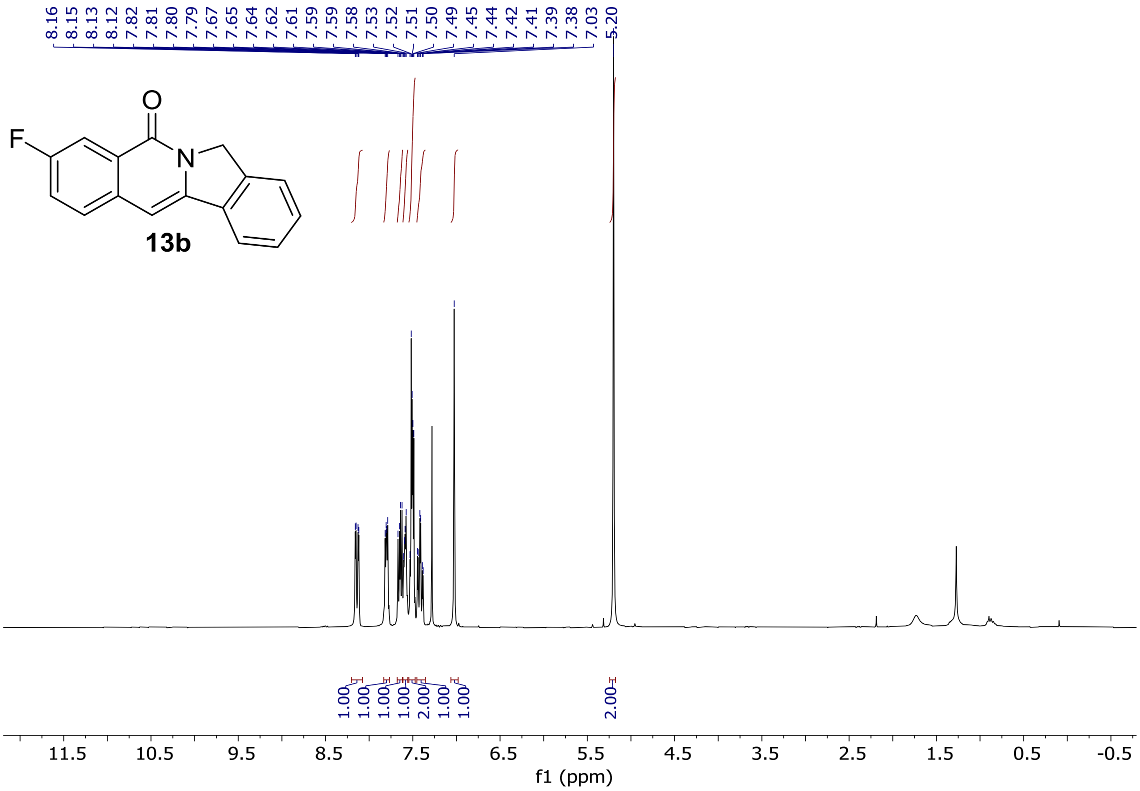

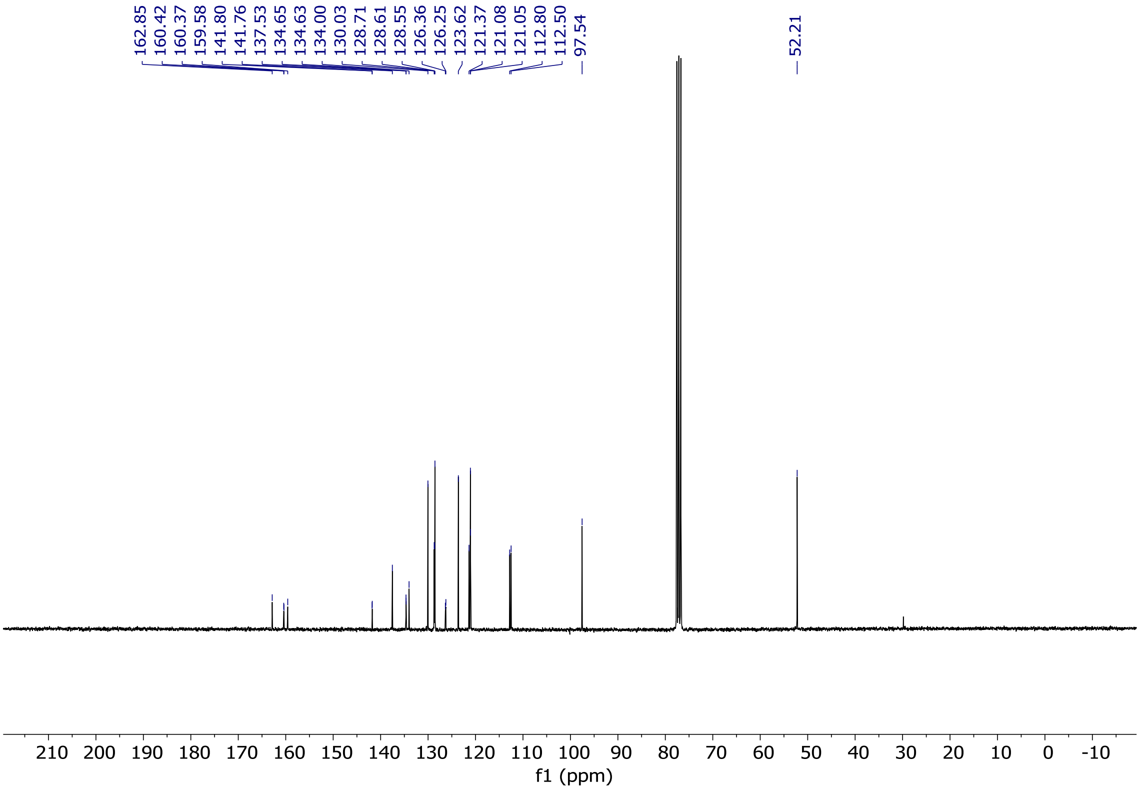

Figure SI-107. ^{1}H-NMR (300 MHz, $CDCl_3$) and ^{13}C {^{1}H} NMR (75 MHz, $CDCl_3$) spectra of compound **13b**

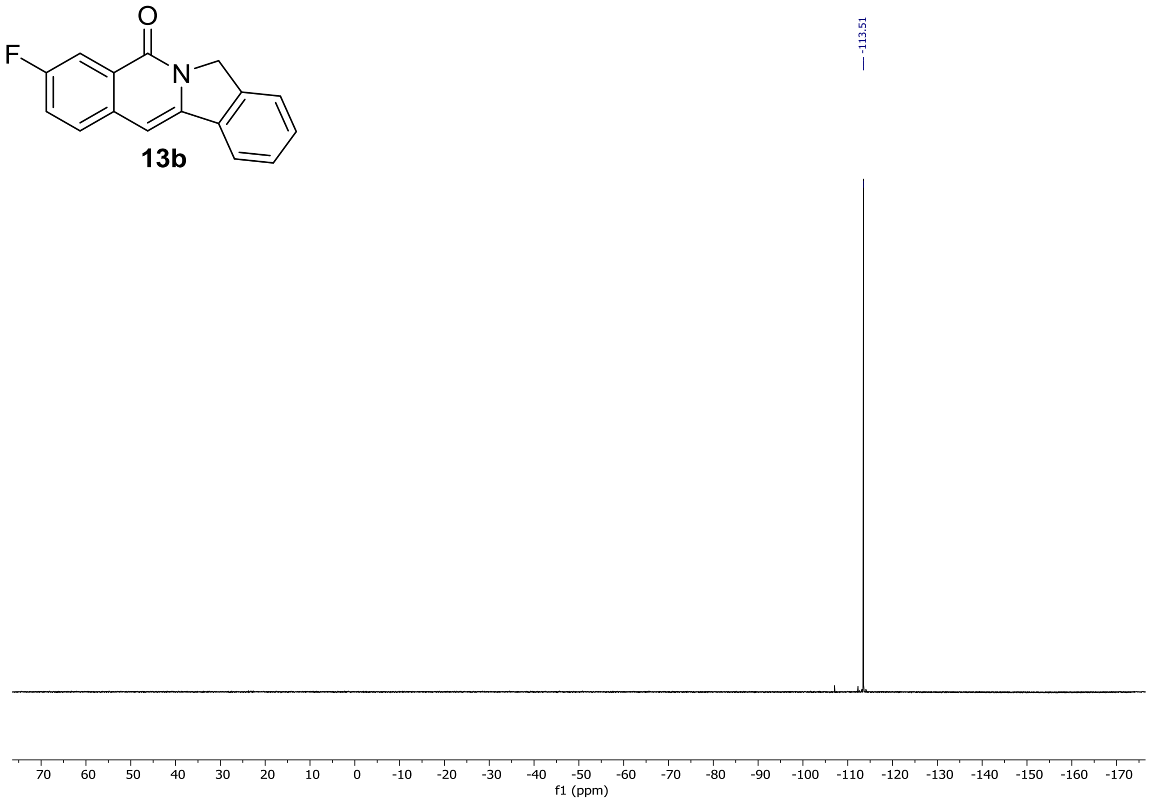

Figure SI-108. ^{19}F NMR (282 MHz, $CDCl_3$) spectrum of compound **13b**

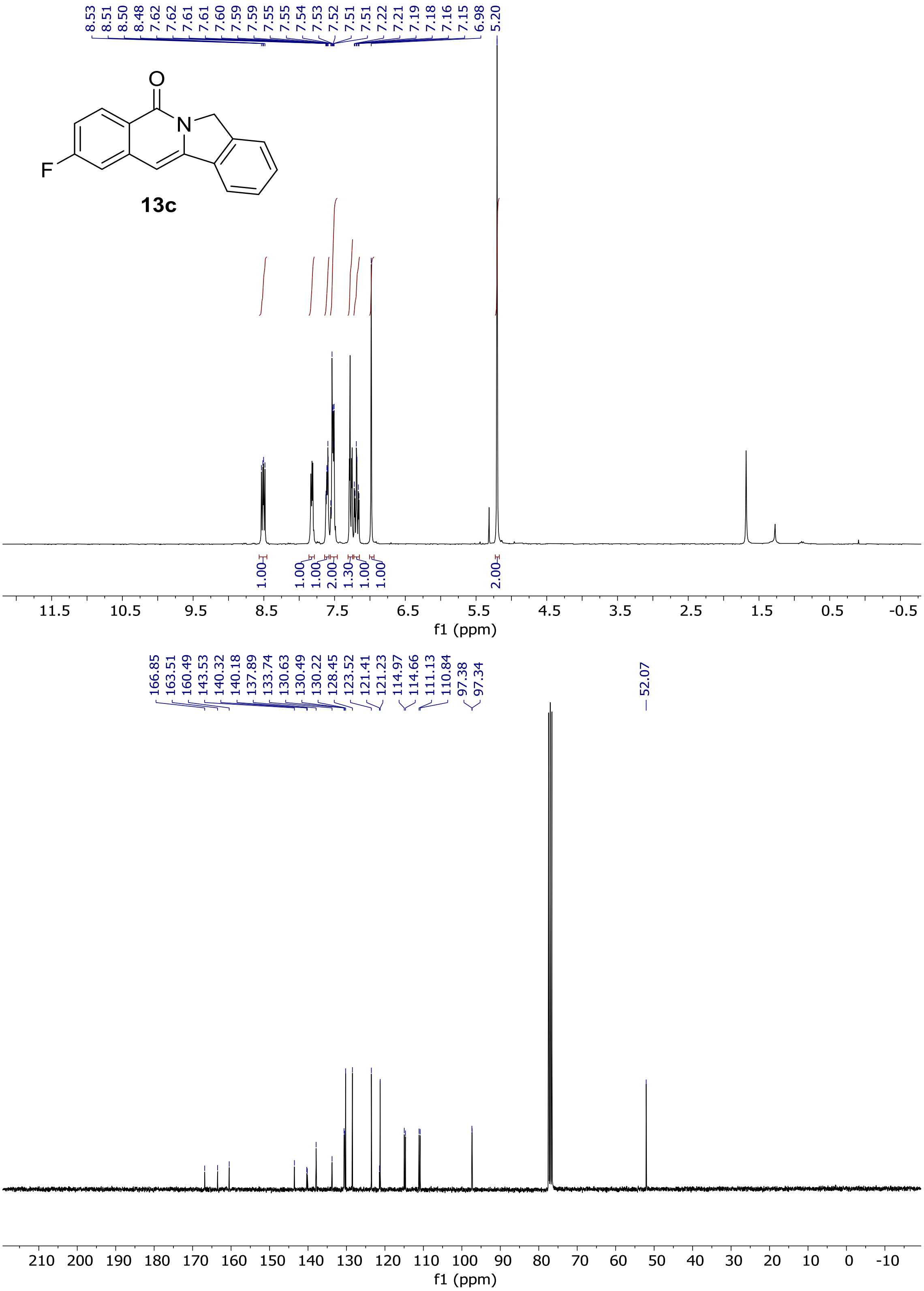

Figure SI-109. ^{1}H-NMR (300 MHz, $CDCl_3$) and ^{13}C {^{1}H} NMR (75 MHz, $CDCl_3$) spectra of compound **13c**

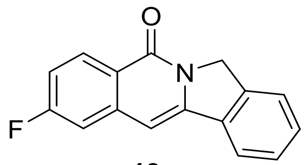

13c

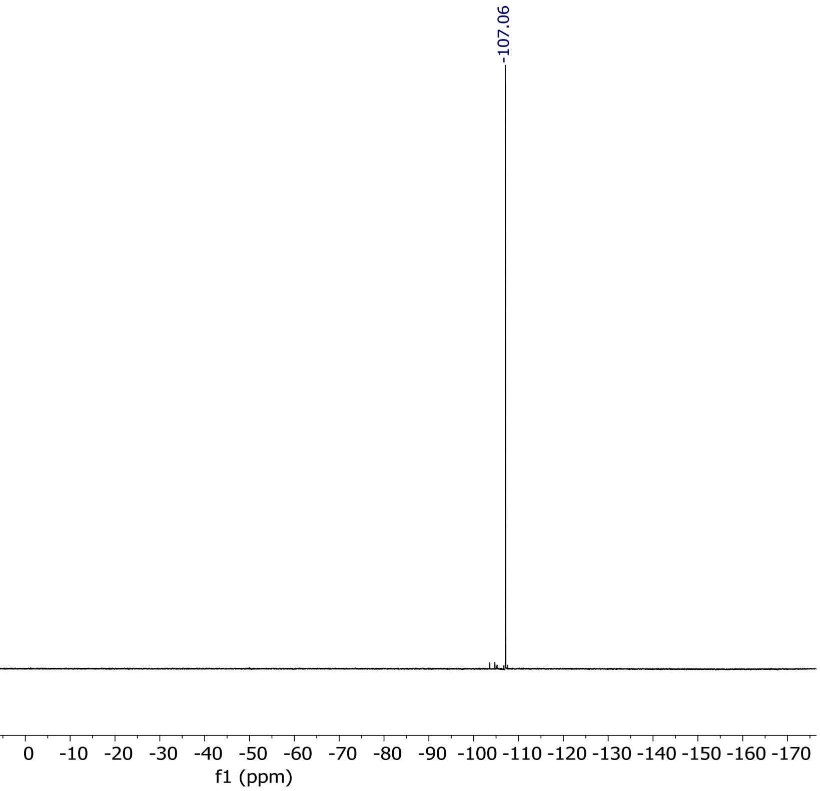

Figure SI-110. ^{19}F NMR (282 MHz, $CDCl_3$) spectrum of compound **13c**

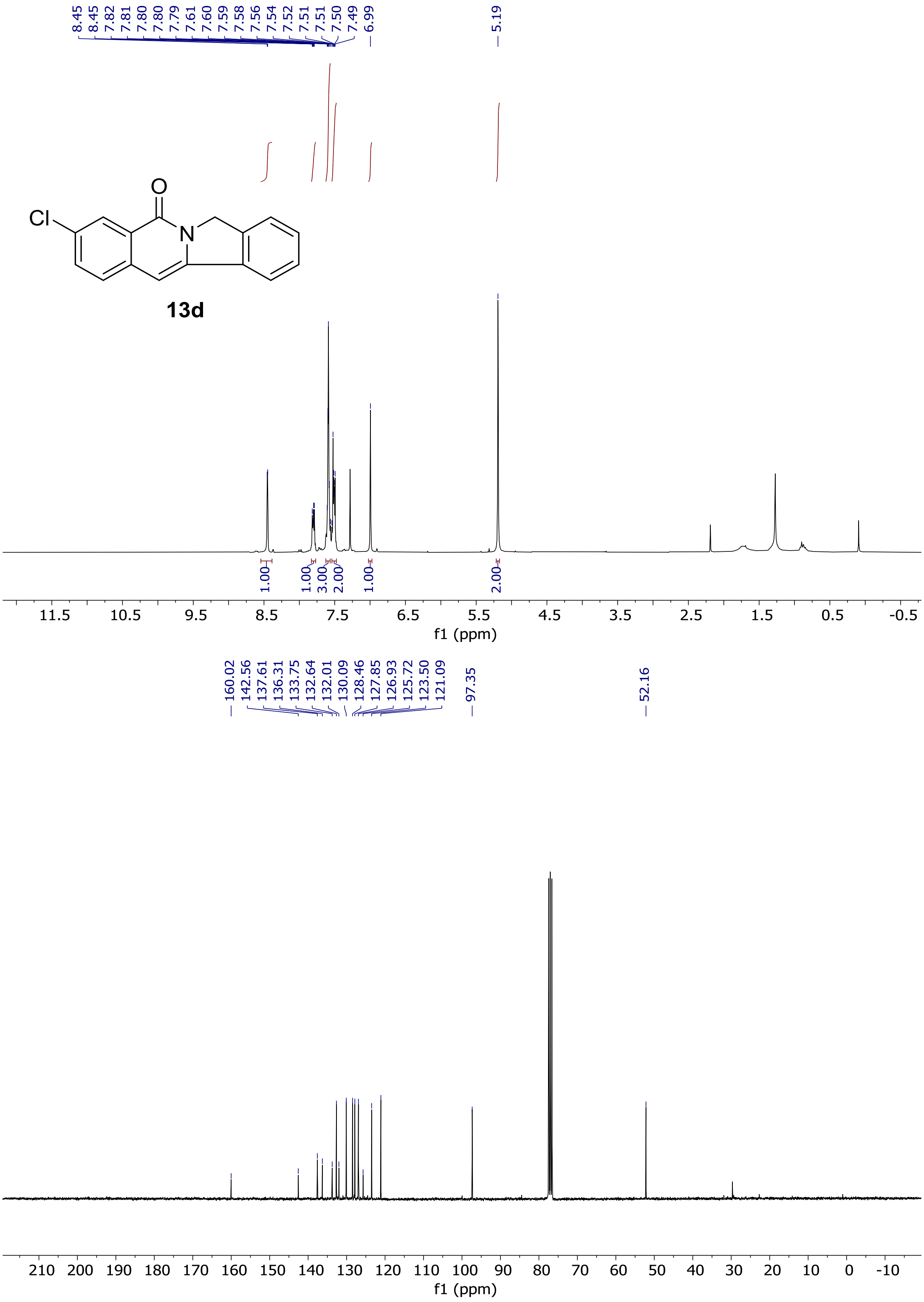

Figure SI-111. ^{1}H-NMR (300 MHz, $CDCl_3$) and ^{13}C {^{1}H} NMR (75 MHz, $CDCl_3$) spectra of compound **13d**

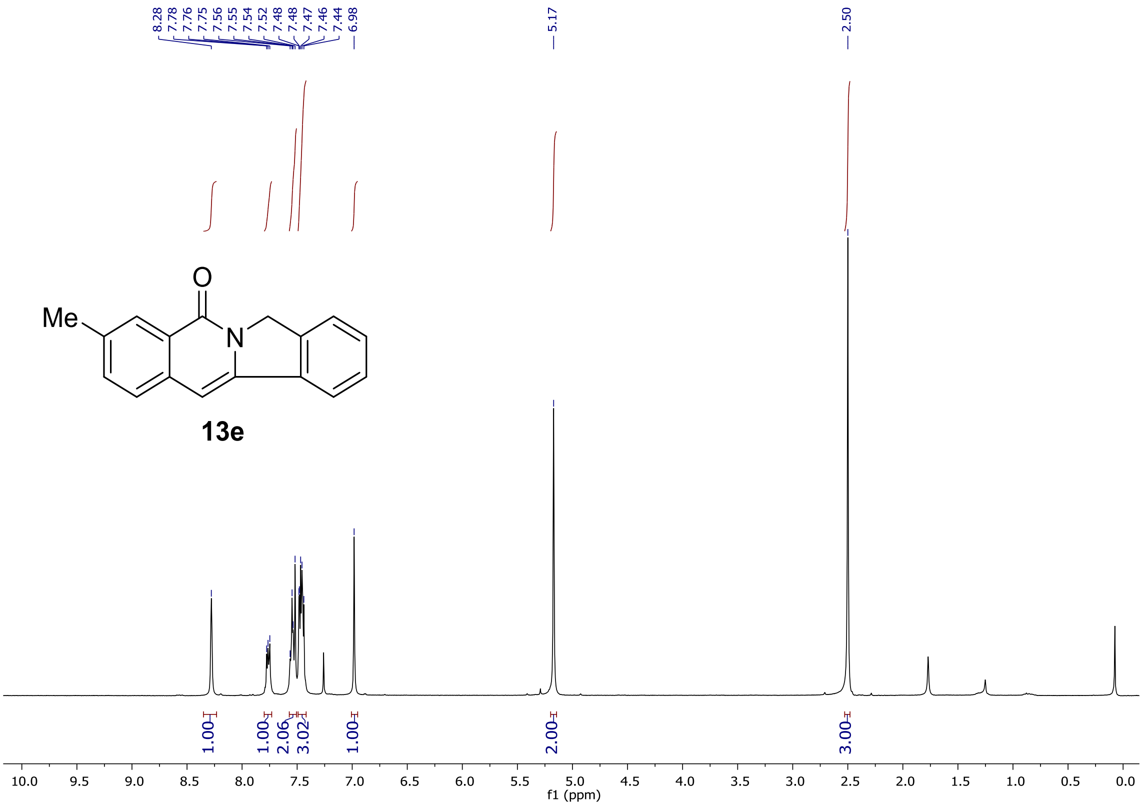

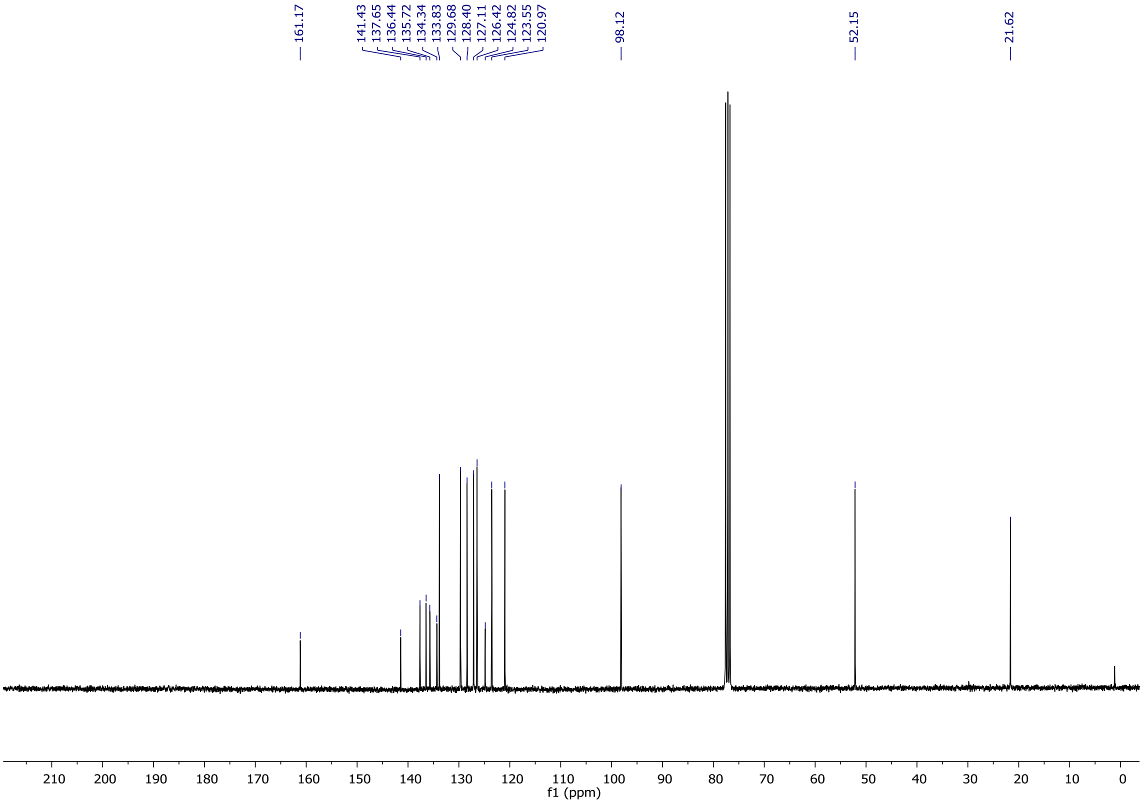

Figure SI-112. ^{1}H-NMR (300 MHz, $CDCl_3$) and ^{13}C {^{1}H} NMR (75 MHz, $CDCl_3$) spectra of compound **13e**

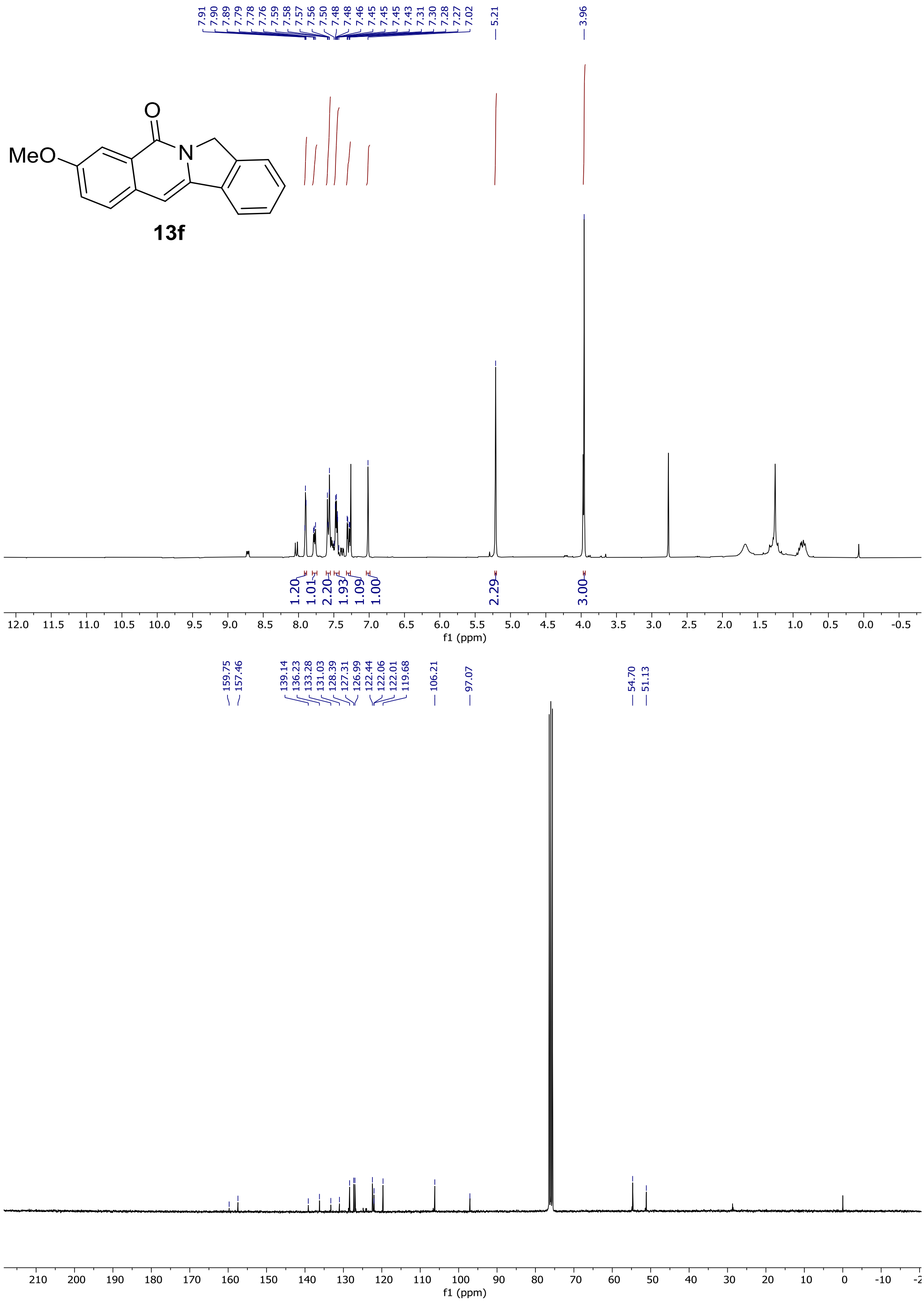

Figure SI-113. ^{1}H-NMR (300 MHz, $CDCl_3$) and ^{13}C {^{1}H} NMR (75 MHz, $CDCl_3$) spectra of compound **13f**

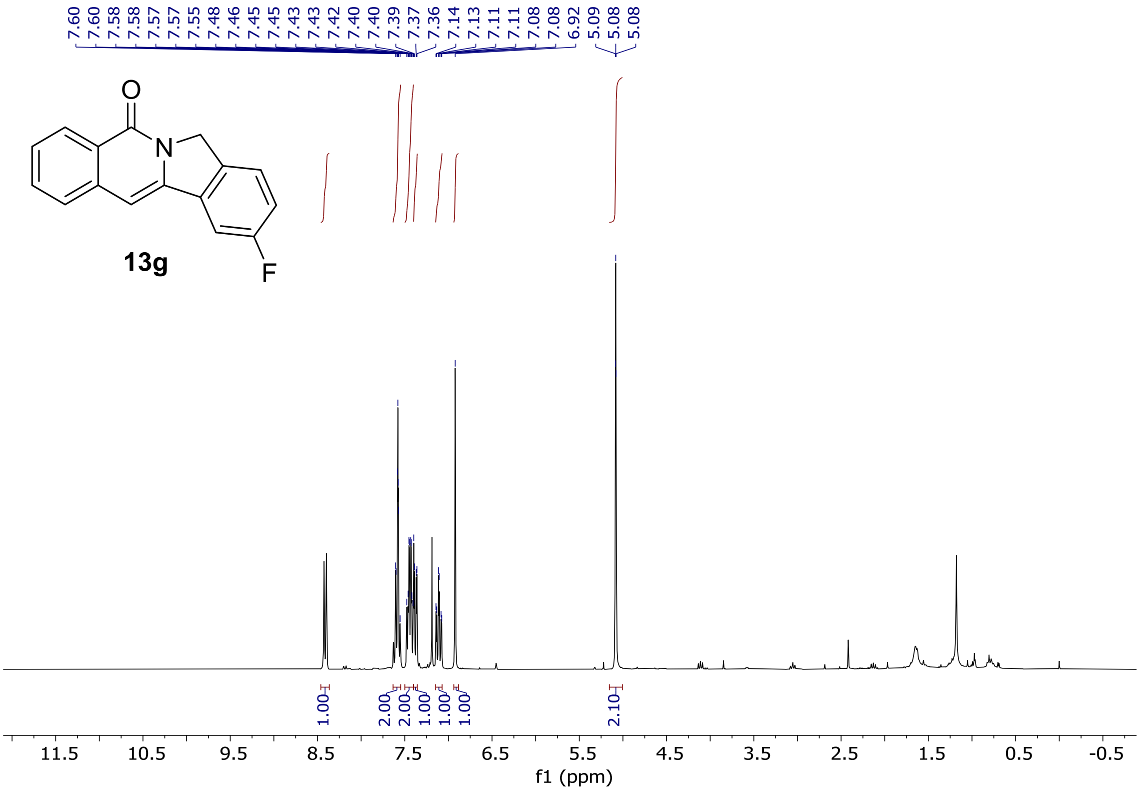

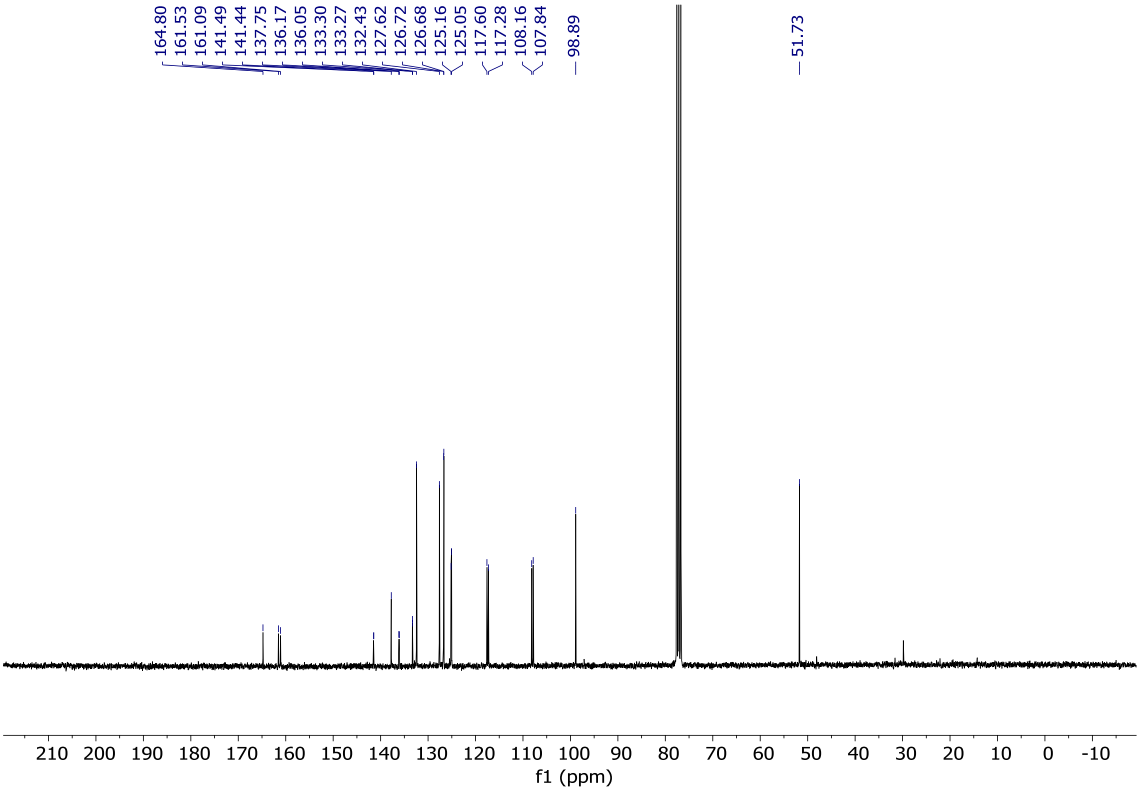

Figure SI-114. ^{1}H-NMR (300 MHz, $CDCl_3$) and ^{13}C {^{1}H} NMR (75 MHz, $CDCl_3$) spectra of compound **13g**

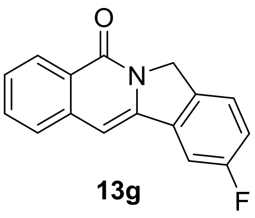

13g

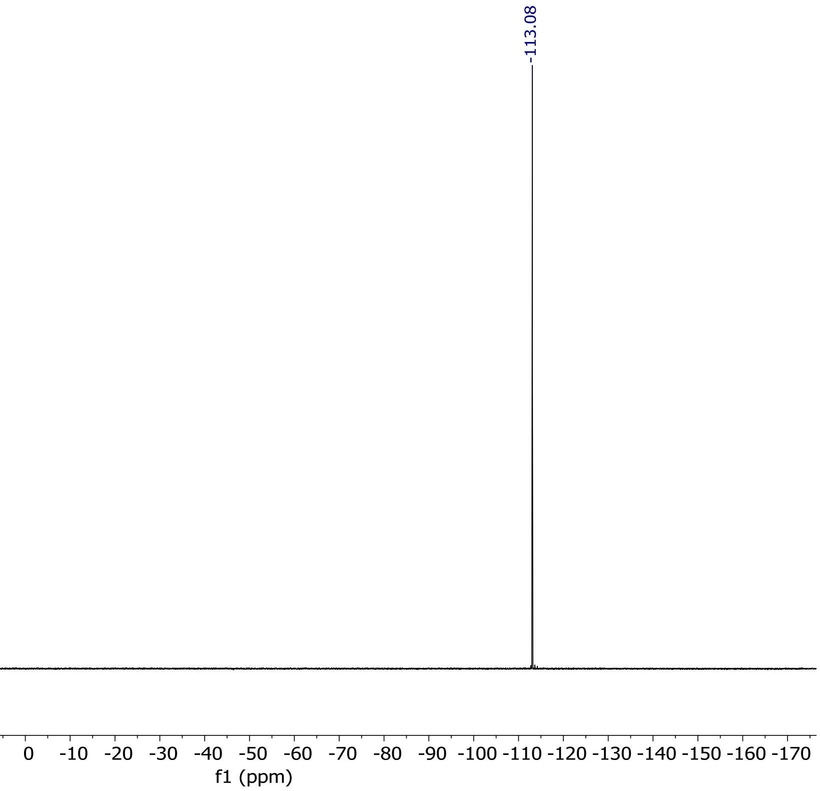

Figure SI-115. ^{19}F NMR (282 MHz, $CDCl_3$) spectrum of compound **13g**

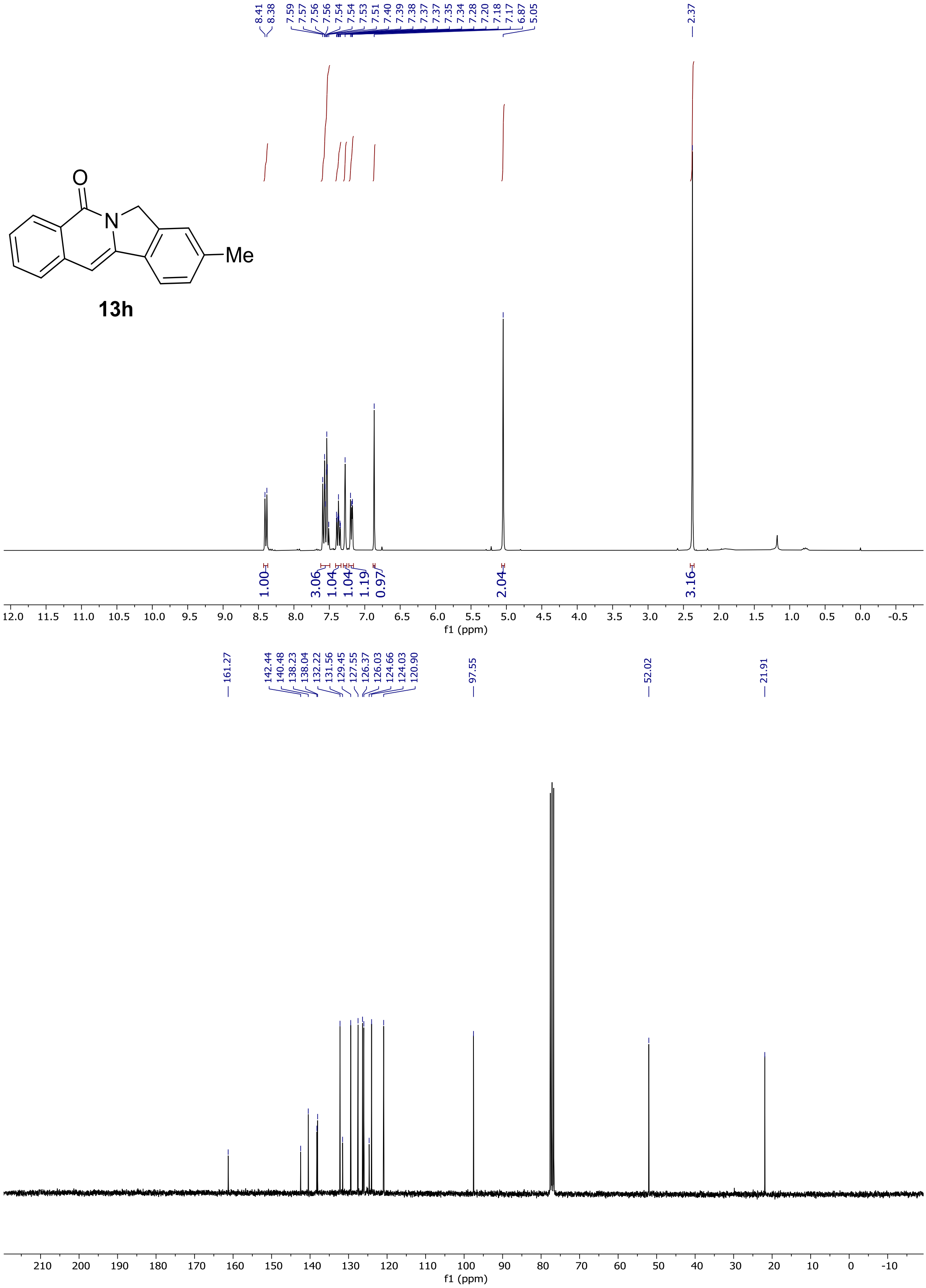

Figure SI-116. ^{1}H-NMR (300 MHz, $CDCl_3$) and ^{13}C {^{1}H} NMR (75 MHz, $CDCl_3$) spectra of compound **13h**
